# Genome sequence and comparative analysis of clavicipitaceous insect-pathogenic fungus *Aschersonia badia* with *Metarhizium* spp.

**DOI:** 10.1186/s12864-016-2710-6

**Published:** 2016-05-17

**Authors:** Yamini Agrawal, Tarun Narwani, Srikrishna Subramanian

**Affiliations:** CSIR-Institute of Microbial Technology, Sector 39-A, Chandigarh, 160036 India

**Keywords:** Chitinase, Destruxin, Host-specificity, Pathogen-host interactions

## Abstract

**Background:**

*Aschersonia badia* [(*Ab*) Teleomorph: *Hypocrella siamensis*] is an entomopathogenic fungus that specifically infects scale insects and whiteflies. We present the whole genome sequence of *Ab* and its comparison with two clavicipitaceous fungi *Metarhizium robertsii* (*MR*: generalist entomopathogen) and *M. acridum* (*MAC*: acridid-specific entomopathogen) that exhibit variable host preferences. Here, through comparative analysis of pathogen-host interacting genes, carbohydrate active enzymes, secondary metabolite biosynthesis genes, and sexuality genes, we explore the proteins with possible virulence functions in clavicipitaceous fungi. Comprehensive overview of GH18 family chitinases has been provided to decipher the role of chitinases in claviceptaceous fungi that are either host specific or generalists.

**Results:**

We report the 28.8 Mb draft genome of *Ab* and its comparative genome analysis with *MR* and *MAC*. The comparative analyses suggests expansion in pathogen-host interacting gene families and carbohydrate active enzyme families in *MR*, whilst their contraction in *Ab* and *MAC* genomes. The multi-modular NRPS gene (*dtxS1*) responsible for biosynthesis of the secondary metabolite destruxin in *MR* is not conserved in *Ab*, similar to the specialist pathogen *MAC*. An additional siderophore biosynthetic gene responsible for acquisition of iron was identified in *MR*. Further, the domain survey of chitinases suggest that the CBM50 (LysM) domains, which participate in chitin-binding functions, were not observed in *MAC*, but were present in *Ab* and *MR*. However, apparent differences in frequency of CBM50 domains associated with chitinases of *Ab* and *MR* was identified, where *MR* chitinases displayed a higher proportion of associated CBM50 domains than *Ab* chitinases.

**Conclusions:**

This study suggests differences in distribution of *dtxS1* and chitinases in specialists (*Ab* and *MAC*) and generalists (*MR*) fungi. Our analysis also suggests the presence of a siderophore biosynthetic gene in the *MR* genome which perhaps aids in enhanced virulence potential and host range. The variation in association of CBMs, being higher in generalists (*MR*) and lower in specialists (*Ab* and *MAC*) fungi may further be responsible for the differences in host affiliation.

**Electronic supplementary material:**

The online version of this article (doi:10.1186/s12864-016-2710-6) contains supplementary material, which is available to authorized users.

## Background

The fungal family Clavicipitaceae (Hypocreales, Ascomycota) includes many insect-pathogens such as *Metarhizium* spp. and these fungi have been exploited for their mycoinsecticidal abilities. Family Clavicipitaceae, as defined in modern taxonomy [Clavicipitaceae *sensu stricto* (*s.s.*)], includes three lineages specific to scale insects and whiteflies, referred to as *Hypocrella*, *Regiocrella* and *Torrubiella;* and one generalist lineage, referred to as *Metacordyceps* [1]. In this study, we have selected *Aschersonia badia* (*Ab*), an insect-pathogenic fungus belonging to the *Hypocrella* lineage for whole genome sequencing.

*Aschersonia* spp*.* are insect-pathogenic fungi that specifically infect whiteflies (Homoptera, Aleyrodidae) and scale insects (Homoptera, Coccidae). These are predominately found in tropical and sub-tropical areas. *Aschersonia* spp. [*A. aleyrodis* (Teleomorph: *Hypocrella libera*)] were one of the first fungal entomopathogens utilized as a biocontrol agent [2] and was reported as the cause of epizootics amongst whitefly populations in greenhouses and guava and citrus groves during the 20^th^ century in various parts of the world such as Azerbaijan, Bulgaria, China, Florida, Jamaica, Japan and Russia [3–6]. Moreover, *Aschersonia* spp. show adaptation to low relative humidity [7], perseverance on plant exteriors [8], and compatibility with insect parasitoids [9] in the management of whitefly pests. However, these fungi take a long time to grow in culture, and are not effective against all host stages and this has limited their successful exploitation against insect pests [10].

*Metarhizium* spp. (Clavicipitaceae *s.s.*, Metacordyceps) are well known entomopathogenic fungi and are best suited targets for biocontrol measures. *Metarhizium robertsii* (*MR*: previous name *M. anisopliae*) is a broad-spectrum insect pathogenic fungus that has been approved by the United States Environmental Protection Agency (USEPA) as an active ingredient for pest control [11, 12]. *M. acridum* (*MAC*) is a specialist pathogen against locust [13]. These diverse features of clavicipitaceous entomopathogens drive our interest towards comparison of *Ab* genome with the *MAC* and *MR* genomes that are adapted to different host ranges and lifestyles in order to get insights into the evolution of host affiliation of fungal entomopathogenicity.

Fungal entomopathogens are known to exhibit contact-based infection through the host cuticle unlike bacterial and viral entomopathogens which are required to be ingested by the target pest [14]. The primary structural component of host cuticle (arthropod exoskeleton) and cell walls of filamentous fungi is chitin which is the second most abundant natural biopolymer after cellulose. Enzymatic degradation is a crucial step in entry of the fungal spore into the insect body where chitinases play an important role in hydrolyzing the chitin-rich insect cuticle [15]. *Metarhizium* spp. are known to be prolific producers of chitinases, and many of the *MR* chitinases have been suggested to be involved in pathogenicity [16, 17]. However, in-depth analysis of chitinases and their relation with pathogenic lifestyles of clavicipitaceous entomopathogens have not been explored. Therefore, in the present study, we explore the diversity of chitinases in *Ab*, *MAC* and *MR*.

## Methods

### Fungal strain and maintenance

*Ab* strain MTCC 10142 was retrieved from the Microbial Type Culture Collection (MTCC), CSIR-Institute of Microbial Technology, Chandigarh, India. As per MTCC records, the *Ab* strain was isolated from Homopteran larva. Fungal culture was grown on potato dextrose agar medium and incubated at 25 °C for 20–25 days. DNA isolation from the fresh mycelia was performed using ZR Fungal/Bacterial DNA kit (Zymo Research, Catalogue number D6005) as per the instructions provided in user manual.

### Genome sequencing, assembly and annotation

2x100 paired-end shotgun sequencing (average insert size of 350 bp) of the *Ab* genome was performed using Illumina HiSeq 1000 technology at the Centre for Cellular and Molecular Platforms (C-CAMP) Bangalore, India. The reads were filtered using NGS QC Toolkit v2.2.3 [18] with length cutoff 70 % and base call quality greater than 20; reads were also trimmed for adapters using FastQC [19] and CLCbio wb6.0 genomics workbench (http://www.clcbio.com). The datasets were filtered for the presence of reads from bacterial DNA that may be present as contaminants. These were then further processed for genome assembly. The high quality reads were assembled into contigs using SPAdes 2.5.1 [20]. Before running SPAdes as an assembler, its 'read-error-correction’ module was used to rectify any orientation issues and InDels in the paired reads. Then its Assembly protocol was run at K-mer range 51–69 with step of +2 and ‘--careful’ option. The contigs obtained from these assemblies were scaffolded using SSPACE v2.0 [21]. Completeness measure was predicted using Core Eukaryotic Genes Mapping Approach (CEGMA) v2.5 with default parameters [22].

Genome annotation was performed using MAKER pipeline with Augustus as the gene predictor [23, 24]. *Fusarium graminearum* (*Fg*) was selected, as a model for gene calling, from the Augustus pre-trained dataset. EST and protein homology evidence of *Fg* was also provided to MAKER. The repeats were masked using RepeatMasker v4.0.3 (http://www.repeatmasker.org/). *MAC* CQMa 102 (PRJNA38715) and *MR* (PRJNA230500) genomes were reannotated using the same methodology for consistency. Both reannotated and the original set of genes/proteins from *MAC* and *MR* genomes were used for the computational analyses. The predicted proteins were subjected to BLASTp against the non-redundant (nr) database at the National Centre for Biotechnology Information (NCBI) using an E-value cutoff of 10^−5^. The draft genome of *Ab* (*Hypocrella siamensis*) was deposited at NCBI-GenBank under the accession number JMQE00000000.

### Phylogenetic analysis

To validate the phylogenetic position of *Ab*, *MAC* and *MR*, four gene markers: mitochondrial ATP6 (*atp6*), the largest and second largest subunits of RNA polymerase ІІ (*rpb1* and *rpbII*) and β-tubulin (*tub*) genes were retrieved from the *Ab*, *MAC* and *MR* genomes and were concatenated. These concatenated sequences were included in the four gene concatenated sequence dataset of hypocrealean fungi [1]. Maximum Likelihood (ML), Maximum Parsimony (MP) and Neighbor-Joining (NJ) phylogenetic reconstruction was performed using Randomized Axelerated Maximum Likelihood (RAxML) v8.0 [25], Phylogenetic Analysis Using Parsimony (PAUP) v4.0 [26] and Molecular Evolutionary Genetics Analysis (MEGA) v6.0 [27], respectively. Ambiguously aligned regions were excluded from the analysis using the trimAl tool [28]. Bootstrap analysis with 1000 replicates was performed.

### Cluster of orthologous (COG) groups

The COG clusters were created by subjecting the predicted proteomes to BLASTp against the COG database [29] 2014 update (http://www.ncbi.nlm.nih.gov/COG/) with an E-value threshold of 10^−5^. For each COG mapping, functional category assignment was given based on COG category letter associations. Reciprocal best BLAST hits (E-value threshold of 10^−10^) obtained using PERL script of Proteinortho v2.0 [30] were used to characterize the orthologous proteins among all three genomes. Computational Analysis of gene Family Evolution (CAFE) v3.0 [31] was used to estimate the gene family expansions and contractions in *Ab*, *MAC* and *MR* for the CAZymes glycoside hydrolases (GHs, 55 families), glycosyltransferases (GTs, 37 families), carbohydrate esterases (CEs, 10 families), auxiliary activities (AAs, 10 families) and carbohydrate binding modules (CBMs, 17 families) and the trasposase gene families (DNA transposase gene families: 10, Retrotransposase gene families: 7). ML Newick tree prepared through MEGA was used as an input for CAFE. *P-*value cut-off of 0.01 was selected to calculate the significant changes in gene numbers.

### Protein domain search

Secondary metabolite biosynthetic clusters were identified by using the predicted proteome as query on the antiSMASH server [32]. Pathogen Host interacting (PHI) partners were identified by subjecting the predicted proteomes to BLASTp against the PHI database [33] v3.6 with an E-value threshold of 10^−5^. The domain architecture of chitinases and destruxins were annotated using the Pfam v27.0 and SMART v7.0 databases [34–36]. Molecular weight was evaluated using the Protparam tool [37].

### Phylogenetic analysis of non ribosomal peptide synthase (NRPS) biosynthetic gene types

NRPS genes identified from the three genomes *Ab*, *MAC* and *MR* were combined with the phylogenomic NRPS dataset reported previously [38]. Their phylogenetic analysis was performed as described above. Multiple sequence alignment file used for the phylogenetic analysis is provided as Additional file 1 (A, B).

Sequence similarity of destruxin gene was calculated using SIAS server (http://imed.med.ucm.es/Tools/sias.html). After running BLASTp [Query: dtxS1 (*MR*), Database: Ab_Proteome], the alignments of interest (*dtxS1* and *Ab-Node74-Gene7905*) were uploaded in SIAS server and % identities and % similarities were obtained.

### Carbohydrate active enzymes (CAZy) search

CAZY classes Glycoside hydrolases (GHs), glycosyltransferases (GTs), polysaccharide lyases (PLs), carbohydrate esterases (CEs), auxiliary activities (AAs) and carbohydrate binding modules (CBMs) were searched from the CAZy database. CAZy families were assigned to the proteins by subjecting the predicted proteome of all the three genomes to dbCAN web server [39].

### Transposable elements (TEs) and Repeat induced point mutation (RIP) analysis

TEs were classified by subjecting the genome sequences of *Ab, MAC* and *MR* to BLASTn against the Repbase (http://girinst.org) libraries of RepeatMasker (http://www.repeatmasker.org). RIP indices were estimated using the RIPCAL v1.0 [40].

### Identification of MAT idiomorphs

Mating type genes control sexual development in fungi [41]. Putative mating type loci were identified by the presence of conserved domains and the sequence similarities to the corresponding MAT genes in other filamentous fungi. Conserved alpha domain, Proline–Proline–Phenylalanine domain, High–Mobility–Group (HMG) domain with a DNA binding site and MatA HMG box with a DNA binding site were searched to identify the mating type loci MAT1-1-1, MAT1-1-2, MAT1-1-3 and MAT1-2-1, respectively [42, 43]. Synteny for the mating type locus among all the three genomes was analyzed by locating the position of MAT locus and their flanking genes. The absence of any gene was reconfirmed by mapping back the reads on these genes from related fungi such as *Metarhizium majus*, *Trichoderma reesei*, *Fusarium graminearum*, *Beauveria bassiana*, *Cordyceps militaris*, *Ophiocordyceps sinensis*, *Hirsutella thompsonii*, *Isaria farinose*, *etc*. This was done mainly to verify that they were not missing in the three genomes due to assembly errors.

### Glycoside hydrolase family 18 (GH18) chitinases search

The chitinase sequences were searched in the predicted proteomes of *Ab*, *MAC* and *MR* genomes using ProfileScan (http://www.csd.hku.hk/bruhk/gcgdoc/profilescan.html) with the conserved active site signature motif [LIVMF]-[DN]-G-[LIVMF]-[DN]-[LIVMF]-[DN]-x-E (Prosite No. PS01095) [44]. The identified chitinase sequences from the three genomes were aligned using PCMA [45] and profile hidden Markov model was built using HMMER v3.1b1 [46]. This HMMER profile was further used to scan the predicted proteome of the genomes to find other chitinase sequences that have mutations/SNPs at conserved consensus motifs. GH18 chitinase sequences of fungal origin were selected from a previously reported dataset [47]. All retrieved chitinases from the three genomes and GH18 family chitinase sequences of fungal origin were assembled and aligned using MAFFT v7.0 [48]. For subgroup A, B and C fungal chitinases, three separate alignment files were prepared as previously described [47, 49, 50] (Additional file 2).

## Results

### Phylogenetic relationships of *Ab* and *Metarhizium* species

The phylogenetic analysis grouped *Ab*, *MAC* and *MR* within the Clavicipitaceae *s.s.* clade (Additional file 3: Figure S1). *Ab* MTCC 10142 strain was clustered within the *Hypocrella* clade whereas *MAC* and *MR* were clustered within *Metacordyceps* clade (ML, MP and NJ analysis parameters presented 100 % support values) thus validating the phylogenetic identification of the organisms under study. The phylogenetic tree displayed the paraphyletic occurrence of family Clavicipitaceae into clades A, B and C, *viz.*, Clavicipitaceae *s.s.*, Ophiocordycipitaceae and Cordycipitaceae, respectively and the grouping of Clavicipitaceae *s.s.* clade into four lineages: three specific to scale insects and whiteflies, *Hypocrella*, *Torrubiella* and *Claviceps*; with one as a generalist, *Metacordyceps* (Additional file 3: Figure S1).

### Genome features

The Illumina HiSeq 1000 shotgun sequencing of *Ab* genome resulted in 12,732,904 paired-end (PE) reads (insert size of 350 bp and length 101 bp). A total of 11,990,083 high-quality reads after filtering and trimming were assembled into 2,034 contigs (maximum contig length as 246 Kb and minimum as 600 bp) with a coverage of 90x. Genome size as assembled with Illumina PE data was 28.8 Mb (N50: 27770). Assembly of the core genomic regions was predicted to be 94 % complete based on CEGMA analysis. Table 1 lists the genome sequencing, assembly and annotated features for *Ab*. The total number of genes obtained from *Ab*, *MAC* and *MR* genomes was 9292, 10853 and 12880, respectively. The core gene pool is represented by 5586 genes, common in all three genomes and the predicted unique gene content for *Ab*, *MAC* and *MR* was 3402, 3352 and 5247 genes, respectively (Additional file 3: Figure S2A). A comparative distribution of functional features of predicted proteins suggests that about 18 % function in cellular processes and signaling, 19 % in information storage and processing, and 36–39 % in metabolism (Additional file 3: Figure S2B).

**Table 1:** Genome sequencing, assembly and annotated features

Features	*Ab*
Genome Size	28.8 Mbp
PE reads	12,732,904
High quality reads	11,990,083
N50 contigs	27,770
Contigs	2,034
Max. contig length	246 Kbp
Min. contig length	600 bp
Coverage	90x
Total length	28,856,701
GC (%)	53.1
Total genes annotated	9,292

### Genes involved in biosynthesis of secondary metabolites

Secondary metabolites are quite diverse in fungi and vary substantially in their structure and biological activity. Some of the secondary metabolites secreted by fungi aid in pathogenicity mechanisms and toxicity reactions while others are useful for therapeutic purposes [51]. The most common gene types involved in their synthesis include NRPS: non-ribosomal peptide synthases, PKS: polyketide synthases, and TS: terpene synthases. All the predicted genes involved in synthesis of secondary metabolites were identified from the three genomes where *MR* shows higher abundance of these predicted genes than *Ab* and *MAC* genomes (Table 2). One of the appealing observations is the presence of siderophores in the *MR* genome (Table 2) that could perhaps be related to its enhanced pathogenicity and host range [52–54].

**Table 2:** Gene types involved in the biosynthesis of secondary metabolites in *Ab*, *MAC* and *MR* genomes

Gene type	*Ab*	*MAC*	*MR*
NRPS	9	13	15
NRPS-T1PKS	5	4	4
NRPS-Terpene	0	2	0
NRPS-T1PKS-Terpene	0	1	1
T1PKS	11	10	16
T3PKS	1	0	0
Unusual HglD/E-like PKS	2	0	1
Terpene	7	6	13
Terpene-T1PKS	1	0	2
Other	8	5	13
Siderophore	0	0	1
Total	44	41	66

NRPS are modular enzymes involved in the biosynthesis of diverse low molecular weight bioactive metabolites that participate in various fundamental metabolic functions of fungi such as growth and development, virulence, niche specific functions, stress responses, *etc.* [38, 55]. A set of three core domains (A-T-C) forms the basic module of NRPS where A is adenylation domain for recognizing and activating the substrate (initiation), T or PCP is thiolation domain or peptidyl carrier protein (PCP) for binding and transferring the activated substrate (elongation) and C is the condensation domain for forming peptide bonds between elongated amino acid (aa) chain (termination) [38, 56]. Additionally, accessory domains like epimerization (E) domain responsible for altering the aa configuration from L to D form, N-methylation domain (nMT) responsible for transfer of the methyl group to the substrate may also be present in the NRPS module [38, 57]. The number and order of these modules on each NRPS genes is known to govern the structural variability of the resulting product [38, 58, 59].

**Fig. 1:**
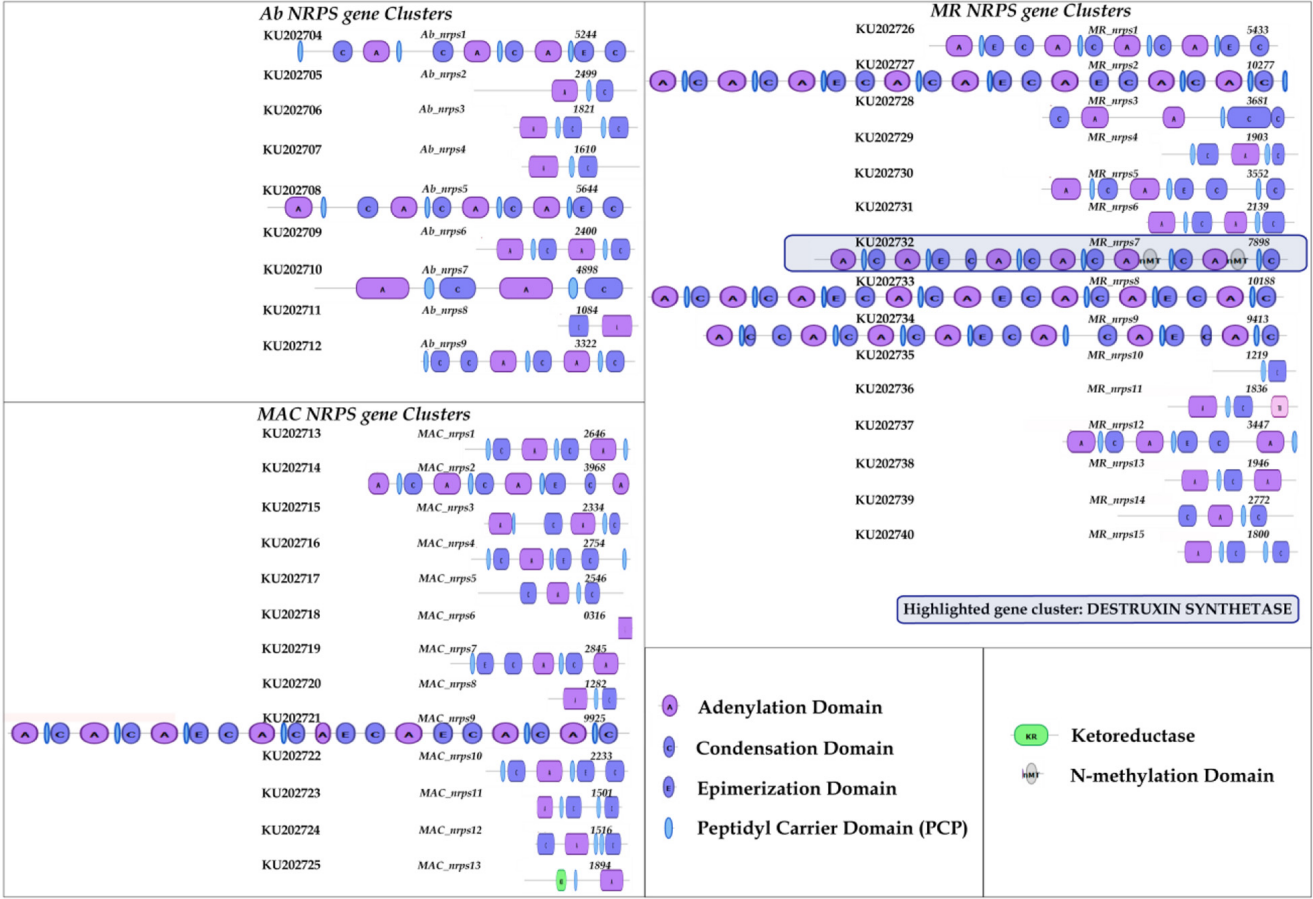
Secondary metabolite biosynthetic NRPS genes and their domain distributions in *Ab, MAC* and *MR* genomes, as identified by antiSMASH server. A total of 9, 13 and 15 NRPS genes were identified in *Ab, MAC* and *MR* genomes. The core module of NRPS contains three domains (A-T-C), A: adenylation domain for initiation, T/PCP: thiolation domain/peptidyl carrier protein for elongation, and C: condensation domain for termination. The hexa-modular structure of NRPS gene (along with nMT in each of the last two A-T-C modules) encodes destruxin synthetase (dtxS1), an insecticidal host-specific gene. This hexa-modular structure of NRPS gene and nMT are only seen in *MR* highlighted gene, suggesting presence of *dtxS1* in *MR* genome while its absence in *Ab* and *MAC* genomes. GenBank accession number of each gene is shown on left side of the gene schematic. The numbers by the end of each gene schematic denote the gene length (amino acids)

The domain architecture of all predicted NRPS genes in the three genomes were compared (Fig. 1). These genes were nomenclatured as *X_nrps-z*, where *X* is the organism label (*Ab*, *MAC* or *MR*) and *z* is the designated number. Conservation of basic A-T-C domains was seen in all predicted genes, with the exception of *Ab*_nrps8, *MAC*_nrps6, *MAC*_nrps13 and *MR*_nrps10. *MR* genome contained many multimodular NRPS genes in comparison to the other two genomes suggesting their evolution to perform niche adaptation functions [38]. One such insecticidal host-specific multimodular gene is destruxin synthetase (*dtxS1*), which is reported selectively in the *MR* genome, and is absent in the *MAC* genome [58–60]. This *dtxS1* contains the hexa-modular structure of NRPS (along with nMT domain in each of the last two A-T-C modules) and is postulated to be one of the factors in evolution of fungal affiliation to diverse host niches [59, 60]. In the present analysis, none of the NRPS genes was observed to have six A-T-C modules in *Ab* and *MAC*. Further, no nMT in conjugation with A-T-C module was observed, suggesting the absence of this gene in the specialist pathogen *Ab*, as reported previously for *MAC* [58, 59]. However to further confirm this, *Ab* gene homologous to the *dtxS1* gene of *MR* was identified based on sequence similarity in the *Ab* genome. BLASTp indicated 81 % similarity (31 % identity) of *Ab*-Node74 gene-7905 (*Ab*-nrps-7905 (~5.2 kb) with *dtxS1* gene (~8 kb) of *MR*. No similar gene in *MAC* was identified. Therefore, domains of *MR dtxS1* gene and *Ab*-nrps-7905 were compared. It revealed presence of only three complete A-T-C modules in *Ab*-nrps-7905 that cannot be referred to as destruxin synthetase.

**Fig. 2:**
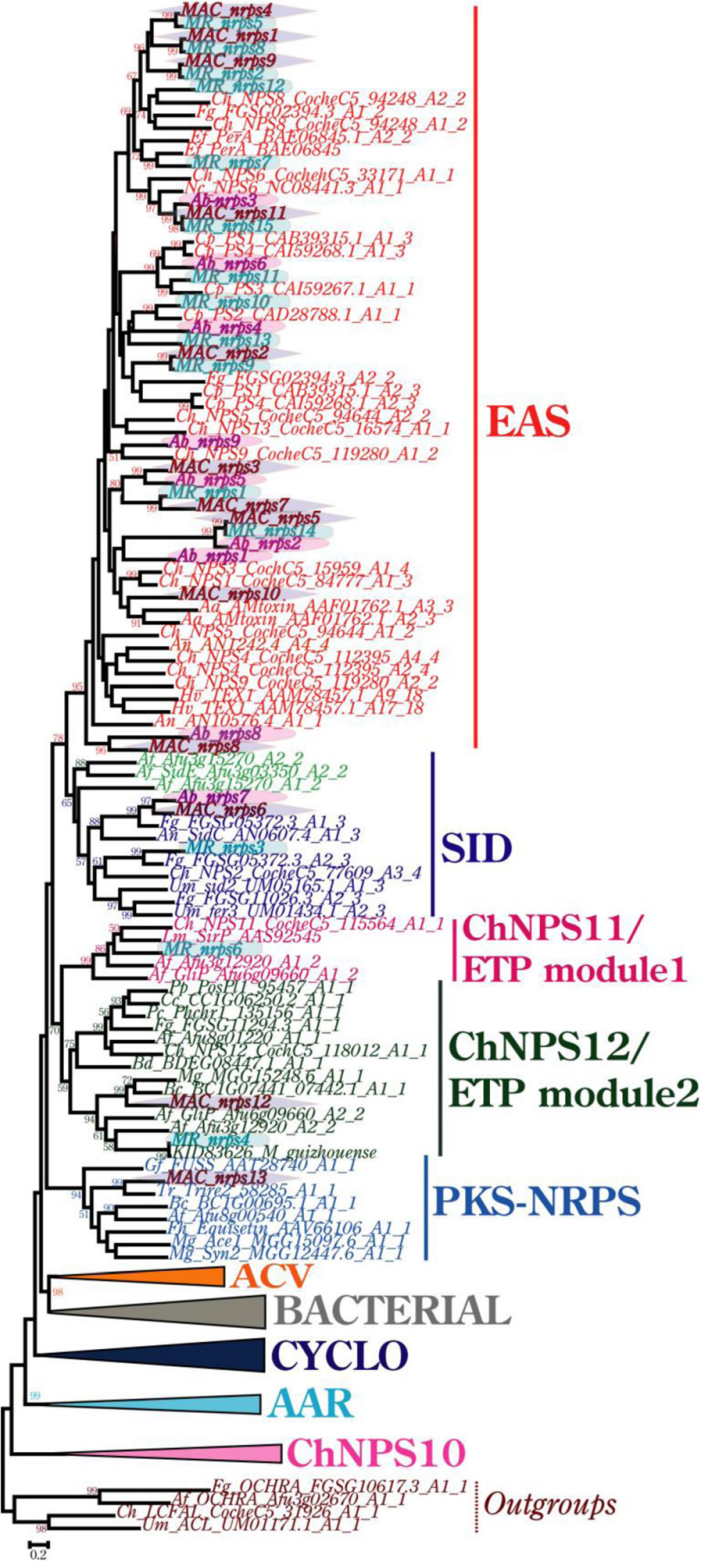
ML phylogenetic tree of NRPS gene types. Bootstrap support (>50 %) is shown over the branches. NRPS gene sequences obtained from *Ab*, *MAC* and *MR* genomes are highlighted and shaded. The evolutionary tree is drawn to scale with distances in the units of the number of amino acid substitutions per site. EAS (Euascomycete clade synthetases) and SID (siderophore synthetases) subclasses are multimodular. The remaining are mono/bi-modular NRPS types, where fungal NRPS from the three genomes clustered in ChNPS11/ETP module 1 toxin-like synthetases, ChNPS12/ETP module 2 toxin-like synthetases and PKS-NRPS hybrid synthetases. Clusters ACV (ACV synthetases), Bacterial, CYCLO (cyclosporin synthetases), AAR (α-aminoadipate reductases) and ChNPS10-like synthetases are collapsed to improve readability since they have no members from the species under investigation. Full phylogeny is available in Additional file 3: Figure S3

Phylogenetic relationships of predicted NRPS genes in *Ab*, *MAC* and *MR* were further assessed to identify their orthologous genes and to trace their duplication and divergence history (Fig. 2, S3). List of predicted NRPS gene cluster sequences identified in *Ab*, *MAC* and *MR* genomes is provided in Additional file 4. Fungal NRPS have been classified into two main groups: mono/bimodular NRPS of ancient origin, involving bacterial and fungal NRPS (subfamilies clustered: α-aminoadipate reductases (AAR), ChNPS10-like synthetases, ChNPS11/ETP toxin module 1 synthetases, ChNPS12/ETP toxin module 2 synthetases, Cyclosporin synthetases (CYCLO) and PKS-NRPS); and multimodular NRPS of recent origin, involving exclusively fungal NRPS (Subfamilies clustered: Euascomycete-only synthetases (EAS) and siderophore synthetases (SID)) [38]. In the present phylogenomic analysis, we observed the clustering of predicted genes from the three genomes mostly in the EAS subfamily that is specific for the ascomycete lineage (Fig. 2). One gene each from *Ab*, *MAC* and *MR* (*Ab_nrps7*, *MAC_nrps6* and *MR_nrps3*) grouped in the SID subfamily (Fig. 2), responsible for iron uptake [61]. Both EAS and SID subfamilies are observed to exhibit low conservation of their domain architecture owing to autonomous domain gain/loss mechanisms that likely aid in specialized niche adaptive roles [38]. *MR_nrps6* was observed to cluster with ChNPS11/ETP toxin module 1 synthetases whereas *MAC_nrps12* and *MR_nrps4* were clustered with ChNPS12/ETP toxin module 2 synthetases subfamilies (Fig. 2).

### Pathogen-host interacting (PHI) genes

**Fig. 3:**
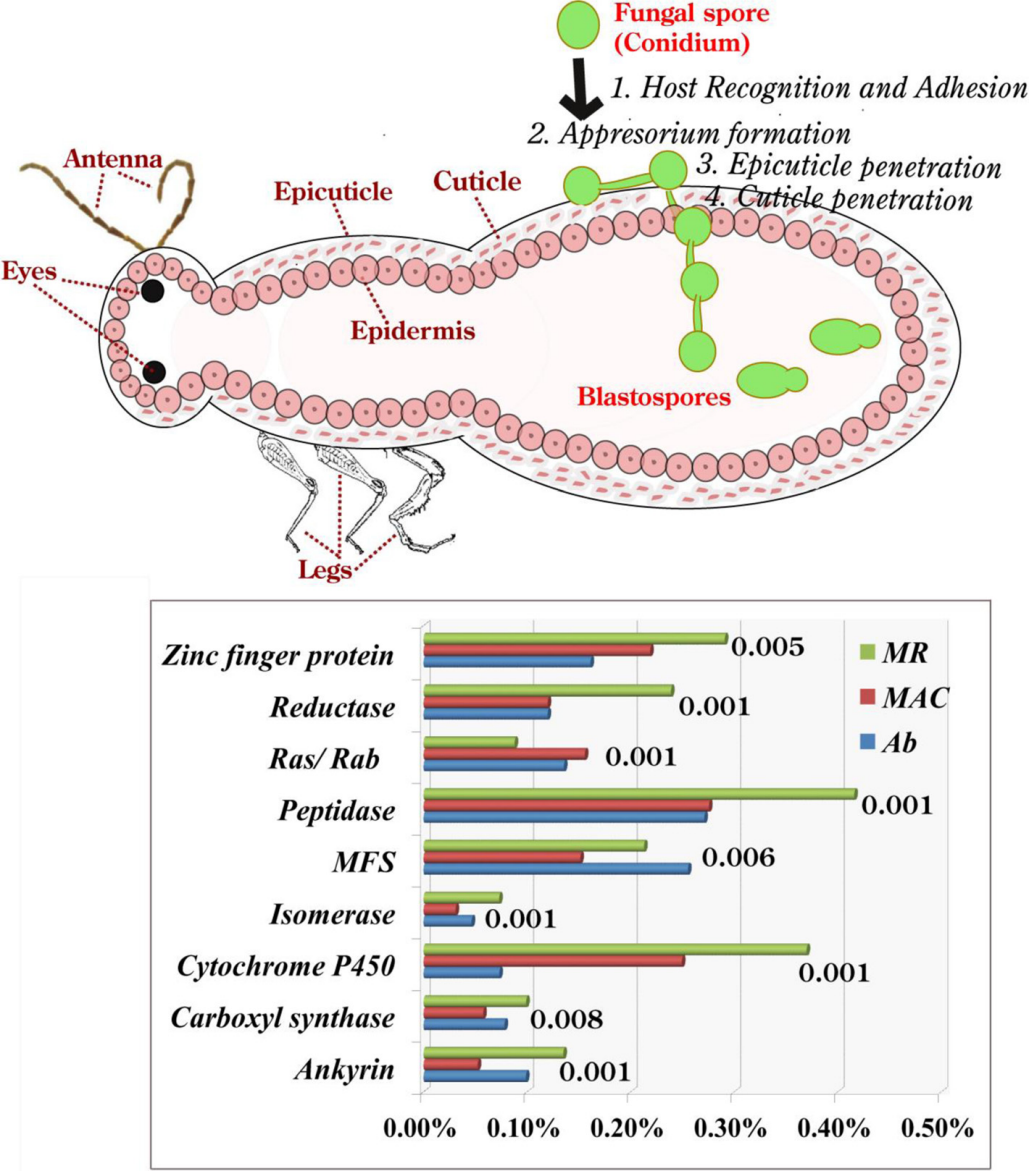
Pictorial illustration of the principal steps involved in fungal-arthropod interactions and the comparative distribution of statistically significant PHI genes (*P*-value < 0.01), identified from *Ab*, *MAC* and *MR* genomes. *P*-values are mentioned adjacent to each PHI gene bar. X-axis represents the genomic % of each PHI gene and Y-axis: statistically significant PHI gene matches

The identification of entomopathogenicity-related genes is important in order to understand the pathogenicity mechanisms of the fungal taxa that could be explored in development of mycoinsecticidal strategies. PHI database (http://www.phi-base.org/) catalogues 3944 genes and 6473 interactions (http://www-phi4.phibase.org/releaseNote.htm) to be involved in pathogenicity. In our analysis, we obtained 1901, 2093 and 2378 of PHI genes from *Ab*, *MAC* and *MR* genomes respectively. Figure 3 shows the important steps in pathogen-host interactions and the distribution of statistically significant (not evolved due to random chance) PHI genes (with CAFE *P*-value < 0.01) in the three genomes as evidenced from the CAFE analysis. PHI genes are known to be involved in host recognition, signaling, adhesion, appresoria development, formation of infection structures, host colonization, conidiation, spore germination, cuticle penetration, nutrition, *etc.* [62–66]. We identified genes with matches to those in PHIbase that have demonstrated roles in insect-pathogenicity in the three genomes. The detail of each PHI gene identified is provided in Additional file 5. Our analyses suggest a variability in the distribution of these genes in the three genomes, indicating a contraction of PHI families in *Ab* (CAFE average expansion is negative = –1.45) and *MAC* (CAFE average expansion is negative = –0.88), whereas they are significantly expanded in *MR* (CAFE average expansion = 1.46) as suggested by previous reports where also larger gene families were observed in generalists genomes as compared to the specialists genomes due to dynamic loss and gain of genes [13, 60].

### Carbohydrate active enzymes

A total of six classes of carbohydrate active enzymes as provided in CAZy database have been compared. The genes from each class: carbohydrate binding modules (CBMs), glycoside hydrolases (GHs), glycosyltransferases (GTs), polysaccharide lyases (PLs), carbohydrate esterase (CE), and auxiliary activities (AAs) are provided in Additional files 6, 7, 8, 9, 10 and 11, respectively.

The prevalence of associated functional modules, CBMs was found to be quite variable in the three fungal genomes (Additional file 6). CBM38 (inulin binding function) and CBM54 (xylan, glucan and chitin binding function) were observed to be unique to *Ab*. CBM1 (cellulose binding domain) family members were observed in *MAC* and *MR.* CBM18 and CBM50 (LysM) family modules known to be associated with chitinase catalytic domains and are implicated to bind chitin were found to be commonly present in all three genomes. However, their frequency was lowest in *MAC* genome. CAFE analysis suggests a contraction of CBM families in *Ab* and *MAC* genomes, whereas they are expanded in the *MR* genome.

GHs, GTs, PLs, CEs and AAs are the catalytic enzyme classes that direct the lysis, synthesis or alteration of carbohydrate moieties [67–70]. The three genomes show the predominance of GH16 (xyloglucan: xyloglucosyltransferase), GH18 (chitinase) and GH76 (α–1,6-mannanase) family enzymes (Additional file 7). GH9 (endoglucanase) and GH120 (β-xylosidase) were observed to be unique to the *Ab* genome. GH54 (α-L-arabinofuranosidase; β-xylosidase), GH88 (d-4,5-unsaturated β-glucuronyl hydrolase), GH95 (fucosidase) and GH117 (α-1,3-L-neoagarooligosaccharide hydrolase) were exclusive to the *MR* genome. GT2 (cellulose/chitin synthase) and GT32 (α-1,6-mannosyltransferase/N-acetylglucosaminyltransferase) were commonly observed in the three genomes (Additional file 8). *Ab* displayed exclusive occurrence of PL10 (pectate lyase) family enzyme, whereas *MAC* and *MR* displayed PL7 (alginate lyase), PL8 (hyaluronate lyase) and PL20 (endo-β-1,4-glucuronan lyase) family enzymes (Additional file 9). CE1 (acetyl xylan esterase; carboxylesterase) and CE10 (arylesterase; carboxyl esterase; acetylcholinesterase; sterol esterase) family enzymes were predominantly present in all the three genomes (Additional file 10). AA3 (glucose-methanol-choline (GMC) oxidoreductases) and AA7 (oligosaccharide oxidase) were the most frequent families in the three genomes (Additional file 11). CAFE analysis indicated the contraction of CAZY families (GHs, GTs, CEs and AAs) in *Ab* and *MAC* genomes, whereas their expansion in *MR* genome.

### Repeat induced point mutation (RIP) and diversity of transposable elements (TE)

Repeat-induced point mutation (RIP) is a protection system in fungal genomes that operates on duplicated sequences and checks the development of TEs through hypermutation [71, 72]. A genome-wide RIP analysis was performed on the *Ab*, *MAC* and *MR* genomes. Summary of RIP signatures predicted in the three genomes is provided in Additional file 12. RIP frequency index (CpA + TpG)/(ApC + GpT) was calculated as 0.72 in *Ab*, 0.94 in *MAC* and 1.06 in *MR* (Additional file 13), where the threshold was 1.03 and the RIP index ≤ 1.03 indicated RIP activity. Therefore, *Ab* and *MAC* genomes were suggested to present significant RIP activity consistent with earlier reports supporting occurrence of RIPs in specialists genome whereas their absence from non-specialists genomes [13, 60]. The frequency of putative transposase genes suggests their expansion in *MR* genome as compared to the *Ab* and *MAC* genomes (Additional file 14). This observed difference is suggested to be related to the effect of RIP activity [13, 60].

### Mating type

**Fig. 4:**
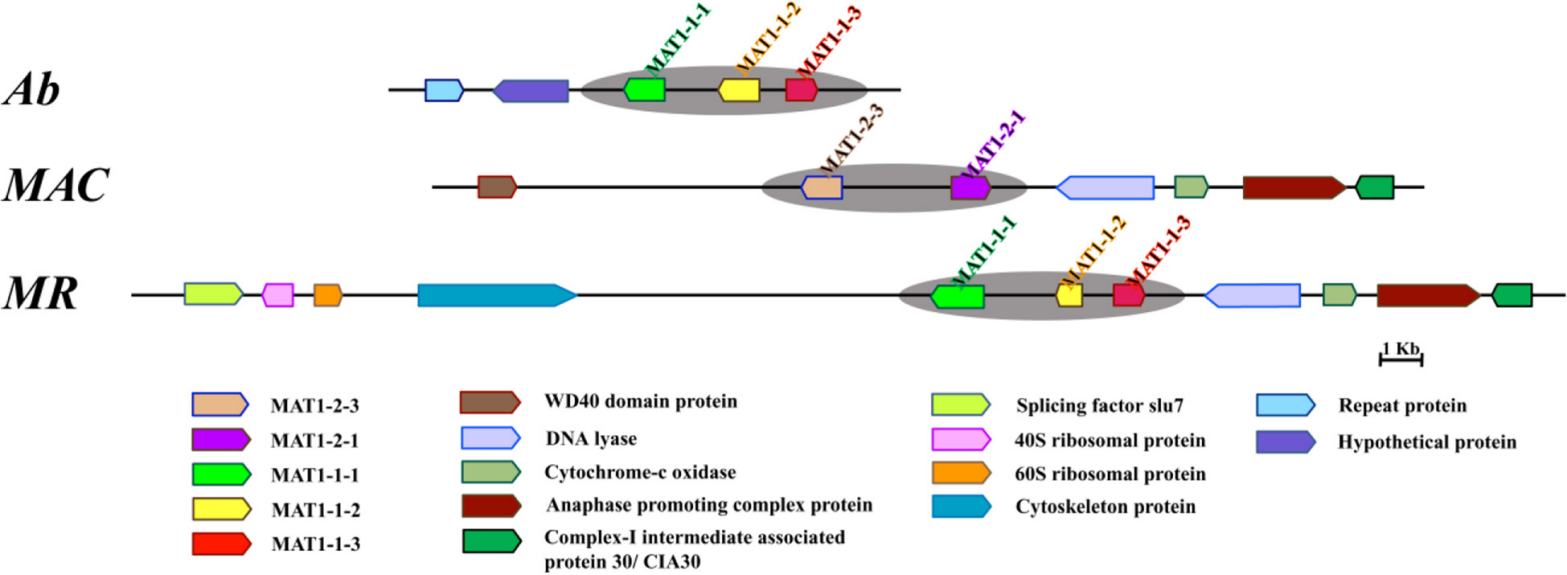
Synteny of mating-type loci and their flanking regions in *Ab*, *MAC* and *MR* genomes. MAT1 loci (highlighted with grey shading) and their flanking gene regions showing orthologous relationships are marked with the same color

The three genomes show a variety of common fungal mating genes (Additional file 15). The mating type genes MAT1-1-1, MAT1-1-2 and MAT1-1-3 were identified in *Ab* and *MR*. Interestingly, syntenic analysis revealed the presence of mating type genes MAT1-2-3 and MAT1-2-1 in place of MAT1-1-1, MAT1-1-2 and MAT1-1-3 in *MAC* and the conservation of genes flanking the MAT idiomorph in *MAC* and *MR* (Fig. 4). The mating locus MAT1-2-3 was initially reported in the genus *Fusarium* (Teleomorph: *Gibberella*) (*Nectriaceae*, *Hypocreales*) [73] that was recognized based on its expression profile, displaying upregulation during the sexual developmental process similar to other MAT loci. However, no known functional domain was identified in this MAT1-2-3 locus. Therefore, in *MAC* genome, this putative MAT idiomorph was identified based on sequence similarity with *Fusarium* MAT1-2-3 locus. *Ab* is already known to be sexually fertile based on the detection of its teleomorph *Hypocrella siamensis* [74].

### Phylogenetic classification of putative cuticle degrading chitinases

Fungal chitinases are known to belong exclusively to the GH18 family [75–77]. Based on amino acid sequence similarity of their GH18 domains [49, 77], these have been classified into three subgroups A, B and C. These display differences in their enzymatic properties—exo for sg-A and sg-C, whereas endo for sg-B; the presence or absence of carbohydrate binding modules (CBMs), absent in sg-A, whereas present in sg-B at C-terminal and in sg-C at N-terminal—and the architecture of their substrate binding cleft [75–77]. In this study, we identified 16, 23 and 28 chitinase genes in *Ab*, *MAC* and *MR* genomes, respectively. Their properties are presented in Additional file 16. These chitinases were nomenclatured based on their increasing molecular weights and are represented as *X Chit-z*, where *X* is the organism label (*Ab*, *MAC* or *MR*) and *z* is the designated number. A few of the *MAC* and *MR* chitinases were named on the basis of sequence similarity between *MAC* and *MR* chitinases. The three datasets, *viz.*, subgroup A, B and C were analyzed to reconstruct their phylogenetic relationships.

#### Subgroup-A (sg-A) dataset

**Fig. 5:**
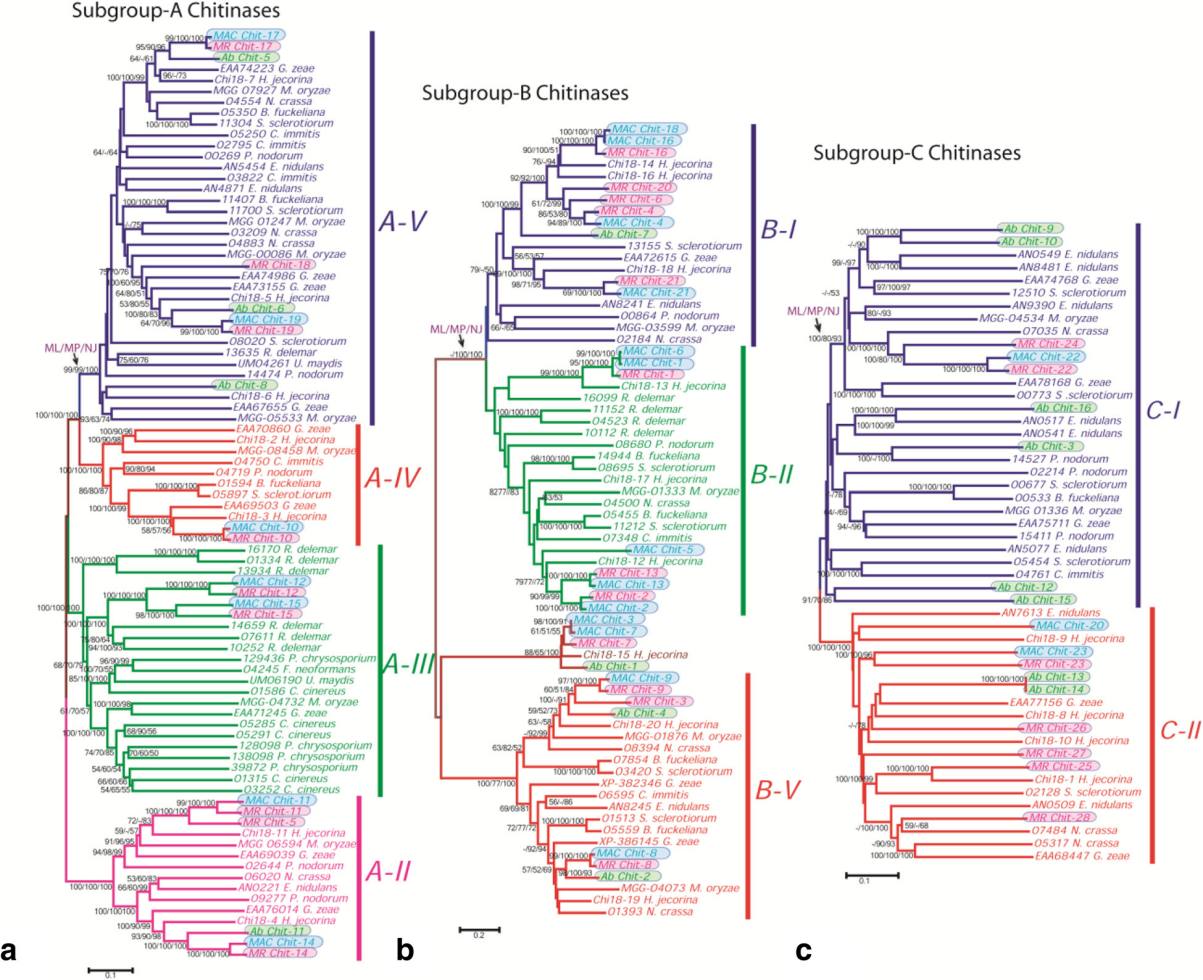
Phylogenetic relationships of subgroups A, B and C chitinases (**a**–**c**). The series of values over the branches corresponds to ML, MP and NJ bootstrap values (>50 %). Chitinase sequences obtained from *Ab*, *MAC* and *MR* genomes are shaded. The tree is drawn to scale with evolutionary distances in the units of the number of amino acid substitutions per site

This dataset contained 85 chitinase sequences, including 20 sequences retrieved from *Ab*, *MAC* and *MR* genomes (Fig. 5a). *Ab* chitinases were only observed in clades A-V and A-II. However, *MAC* and *MR* chitinases were observed in all sg-A clades: A-V, A-IV, A-III and A-II. The majority of chitinase sequences from the three genomes grouped with orthologs from *Trichoderma reesei* (*Hypocrea jecorina*) [Ascomycota, Hypocreales, Hypocreaceae] and other plant pathogenic fungi.

#### Subgroup-B (sg-B) dataset

This dataset included 68 chitinase sequences, including 29 sequences retrieved from *Ab*, *MAC* and *MR* genomes (Fig. 5b). All chitinases were clustered with *H. jecorina* chitinases. Interestingly, *MAC* was found to contain more chitinases than *MR*. However, *Ab* contained the least number of sg-B chitinases. Further, *Ab Chit-1*, *MAC Chit-3*, *MAC Chit-7* and *MR Chit-7* were observed to cluster with *H. jecorina Chi-15*, forming a separate clade in sg-B.

#### Subgroup-C (Sg-C) dataset

This dataset included 50 chitinase sequences, including 18 sequences retrieved from *Ab*, *MAC* and *MR* genomes (Fig. 5c). The number of sg-C chitinases was higher in the *Ab* genome as compared to the *MR* and *MAC* genomes.

### Domain architecture of chitinases

**Fig. 6:**
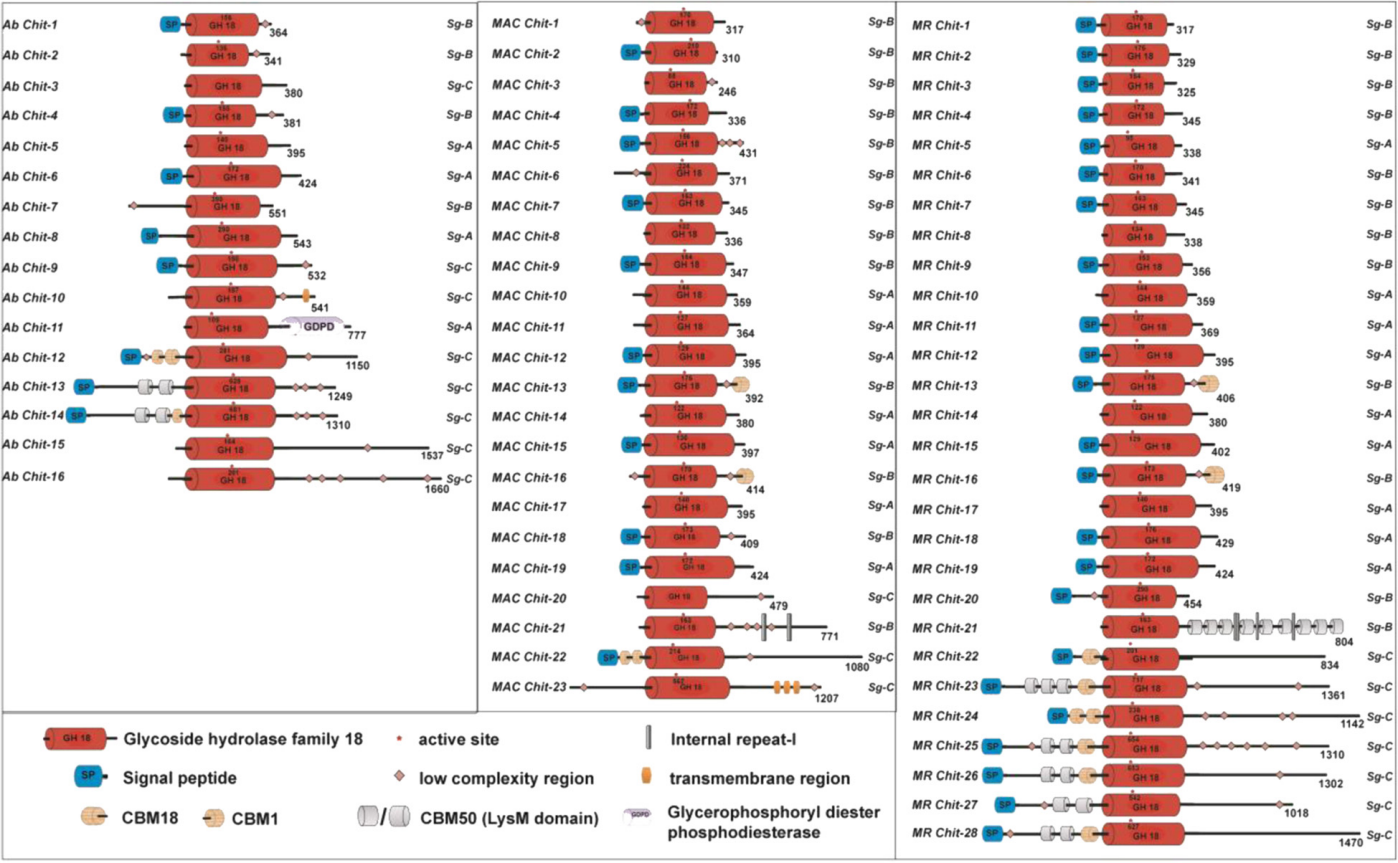
Domain organization of *Ab*, *MAC* and *MR* chitinases. Protein domains, as identified with Pfam and SMART databases are presented. *AbChit-1*, *AbChit-3* and *MACChit-20* lack the active site residue ‘E’ in their catalytic motif (DXXDXDXE). All other chitinase sequences contain intact conserved catalytic motifs. The numbers by the end of each chitinase schematic denote the protein length (amino acids)

The domain architecture for all the chitinases identified by sequence similarity from the three genomes is presented in Fig. 6. It reveals interesting details about domain distribution in chitinases present in the three genomes. Intriguingly, an additional domain glycerophosphoryldiester phosphodiesterase (GPDP) in sg-A classified chitinase *Ab Chit-11* was observed in *Ab* genome. This enzyme GPDP is known for glycerophospholipid metabolism and is indicated to be associated with extracellular events like cell wall organization [78]. Furthermore, the distribution of CBM50 (or LysM domain) known for glycan (chitin) binding functions and predicted as virulence factors [79] was quite variable in the three genomes. Only *Ab* and *MR* genomes displayed the presence of these domains whereas no CBM50 domain was identified in the *MAC* genome. However, higher numbers of CBM50 domains in comparison to *Ab* were observed in *MR* genome. Further, about 82 % (23/28) of chitinases have a signal peptide (SP) signature sequence in *MR* while only 50 % in the other two species contain the SP (Fig. 6).

The sg-B chitinases are known to possess CBMs (CBM1) as their C-terminal domains [76]. Our analysis shows CBM1 is present in the *MAC* and *MR* genomes but absent in the *Ab* genome. Multiple (nine) copies of C-terminal CBM50 domains were observed in *MR* genome.

The sg-C chitinases too displayed some interesting differences. Most of sg-C chitinases contained the N-terminal CBM18 and CBM50 domains in *Ab* and *MAC* genomes. However, *MR* genomes showed regular characteristics of sg-C chitinases, *i.e.*, high molecular weight and presence of multiple domains.

## Discussion

It is often suggested that diversity in pathogen-host relationships contributes to species divergence but the knowledge of genetic mechanisms responsible for speciation and varied host interactions in fungal entomopathogens have been quite limited [80, 81]. The entomopathogenic fungal lineages possess a number of potential pathogenicity associated genes in their genome to acclimatize to varied niches [60, 82–85]. The evolutionary dynamics of host affiliation observed in Clavicipitaceae have been aptly explained by the host-habitat hypothesis where the proximity in the habitat has been related to the acquisition of new hosts [86]. *Metacordyceps* subfamily in Clavicipitaceae lineage is of interest as it contains pathogenic species varying from specialists (with a narrow host range), transitional (with intermediate host range) to generalists (with broad host range) categories [59, 60]. Comparative analyses of their genomes has already been reported that suggests the association of generalists with loss of RIPs (genome defense) and in turn expansion of gene/protein families, whereas the retention of RIPs in specialists fungi [13, 60]. In the current study, comparative genomics of *Ab* belonging to the *Hypocrella* subfamily with *Metacordyceps* members *MAC* and *MR* further aids in understanding the genomic basis for host niche adaptations in the Clavicipitaceae lineage. *Ab*, being a specialist fungus shows an evolutionary pattern similar to the specialist genome *MAC*, with a similar occurrence of RIPs and in the evolution of protein families (contraction). This study also supports that during their evolution generalist fungi have lost the active RIP mechanism which may have aided broader host affiliation by the retention of duplicated genes and in turn protein family expansions. Generalist’ pathogens are suggested to have evolve higher numbers of multi-modular NRPS genes (such as *dtxS1*), siderophores, CBMs and chitinases as contributory factors in acquiring new virulence mechanisms to enhance their pathogenicity potential [13, 60].

### Multimodular NRPS, *dtxS1* as a virulence factor

The hexamodular NRPS gene responsible for the production of destruxins, identified as *dtxS1* gene in *MR* genome was reported to be involved in the pathogenicity of generalist fungus *MR* [59]. It was postulated that acquisition of *dtxS1* gene in *Metarhizium* lineages was in coordination with the evolution of host specificity [59]. Our genome analysis suggests that *Ab* genome does not contain the NRPS *dtxS1* multi-domain gene as reported for specialist pathogens [58–60]. The NRPS subfamilies are known to cluster into two main groups: mono/bimodular NRPS (of ancient origin, bacterial and fungal NRPS) and multimodular NRPS (recent origin, exclusively fungal NRPS) [38]. The multimodular NRPS subfamilies were identified to contain variable domain architecture in response to the niche adaptation roles, illustrating frequent incidences of domain gain and loss events, unlike mono/bimodular NRPS that are less variable in architecture [38, 55]. The destruxin biosynthesis NRPS gene belongs to the multimodular NRPS cluster, is likely to have originated via a gene/domain duplication event in *MR* to form NRPS *dtxS1* gene. Gain of multimodular NRPS gene (such as *dtxS1*) could therefore be suggested as responsible for the generalist behavior and enhanced virulence of *MR* [59, 60].

### Siderophores as virulence factors

Iron is an essential micronutrient for life and the ability of pathogens to cope with limited iron within the host organism represents an evolutionary force for the development of virulence [54, 87]. Siderophores are high-affinity small molecules that function in iron uptake [88]. Pathogenic fungi have evolved the production of these siderophores for acquisition of iron from the host organism they infect, to ensure their own survival [54, 87, 89]. Maintenance of iron homeostasis therefore suggests association of fungal pathogenicity and siderophore biosynthesis [52–54]. There are reports from pathogenic fungi such as *Alternaria alternata* [90], *Aspergillus fumigatus* [53, 54, 91], *Cryptococcus neoformans* [92], *Fusarium graminearum* [93], *Magnaporthe oryzae* [94], *Metarhizium robertsii* [95] that correlate iron acquisition and virulence. For example, mutagenesis studies on murine model of invasive aspergillosis caused by *Aspergillus fumigatus* displayed complete lack of virulence on elimination of the entire siderophore biosynthetic gene (*ΔsidA* mutant) demonstrating critical role of siderophores in pathogenicity and thereby in fungal-host interactions [91, 96, 97]. Likewise, another mutagenesis study of two NRPS enzymes sidD (intracellular siderophore) and sidC (extracellular siderophore) investigated the involvement of siderophores in pathogenesis in *MR* [95]. Based on these studies, it could therefore be proposed that fungal entomopathogens have evolved the genes involved in siderophore biosynthesis to raise their virulence potential.

### Chitinases as virulence factors

Chitinases have been suggested as the determining factor of virulence in fungal entomopathogens with their involvement in degradation of arthropod cuticle [50, 98]. In the present study, we observe a higher proportion of chitinases in the genomes of the insect pathogens (*Ab*, *MAC* and *MR*) compared to other non-insect pathogens like *Aspergillus* spp. and *Rhizopus* spp. [13, 99]. Sg-B and sg-C chitinases are commonly known to be involved in virulent and aggressive functions towards insects in entomopathogenic fungi [100–102] likely due to the presence of additional domains like CBM1, CBM18 and CBM50 responsible for chitin binding functions [77]. Interestingly, only the *MR* genome is observed to contain chitinases (*MR Chit-9* classified under sg-B clade) with multiple CBM50 domains at the C-terminal. Similar observation could also be viewed in sg-C classified *MR* chitinases with the presence of multiple copies of N-terminal CBM18 and CBM50 domains. The presence of comparatively expanded numbers of accessory domains, CBMs in *MR* genome may relate to evolution of multiple host adaptations [103, 104]. Moreover, the complete lack of LysM effectors (CBM50 domain) in *MAC* genome may also be associated with its pathogenicity potential and in turn host-specific behavior [79, 105]. This could also be supported with overall distribution of CBM1, CBM18 and CBM50 domains in *Ab*, *MAC* and *MR* genomes.

### Mating type

Mating type genes are the governing players of sexual functions where the presence or absence of two compatible mating type idiomorphs, MAT1-1 and MAT1-2 decides homothallism (self-fertility) or heterothallism (self-sterility but cross-fertility) in filamentous ascomycetes [41, 106]. Clavicipitaceous fungi too show diversity in their mode of mating [86, 107, 108]. The present analysis based on sequence similarity searches identified putative mating type idiomorphs, MAT1-1 (MAT1-1-1, MAT1-1-2 and MAT1-1-3) in *Ab* suggesting its potential for sexual fertility, which could also be supported by its known teleomorphic form *Hypocrella siamensis* [74]. Mating type genes, MAT1-2-3 and MAT1-2-1 in place of MAT1-1-1, MAT1-1-2 and MAT1-1-3 were identified based on synteny analysis in *MAC* and the conservation of genes flanking the MAT1-1 idiomorph in *MAC* and *MR* (Fig. 4).

## Conclusions

The present study is an attempt to understand the evolution of entomopathogenicity in Clavicipitaceae *s.s.* members in relation to their adaptations to host-range. In this study, we suggest the involvement of multimodular NRPS genes, siderophores, CBMs and chitinases in generalist clavicipitacean fungal entomopathogen *MR* that could have evolved for diverse host affiliations, as compared to specialist entomopathogens *Ab* and *MAC*.

### Availability of supporting data

Additional files 1, 2, 3, 4, 5, 6, 7, 8, 9, 10, 11, 12, 13, 14, 15 and 16.

## Additional files

Additional file 1: Multiple sequence alignment of NRPS genes. (ZIP 225 kb) Additional file 2: Multiple sequence alignment of subgroup A,B and C fungal chitinases. (DOC 593 kb) Additional file 3: Phylogeny and comparative genomics of clavicipitaceous fungi Ab, *MAC*, and *MR*. (PDF 1319 kb) Additional file 4: List of accession numbers for predicted NRPS gene cluster sequences identified in Ab, MAC and MR genomes. (XLS 10 kb) Additional file 5: Major protein families of Ab, MAC and MR genomes implicated in the pathogen-host interactions. (XLS 20 kb) Additional file 6: Analysis of carbohydrate-binding module (CBM) containing proteins, arranged by CBM family. (XLS 14 kb) Additional file 7: Carbohydrate-degrading enzymes, arranged by GH family. (XLS 67 kb) Additional file 8: Carbohydrate-degrading enzymes, arranged by GT family. (XLS 23 kb) Additional file 9: Carbohydrate-degrading enzymes, arranged by PL family. (XLS 7 kb) Additional file 10: Carbohydrate-degrading enzymes, arranged by CE family. (XLS 15 kb) Additional file 11: Carbohydrate-degrading enzymes, arranged by AA family. (XLS 15 kb) Additional file 12: Summary of RIP signatures in *Ab, MAC* and MR genomes. (XLS 46 kb) Additional file 13: RIP frequency index in *Ab*, *MAC* and *MR* genomes. (XLS 8 kb) Additional file 14: Putative transposase genes encoded by *Ab*, *MAC* and *MR. (XLS 10 kb)*
Additional file 15: Sexuality related genes in *Ab*, *MAC* and *MR* genomes. (XLS 22 kb) Additional file 16: Properties of chitinases retrieved from the genomes of *Ab*, *MAC* and *MR*. The active site position, number of amino acid, molecular weight (MW), presence of signal peptide and domains (CBM, LysM and others in addition to the catalytic domain) have been analyzed for the respective genes in the genome and listed. (XLS 21 kb)
