# Supplementary material for: Genome sequence and comparative analysis of clavicipitaceous insect-pathogenic fungus Aschersonia badia with Metarhizium spp
Source: BMC Genomics. 2016 May 17;17:367. doi: 10.1186/s12864-016-2710-6 (PMC4869207; doi:10.1186/s12864-016-2710-6)
Supplement: Additional file 2: — Multiple sequence alignment of subgroup A,B and C fungal chitinases. (DOC 593 kb) [file 12864_2016_2710_MOESM2_ESM.doc]

**Subgroup-A dataset:**

**>AbChit5**

**----------------------------------------------------------------------------------------------------------------------------------------------------------------------------------------------------------------------------------------------------------------------------------------------------------------------------------------------------------------------------------------------------------------------------------------------------------------------------------------------------------------------------------------------------------------------------------------------------------------------------------------------------------------------------------------MTTSGYRTVAYYVN--WAIY--A-RKHRPQDLP--VENLTHVLYAFANVRPD-SGE-VYLTDT-WADTDIHWEG--------D--------SWNDSGN-NIY--GCLKQLNLLKRRN-RNLKVLLSIGGWT-YSSN----FKDPASTPQGRQAFARTSVELLKNLGFDGIDIDWEYPQ---------NQ--------------QEAQNYVYLLAAVRQ----ELDNYAARS--A-NSHHFELTVACPG----GSYNYEKLD--VRGMDRYLDFWNLMAYDYAG-S-WDQVSAHQANLYPK--GDSST-----------PFSTSAAVGFYTGR-GVAAEKIVIGMPLYGRAFENT----------------------------------NGIGQSFQGV----------G-EGTWE----------------------------N-------GVFDYKKL---------------------------------------------------------------------------------PLEGSEE-LYDDT----AQATYCF--NACQRKLVSYDTPLMAKKKAD-YIKAKCLGGAMWWESSGDKS----G------PDGLISTVVDTL---------GGSQSFQKLENCIEY-----PETKYDNLRA---------GFPNN------------------------------------------------------------------------------------------------------------------------------------------------------------------------------------------------------------------------------------------------------------------------------------------------------------------------------------------------------------------------------------------------------------------------------------------------------------------------------------------------------------------------------------------------------------------**

**>AbChit6**

**----------------------------------------MPSLLAISTA----------------------AF----------------------------------------------------------------------------------------------------------------------------------------------------------------------------------------------------------------------------ATLQAVLQLA-------------------------------------------------------------------------------------------------------------------------------------------------------------------------------------------------------------------------------------------------------------------------------------------------------------------TPAFAFENAT------------------------------------------DKGAMNYVNSVYYTN--WAMY--G-RNYQPANLS--ASQVSHVLYAFMNLRAD--GT-VYSGDT-WADTDKHFAT--------D--------SWNDSGK-NVY--GCVKQMYLLKKAN-RQLKVMLSIGGWT-WSTN----FPAAASTAATRSTFAKTAVEFVKDWGFDGVDIDWEYPA---------DE--------------TEATNMVLLLQAVRD----ELDALQAES--A-PNYHFQLSIAAPA----GPEKYKKLH--LAELGKVLDFVNLMAYDYSG-S-WSQYSGHDANLYNSNKNPDAT-----------FFNTDDAVEAYIKG-GVPASKLVLGMPIYGRSFENT----------------------------------DGIGKPFSGI----------G-EGSWE----------------------------K-------GVWDYKAL---------------------------------------------------------------------------------PKAGAVL-QYDSD----AKGYYSY--DPSTRELISFDTPGMIREKVT-YLKGKGMGGSMFWEASADKE----G------PDSLIGTSASEL---------G---SLDVTQNLLDY-----PNSQYENMKN---------HMA--------------------------------------------------------------------------------------------------------------------------------------------------------------------------------------------------------------------------------------------------------------------------------------------------------------------------------------------------------------------------------------------------------------------------------------------------------------------------------------------------------------------------------------------------------------------**

**>AbChit8**

**----------------------------------------MYSSVLSILP----------------------LLAQI--------------------------------------------------------------------------------------------------------------------------------------------VTAAPAQRQD------------------AHRRDLMCIEVKASTI---YLPTTIYINGASQPTPIPTITQ-------VKGSEAQLAAAASPTATN----K--------PVQPNKIIVTEGLR---------------------------------------------------------------------------------------------------------WGNKTRFGNSTANCTDHRGTGKMLDVGIT----------------------------------------ANLPTKVKAGKA--------------------------------------------------------------------------------------AKPTAPIAYID------------------------------------------TSKSVAPRNVLYFTN--WGIY--G-ANYQPQDVP--ADKVTHLLYAFGDMKDD--GT-VVSSDP-YADVQKRYDG--------D--------ALFKRAS-DAY--GVVNQLYGMKKKN-RNLKTLLSIGGYT-YSQQ--GKFLKFAGSDQGRKTFASSAVKLLADWGLDGIDIDWEYPA---------NA--------------AEAKYFVELLKETRA----ALDDYAAAN--G-QKYHYLLTVAASA----GPDHYRLLD--LKAMDQYVDAWHLMAYDYAG-T-WDTTTGHQANVNADPSNMAST-----------KFNTDQVVGDYTRA-GIAPSKLVLGLPLYGRSFGDT----------------------------------DGLGKPFQGA----------G-DGAQQ------------------------------------GSILYRDL---------------------------------------------------------------------------------PRSGATV-QVDGR----VGAAWSY--DAKARQLVSFDNVASAKLKAN-YVKNKSLGGAVFWEASGDKK----G------DESLVDTMARSL---------G---HLDETNNMLSY-----PQSQYDNIKN---------GASET------------------------------------------------------------------------------------------------------------------------------------------------------------------------------------------------------------------------------------------------------------------------------------------------------------------------------------------------------------------------------------------------------------------------------------------------------------------------------------------------------------------------------------------------------------------**

**>AbChit11**

**-------------------------------------------------------------------------------------------------------------------------------------------------------------------------------------------------------------------------------------------------------------------------------------------------------------------------------------------------------------------------------------------------------------------------------------------------------------------------------------------------------------------------------------------------------------------------------------------------------------------------------------------------------------------------------------------------MYLTG--QHNVVP-----SSDSRL--IDGITHVILAFMRSD-------VFNSED---------AS-------------------------PEF--PLFTTVKETRRHFTAETKVMIATGGWG-DSLG----FEEASRNATSREKWCSNVKRMIDMTGADGVDIDWEYPGAVLTSNVPSGN-RDDYKIIPNEKRRWEIEAYVELLSDLRK----AI------------GRHKLLSIAVPGLER-DMMAFSAET--VPRLVQVVDFINVMTYDLLN-R-RDVQVKHHSGV----------------------VASLDSIQTYMAR-GAPSQMLNLGLGYYVKWALTEK---CDA----------GQILE---------CRTQLLEDPNSGAD-----------LGR------------------------------T-------AGFSWHDQVPP---------------------------------------------------------DLEP---------------SFARALADG-HYFED-----GSFGYW--DAAELRWWTFDTEKVILSKLRTIVGPLRLGGVFAWGLGEDAP-----------AFRHLSATFDGL---------KELRNTE---------------GKRDELLAVISQVTTASPRPSTHVELGPRPYYLVNNMTDGPLRSQLESCKEKEMRPSKFSIGHRGGACLQFPEHS--QESTMA-GARMGVGVLECDVTFTKDLQLVCRHSQCDLHTTTNIVSIAALNAKCTKPFIPAANGKPASAKCCTSDITLAEYKSLCAKMDGFNASATNAHDFLSGTPSWRTDLYATCGKTVHLTEHIAMVEKLGLHHTPELKVPEVTMPFTGPNNSTYT------------YERFAQQMIDAYKTARVPPSRVIAQTFDHSIMRFWLLREPEFAKTAILLDQTADEG-FGTMAQAIDN-----LAVYAQEGVQTMAPPLHY-------LVETRNKTIVPSAYAKR---ASQLGLKLITWSLERSGPLAAVHS-----RGDY----------YY-------------------GTIQDAVTKDGDLYTYVDVLARQVGV---VGMFSDWSATVTF------------------YAN---CF--------------------GIGLM------------------------------------------------**

**>MAC_Chit10**

**MSS-------------------------------------------------------------------------------------------------------------------------------------------------------------------------------------------------------------------------------------------------------------------------------------------------------------------------------------------------------------------------------------------------------------------------------------------------------------------------------------------------------------------------------------------------------------------------------------------------------------------------------HSPPRKSRLAT------------------------------------------SVARVMYTNAVYFSN--NRVY----QGDTPAMLN--YSCVNRVYYAYASVGPD--GG-VFLSDE-WADARAPIDG--------------------------VQ--GALGSLMHLKQRH-PHLQVVLSIGGGA-CSNL----FPVVAGNAHLRENLARSALGLVEASGLDGIDIAWEYPC---------SV--------------EQGNDFLALLATIRL----HL-----------PDDRYILTAALPA----TKSVLQFID--LRMAADYLDAVNLMAYDFFG-M-WTPKSGHHSQLYAMSRD---------------EPSGSSGVAHLMSH-GFPSGGILLGIPTYGRSFQHA----------------------------------TGPGQKFKG--------------GGGN----------------------------D-------GTFEYNQL---------------------------------------------------------------------------------PRKGCKE-SVDKR----HISAQCV---GGDGGFVTYDNPDTVKAKAS-FAKQKGLGGLFYWNGTADSK---ET------SRSLVAAGFRAL---------HTS-----------------------------------------------------------------------------------------------------------------------------------------------------------------------------------------------------------------------------------------------------------------------------------------------------------------------------------------------------------------------------------------------------------------------------------------------------------------------------------------------------------------------------------------------------------------------------------------------------------**

**>MAC_Chit11**

**MRS---------------------------------------------------------------------------------------------------------------------------------------------------------------------------------------------------------------------------------------------------------------------------------------------------CTFPLAAAA------------------------------------------------------------------------------------------------------------------------------------------------------------------------------------------------------------------------------------------------------------------------------------------------------------------------------------------------------------------------TAVLAAPRYAMYFDQ--WHKATL------PPKDV--TAGVHYVITAFAPSE-------TFN------------SG-------------------------SSY--QPFMPLDQVRALFDEGTKVCMAIGGWG-DTSG----FSIGAATEEKRKTYAKNVATALNKLGYDCVDIDWEYPG---------GN-GQDYKQTPNDKKVSEIDTYPLLLQEIKT----AI------------G-DKELSIAIPGREG-DMIAFTAEQ--VPKIDRI-----VMAYDIMN-R-RDNATNHHTSV----------------------VDCAHTIDIYIKR-GMTASKMNLGFAFYAKYFTTKDGFKC------------TEPTG---------CATAVLEAA-DGSD-----------PGL------------------------------S-------GAVTFEIENYN-----------------------------------------------------------NA---------------AFVKALQNG-KEDAD----KGGMWYW--DSSTKQYWTWDAPDLVARKFKEIVAAKKLGGVFAWSLAQDSH-----------DWSHFKAMQAGV---------KSL-----------------------------------------------------------------------------------------------------------------------------------------------------------------------------------------------------------------------------------------------------------------------------------------------------------------------------------------------------------------------------------------------------------------------------------------------------------------------------------------------------------------------------------------------------------------------------------------------------------**

**>MAC_Chit12**

**MRFEF------------------------------------------------------------------TLWT---------------------------------------------------------------------------------------------------------------------------------------------------------------------------------------------------------------------------ALLSYVA---------------------------------------------------------------------------------------------------------------------------------------------------------------------------------------------------------------------------------------------------------------------------------------------------------------------------------------------------------------------------PALGGNLLVGYYPT--WKHG-------NISTMD--LSNYTHINIAFAIPNEQ--GA-LKFEGD-----------------------------------------AFMDKVVPKLQGG--GSKVLVSVGGWS-GSAN----FSTIVKDTALSGTLTKNIIDMMKKYKLDGIDIDWEFPG---------RK-GSDCNTVDEQ---NDATNFLKYLTDLRA----KMDEA--------LGKGKLITLALRVQPFDGPGG---KD--VSGFAKVVDFANLMQYDLNG-A-WGETSGPLAPLNFEKGKGTQ-------------QSFATAIEEWTKA-GWPAKQLTGGAAFYGYSATATEDMLK------------SNP--------------PSQYAKMAKE---------RP-KGDQDD--E--MVTEP-C--------SSAAATYT-------GIWVYKNLRKQG--------------------------------------------------------VLSS--P------------DTAEAPWVR-TFDNK----TMTPWLF--NPQTKTFISYDDPQSLAAKVK-FAESKGLGGMMVWSIERDY------------NGELLSELKKFS---------SK------------------------------------------------------------------------------------------------------------------------------------------------------------------------------------------------------------------------------------------------------------------------------------------------------------------------------------------------------------------------------------------------------------------------------------------------------------------------------------------------------------------------------------------------------------------------------------------------------------**

**>MAC_Chit14**

**MAAS-----------------------------------------------------------------------------------------------------------------------------------------------------------------------------------------------------------------------------------------------------------------------------------------------------------------------------------------------------------------------------------------------------------------------------------------------------------------------------------------------------------------------------------------------------------------------------------------------------------------------------------------------------------------------------------THEPFAPRCVMYLTG--QHNVVP------SDQEL--TKHITHVVLAFMRSD-------VFNSDQ---------QS-------------------------PEF--PLFTTVGEVREKFHHETKVMVAVGGWG-DSKG----FEEANKDAKSRERWCENVKEMIDLTGADGVDIDWEYPG---------GN-RDDYKIIPNEKRKWEIEAFVQLLTDLRV----AI------------GREKLLSIAVPGLER-DMMAFTEET--VPRIVKQVDFINVMTYDLLN-R-RDTLVKHHSGV----------------------SASLESIESYMAR-GASAQLLNLGLAYYVKWAMTEE---CDP----------GHPLS---------CATQLLEDPETGAD-----------LGR------------------------------T-------AAFSWHDKIPE---------------------------------------------------------ELEK---------------SFSRAVTDG-KYFED-----GSFGYL--DVEETRWWTFDTPSVIHRKFEDIVKERRLGGVFAWGLGEDAP-----------VFSHLSATLHEL---------QNLTRDGMGE-----------AASKDEL----------------------------------------------------------------------------------------------------------------------------------------------------------------------------------------------------------------------------------------------------------------------------------------------------------------------------------------------------------------------------------------------------------------------------------------------------------------------------------------------------------------------------------------------------------------------------------**

**>MAC_Chit15**

**MQFGR------------------------------------------------------------------VLLT---------------------------------------------------------------------------------------------------------------------------------------------------------------------------------------------------------------------------AVLCHLA--------------------------------------------------------------------------------------------------------------------------------------------------------------------------------------------------------------------------------------------------------------------------------------------------------------------------------------------------------------------------PALAAPPIIVGYYPT--WKHE-------VLSKLD--LSNYTHVNVAFAIPDED--AN-LEFEGD-----------------------------------------KLMPEIVPKLQGH--NTKVLVSVGGWT-GSAF----LSNITKKPELSAALTMNIIELMKKHNLDGIDIDWEYPG---------QA-GSPCNFFDKE---NDTRNFLAYLKSLRT----KV------------GRDKLITLAVRTTPFAGPGG---ED--VSEFAKVVNFANLMQYDMNG-A-WGQTTGPNAPLNFERGKATQ-------------QSFATAIEAWTKA-GWPAGQLTSGLAFYGRSLTATVDMLA------------ANP--------------MSQYQGFSKQ---------VP-RGDRLD--A--PTAEV-CPPNSATTVNGT-RAFS-------GTWTYANLREQG--------------------------------------------------------VLST--P------------TTASAPWVR-TFDNI----TMTPWLF--NPEDKTFITYDDPASLRAKVK-FAKSKGLAGAMAWEMTGDF------------NGELVSAVRAAA-----------------------------------------------------------------------------------------------------------------------------------------------------------------------------------------------------------------------------------------------------------------------------------------------------------------------------------------------------------------------------------------------------------------------------------------------------------------------------------------------------------------------------------------------------------------------------------------------------------------------**

**>MAC_Chit17**

**----------------------------------------------------------------------------------------------------------------------------------------------------------------------------------------------------------------------------------------------------------------------------------------------------------------------------------------------------------------------------------------------------------------------------------------------------------------------------------------------------------------------------------------------------------------------------------------------------------------------------------------------------------------------------------------MGNDGYRTVVYFVN--WAIY--A-RKHRPQDLP--VENLTHVLYAFANVRTD-TGE-VHLTDT-WADTDIHWEG--------D--------SWNDTGN-NIY--GCLKQLNLLKKRN-RNLKVLLSIGGWT-YSAN----FKNPASTRQGRETFAKSSVELLKNLGFDGIDIDWEYPQ---------NP--------------EEARNYVELLAAVRQ----ELDAYAAKC--V-DNYHFELTVACPA----GPNNYEKME--IAEMDKYLDFWNLMAYDYAG-S-WDSVSGHQANIYPG--NDAST-----------PFSTNAAIDYYTSH-GVAANKIVLGMPLYGRAFENT----------------------------------DGPGKPFQGV----------G-EGTWE----------------------------N-------GVFDYKKL---------------------------------------------------------------------------------PLEGSEE-FSDDQ----AQASYCY--SAANRKLVSYDTAAMAKTKAE-YVKQRALGGAMWWESSGDKS----G------SDSLISTVVETF---------GGPGCLQKLDNCITY-----PETKYDNLRD---------GFPNN------------------------------------------------------------------------------------------------------------------------------------------------------------------------------------------------------------------------------------------------------------------------------------------------------------------------------------------------------------------------------------------------------------------------------------------------------------------------------------------------------------------------------------------------------------------**

**>MAC_Chit19**

**----------------------------------------MPSLFAQSLA----------------------II----------------------------------------------------------------------------------------------------------------------------------------------------------------------------------------------------------------------------ATLQATLGLA-------------------------------------------------------------------------------------------------------------------------------------------------------------------------------------------------------------------------------------------------------------------------------------------------------------------TPVSAPDTVI------------------------------------------GKRAGGYVNAVYFTN--WGIY--G-RNYQPADLP--ASQISHVLYSFLNLSAN--GT-VYSGDT-WADIDKHYPN--------D--------SWNDVGN-NVY--GCAKQLFLLKKAN-RKMKTMLSIGGWT-WSTN----FPAAASTAATRSNFAKSAVTIMKDWGFDGIDVDWEYPA---------DD--------------TQATNMVLLLQAVRD----ELDAYAAKF--A-PGYHFQLSIAAPA----GATNYNKLH--LADLGKVLDYINLMAYDFSG-S-WSNSSAHNANLYANPSNLNAT-----------PFNTDDAVNDYIKG-GVPASKIVLGMPIYGKSFQKT----------------------------------NGIGKPFSGI----------G-DGSWE----------------------------N-------GVWDYKVL---------------------------------------------------------------------------------PKAGARV-IYDDV----AKGYYSY--DNRTQELISYDTPDITKEKVT-YLKSKGLGGSMFWEASADRK----G------PDSLIGTSSNKL---------G---GPDATENLLNY-----PDSKYDNMRK---------QMA--------------------------------------------------------------------------------------------------------------------------------------------------------------------------------------------------------------------------------------------------------------------------------------------------------------------------------------------------------------------------------------------------------------------------------------------------------------------------------------------------------------------------------------------------------------------**

**>MR_Chit5**

**MRC---------------------------------------------------------------------------------------------------------------------------------------------------------------------------------------------------------------------------------------------------------------------------------------------------SFLVVLATT------------------------------------------------------------------------------------------------------------------------------------------------------------------------------------------------------------------------------------------------------------------------------------------------------------------------------------------------------------------------AATFAAPRHVVYFDH--------------------------------------------------------------------------------------SY--QPFMPLDQIRALFDRGTKVCMAIGGWG-DTSG----FSVGAVNEESRKRYAKNVAEAVDRLGYDCVDVDWEYPG---------GN-GQDYGQIPNEQKVSEIETYPLLLAEIKA----AI------------G-RRELSIAVPGREA-DMIAFTAEQ--VPKIDKTVDFINVMTYDLMN-R-RDNATNHHTSV----------------------EGSLQTVDIYIQR-GMTPSKMNLGFAFFAKYFTTKHGAQC------------LEPTG---------CPTAVLESA-DGSD-----------THL------------------------------S-------GAVTFEIDNYNS----------------------------------------------------------NA---------------AFANALKNG-KQDSA----RGGQWFW--DASTEIYWTWDTPDLIARKFKEIVSARKLGGVMAWSLAQDSH-----------DWSHSKAVQAGV---------KEL-----------------------------------------------------------------------------------------------------------------------------------------------------------------------------------------------------------------------------------------------------------------------------------------------------------------------------------------------------------------------------------------------------------------------------------------------------------------------------------------------------------------------------------------------------------------------------------------------------------**

**>MR_Chit10**

**MSS-------------------------------------------------------------------------------------------------------------------------------------------------------------------------------------------------------------------------------------------------------------------------------------------------------------------------------------------------------------------------------------------------------------------------------------------------------------------------------------------------------------------------------------------------------------------------------------------------------------------------------HSRPRKSRIAT------------------------------------------SVARVMYTNAVYFSN--NRVY----QGDTPAMLN--YSCVNRVYYAYASVGSD--GG-VFLSDE-WADARAPIDG--------------------------VQ--GALGSLMHLKQRH-PHLQVVLSIGGGA-CSNL----FPMVAGNAHLRENLARSALGLVEASGLDGIDIAWEYPC---------SV--------------EQGNDFLALLATIRL----HL-----------PEDRYILTAALPA----TKTVLQFID--FSMAADYLDAVNLMAYDFFG-M-WTPKSGHHSQLYAMSRD---------------EPSGSSGVAHLMSH-GFPSGGILLGIPTYGRSFQHA----------------------------------TGPGQKFKG--------------GGGN----------------------------D-------GTFEYNQL---------------------------------------------------------------------------------PRKGCKE-SVDKR----HISAQCV---GGDGGFVTYDNPDTVKAKAA-FAKQKGLGGLFYWNGTADSK---EA------SRSLVAAGFRAL---------HTS-----------------------------------------------------------------------------------------------------------------------------------------------------------------------------------------------------------------------------------------------------------------------------------------------------------------------------------------------------------------------------------------------------------------------------------------------------------------------------------------------------------------------------------------------------------------------------------------------------------**

**>MR_Chit11**

**MRS---------------------------------------------------------------------------------------------------------------------------------------------------------------------------------------------------------------------------------------------------------------------------------------------------CMFLLLAAA------------------------------------------------------------------------------------------------------------------------------------------------------------------------------------------------------------------------------------------------------------------------------------------------------------------------------------------------------------------------TAVSAAPRYVMYFDQ--WHKTTL------PPKDV--TAGVNYVITAFAPST-------TFN------------SG-------------------------SSY--QPFMPLDQVRALFDKGTKVCMAIGGWG-DTSG----FSTGAATETTRKTYAKNVAAAVKTLGYDCVDVDWEYPG---------GN-GQDYKQTPNDKKVSEIETYALLLQEIKT----AI------------G-EKELSIAVPGKEG-DMIAFTAEQ--VPKIDKAVDFVNVMTYDIMN-R-RDNATNHHTSV----------------------VDCAHTIDTYIKR-GMTASKMNLGFAFYAKYFTTKDGVEC------------AEPTG---------CPTAVLEAP-DGSD-----------LNL------------------------------S-------GAFTFEIENYS-----------------------------------------------------------KA---------------AFTKALQNG-KEDKA----KGGMWYW--DSSTKQYWTWDAPDLIARKFKEIVAAKKLGGVMAWSLAQDSH-----------DWSHFKAMQAGV---------KSL-----------------------------------------------------------------------------------------------------------------------------------------------------------------------------------------------------------------------------------------------------------------------------------------------------------------------------------------------------------------------------------------------------------------------------------------------------------------------------------------------------------------------------------------------------------------------------------------------------------**

**>MR_Chit12**

**MRFES------------------------------------------------------------------TLWT---------------------------------------------------------------------------------------------------------------------------------------------------------------------------------------------------------------------------ALLSYVA---------------------------------------------------------------------------------------------------------------------------------------------------------------------------------------------------------------------------------------------------------------------------------------------------------------------------------------------------------------------------PALGANVLVGYYPT--WKHA-------AMSNMD--LSNYTHVNVAFAIPHEN--GS-LAFEGD-----------------------------------------TFMDKLVPKLQGA--GSKVLVSIGGWS-GSAN----FSSITKDTALSGALTKSIIDMMTKYKLDGIDIDWEFPG---------RK-GSDCNVVDEQ---NDATNFLKYLNDLRA----KMDEA--------FGKGKLITLALRIQPFDGPGG---KD--VSGFAKVVDFANLMQYDLNG-A-WGETSGPLAPLNFEEKKGTQ-------------LSFATAIEAWTKA-GWPAKQLTAGAAFYGYSVTATEDMLK------------TNP--------------PNQYAKMAKE---------RP-KGDQED--E--MVTEP-C--------SSAPKAYT-------GIWTYKNLRGQG--------------------------------------------------------VLSS--P------------DEAKAPWVR-TFDNK----TMTPWLF--NSESKVFISYDDQRSLAAKVK-FAESKGLGGMMVWSIERDY------------NGELLSELKKFG---------SK------------------------------------------------------------------------------------------------------------------------------------------------------------------------------------------------------------------------------------------------------------------------------------------------------------------------------------------------------------------------------------------------------------------------------------------------------------------------------------------------------------------------------------------------------------------------------------------------------------**

**>MR_Chit14**

**MATS-----------------------------------------------------------------------------------------------------------------------------------------------------------------------------------------------------------------------------------------------------------------------------------------------------------------------------------------------------------------------------------------------------------------------------------------------------------------------------------------------------------------------------------------------------------------------------------------------------------------------------------------------------------------------------------THQPFATRCVMYLTG--QHNVVP------SELEL--TKHITHVVLAFMRSD-------VFNSDQ---------QS-------------------------PEF--PLFTTVGEVREKFHHETKVMVAVGGWG-DSKG----FEEANKDAKSRKRWCKNVKKMIDLTGADGVDVDWEYPG---------GN-RDDYKIIPNERRKWEIEAFVQLLTDLRV----AI------------GSDKLLSIAVPGLER-DMMAFTEET--VPRIVKQVDFINVMTYDLLN-R-RDTLVKHHSGV----------------------SASLESIESYIAR-GASAPVLNLGLAYYVKWAMTEE---CDP----------GHPLS---------CPTQLLEDPETGAD-----------LGR------------------------------T-------AAFSWHDKIPE---------------------------------------------------------ELEQ---------------SFSRAMTNG-KYFED-----GSFGYL--DVKEARWWTFDTPNVIHRKFKDIVKQRRLGGVFAWGIGEDAP-----------AFSHLSATLHEL---------QNLTKDDMGE-----------AASKDEL----------------------------------------------------------------------------------------------------------------------------------------------------------------------------------------------------------------------------------------------------------------------------------------------------------------------------------------------------------------------------------------------------------------------------------------------------------------------------------------------------------------------------------------------------------------------------------**

**>MR_Chit15**

**MQFNP------------------------------------------------------------------ILLA---------------------------------------------------------------------------------------------------------------------------------------------------------------------------------------------------------------------------AVLSHLA--------------------------------------------------------------------------------------------------------------------------------------------------------------------------------------------------------------------------------------------------------------------------------------------------------------------------------------------------------------------------PALAAPPIIVGYYPT--WRHT-------VLKDLD--LSGYTHINIAFALPDEK--AN-LALEGE-----------------------------------------ENMPEIVPKLQEK--GSKVLVSVGGWT-GSAF----FSNITTE-GMRETFANNVIELMKKHNLDGIDFDWEYPG---------QA-GSPCNFFDEQ---NDTNNFLAFLDLMRS----KVNG---------LGGDKLITLAVLNRPFAGPGG---ED--VSKFANFVNFTNLMQYDMNG-A-WGNITGPNAPLNFEPGKATQ-------------RSFATAIEAWTKA-GWPADKLTSGLAFYGRSVTTDVDMLA------------QDP--------------VTQYANFSKQ---------VP-KGDDLD--E--LKPET-CPPNSAVSVNSTEEAWS-------GTWRYASLRGQG--------------------------------------------------------VLTA--P------------TTPSPPWVR-TFDSI----TMTPWLF--NPETKTYLTYDDPESLDAKVK-FALSKGLAGTMAWEIAGDF------------NGELLAAVRGAL---------GV------------------------------------------------------------------------------------------------------------------------------------------------------------------------------------------------------------------------------------------------------------------------------------------------------------------------------------------------------------------------------------------------------------------------------------------------------------------------------------------------------------------------------------------------------------------------------------------------------------**

**>MR_Chit17**

**----------------------------------------------------------------------------------------------------------------------------------------------------------------------------------------------------------------------------------------------------------------------------------------------------------------------------------------------------------------------------------------------------------------------------------------------------------------------------------------------------------------------------------------------------------------------------------------------------------------------------------------------------------------------------------------MSNDGYRTVAYFVN--WAIY--A-RKHRPQDLP--VENLTHVLYAFANVRTD-TGE-VHLTDT-WADTDIHWEG--------D--------SWNDTGN-NIY--GCLKQLNLLKKRN-RNLKVLLSIGGWT-YSAN----FKNPASTPQGRETFAKSCVELLKNLGFDGIDIDWEYPQ---------NP--------------EEARNYVELLAAVRQ----ELDAYAAKC--V-DNYHFELTVACPA----GPNNYEKFE--IAEMDKYLDFWNLMAYDYAG-S-WDSVSGHQANIYPG--NNAST-----------PFSTNAAVDYYTSH-GVAANKIVLGMPLYGRAFENT----------------------------------DGPGKPFQGV----------G-EGTWE----------------------------N-------GVFDYKKL---------------------------------------------------------------------------------PLEGSEE-FSDDQ----AQASYCY--SAANRKLVSYDTAPMAKTKAE-YVKQRALGGAMWWESSGDKS----G------SDSLISTVVETF---------GGPGCLQKLDNCITY-----PETKYDNLRD---------GFPNN------------------------------------------------------------------------------------------------------------------------------------------------------------------------------------------------------------------------------------------------------------------------------------------------------------------------------------------------------------------------------------------------------------------------------------------------------------------------------------------------------------------------------------------------------------------**

**>MR_Chit18**

**----------------------------------------MPSLFASSLA----------------------LLAA------------------------------------------------------------------------------------------------------------------------------------------------------------------------------------------------------------------------------------------------------------------------------------------------------------------------------------------------------------------------------------------------------------------------------------------------------------------------------------------------------------------------------------------------------SATGAAAACAG------------------------------------------KKNSTGYVNAVYFTN--WGTHN-G-TNFQPAALP--APQISHLNYAFLNLTIN--GT-VFSADP-VADTERRYPG--------DFQLTLPAAQDKSDTA-NVY--GAVKQLFLLKKAN-RHMKLLISIGGWT-WSQN--PAFATVAASNATRLTFARSAVELVRDWGFDGIDLDWEYPQ---------DA--------------KQAADMVLLLQALRD----ELDAYAARS--A-PGYRFQLTIASPA----GAAAYNKLQ--LAKLGAILDYVHLMAYDFAG-S-WSNASGHLAHLY---TNPQLV-----------NRSADSAVRDYIKG-GVPPHKLVLGMPIYGRAFPNT----------------------------------TGLGKPFTSTP--------PT-EGNEE----------------------------P-------DTYMYNKL---------------------------------------------------------------------------------PKAGAVE-MYDDV----AKAVYSY--DNRTRELISYDTPTVTRDKVA-YVKRLGLGGSMFWEASGDRN----G------SQSLIGTSLRAL---------G---SLDSSQNLLDY-----PDSRYANIRN---------KMACS------------------------------------------------------------------------------------------------------------------------------------------------------------------------------------------------------------------------------------------------------------------------------------------------------------------------------------------------------------------------------------------------------------------------------------------------------------------------------------------------------------------------------------------------------------------**

**>MR_Chit19**

**----------------------------------------MPSLFAQSLA----------------------II----------------------------------------------------------------------------------------------------------------------------------------------------------------------------------------------------------------------------ATLQATLGLA-------------------------------------------------------------------------------------------------------------------------------------------------------------------------------------------------------------------------------------------------------------------------------------------------------------------TPVSAPDTVT------------------------------------------GKHAGGYVNAVYFTN--WGIY--G-RNYQPADLP--ASQISHVLYSFLNLSNN--GT-VYSGDS-WADIDKHYPN--------D--------SWNDVGN-NVY--GCVKQLYLLKKAN-RNMKTMLSIGGWT-WSTN----FPAAASTAATRSNFAKSAVTIMKDWGFDGIDVDWEYPA---------DD--------------AQAANMVLLLQAVRD----ELDAYAAKF--A-QGYHFQLSIAAPA----GPANYNKLH--LGDLGKVLDYINLMAYDFSG-S-WSNSSAHNANLYANPGNLNAT-----------PFNTDDAVNDYIKG-GVPASKIVLGMPIYGKSFQKT----------------------------------NGIGKPFSGV----------G-DGSWE----------------------------N-------GIWDYKVL---------------------------------------------------------------------------------PKAGATV-IYDDV----AKGYYSY--DNRTQELISYDTPDITKEKVT-YLKSKGLGGSMFWEASADRQ----G------PDSLIGTSSNKL---------G---GPDTTENLLNY-----PDSKYDNMRK---------QMA--------------------------------------------------------------------------------------------------------------------------------------------------------------------------------------------------------------------------------------------------------------------------------------------------------------------------------------------------------------------------------------------------------------------------------------------------------------------------------------------------------------------------------------------------------------------**

**>EAA76014_G_zeae_A_II**

**MHFLKMSF--------------------------------------------------------------AWLL----------------------------------------------------------------------------------------------------------------------------------------------------------------------------------------------------------------------------ALLAMATA-------------------------------------------------------------------------------------------------------------------------------------------------------------------------------------------------------------------------------------------------------------------------------------------------------------------------------------------------------------------------ADSAEPFRCIMYLTG--QHDVVP-------AKHQ--FKGVSHVVIAFMRSE-------FFNVDE---------QP-------------------------DDY--PMFTSVSDVRARVPQHTKVMVAIGGWG-DTQG----FEEAAKTYFSRKRWTRQVAAMVKATEADGIDVDWEYPG---------GN-RDDYKEIPNSEREWEIEAFVSLLQELRA----AL------------GPEKILSAAVPGKEG-DLMAFTTDT--VPRIMKEVNFLNIMSYDLMN-R-RDNTTVHHSGV----------------------ENSQEAVQRYIDR-GASPSSVNLGLGYYVKWFMTEK---CDP----------AKPVG---------CRTPILEDPETGAD-----------LGK------------------------------T-------GGFSWHDEVPK---------------------------------------------------------DVAQ---------------SFSRARYDG-KYDVD-----GSYYYW--DEQELRWWSFDTTRSIQTKFERIVPQLKLGGVFAWGIGEDAP-----------DFEHFLTTAEEV---------RKIREGD---------------QVKDEL----------------------------------------------------------------------------------------------------------------------------------------------------------------------------------------------------------------------------------------------------------------------------------------------------------------------------------------------------------------------------------------------------------------------------------------------------------------------------------------------------------------------------------------------------------------------------------**

**>EAA69039_G_zeae_A_II**

**MHLS--------------------------------------------------------------------------------------------------------------------------------------------------------------------------------------------------------------------------------------------------------------------------------------------------ALLGLGA--------------------------------------------------------------------------------------------------------------------------------------------------------------------------------------------------------------------------------------------------------------------------------------------------------------------------------------------------------------------------AAVTSASRNIIYYDQ--WHTKDL------PSKDI--TSGVTHVMMSFANSS-------LFTTQ----------PG-------------------------GKY--EPFQPLEKVRSLFDHDIKVCLAVGGWG-DNAG----FDEGVKTDRSRERFARNVASTLDRLGFDCVDIDWEYPG---------GN-GQDYKQVPNSKKTYEIKAFPKLLKEIKK----FI------------G-EKELSIAVPGLER-DMIAYLPTE--APLINKYVDFVNVMTYDLMN-R-RDHYTTHHVSI----------------------AGAARAIDKYISL-GFPPSKLVLGIPFYAKYFMTKKGYTC------------TEPIG---------CPTELLENPEDGSD-----------TGK------------------------------S-------GSMTFEAANFAAA-PTNLTTSTDATCGAGTFFKCPAGSCCAASGWCGSTPAHCGTGCQSAFGK-CDGVDINN---------------SFHKALKDG-RTDTV----NGGQWYW--DSETRIFWTWDTAELIAQKIAFMAQTRGVKSVMAWALALDSN-----------DWSHLKAMQQGF---------KDVNA---------------------------------------------------------------------------------------------------------------------------------------------------------------------------------------------------------------------------------------------------------------------------------------------------------------------------------------------------------------------------------------------------------------------------------------------------------------------------------------------------------------------------------------------------------------------------------------------------------**

**>Chi18-4_H_jecorina_A_II**

**-------------------------------------------------------------------------------------------------------------------------------------------------------------------------------------------------------------------------------------------------------------------------------------------------------------------------------------------------------------------------------------------------------------------------------------------------------------------------------------------------------------------------------------------------------------------------------------------------------------------------------------------------------------------------------------------------MYLTG--QHVVMP------SEDHL--IDPITHVILAFMRSD-------VFNVDE---------TP-------------------------TEF--PLFATVSEVRKQFKPATKIMVAIGGWG-DSQG----FEAAARDATSRRRWAGQVKAMVDLTGADGVDIDWEYPG---------GN-RDDYKLIPNSQRKWEIEAFPLLVAELRS----AL------------GDDKLLSVAVPALER-DLMAFTNST--VPSIVEHVDFINLMTYDMMN-R-RDSYVKHHSGI----------------------DESEAAIRRYMDR-GAPPHKLNLGLGYYAKWFMTEK---CDA----------QQPLG---------CRTQLLEDPTSGAD-----------LGR------------------------------T-------GAFSWHDEVPV---------------------------------------------------------ELNE---------------SFTRAQAHG-FYDYD-----GSYGYW--DAEEKRWWSYDTPDTIGARIAWLVGQLELGGVFAWGLGEDAP-----------LFEHLRATVDGI---------LALREVDEGE-------------SKDEL----------------------------------------------------------------------------------------------------------------------------------------------------------------------------------------------------------------------------------------------------------------------------------------------------------------------------------------------------------------------------------------------------------------------------------------------------------------------------------------------------------------------------------------------------------------------------------**

**>Chi18-11_H_jecorina_A_II**

**------------------------------------------------------------------------------------------------------------------------------------------------------------------------------------------------------------------------------------------------------------------------------------------------------------------------------------------------------------------------------------------------------------------------------------------------------------------------------------------------------------------------------------------------------------------------------------------------------------------------------------------------------------------------------------------------------------------SLPDHSV--TAGVTHVTTAFADSV-------IFN------------SG-------------------------TWY--SPFMAPDQVRSLFDTGTKLCLAVGGWG-QSAG----FDAAAKTDETRQLYAKNVADTIKNLGYDCVDIDWEYPG---------GN-GEDYKVVPNSEKAWEIEAYPLFLEAVRK----AL------------PENIELSIAVPARVE-DMMAFTAEN--VAKINGIVNYVNIMTYDLMN-R-RMNETTHHTSV----------------------NGSLNSVNTYLER-GLSPDKINLGFAFYAKWFSTEAGYQC------------TTPTG---------CPTALLENP-DGSD-----------PLV------------------------------S-------GAITFEASTYA------------------------------------------------------------G---------------KLEHAVANG-IADEE----EGGQWWW--DAAEEVYWTWDTADLVARKFSEIVVPKKLGGVFAWSLAQDSY-----------DWSRFKALQ--------------------------------------------------------------------------------------------------------------------------------------------------------------------------------------------------------------------------------------------------------------------------------------------------------------------------------------------------------------------------------------------------------------------------------------------------------------------------------------------------------------------------------------------------------------------------------------------------------------------------**

**>06020_N_crassa_A_II**

**MIFLKLPL----------------------------------------------------------------LLGA--------------------------------------------------------------------------------------------------------------------------------------------------------------------------------------------------------------------------VMLAMMGGSGVM---------------------------------------------------------------------------------------------------------------------------------------------------------------------------------------------------------------------------------------------------------------------------------------------------------------------------------------------------------------------AATSRPLRCIMYLTG--QHPVAP-------QIDQ--LDHVTHVALAFMSPS-------IFNDP----------LN-------------------------QEW--PLFTTVHHVRPRFPNHTKVLIAIGGWG-DTIG----FSVAALNDETRTLFAANVAKMVEATGADGVDVDWEYPG---------GN-GEDYKQIPNSDKRWEIAAYPLLLQELRS----AL------------GPDKVISAAVPGLHR-DMLAFTRET--VPRIMRHVDFLNVMTYDMMN-R-RDTVTKHHTGV----------------------ELSLQAVDAYVAA-GAAPQKLNLGFAFYLKYFKTEQDACA-Q----------TSPVG---------CPTVEMEDPVSGAD-----------LGR------------------------------S-------GAFSWHDDVPK---------------------------------------------------------HLRA---------------SYDRALAQG-VYDHD----QGGYYYW--DPTEALWWTFDTPDAIKQKFPKVMEKRRLGGVFAWGLGEDAP-----------NYDHLAALNEGC---------VAFAISWPKTPAAFI-----FSFSSSSISS--------------------------------PTIEQIQHC-------------HLPSVWLTKPDR------------RRGWAYF-----------------------------FAERGHRVYAPHLPFH---------------------------GRSGALVHYERVKAITPQTVENLYTAINK----------------TNNP-----------EWPAAKTHTQWPGSGVRGDPIFDEYVKQLVPLYMDPHERQA--AAQEAVASLLRF-------LKRPAILVGQGSG----ANVAWLVADVAP--DLIHAIVAVEPLGPPFGS-------ALGNQNGQLGPTSGFTRAAGIRQYGLADIPM--QFDPPVAAPESFEELFGGTYPAFEPIPVRKYYARGRQGKFCFEQGPAGLPVGNSTESRAEPRKLTNLIEILQLVVTTEASFHRFSDW-ATVHFLRQAGATVKHHRLEEHGIHGNGHLCFLEKNSNDVANHVLNWLHNETGVPKLQVLEARPSPSQAINPSGDHRQSLILPPEKTCPTKKETSRN--------**

**>09277_P_nodorum_A_II**

**MAL---------------------------------------------------------------------LIGL--------------------------------------------------------------------------------------------------------------------------------------------------------------------------------------------------------------------------SFWLLLMAAAAAA-------------------EP--------------------------------------------------------------------------------------------------------------------------------------------------------------------------------------------------------------------------------------------------------------------------------------------------------------P--------------------------------FLTQGKLRWIMYLTG--QHNVVP----EYPMETM--LRGVTHVTLAFMRSD-------IFNDA----------NR-------------------------TEW--PLFTTVDEVRPKFNNGTRVQVAIGGWG-DTAG----FEKGAKTEKSRKLFASNVKAMLEATGADGIDMDWEYPG---------GN-GEDYKVNPNSSKVWEITAFPELLAAVRA----AV------------GPLMTISAAVPGLER-DMLAFTPAT--IPRIMESIDFLNVMTYDMMN-R-RDNVTKHHAGT----------------------QQSLAAIKAYRKR-GVPADRLNVGTAFYVKWFKTANGKDC-S----------AQATG---------CPTELMEDPKTGAD-----------LGK------------------------------A-------GAFSWHDQVPS---------------------------------------------------------ELSN---------------SFDKALKHG-VCDRE----SGGCTYW--DADERLFWSWESKDTIYRKLRVVIGEMKLEGAFAWGLGEDAP-----------EFEHLSGANHGI---------SDEEISR-----------------------------------------------------------------------------------------------------------------------------------------------------------------------------------------------------------------------------------------------------------------------------------------------------------------------------------------------------------------------------------------------------------------------SS---------------------------------------------------------------------------------------------------------------------------------------------------------------KIDAMRNAARWRNEL**

**>02644_P_nodorum_A_II**

**MRA---------------------------------------------------------------------------------------------------------------------------------------------------------------------------------------------------------------------------------------------------------------------------------------------------SLLSPLL--------------------------------------------------------------------------------------------------------------------------------------------------------------------------------------------------------------------------------------------------------------------------------------------------------------------------------------------------------------------------FAASVTARFVMYADE--WHPTRP------TNPKD--RSGIDHVVIAFAMAN----------------------AT-------------------------ASF--QPKVPISTIRSEF-PGAKLMIAVGGWG-DDVG----FLQASKTDAAIAQFAADVGTMLTNTGADGVDIDWEYPG---------GN-GGDYKEVLNSDKAYQIEAYPKFLAAIRA----AI------------G-NKLLSIAVPGKKG-DMIAYTKET--GPKIWRSVDFINVMSYDLMN-R-RDNVTKHHSSV----------------------VDSAATIERYLAI-GAPPEKLNLGFAYYAKYFTTKPGCTS------------NPPLG---------CPIVLAEDPVTGKD-----------TLT------------------------------S-------GAWTFEPSHMKPVDASALTVSYDGTCGPDKGTKCSSG-CCSQYGNCGNSPEHCSGACQHAFGTGCTDPDVAG---------------SWQSALAGG-MTDNR----AGGQYFF--DTENNLFWTWDTPALISRKFDDIVRKYKLGGVMAWSLGEDSF-----------DWSHIKAMADEL---------EKGGYGS-GEPSA--------QSSQPKIPT---------GSDSAPLEQGPV----------DPQAPNIASA-------------PTPSATF--------------------------DVVWVDG------------------TSEGPAGDHATQPGSPD------------------------------TFVASQQQNEQASTPQ---------------------------QKNP-LPIPIPIQP-ASPSPSTRTRRP----RPRP--------------RPRPRPSPRPLRRPRPSPLR---------KPTPILPAAPSNTVILASPAQTASS-----VLPSPSFNALPFSPEFLA-------ALGGEEGR-------RRELHRRQGGGVDLE---QEGGEGAAEEE-----GGEEEAFAYM----WY----------------------------------WID--------------FAD----------------------------------------------------------------------------------------------------------**

**>AN0221_E_nidulans_A_II**

**-------------------------------------------------------------------------------------------------------------------------------------------------------------------------------------------------------------------------------------------------------------------------------------------------------------------------------------------------------------------------------------------------------------------------------------------------------------------------------------------------------------------------------------------------------------------------------------------------------------------------------------------------------------------------------------------------MYLTGVRQHNVVP-------EPSL--VSDVTHVALAFMQSS-------IFNRN----------ST-------------------------SSW--PLFTTVDAVRTEFSNGTSILISIGGWG-DTQG----FSTAARTDESRKLFAENIRRMVDDTGADGVDIDWEYPG---------GN-GEDYKRIPNSEKFWETAAYPQLLAEIRA----AI-------------PDKLITAAVPGLPR-DMLAFTKET--IPSISASVDFLNVMTYDLMN-R-RDTVTKHHTGV----------------------QLSLESIDAYIEA-GMPPEKLNLGLAYYVKWFKTVPGEQC-A----------KQPIG---------CPTVLMEDPDTGAD-----------LGQ------------------------------A-------GAFSWNDRVPQ---------------------------------------------------------ELAA---------------SFEKAKISG-EYDPF----GGGYYFW--DSNENIFWSWDTANAIKRKFPAILEKRKLGGVFAWGLGEDAP-----------EFAHLKATTAGL---------GDSSAAP-----------------------------------------------------------------------------------------------------------------------------------------------------------------------------------------------------------------------------------------------------------------------------------------------------------------------------------------------------------------------------------------------------------------------GG---------------------------------------------------------------------------------------------------------------------------------------------------------------KLSTSRK-----DEL**

**>MGG_06594_M_grisea_A_II**

**MKF---------------------------------------------------------------------------------------------------------------------------------------------------------------------------------------------------------------------------------------------------------------------------------------------------SLSALAL--------------------------------------------------------------------------------------------------------------------------------------------------------------------------------------------------------------------------------------------------------------------------------------------------------------------------------------------------------------------------ANAVSATRWMVYYDQ--YHITPP------PNKTL--TAGITHVITAFAQSD-------LFLN-----------SS-------------------------GKY--EPFKPISEIRGMFNNGTKVCMAIGGWG-DNVG----FSKAALTDESRKEFAKNVAATVDRLGYDCVDMDWEFPG---------GN-GQDYKQNPNSGRVSEIETFPLLLQDIKA----AI------------G-TKELSIAVPGKEV-DMIAYTAEQ--VPKINAAVDFINVMAYDLMN-R-RDNVTLPHTDV----------------------KGSAAVVDRYIAL-GCEPKKLNLGFAFYAKWFTTAKGVEC------------TTSTG---------CETALLEDA-EGND-----------TGL------------------------------S-------GAFTFEAANFATT-PTNLTLTTDASCGANTFNKCAEGDCCSQYGFCGNTAAHCGTGCQFGYGK-CDGFSTKD---------------SFQKAIANG-KTDEA----LGAQWYW--DSTASLYWSWDTPALMKQKINDIVFAKGLGGGYAWTLGEDTF-----------DYSHLDTLRDGL---------NKKCKA--------------------------------------------------------------------------------------------------------------------------------------------------------------------------------------------------------------------------------------------------------------------------------------------------------------------------------------------------------------------------------------------------------------------------------------------------------------------------------------------------------------------------------------------------------------------------------------------------------**

**>128098_P_chrysosporium_A_III**

**------------------------------------------------------------------------MC----------------------------------------------------------------------------------------------------------------------------------------------------------------------------------------------------------------------------SLRSSTQSAVASP---------------------------------------------------------------------------------------------------------------------------------------------------------------------------------------------------------------TPSPAGGS------------------------------------------------------------------------------------------SVPPTSITG------------------------------------------NSSTDDIVATTWYTG--WNA-----DQLPVDQVS--WDKYTSVTYAFALTQAD--GT-ILLQDS-------------------------------------DV--ALLPQFVEAAHSH--NTRAMLTIGGWT-GSMY----FSPMVATADNRTSFVNAAMQLVSQYNLDGLDFDWEYPG---------AS-G-DCNVNTP----QDTQNFLLFLQELRS----Q-------------APNITLSAAVALTPFVDTSGSPATD--VSGFAKVLDYIAIMDYDVFG-PGWSKVAGPNAPLNDTCAPSGQQ-----------DGSAVSAVQAWTSA-GFPANQIVLALAAYGHGFVVDSTAAF------------DTSNS--------GTTLLAAYPPVANSS--------NP-QGDPTDVWSAGGAVDE-C--------G-RVTQPG-----WGGVWNFAGMISGG--------------------------------------------------------YLDQ--N------------GTAVAGLGY-RFDTC----SQTPYIY--LEDRQTMVAYDDPTSIAAKGK-FIAEQGLRGFAIWEAAGDY------------KDLLLDAVSNAI---------GIEEVGC-------------------------------------------------------------------------------------------------------------------------------------------------------------------------------------------------------------------------------------------------------------------------------------------------------------------------------------------------------------------------------------------------------------------------------------------------------------------------------------------------------------------------------------------------------------------------------------------------------**

**>39872_P_chrysosporium_A_III**

**----------------------------------------------------------------------------------------------------------------------------------------------------------------------------------------------------------------------------------------------------------------------------------------------------------------------------------------------------------------------------------------------------------------------------------------------------------------------------------------------------------------------------------------------------------------------------------------------------------------------------------------ATASV------------------------------------------TNATVDMIAAAYYAG--WHGVGNSYYNFTLDDVA--WTKYTHLIYAFAVTDPAAPEN-ATLADS-------------------------------------DV--ELLTEFVAKAKNN--SVKAMLSIGGWT-GGRA----FSTNVGSAENRTAFVKTVVGLKNQFQLDGIDFDWEYPN---------DA-GIGCNAVSP----NDTANFLTFLQELRA----DP-----------DGKDLILSAATPVLPWKDETGSPSTD--VSGFSKVLDFITIMNYDVWG-S-WSSAVGPNSPLNDTCAAAANQ-----------MGSAVSAVKAWTAA-GMPAHQIVLGVASYGHSFTVPPSAAF------------TSGDD----------NTLAAYPAFNSTS--------FP-LGDGWD--GP-QGTDV-C--------GVTNNNPG-------GNWDYWGLVSEG--------------------------------------------------------WVWP--N------------GTARPGIES-RFDEC----SQTPYVY--NKTSQIMVSFDDPAAFKAKGE-FILSAGLRGFNIWEAGGDF------------KDYLLDAISVAT---------GM------------------------------------------------------------------------------------------------------------------------------------------------------------------------------------------------------------------------------------------------------------------------------------------------------------------------------------------------------------------------------------------------------------------------------------------------------------------------------------------------------------------------------------------------------------------------------------------------------------**

**>138098_P_chrysosporium_A_III**

**MWTIP-------------------------------------------------------------------AL----------------------------------------------------------------------------------------------------------------------------------------------------------------------------------------------------------------------------GFLSLL-------------------------------------------------------------------------------------------------------------------------------------------------------------------------------------------------------------------------PAGIRAVPV-----------------------------------------------------------------------------------------------------------------------------------------CTGTPAAIATTWYTG--WHA-----QHLSLEDIS--WEKWSSISYAFAYTTPD--PAVVALQDS-------------------------------------DA--DILKRLVDMAHQN--HVDVLLTVGGWT-GSRY----FSTAVGSAENRTAFANALLGLVQQYNLDGIDIDWEYPN---------KQ-GLGCNVISP----QDTPNFLALLQELRA----SP-----------AGQNMTISTATSIVPFTSPDGTPTAD--VSPFAAVLDYVTLMDYDVWG-A-WSPAVGPNAPLADACAAPEHQ-----------QGSATSAVAAWTRA-GLPASKIVLGVAAYGHSYHVAPADAF------------GGASSAG-EAQPPGTTPVAPYPPFDAAQ--------QP-MGDVWDEASP-AGTDA-C--------G-NPVAGG-----FSGIYNYRGLVEKG--------------------------------------------------------YLDA--N------------GTAVSGMGF-RYDTC----SETPFLY--DPNTSTMISYDDPQSFAAKGR-FIHESRLLGFAMWEAAGDY------------EDMLLDAISDGL---------GIGEDACQGTMSL--------------------------GLP-------------------------------------------------------------------------------------------------------------------------------------------------------------------------------------------------------------------------------------------------------------------------------------------------------------------------------------------------------------------------------------------------------------------------------------------------------------------------------------------------------------------------------------------------------------------L**

**>129436_P_chrysosporium_A_III**

**--------------------------------------------------------------------------------------------------------------------------------------------------------------------------------------------------------------------------------------------------------------------------------TGQGRPTRRG------------SATTPTSSTSASAPSAR--------------SHP------------------------------------------------------------------------------------------------------------------------------------------------------------------------------------------------------------------------------------------------------------------------------------PSSSAAAAAPAHPTSAP------------------------------------------AAPQTRPLVMAYYPS--YTA-----ASFPPERVD--FTRYDWVDFAFAVPQAG--GG-LGWDGA---------DG-------------------------SAG--DVLRRLVARAHAA--GKHVKVSVGGWT-GSAR----FSGAVAGADGRGALVDAIVRLYGAYALDGIDIDWEYPG---------QP-GADGNAYAP----QDTANFLAFLRLLRQ----RL------------PPQAKISAAAMTVPWADAHGAPLAD--ASAFAQVLDWILIMNYDTWG-S--ASPPGPNAPLSNACHNSTQA-----------SASAVAGIAAWTAA-GFPASKIVLGVPSYGYISHSSADGLQ------------TRSG-------------------TDGAGSAGVPVVLNE-DGNAAD-----------------------------------GEVQFRDLVQQG--------------------------------------------------------VLHT-ADTLSFAGHTIANVFTGLRGFER-RWDAC----SSTPFLR--SARAGQVVAYDDPQSLELKAA-LARYAGLRGVNMFDAHGDTD-----------AGDLVDAIRRGL---------GL------------------------------------------------------------------------------------------------------------------------------------------------------------------------------------------------------------------------------------------------------------------------------------------------------------------------------------------------------------------------------------------------------------------------------------------------------------------------------------------------------------------------------------------------------------------------------------------------------------**

**>MGG_04732_M_grisea_A_III**

**M-----------------------------------------------------------------------------------------------------------------------------------------------------------------------------------------------------------------------------------------------------------------------------------------------------SLVNLSRSALALL-------------------------------------------------------------------------------------------------------------------------------------------------------------------------------------------------------------LAASNFAGVNAFP---------------------------------------------------------------------------------------AASDVAAAA------------------------------------------TQGPAGKVSTGYFAG--YHAKRKG-VTFGVDQIP--WDKYDDVKYAFAETTAD--GS-LDLSKS-------------------------------------AP--EELPKFVDAAHAH--GKKAQVALGGWA-GSIH----FSSNVGSAENRTAFVKTCLDLVQKYNLDGLDFDWEYPN---------KQ-GLGCNAISP----NDTANFISFLTELRK----AQ------------PKPLLLTAAVSLYPYNDAAGQQSANGALKPMGDLLDYSIIMGYDVFG-A-WAPTGGPNAPLEQACDPRNN------------QGGIKEGVAKWVAA-GVPAEKLILGIGAYGHGFTVSQADAF------------VPTTD----GKP---FPLSAYPKNNGTRR----------QGSSWD--DD-PAVDA-C--------G-AAQPAG-------GTFTFWSMVTEGQ-------------------------------------------------------FLDE--S------------GKPRAGIAY-GYDDC----SQTPFVY--NNATSWWVSYDDSKSLGVKGK-YITDNKLAGFSVWELGGDY------------KNIVVDAMRGSV---------GL------------------------------------------------------------------------------------------------------------------------------------------------------------------------------------------------------------------------------------------------------------------------------------------------------------------------------------------------------------------------------------------------------------------------------------------------------------------------------------------------------------------------------------------------------------------------------------------------------------**

**>UM06190_U_maydis_A_III**

**MKLLPLG---------------------------------VLALAS--------------------------LFTSSLAA---------------------------------------------------------------------------------------------------------------LNND----------------------------------------------------------------------------GSAHFGRTANGQVVAGSSYADSSSTLSFAADRAASARST--------------------------------------------------------------------SSSGRFSI----------------------------------------------------QRRSLWQRLFGNSAPGSQRKRRAVPSARLLARQRPSRL---------------------GTGSS-RKTKRTRGSCKAPTTASASAAASA--------------------------------------------------------------------------SSSTSTSASIPPSPTKSVNE------------------------------------------TRTSTNKLMAGYWAD--WTA-----STLPATSID--FSKFDIVNYAFALPTAE--FD-LWIPTD------------------------------------PSG--NLLRGFVKAAKAG--NAKPMLSLGGWG-GSTY----FSPAVRTAASRATFISNIVKTYNQYGLDGIDLDWEYPG---------QA-GSG-NLLDA----SDTRNYQTFLTELRA----AL------------PKGALITGAVAHTPWMASNGQPVAS--VARAAAAMDYIMIMNYDVWG-S--SSNPGPNAPLANLCGNSTQP-----------RANAAAGVKAWSAA-GMPRDKILLGIPAYGYINTSKKQTLR------------TRSRS------------RSSSSQRRAAK------LTSS-DGSTS----------------------------S-------GQINFSTLVSQG--------------------------------------------------------ALKLGAD----------GLFDGAGGFTK-YWDDC----SDTPYLS----DASRVITYDDTSSIFDKGA-FASAAGIAGISMWSLDGDTE-----------SWALTNSAIAGM---------SSTGAISS------------------------------------------------------------------------------------------------------------------------------------------------------------------------------------------------------------------------------------------------------------------------------------------------------------------------------------------------------------------------------------------------------------------------------------------------------------------------------------------------------------------------------------------------------------------------------------------------------**

**>04245_F_neoformans_A_III**

**MFLSTPA---------------------------------VLSFVL--------------------------LLASQSSAQGHIAPPGRHRRPRIFKDLQERGQLDERLLPITATTTVLVPMTATRTIWVDSTQLVLGNGGDDGDSDVSGDASSVWSSQASQSTEGASTSSEAMLPTASASSSVSDLTSSSYNTDTQSSSFATGSYIEDVTATSSLLSDSLPTSSSISS----------DYPSFSASQSEAPSSIIGVVTS-SATVTDSSFSTSAGEPTSTGSPSSQST--DTDSAPSPPTSASASTGSGS--------------SLPTSLPQTESATRSEDLGSESTSIASSAIMSDNVSGSESQSASVSSTESFSISETGSASLSGSESVSLSDSFSISATSTDSAAIEAASSSYSGFEPSATASETASASANETGSKTKSQGNWWETSTSSSDWASATEASSSLSTSETASES---------------------ASLTA-SSTASVSDSLTWSENATISSAATA--------------------------------------------------------------------------TISESSTSTSATSTSSSSSA------------------------------------------TSTLASTLMLGYYPD--WSA-----YYLSPESVD--WDRFDILDFAFAIPNSD--GS-LYFTDD------------------------------------SST--DSLQRLVSSGHAA--GKRVKLSIGGWT-GSAY----FSTIVANDALRATFVSNIYDIYNQYNLDGIDIDWEYPG---------TA-GADGNAVSS----DDSANFLIFLQDLRA----AL------------PSEAIITTATQVWPFADSNGNPMTD--VSEFAKVLDWILIMNYDVWG-S--SSTPGPNAPLSDGCGNSTQP-----------LANAYAAVASWTSA-GMPANQITLGVPAYGYIQVSSASSLI------------QRRS-----------LPLLPHKRSKHAKKASHVTVQNE-SGGTTD-----------------------------------GQVMWYGLLNQG--------------------------------------------------------ALTL-SD----------GQYVGAGGFTR-YWDSC----SSTPWLK--SSESGQIVTYDDTESMNLKAQ-FAAQAGLRGCNVFSVDGDWT----G-----SSWPLTDAVRSGL---------GL---------------------------------------PAV------------------------------------------------------------------------------------------------------------------------------------------------------------------------------------------------------------------------------------------------------------------------------------------------------------------------------------------------------------------------------------------------------------------------------------------------------------------------------------------------------------------------------------------------------------------**

**>16170_R_oryzae_A_III**

**MRHILFT-----------------------------------------------------------------LLT-------------------------------------------------------------------------------------------------------------------LC------------------------------------------------------------------------------------------------------VVFSYTALAASSHSKG--------------------------------------------------------------------HISKHKSLSHHHTNKRTHYKTIHH---------------------------------------------------HSSGKKKPTKKSKKSTKHSSKHL----------------------------SKKHTKKHTTKHTKKHTTKHVVK--------------------------------------------------------------------------HTT---KHTTKHTSSNKKNT--------------------------------------PNASSLTFPNHKVVAYVLD--WDI---------PKNIK--WDKLDHIAYAFAEPNAN--GV---LKGF-------------------------------------DG--NNLKSVVTQAHKN--KVGVSISIAGWS-GSIH----MSTLVGSVSKRNTFVRNIVNMVKKYKLDGVNIDWEFPN---------SPDSIANAARNP----NDTANLLIFIKLLRQ----KLDTTF-------PKAHKVITVAASVGPFLDERGRSIRH-FEPDWVTNVDYFYLMSYEYNG-N-WNDVAGPNAALWGSSH----------------GWGVDTTVNLWYNA-GIPKNKMHVGVTFYGKVLKTAQPITS------------TSG----------------MYVKLDGHT--------QI-KGDKYD--E--SDADP-C--------PNCKPSYT-------GEYQWRSIVANG--------------------------------------------------------ILE------------------NKNGWTT-YWDEQ----SQTPYAY--HAGKHTFLSYDNTKSFEAKLN-YVKSEGLAGFMIWSLEMDDS-----------KHTLLNTLQGVRK----------------------------------------------------------------------------------------------------------------------------------------------------------------------------------------------------------------------------------------------------------------------------------------------------------------------------------------------------------------------------------------------------------------------------------------------------------------------------------------------------------------------------------------------------------------------------------------------------------------------**

**>01334_R_oryzae_A_III**

**MRNILVI-----------------------------------------------------------------LLS-------------------------------------------------------------------------------------------------------------------LC------------------------------------------------------------------------------------------------------VLFAYTASATSHHS-----------------------------------------------------------------------THKHKSLSHHST-KRAHHVTAH---------------------------------------------------------------------HSSKH----------------------------------KKSTKKITKKKTNKKVTK--------------------------------------------------------------------------KSK---KHTTKKASVKKVN-------------------------------------------DKSYPNHKVIAYVLD--WDI---------PKNIQ--WSKLDHIAYAFAEPDAK--GN---LKGF-------------------------------------ES--GNLKSVVTQAHKN--NVGVSISVAGWS-GSIH----MSTLLNDVQSRETFVKNVVNMVQKYSLDGVNLDWEFPN---------SRDSIACASRNP----NDTANLLTFIQLLRK----SLDAKY-------PKVHKVITVAASVGPFLDSNGHAVRH-FESGWVSDVDYFYLMSYEYNG-N-WNDVAGPNAALRGSSR----------------GWGVETTVNLWYNA-GIPKNKMHVGITFYGKVLKTAQPITA------------TSG----------------MYVKLDGHT--------QI-KGDKHD--E--LAADP-C--------PNATPGYT-------GEYQWRSIVANG--------------------------------------------------------ILE------------------NKNGWTT-YWDEQ----SQTPYAY--HAGNSTFLTYDNPKSFEAKLD-YVKSQGLAGFMIWSLEMDDS-----------KHTLLKSIQGVRK----------------------------------------------------------------------------------------------------------------------------------------------------------------------------------------------------------------------------------------------------------------------------------------------------------------------------------------------------------------------------------------------------------------------------------------------------------------------------------------------------------------------------------------------------------------------------------------------------------------------**

**>13934_R_oryzae_A_III**

**MKV--------------------------------------------NV-----------------------FF----------------------------------------------------------------------------------------------------------------------------------------------------------------------------------------------------------------------------LFISFILYVLAQD--------------------------------------------------------------------------------------------------------------------------------------------------------------------------------------------------------------------------------------------------------------------------------------------------------------------------------------------------------------------RPIFGSHKLVAYIVD--WDL---------PRTIY--WDKLDHVSYAFAVPNKE--GD---LNGF-------------------------------------NK--TQLNQVVKEAHHH--GKGVSLAIGGWT-GSIY----FSSLVRTEASRKSFATNLINAVEEYDLNGLNIDWEYPN---------DPRGIFCNRRNL----QDTSNFLALIQLLRE----TLDKKY-------PKEHKIITAAVSTYTFKDDKQNSIKT-LDDGWSKYMDAFYIMAYDLNG-I-FSQNSSANAPLEISNMTNP-------------TTSGAAAVQSWISA-GIPSNKIYLGVPFYGYTHKTTAEINL------------ETG----------------IHVPFDRKIP-------QI-KGDSYD--D--FAADP-C--------PGANRSFS-------GEIQWRTIEQRG--------------------------------------------------------IDR------------------NASGWAT-FWDST----SQTPFAY--KQDNQQFVTFDNPTSLRIKSN-YVNENRLGGVMLWSLEMDDT-----------DHSLLEALQDVRT--------------------------------------------------------------------------------------------------------------------------------------------------------------------------------------------------------------------------------------------------------------------------------------------------------------------------------------------------------------------------------------------------------------------------------------------------------------------------------------------------------------------------------------------------------------------------------------------FI---------S--------------**

**>07611_R_oryzae_A_III**

**MKII--------------------------------------------------------------------------------------------------------------------------------------------------------------------------------------------------------------------------------------------------------------------------------------------------SLVSCTAGLYLLSS------------------------------------------------------------------------------------------------------------------------------------------------------------------------------------------------------------------------------I-------------------------------------------------------------------------------TQASPVSKLQG------------------------------------------RDISSDKVVVGYFPN--WLY-----GRYTPSKID--FSKYTHIYYAFAIQNTA--SS-PTWSDS--G----VFDS-------------------------YVA--YGFPKLISLAHAA--GTKVLVSIGGWS-GSTQ----FSTMAASQANRQAWIDWNINFIKQYNTDGVDIDWEYPT---------SK-GAGCNAVND----NDVQNLNTLVKELRS----ALDNSF-------PNDHKELTMAVHITPFGGA--TPVTD--ASGFVPYVDRFHVMTFDVNG-A-WNSTSGPNAPFHNTPGWGYG-------------AGFVEGIQSWNAA-GVPYKKLAGGIPFYGRAQTLSVD---------------SAP--------------KTQYNPAVSPN--------PP-LGDQLD--G--PWQDAYC--------SSDSQSAS-------GVWRWAYLRSQG--------------------------------------------------------ILTS--P------------TTAASPWIR-NFDDH----TQTPWLY--NPTNKQYISYDDPVSLKVKVQ-YAIDQGLAGLFAWSVDED-------------NGELLSVIASILDENPTKTSIGTSTGTATGATNTTT-----AGMTTNSASS-TSS-GGCANVPTW-----------------------------------------------------------------------------------------------------------------------------------------------------------------------------------------------------------------------------------------------------------------------------------------DASTAYTGGSQV--------VYN--------------------------------------------------------------DKLYKA-----------KWWNQG------------ETP---------------------------SLNDSSWSVWTNI--------------------------KC--------------------------------------------------------------------------**

**>10252_R_oryzae_A_III**

**----------------------------------------------------------------------------------------------------------------------------------------------------------------------------------------------------------------------------------------------------------------------------------------------------------------------------------------------------------------------------------------------------------------------------------------------------------------------------------------------------------------------------------------------------------------------------------------------------------------------------------------------------------------------------------------------MVIGYFPN--WLY-----ANYPVENIP--YTKYTHINYAFAILNNP--DNLPSFTDD-W----------------------------------AVE--SSFPKLVQMAHQS--KTKVLLSVGGWT-GSKR----FSPMVASKESRRNFINWNVEFMEKYNTDGVDIDWEYPG---------KQ-AAGCNEVAD----DDADNFLLLLKELRL----ALDTKF-------PQQHKEISMAVHVQPFVRS-GTPMSD--VKEFVPYFDHVNIMTYDMNG-A-WASTTGPNAPFKYLEGKGAP-------------FSFTDSIKQWKLA-GVPSEKITAGLAFYGRAMKANVNMI-------------TDP--------------TNQYQPSQLG---------AP-KGDSDD--A--YWQDPYC--------NVEPGGLS-------GIWKWTNLRKEG--------------------------------------------------------ILKD--D-----------FITPGEGWVR-HWDDV----TQTPWLF--NPTTQHYITYDDPQSLNIKVQ-HALCEDLAGVMGYPSR--------------------------------------------------------------------------------------------------------------------------------------------------------------------------------------------------------------------------------------------------------------------------------------------------------------------------------------------------------------------------------------------------------------------------------------------------------------------------------------------------------------------------------------------------------------------------------------------------------------------------------------------------**

**>14659_R_oryzae_A_III**

**MK--G------------------------------------------------------------------YLF----------------------------------------------------------------------------------------------------------------------------------------------------------------------------------------------------------------------------VILSLYF---------------------------------------------------------------------------------------------------------------------------------------------------------------------------------------------------------------------------------------------------------------------------------------------------------------------------------------------------------------------IKVLHAATKNDKVIIGYYQS--------------FSKID--FDRYTHINYAFAVMIKG--NT-PVWSDP---------E--------------------------SIE--TQLPQLVSAAHKK--NVKVLTSIGGWT-GSLT----F----------------------RFKLDGIDIDWEYPG---------RQ-GAGCNTFDEK---NDVKNFLTLLKELRQ----AIRRE--------FKEKKEITIAAFVNPL-------AAD--VSGFAKVLDRVNVMTYDING-A-WNAQTGPNAPLNAPQG----------------QISFSSSIESWIQA-GMPAHQLTGGLAFYGRSTTAKVNMLK------------KKS----------------LYQDQVQGK--------IP-QGDQDD--T--FSQDPYC--------SKDPGGLS-------GTWQYSHLLSQQ--------------------------------------------------------VLKT--A------------QKARKPWVR-TWDKA----TSTPWLF--NPKTNIFISYDDPKSISKKAC-YAHNKHLAGVMVWAINQDTD-----------QHDLLKAAYKVKT--------GK---------------------------------------------------------------------------------------------------------------------------------------------------------------------------------------------------------------------------------------------------------------------------------------------------------------------------------------------------------------------------------------------------------------------------------------------------------------------------------------------------------------------------------C--------------------------------------------------------------------------**

**>EAA69503_G_zeae_A_IV**

**M------------------------------------------------------------------------------------------------------------------------------------------------------------------------------------------------------------------------------------------------------------------------------------------------------------------------------------------------------------------------------------------------------------------------------------------------------------------------------------------------------------------------------------------------------------------------------------------------------------------------------------------AS------------------------------------------NLSDVMFTNAVYFPN--YRIY----QGDTPGMLN--YSCINHVYYAYASVSAD--GG-VFLSDE-WADAGAPVDG--------------------------VQ--GGLGSLMHLKQKH-PHLRVVLSIGGGN-SSEI----FPVVASSTLLRDNFARSARGLVEASGLDGIDIAWEYPC---------DA--------------QQGYDFLALLAAVRI----HL-----------PEEHYVLTAALPA----AKAVLQFLD--LMEIAEYLDFINLMAYDFFG-S-WTPKAGHHAQLYAMDKE---------------ETSASSGVSYLMSK-GFPPKKILLGIPTFGRSFLHC----------------------------------TGAGHKYKGI-------------GGAE----------------------------D-------GTFEYNQL---------------------------------------------------------------------------------PRKGCKE-VVDKR----HVAAQCM---GADGGFVTYDNPDTVKMKAG-FCKQKGLGGLFYWNAPADSK---DK------SRSLVAAGFRAL---------HSSSS---------------------------------------------------------------------------------------------------------------------------------------------------------------------------------------------------------------------------------------------------------------------------------------------------------------------------------------------------------------------------------------------------------------------------------------------------------------------------------------------------------------------------------------------------------------------------------------------------------**

**>EAA70860_G_zeae_A_IV**

**M-----------------------------------------------------------------------------------------------------------------------------------------------------------------------------------------------------------------------------------------------------------------------------------------------------------------------------------------------------------------------------------------------------------------------------------------------------------------------------------------------------------------------------------------------------------------------------------------------------------------------------------------------------------------------------------------PTQYINAVYYPS--WRCY----KERPPSCLD--ISSITHIFYAFVGQVSS-FKQHFQAIDD-WADNEKMVDG--------------------------EK--GCLAAISKLKSQH-PHIKTLVSIGGGS-SSKE----FPALAANKTARQTFARRISEFCVTHQFDGVDIDWEHPQ---------TP--------------EAGRNYVLFLQDIRN----VM-----------THSQFLLTSALPT----GEYCLKHIN--LPVVAHLLDFLNLMGYDFTG-G-WTDVCGHHAQLLPPSQNLNEVY-------PTLRKSCQRGVDFLIAN-GFPRHKIILGIPVYARYFGQA----------------------------------RGPGHPFKGA-----------------------------------------------------GEIDYCDL---------------------------------------------------------------------------------PDEWVTNAEVDQS----VAAASFV-DNKSDKGFVSFDVPSTVSIKAG-YAKALGLGGLFYWTGAGDRK----G------CESLVTAGWTAL---------NSQ-----------------------------------------------------------------------------------------------------------------------------------------------------------------------------------------------------------------------------------------------------------------------------------------------------------------------------------------------------------------------------------------------------------------------------------------------------------------------------------------------------------------------------------------------------------------------------------------------------------**

**>Chi18-3_H_jecorina_A_IV**

**M------------------------------------------------------------------------------------------------------------------------------------------------------------------------------------------------------------------------------------------------------------------------------------------------------------------------------------------------------------------------------------------------------------------------------------------------------------------------------------------------------------------------------------------------------------------------------------------------------------------------------------------AT------------------------------------------SVARVMYTNAVYFPS--SRIY----QGDSPGMLN--YSCINHVYYAYASVTAD--GN-VLLGDE-WADARAPVDG--------------------------VH--GGLGSLMHLKQRH-PHLQVVLSIGGST-ASEV----FPIVASNTLLRDNFARSALGLVEASGLDGIDIAWEFPS---------EA--------------RHGQDFLALLAAVRI----HL-----------PDDRFILTAVLPA----AKEVLQLID--LARAAEYLDYINLVAYDFFG-T-WTSKSGHHAQLYTLNKD---------------EPSASSGVAYLMAQ-GFPAKNILLGIPTYGRSFLKS----------------------------------NGPGQEFNGV-------------GG-Q----------------------------D-------GTFEYSEL---------------------------------------------------------------------------------PRKGCKE-IVDRR----YIAAQCV---GGDGGFVTYDNHETVKVKAE-FCKQKGLGGLFYWNGPADSR---DQ------SRSLIAAGFRAL---------HTS-----------------------------------------------------------------------------------------------------------------------------------------------------------------------------------------------------------------------------------------------------------------------------------------------------------------------------------------------------------------------------------------------------------------------------------------------------------------------------------------------------------------------------------------------------------------------------------------------------------**

**>Chi18-2_H_jecorina_A_IV**

**----------------------------------------------------------------------------------------------------------------------------------------------------------------------------------------------------------------------------------------------------------------------------------------------------------------------------------------------------------------------------------------------------------------------------------------------------------------------------------------------------------------------------------------------------------------------------------------------------------------------------------------------------------------------------------------------MNGVYYPS--WLVY----KGKTPATLD--IDNITHLTL-------------VQWIDE-HADVVKDIDG--------------------------ER--GALAALAKLKRNH-PRLKTIVSIGGGT-ASKE----FPALAANSHARETFAKQAREFCDRHVLDGIDIDWEHPS---------NA--------------EQGRDYVKLLKECRR----AL-----------PEPNYLITTALPV----GQYILRHID--LDAVSRIIDYLNLMAYDFTG-S-WTKVCGNHAQLCSPGTRLQSTH-------PELQACATDGVDYILSR-GFPSRKIVLGIPAYARYFPRA----------------------------------EGPGCSTEKA-----------------------------------------------------GEMDYCDI---------------------------------------------------------------------------------PDEWVRQAVVDEA----AVAAWYV-DANGDKGYLTFDVPRTVHMKAR-FAMQRGLGGLFYWTGTGDKG----E------GLSLVAAGRRGL-------------------------------------------------------------------------------------------------------------E-----------------------------------------------------------------------------------------------------------------------------------------------------------------------------------------------------------------------------------------------------------------------------------------------------------------------------------------------------------------------------------------------------------------------------LC--------------------------------------------------------------------------**

**>01594_B_fuckeliana_A_IV**

**MSYPYGA-----------------------------------------------------------------------------------------------------------------------------------------------------------------------------------------------------------------------------------------------------------------------------------------------------------------------------PPIPWNSRP----------------------------------------------------------------------------------------------------------------------------------------------------------------------------------------------------------------------------------------------------------------------------------QIDTRRPSQPSIAA------------------------------------------SISNIMYKNAVYYPN--YRVY----RGETPGSLN--FGCISHVFYAFAHVGPD--GG-VFLSDE-WADMQMPVDG--------------------------AAGGGCLGSFMRLKQQH-PHLQLIISIGGGA-ASQH----FPQVAASAATRDHFGKSVRGLVDATGFNGIDIDWEHPS---------DP--------------EQGRNFLALLAAIRI----HL-----------PDERYLLTAALPA----GRWALQNID--LYKAQDYLDLINLMAYDFHG-S-WSQKSGHHAQLYPGNMP--------------DAANGSAAVDYVVST-GFPASKILLGVPVYGRSFLNS----------------------------------RGPGHPYQGC-------------GG-E----------------------------E-------GTFEYKLL---------------------------------------------------------------------------------PRPGTQE-VVDTT----TISASCY---GGDGGWVSYDNPQT---------------GLFYWTGTADAP----G------PRSLISAGFKAL---------HDA-----------------------------------------------------------------------------------------------------------------------------------------------------------------------------------------------------------------------------------------------------------------------------------------------------------------------------------------------------------------------------------------------------------------------------------------------------------------------------------------------------------------------------------------------------------------------------------------------------------**

**>05897_S_sclerotiorum_A_IV**

**--------------------------------------------------------------------------------------------------------------------------------------------------------------------------------------------------------------------------------------------------------------------------------------------------------------------------------------------------------------------------------------------------------------------------------------------------------------------------------------------------------------------------------------------------------------------------------------------------------------------------------------------------------------------------------------------MYKNAVYYPN--YRVY----RGETPASLN--FSCISHVFYAFAHVGPD--GG-VFLSDD-WADMQMPVDG--------------------------AT--GCLGSFMRLKQQH-EHLQLVISIGGGA-ASQN----FAHVAASAATRDHFGKSVRGLVDATGFNGIDIDWEHPS---------DP--------------EQGRNFLALLAAIRL----HL-----------PDERYLLTAALPA----GRWALQNID--LYKAQDYLDLINLMAYDFSG-S-WSHKSGHHAQLYPGNLQ--------------DAANGSAAVDYVIST-GFPASKILLGVPVYGRSFLNS----------------------------------RGPGHPYHGC-------------GG-E----------------------------E-------GTFEYKLL---------------------------------------------------------------------------------PRPGTQE-VVDTA----TVSASCY---GGDGGWVSYDNPQTVKMKAE-YCRQRRLG-----------V----G-----------------------------------------------------------------------------------------------------------------------------------------------------------------------------------------------------------------------------------------------------------------------------------------------------------------------------------------------------------------------------------------------------------------------------------------------------------------------------------------------------------------------------------------------------------------------------------------------------------------------------------------**

**>04750_C_immitis_A_IV**

**MMFKYTG----------------------------------------------------------------------------------------------------------------------------------------------------------------------------------------------------------------------PTY------------------------------------------------------------------------------------------------------GPWPL------------------------------------------------------------------------------------------------------------------------------------------------------------------------------------------------------------------------------------------------------------------------------------GINGATGQSDNILG------------------------------------------PSDISIYFNAVYYPS--WRIY----KKQPPSSLR--LGCVSHVFYAFAWVNPD--GT-VRLSDE-WADDQMPVDG--------------------------TQ--GCIRAFTQLKEQY-TGMKVILSIGGGGTGSQH----FATVAKDPIALQNFIQSAKNMVDRFGLDGLDIDWEHPS---------DS--------------QQGEDYVNLLRLLRE----GL-----------PWPQYTLTTALPA----GEWALRHIN--LIHAQCYVDLINLMTYDFSG-P-WTPHSGHQSQLFSPHTPHSEA----------AGISCHSGISYVLAQ-GVPSSKILLGISVYGRAFPGT----------------------------------TGVGQTHCIS----------N-ETPED----------------------------K-------IVFDYCDL---------------------------------------------------------------------------------PLPGSVE-QHDDR----LCAAFCV---DRNMGFVSYDSTRTVVQKAK-FVREMALGGLFYWHVAADAV----G------KRSLVAAGYSAL---------HDL-----------------------------------------------------------------------------------------------------------------------------------------------------------------------------------------------------------------------------------------------------------------------------------------------------------------------------------------------------------------------------------------------------------------------------------------------------------------------------------------------------------------------------------------------------------------------------------------------------------**

**>04719_P_nodorum_A_IV**

**MRR----------------------------------------------------------------------------------------------------------------------------------------------------------------------------------------------------------------------------------------------------------------------------------------------------------------------------------------------------------------------------------------------------------------------------------------------------------------------------------------------------------------------------------------------------------------------------------------------------------------------------TFDPHHIDHNTTFS------------------------------------------IPLQAMYWNAVYYPN--WRIY----RDQPPVSLN--YDVISHVFYAFAWVKDD--GT-VYLSDE-WADAQIDVAGAG------DV------P--------ATK--GCLNSFALLKRKY-TKLRVVLSVGGGGKGSEP----FAGVAADPAKRQQFAQSALGLVQQFGIDGIDIDWEHPS---------DS--------------KQGQDFIALLADLRQ----FL-----------PGPQYTLSTALPA----GEWALQHIN--LGHAAHYLDMINLMAYDYSG-P-WTSKCGHQAQLFTPQIPHSPE----------AAISGHSAVSYLVRQ-GVPTHKIVLGVPAYGRSFTGT----------------------------------KGVGHGFSGQ-------------AG-E----------------------------E-------GTFEYRDL---------------------------------------------------------------------------------PRPGATE-HVDER----VGAAYCM---GGDGGFVTYDTPQTVRMKAD-YVRQNALGGLFYWTGTGDASSLTQK------DRSLVYNGFLGL-----------------------F-----PQ----------------------------------------------------------------------------------------------------------------------------------------------------------------------------------------------------------------------------------------------------------------------------------------------------------------------------------------------------------------------------------------------------------------------------------------------------------------------------------------------------------------------------------------------------------------------------------------**

**>MGG_08458_M_grisea_A_IV**

**------------------------------------------------------------------------MFTKWKRD-----------------------------------------------------------------------------------------------------------------------------------------KDQYPSQPTQ------------------------------------------------------------------ASFPMPAPGTSGYPSSYQYGYNNNTAAPPPIPPRPYNLSPA----------------------------------------------------------------------------------------------------------------------------------------------------------------------------------------------------VPP-----------------------------------------------------------------------------YPSAANPNMSA------------------------------------------GGMSRQYINAVYYPN--WRIY----KQLPPSKLQ--VNNITHVFYAFIGVNKD--GT-LKHFDE-YADLQIEADG--------------------------VT--GCLAAVRKLKHQN-PHLKTIVSLGGGS-GSAE----FPALATNPRARDTLARATRQFVDQHGFDGVDIDWEQPS---------NT--------------NEGEAYVALLTALRQ----AL-----------PAPRYLLTTALPC----GEWALQHIP--LGRAAALLDMLNLMSYDFSG-P-WAKVSAHHAQLRHPQPARLPPQA-----DGCLRLSAMQAVSYMIVH-GVHPGQIVLGVPAYARFFRGA----------------------------------RALGAGFSEA-----------------------------------------------------GEVDYNDL---------------------------------------------------------------------------------PTADLDRAMVDPE----LCAAWYLCEGEGGKGFVSLDTPATVRLKAG-YVRANGLAGLFYWQGVGDVT---EG------PKSLVAAGMEGL---------RGFGG---------------------------------------------------------------------------------------------------------------------------------------------------------------------------------------------------------------------------------------------------------------------------------------------------------------------------------------------------------------------------------------------------------------------------------------------------------------------------------------------------------------------------------------------------------------------------------------------------------**

**>EAA67655_G_zeae_A_V**

**MSNTSTSLRTITLKDRPLLPKTIRVS-------------DMLNQIFLIAP----------------------LLVQAA--------------------------------------------------------------------------------------------------------------------------------------------LAVPSVEPR-------------HQHLHGHMEHSHGTRTTMSTAVRRQVEQTIEAVPQFIPPKMPYALKPQ-----TRVTKPTAAASNDDDDDN----TVPGFIP--PRMPFNSKAVQQK-------------------STN-------------------EQASPQRLASVAS---------------------------------------------V-------NNKKTTAKDTKDTKDKMEAKETKDKKDSKNTKSTKNTKNTNAQKLAQVQEDDDDKLPTFIPPRSPFRGLKKPQPTQAVPRDTVPEFIPPRNPWAQSKFNTRSTDSDDQDDPEETDAPDSDQRASRVDLLKRDGEDEDDEYFLEEGDDEFSPSDFPNLGEPEDDTVDASDQHSQDTPAANMSKQQGDPEEGINVQGEENTKNYFGGNFDDDDAENTPARLEARGVAKRNMLYFTN--WGTY----EGFNPENLP--VKEITHVLYSFAKVNAK-DGT-VESSDP-WADVQRTYPG--------D--------N-GGGGN-NAY--GCVRQLYILKKQN-RNLKVLISIGGFD-GSPA----LASGVSTQNGRKRFISTAIKLITDWGFDGIDVDWEYPV---------NA--------------QEARNYVLILNGLRK----ALDKYSQDY--K-LNYRFLLTVASPA----GSSHYNTMD--LKKMDPWVDAWHLMAYDYAG-P-WDSTTGHQANVFASRKSPLAT-----------KLSTDATLNDYIAA-GVSPNKIHLGMPLYGRSFANT----------------------------------AGLGKPYDGV----------A-GSPAN----------------------------L-------GVYLLKDL---------------------------------------------------------------------------------PRPGAVT-TYNAD----LMASYTY--DRKKRELVTMDDLKSAQAKAG-YINERNLGGAFYWEARGDRS----G------SASVVAGVSRTL---------G---TLERSNNLLKY-----PTSIYENIRN---------NRP--------------------------------------------------------------------------------------------------------------------------------------------------------------------------------------------------------------------------------------------------------------------------------------------------------------------------------------------------------------------------------------------------------------------------------------------------------------------------------------------------------------------------------------------------------------------**

**>EAA73155_G_zeae_A_V**

**------------------------------------------------------------------------MVAR----------------------------------------------------------------------------------------------------------------------------------------------EDIP------------------------------------------------------------------------TNVPDLV----------------------------------------------------------------------------------------------------------------------------------------------------------------------------------------------------------------------------------------------------------------------------------------------------------------------VADLNGEDEV------------------------------------------SEQATGSVNAVYFVN--WGIY--G-RNYQPMNLP--ASQLTHVLYAFLNVRAD--GT-VYTGDS-YADLEKHYTG--------D--------SWEEPGT-NAY--GCVKQLFLLKKAN-RKLKVMLSIGGWT-WSTN----FPAAASTAATRATFAQSSVALMKDWGFDGIDVDWEYPA---------ND--------------TDANNMVLLLQAVRK----ELDTYSKQY--A-SGYHFQLSIAAPA----GPENYGKLK--MKELGSVLDHINLMAYDYAG-A-WSAFSGHQANKYANAKIPNAT-----------PFNTDQAVSAYVGG-GVPSGKMVLGMPIYGRAFQNT----------------------------------GGLGQGYSGV----------G-SGSWE----------------------------N-------GIWDYKVL---------------------------------------------------------------------------------PKPGASL-VYDRD----AQASYSY--DANTKELISFDTPGMVENKVL-YVKNKSLGGSMFWEASADKT----G------ADSLLGTSAKKL---------G---SLDSTTNCLTY-----PNSRYANIAK---------GLN--------------------------------------------------------------------------------------------------------------------------------------------------------------------------------------------------------------------------------------------------------------------------------------------------------------------------------------------------------------------------------------------------------------------------------------------------------------------------------------------------------------------------------------------------------------------**

**>EAA74986_G_zeae_A_V**

**----------------------------------------MWLSVRRALA----------------------AV----------------------------------------------------------------------------------------------------------------------------------------------------------------------------------------------------------------------------AISQATFCLALLPR------------------LP-----------------------------------------------------------------------------------------------------------------------------------------------------------------------------------------------------------------------------------------------------------------------------------------------PYTKEI------------------------------------------AQQSKNPVNAVYFTN--WGIY--G-RNFQPQDLP--ASEITQVLFAFLNVKPD--GT-VYTGDA-YADLEKHYQG--------D--------RWDDQEE-NAY--GCVKQLFLLKKAH-RHLKVILSIGGWT-WSTN----FPSAAGTRENRIRFSKSAVTLMKDWGFDGIDVDWEYPN---------DE--------------NEATNFDLLLQAVRD----ELDSYASQN--A-PGHHFLLSIAAPA----GPEKYKKLH--LDKISNIVDQINIMAYDYSG-S-WDSASGHNANLFPYKA--STN-----------PYNSDKAINDYIDA-GVPAEKIVLGMPIYGRSFEGN----------------------------------LGIGKSFSDV----------G-QGSWE----------------------------R-------GVWDYKAL---------------------------------------------------------------------------------PKPGAEI-KYDEE----AQAYYSY--DSIMHELISYDTPEEVEKKVD-YVLKHGLGGSMFWEASGDKK----G------NESLIGTSYNCL---------G---TLDESENWLSF-----PDSRYANIAL---------GMPGQWAQ--------------------------------------------------------------------------------------------------------------------------------------------------------------------------------------------------------------------------------------------------------------------------------------------------------------------------------------------------------------------------------------------------------------------------------------------------------------------------------------------------------------------------------------------------------------V**

**>EAA74223_G_zeae_A_V**

**M--------------------------------------------------------------------------------------------------------------------------------------------------------------------------------------------------------------------------------------------------------------------------------------------------------------------------------------------------------------------------------------------------------------------------------------------------------------------------------------------------------------------------------------------------------------------------------------------------------------------------------------------------------------------------------------GGGPEGFRTVAYFVN--WAIY--A-RKHRPQDLP--VENLTHILYSFANIRSD-SGE-VHLTDS-WADTDIHWDG--------D--------SWNDVGT-NLY--GCMKQLNLLKRRN-RNLKVLLSIGGWT-FSSN----FKGPASTPQGRATFAKSCVDLIKNLGFDGIDIDWEYPQ---------NA--------------DEARNYVELLGAVRY----EMDAYAQTL--S-RPYHFELTVACPA----GATNFQKLD--IRGMDRYLDFWNLMAYDYAG-S-WDQTAGHQANLYPSHDNPVST-----------PFSTSAAIDFYVRS-GVSPSKIVLGMPLYGRAFQNT----------------------------------DGPGRPYQGV----------G-EGTWE----------------------------Q-------GVYDYKAL---------------------------------------------------------------------------------PLEGAQE-YGDRG----CCASYCY--NPQTRTMVTYDTPRVAWDKAE-YVRKWKLGGAMWWESSADKE----G------EKSLITTVVNGF---------GGQGALMRQDNCIEY-----PATKYDNLRD---------GFPNN------------------------------------------------------------------------------------------------------------------------------------------------------------------------------------------------------------------------------------------------------------------------------------------------------------------------------------------------------------------------------------------------------------------------------------------------------------------------------------------------------------------------------------------------------------------**

**>MGG_05533_M_grisea_A_V**

**M---------------------------------------RPTTLVLPLA----------------------VLA--------------------------------------------------------------------------------------------------------------------------------------------------------------------------------------------------------------------------GSVTARLT-------------------------RP-----------------------------------------------------------------------------------------------------------------------------------------------------------------------------------------------EGRGL--------------------------------------------------------------------------------------QSREAPQDITP------------------------------------------NRDSSAYRSVAYVTN--WSIY--G-AKFLPEQIA--VDSISHVQYAFADILAN--GT-VVSSDA-WADTQKQFGN--------G--------TQGGNSS-DAH--GLVEQLYKIKQAN-RNVKMLLSIGGYT-WSPK----FVPVAADAGKRAAFVSSAVALMGDWGMDGLDVDWEYPD---------TA--------------ETNKNCVLLLQELRR----GLDDYSQQH--TGSQYKFSLSMPAPA----GPTHYAAFD--FKAMDASLDFWSIMAFDFAG-S-WDNTTGHQANVYPGPD--LTT-----------KASIDVAVSDYVKA-GVAPGKINLGLPLYGRSFDKT----------------------------------TGLGKGYTAS----------T-AGSLEG--------------------------QA-------GIWIYKDL---------------------------------------------------------------------------------PRAGATV-LYDDV----AKASYTM--DNSTGQLISYDDLKSVQFKSD-YVKQKQIGGVMFWESSQDRP----G------SDSIVRTMARSL---------G---KLESAQNWLSY-----PGSQYANIKN---------NMGQAV-----------------------------------------------------------------------------------------------------------------------------------------------------------------------------------------------------------------------------------------------------------------------------------------------------------------------------------------------------------------------------------------------------------------------------------------------------------------------------------------------------------------------------------------------------------------**

**>MGG_01247_M_grisea_A_V**

**------------------------------------------------------------------------MIC-----------------------------------------------------------------------------------------------------------------------------------------------FDAPTRT------------------------------------------VTVTVDGNGQPQNTPAPVDPV-----THIASIIIPSGTSPAS-----------------SPAGTAKA-----------------------------------------------------------------------------------------------------------------------------------------------------------------------------------QSSQTVIATPS--------------------------------------------------------------------------------------QTSGASNPSPS------------------------------------------AANWSGYRSVLYFTN--WGIY--G-ANYQPQDLP--VDTVTHILYSFANIATD--GE-VISSDT-YSDIDKHYPT--------D--------SWNDLGR-NAY--GCVKQLYLLKKKN-RHLKVLLSIGGWT-WSPK----FAPIAATAAGRNRFATSAVKLLADWGLDGLDIDWEYPT---------NK--------------NEADNYVLLLKACRE----ALDAYSAKN--A-QGYKFLLTVATPA----GPENYGNMN--LEGMDKFVDFWNLMAYDYAG-S-WDTTTGHQSNLYLDDQNKVAT-----------KFSTEKAVQDYFAR-GIDAAKITLGLPLYGRSFAST----------------------------------GGLGKPFSGL----------G-DGSIE----------------------------R-------GVWLYKDL---------------------------------------------------------------------------------PRPGAIV-AYDNV----AKASYSY--DAAKREFVTYDTVDSAREKTR-YMKQKGLGGAVFWEASGDRK----G------DQSLVGTVARGV---------G-LDVLDKSTNLLAY-----PVSQYDNIRN---------GMPGV------------------------------------------------------------------------------------------------------------------------------------------------------------------------------------------------------------------------------------------------------------------------------------------------------------------------------------------------------------------------------------------------------------------------------------------------------------------------------------------------------------------------------------------------------------------**

**>MGG_00086_M_grisea_A_V**

**M---------------------------------------MLRLAAAALG----------------------LL----------------------------------------------------------------------------------------------------------------------------------------------------------------------------------------------------------------------------SLVAPPL----------------------------------------------------------------------------------------------------------------------------------------------------------------------------------------------------------------------------------------------------------------------------------------------------------------------AGASPISSEL------------------------------------------VRRQSGLQNVAYFVN--WGIY--G-RNFHVQNVS--ADKVTHLLYAFANLRDD--GT-VVGGDI-EADVSKHYPT--------D--------SWNDVGN-NAY--GCVKQLYKLKKAN-RHVKVLLSIGGWT-WSTN----FPSAASSEANRNRFASTAVGLMKDWGFDGLDIDWEYPA---------DA--------------TQGQNMVLLLQAVRD----ALDAYAAQH--A-PGYHFLLTIASPA----GPTHYQKMQ--LGQLAGVVDKMYLMAYDYAG-A-WDATSGHQANLFPSGSNPTST-----------PFSTDRAVADYMAA-GVPPSKIVLGMPIYGRTFQNT----------------------------------DGPGGAYSGT----------G-PGSWE----------------------------N-------GIWDYKAL---------------------------------------------------------------------------------PRPGATE-LYDAA----TGATWSY--DAARREMVTYDTADNVRRKVD-WARARGLGGSMFWELSGDRS----D------GASLVGTSAASL---------G---ALDTSQNQLSY-----PDSQYLNIRQ---------GLPGE------------------------------------------------------------------------------------------------------------------------------------------------------------------------------------------------------------------------------------------------------------------------------------------------------------------------------------------------------------------------------------------------------------------------------------------------------------------------------------------------------------------------------------------------------------------**

**>MGG_07927_M_grisea_A_V**

**-------------------------------------------------------------------------------------------------------------------------------------------------------------------------------------------------------------------------------------------------------------------------------------------------------------------------------------------------------------------------------------------------------------------------------------------------------------------------------------------------------------------------------------------------------------------------------------------------------------------------------------------------------------------------------------------MTQRTVAYFVN--WAIY--G-RKHRPQELP--ADKLTHVLYAFANVKPD-SGE-VFLTDT-WADTDIHWEG--------D--------SWNDEGT-NLY--GCLKQLNLLKKAN-RNLKVLLSIGGWT-YSSN----FRAPASTPEGRSHFARTAVELVRQLGFDGIDIDWEYPQ---------NP--------------QEAADLVALLQACRH----ELDAYAHSVDGE-NPYHFELTVACPA----GASNYEKMD--IAGMDPLLDFWNLMAYDYAG-S-WDQTAGHQANLFPSHGNPTST-----------PFSTDAAIAHYASQ-GVALDKIVMGMPIYGRAFENT----------------------------------DGPGAPFQGV----------G-QGTWE----------------------------Q-------GVYDFKAL---------------------------------------------------------------------------------PLPGAEE-RFDEE----AGATYSY--DPATRHMVTYDTVPLARRKAE-FIRERGLGGAMWWESSADKE----G------DESLIGNVVDVF---------GGPEGLKHQENCLAY-----PQTKYDNLRN---------GFPNH------------------------------------------------------------------------------------------------------------------------------------------------------------------------------------------------------------------------------------------------------------------------------------------------------------------------------------------------------------------------------------------------------------------------------------------------------------------------------------------------------------------------------------------------------------------**

**>Chi18-6_H_jecorina_A_V**

**M----------------------------------------FRLLCIGLP----------------------LLARV--------------------------------------------------------------------------------------------------------------------------------------------AVAVPATLSE----------------------------------------------------RCDAPNPPA-----SSLTESVAAISSGPLPFD----TEMGDVETVHREPFDAVPV------------------------------------------------------------------------------------------------------------------------------------------------------------------------------------GPEPASSGVS--------------------------------------------------------------------------------------IEHGHGSEVVG------------------------------------------FGPTSRKKNVVYYAD--WSIY--D-AAFLPQQLP--AEDITHLLYAFAGIEDD--GS-VVSMDP-WADEEKMLSVPGG-PGRND----------TQGLN-DVH--GAVEQVFLLKKRH-RHMKTLLSIGGWN-MSQS--GKFAPVLNTNEGRIRFAKTAVNLLANWGLDGIDIDYEYPI---------NE--------------REAVGFVDLLRECRK----ALDEYASLH--N-QRYHYLLTAAVSA----AQQHYKWLD--MPAIDRYLDAWHLMAYDYAG-S-WDDLSGDQSNVFHDATNPHRT-----------RANSDQAVNDYVAA-GVNPEKIILGIPLYGRSFMNT----------------------------------DGPGKPYEGV----------G-KGSIE----------------------------K-------GVWLYRDL---------------------------------------------------------------------------------PRPGSVV-NLNRD----IIAAYSY--DNATRELVTYDNMETTTLKAE-YLGKKGLGGAVFWEASGDRS----G------DESLIKAVARKM---------A---LLDISMNMLSY-----PQSQFSNIRN---------ATR--------------------------------------------------------------------------------------------------------------------------------------------------------------------------------------------------------------------------------------------------------------------------------------------------------------------------------------------------------------------------------------------------W-----------------------------------------------------------------------------------------------------------------------------------------------------------------**

**>Chi18-5_H_jecorina_A_V**

**----------------------------------------MLNFFGKSVA----------------------LL----------------------------------------------------------------------------------------------------------------------------------------------------------------------------------------------------------------------------AALQATLTSA-------------------------------------------------------------------------------------------------------------------------------------------------------------------------------------------------------------------------------------------------------------------------------------------------------------------SPLATKEHSV------------------------------------------EKRASGFANAVYFTN--WGIY--G-RNFQPADLV--ASDITHVIYSFMNLQSD--GT-VVASDT-YADVEKHYAD--------D--------SWNDVGT-NLY--GCAKQLFKLKKAN-RNLKVMLSIGGWT-YSTN----FASAASTDANRKRFASTAITYMKDWGFDGIDIDWEYPA---------DS--------------TQASNMILLLKEVRS----QLDAYAAQH--A-PGYHFLLSIAAPA----GEVNYSLLR--MADLGQVLDYVNLMXYDYAG-S-WSNASGHDANLYHNPQNPNAT-----------PFNTDDAVKAYING-GVPASKIVLGMPIYGRSFEST----------------------------------SGIGQPFTGI----------G-SGSWE----------------------------N-------GVWDYKAL---------------------------------------------------------------------------------PKAGATV-QYDDV----AKASYSY--DPSTKELISFDTPDVVNTKVS-YLKSLGLGGSMFWEASADKK----G------ADSLIGTSHKAL---------G---ALDSTQNLLSY-----PDSKYDNLKN---------GLI--------------------------------------------------------------------------------------------------------------------------------------------------------------------------------------------------------------------------------------------------------------------------------------------------------------------------------------------------------------------------------------------------------------------------------------------------------------------------------------------------------------------------------------------------------------------**

**>Chi18-7_H_jecorina_A_V**

**----------------------------------------------------------------------------------------------------------------------------------------------------------------------------------------------------------------------------------------------------------------------------------------------------------------------------------------------------------------------------------------------------------------------------------------------------------------------------------------------------------------------------------------------------------------------------------------------------------------------------------------------------------------------------------------MASDGYRSVAYFVN--WAIY--A-RKHRPQDLP--VEKLTHVLYAFANVRQD-TGE-VHMTDG-WADTDIHWEG--------D--------SWNDTGN-NMY--GCLKQLNLLKKRN-RNLKVLLSIGGWT-YSGN----FKGPASTQQGRETFAKSSLELLKNMGFDGLDIDWEYPQ---------NA--------------DEARNFVELLATVRR----ELDAYSATL--P-NYSHFELTVACPA----GPTHFQKLD--VAGMDQYLDFWNLMAYDYAG-S-WDQTSGHQANLHPSYDNPAST-----------PFSTDAAIDYYTRS-GVAPSKIVLGMPIYGRAFENT----------------------------------DGPGKPYSGI----------G-EGSWE----------------------------N-------GIFDYKVL---------------------------------------------------------------------------------PLPGSQE-FWDEA----IGASYCY--NPQTRKLVSYDTPYAARAKAR-YVKEWGLGGGMWWESSADKE----G------HESLIGIVVNEF---------GGPGGLQRKENCVDY-----PQSKYDNLRN---------GFPNN------------------------------------------------------------------------------------------------------------------------------------------------------------------------------------------------------------------------------------------------------------------------------------------------------------------------------------------------------------------------------------------------------------------------------------------------------------------------------------------------------------------------------------------------------------------**

**>03209_N_crassa_A_V**

**M---------------------------------------LSSIVTCVIP----------------------LL------------------------------------------------------------------------------------------------------------------------------------------------------------------------------------------------------------------------------------------------------------------------------------------------------------------------------------------------------------------------------------------------------------------------------------------------------------VISTPA--------------------------------------------------------------------------------------AAATSQFQVQSSG----------------------------------------NTTVSSYRNAVYFTN--WGIY--G-ANFQPQQLP--ASQITHALT----------PS-RVSSDT-YADLEKRYPG--------D--------SWSEPGE-NAY--GCVKQMYLLKKRN-RNMKVLLSIGGWT-YSPK----FPPVAATEEGRRRFASSAVKLVQDWGFDGLDIDWEYPT---------NA--------------REAQDFVLLLRACRQ----ALDDYARQY--A-PGYHFLITIAAPA----GPQHYGVMD--LPGMNPYIDSWHLMAYDYAG-S-WDSTTGHQANLLPSPKNLLTT-----------RFNTDQAVRDFVRR-GIPANKIVLGLPLYGRSFEGT----------------------------------DGLGKPYSGI----------G-AGTLE----------------------------P-------GTWVYRDL---------------------------------------------------------------------------------PRPGAKE-EYDNL----AKATYSY--DALSRELITYDNVLSALVKTK-YIFLRGLGGAVFWEASGDKT----G------AESLIGTLATQM---------K---RLDQTKNLLAY-----PASRYANIRA---------GVPES------------------------------------------------------------------------------------------------------------------------------------------------------------------------------------------------------------------------------------------------------------------------------------------------------------------------------------------------------------------------------------------------------------------------------------------------------------------------------------------------------------------------------------------------------------------**

**>04883_N_crassa_A_V**

**----------------------------------------MFSIKAAALA----------------------VL----------------------------------------------------------------------------------------------------------------------------------------------------------------------------------------------------------------------------SFSSFLVDAAPAPM------------------CPG----------------------------------------------------------------------------------------------------------------------------------------------------------------------------------------------TNVTS--------------------------------------------------------------------------------------TSADTNVTVPD------------------------------------------RADSSGYKNVVYFTN--WGIY--G-RNYQAADLP--ADKITHVLYSFANLKED--GT-VFSSDT-WSDTDKRYPT--------D--------SWNDNGT-NVY--GCVKQLYLLKKAN-RNVRVLLSIGGWT-YSQTSPSRFALTASTAESRTKFATSALALVKDWGFDGIDIDWEYPA---------SE--------------TEAQNFLLLLKEIRS----QMDKYAAAH--A-DGYHFLLTMAASA----GPSKYGVLESSMKEIGETLDFMNLMAYDYAG-A-WDKKAGHQANLYPDEKNPDTT-----------PFSTDRAVTDYIKF-GIPSNKIVLGMPLYGRAFAST----------------------------------DGPGTAYSGV----------G-EGSWE----------------------------K-------GIWDYKVL---------------------------------------------------------------------------------PKSGAKV-FLDEK----VGASWSY--DETNKVMVSYDTPEMVKQKVS-YIKEKGLGGAMYWEASGDRT----D------KDSLMTVVKDGL---------G---TLDSEKNLLEY-----PDSQYDNMKK---------GMSS-------------------------------------------------------------------------------------------------------------------------------------------------------------------------------------------------------------------------------------------------------------------------------------------------------------------------------------------------------------------------------------------------------------------------------------------------------------------------------------------------------------------------------------------------------------------**

**>04554_N_crassa_A_V**

**MTASRHILYATALN-QTTKTRRIRIAKQSHEKTPINGQTPPPRAFSPSLTSPFVHLSPESQVEQSISSAPSRLLRRIEVHL--------------------------------------------------------------------------------------------------------------------------------------HRILSVPLGPAFCNPRNPKGCAIVCHPH-Q-------------------------LYSGHQHPTATIATTATVT--FTESVTATITSVDKGPTPSSV---T--------STTPAT----------------------------------------------------------------------------------------------------------------------------------------------------------------------------------------TETSTTTTEQ--------------------------------------------------------------------------------------QTTPTSPTMGG------------------------------------------GPDTTGYRTVAYFVN--WAIY--G-RKHRPQDLP--AEKLTHILYAFANVRPD-SGE-VYLTDQ-WADTDIHWEG--------D--------SWNDSGT-NLY--GCLKQLNLLKRRN-RNLKVLLSIGGWT-YSSN----FKEPASKEEGRRRFAESAVELVKNLGFDGLDIDWEYPQ---------NA--------------QEASDFVSLLSACRS----ALDAYASTV--P-GNPHFEMSVACPA----GPQNYEKMD--LPGMDRYLDFWNLMAYDYAG-S-WDQVAGHQANLFHCNSNPAAT-----------PFSTQRAVEYYLST-GVAADKIVLGMPLYGRAFQGT----------------------------------EGLGQPYSGV----------G-EGTWE----------------------------N-------GVHDYKKL---------------------------------------------------------------------------------PLEGAEE-RVDYE----SGATYCY--HPGTKTLVSYDTPGMARMKAE-YIRERGLGGAMWWESSADKE----G------PDSLIGTVVEGL---------GGLPALRWDGNCIDY-----PNTKYDNLRA---------GFPGN------------------------------------------------------------------------------------------------------------------------------------------------------------------------------------------------------------------------------------------------------------------------------------------------------------------------------------------------------------------------------------------------------------------------------------------------------------------------------------------------------------------------------------------------------------------**

**>05350_B_fuckeliana_A_V**

**M---------------------------------------------------------------------------------------------------------------------------------------------------------------------------------------------------------------------------------------------------------------------------------------------------------------------------------------------------------------------------------------------------------------------------------------------------------------------------------------------------------------------------------------------------------------------------------------------------------------------------------------------------------------------------------------GGGHGYRAVAYFVN--WAIY--G-RKHFPQELP--AEKLTHILYAFANVRPD-SGE-VYMTDG-WADTDIHFEG--------D--------SWNDVGT-NLY--GCLKQLNILKSRN-RSLKILLSIGGWT-YSSN----FAQPASTESGRRKFAESAVDLIRNLGFDGIDIDWEYPK---------NA--------------EEARDYVLLLRECRE----AMDRYSNTL--P-TPHHFELTVACPA----GPQNYNNMD--IYGMNQYLDFWNLMAYDYAG-S-WDGVAGHQANVLASHNNPSST-----------PYNTEHAVRAYEAK-GVRSHKIVLGMPIYGRAFQGT----------------------------------EGLGRPFNGV----------G-EGTWE----------------------------N-------GVHDFKKL---------------------------------------------------------------------------------PQEGARE-FVDEE----TCASYSY--NENTKTLISYDTVEVGRRKAH-YIKQRGLGGAMVCFVPNPLP----------------------------------------------------------------------------------------------------------------------------------------------------------------------------------------------------------------------------------------------------------------------------------------------------------------------------------------------------------------------------------------------------------------------------------------------------------------------------------------------------------------------------------------------------------------------------------------------------------------------------------------------**

**>11407_B_fuckeliana_A_V**

**M---------------------------------------ILTFMQYFLL----------------------IL----------------------------------------------------------------------------------------------------------------------------------------------------------------------------------------------------------------------------TNLNSLVFSAAIP-----------------------------------------------------------------------------------------------------------------------------------------------------------------------------------------------------------------------------------------------------------------------------------------------------------------AKNDKSVSA------------------------------------------VGYTGGYHNAAYFVN--WAIY--G-RNYQPQQLP--ATKLTHVLYAFANLQTD--GT-VYLSDT-YSDLQKHYPT--------D--------SWNDVGN-NVY--GCIKQLYLLKKNN-RQMKTLLSIGGWT-YSTN----FAAAASTNATRTKFAATAVQFVQDLGFDGIDIDWEYPA---------ND--------------VEAGNFVLLLQAVRS----ALDAYAAQH--A-PGYHFLITVASPA----GPEKYNVLH--LADMDKYLDSWHLMAYDYAG-S-WDNVTGHMANLNPSVNNNSST-----------PYSTQRAITDYIAK-GVSVSKIIMGLPLYGRAFEST----------------------------------IGMGLTFNGI----------G-TGSWE----------------------------S-------GVWDYKDL---------------------------------------------------------------------------------PRAGATE-YYASD----VVGTYSY--DNSTKELVSYDNVKAIQTKTE-YIMSSGLGGAMFWESSGDRN----D------SKSLISTTAGIF---------P---SLDQTQNLLTY-----NASAYANLAA---------GMPGV------------------------------------------------------------------------------------------------------------------------------------------------------------------------------------------------------------------------------------------------------------------------------------------------------------------------------------------------------------------------------------------------------------------------------------------------------------------------------------------------------------------------------------------------------------------**

**>11304_S_sclerotiorum_A_V**

**M---------------------------------------------------------------------------------------------------------------------------------------------------------------------------------------------------------------------------------------------------------------------------------------------------------------------------------------------------------------------------------------------------------------------------------------------------------------------------------------------------------------------------------------------------------------------------------------------------------------------------------------------------------------------------------------GGGDGYRAVAYFVN--WAIY--G-RKHFPQELP--AEKLTHVLYAFANVRPE-SGE-VYMTDG-WADTDIHFEG--------D--------SWNDVGT-NLY--GCLKQLNILKSRN-RSLKILLSIGGWT-YSSN----FAQPASTESGRRRFAESAVDLIRNLGFDGIDIDWEYPK---------NT--------------DEARDYVLLLRECRE----AMDRYSSTL--S-NFHHFELTVACPA----GPQNYNNMD--VRSMNEYIDFWNLMAYDYAG-S-WDSVAGHQANLHPSESNPDST-----------PFNTEHAVRAYEAR-GVQGHKIVLGMPIYGRAFQGT----------------------------------EGLGKPFNGV----------G-EGTWE----------------------------N-------GVHDFKKL---------------------------------------------------------------------------------PQEGAEE-IVDEE----TCASYSY--NEDTKTLITYDTVEIARRKAY-YIKERGLGGAMWWESSADHP----ER-----EKSLIHNVVDVL---------G---ILEGSDNCLEY-----PESKYDNLRN---------GF---------------------------------------------------------------------------------------------------------------------------------------------------------------------------------------------------------------------------------------------------------------------------------------------------------------------------------------------------------------------------------------------------------------------------------------------------------------------------------------------------------------------------------------------------------------------**

**>11700_S_sclerotiorum_A_V**

**----------------------------------------MHLFMQYSVL----------------------AL----------------------------------------------------------------------------------------------------------------------------------------------------------------------------------------------------------------------------TCLSSFASASAVPHG------------------------------------------------------------------------------------------------------------------------------------------------------------------------------------------------------------------HYSKK--------------------------------------------------------------------------------------SGVKAAVGGTV------------------------------------------QTVSGGYHNTAYFVN--WAIY--G-RNYQPQQLP--ASKLTHTIYAFANLKTD--GT-VYLSDT-YSDLEKHYST--------D--------SWNDTGN-NVY--GCIKQLYLHKKHN-RQMKTLLSIGGWT-YSAN----FAAAASTEATRARFASTAVQFVQDLGFDGLDIDWEYPA---------ND--------------VEAANFVLLLKAIRS----ALDAYAARY--A-PGYHFLITAATPA----GPDHYNVLH--MADMDKYLDSWHLMAYDYSG-S-WDNTTAHNANLYPSVLNPKST-----------PFSTQRAIVDYVAK-GVPISKIILGLPINGKSFLNT----------------------------------TGLGMQATGI----------G-PGSWE----------------------------A-------GIYDYKVL---------------------------------------------------------------------------------PRAGATE-FYLPD----VVATYSY--DNVTKELISYDNVKSVSMKTK-YIMTSGLGGAMFWETSADKT----D------DGSLISTTARLF---------P---ALDQARNLLTY-----NASAYANMAA---------GMPGE------------------------------------------------------------------------------------------------------------------------------------------------------------------------------------------------------------------------------------------------------------------------------------------------------------------------------------------------------------------------------------------------------------------------------------------------------------------------------------------------------------------------------------------------------------------**

**>08020_S_sclerotiorum_A_V**

**MINSE-------------------------------------------------------------------IYKA---------------------------------------------------------------------------------------------------------------------------------------------AEHLPTQPQP------------------------------------------------------------------SRVYIIFSLSSPSPKACG---------------QP--------------------------------------------------------------------------------------------------------------------------------------------------------------------------------------------------------------------------------------------------------------------------------------------------------------------------------------------VQYIVSVVKSRAWKYRRR--LAIY--G-RQFFPQELP--VANLTHVLYAFANIDPE-SGA----------------------------------------------------QMYILKKQN-RNLKVLLSIGGWT-YSSN----FPAAASTISKRATFASSVVSLVQNLGLDGIDVDWEYPS---------DD--------------TEANDFVLLLQEVRD----ALDRFGDSL--Q-SPYHFLLTVASPA----SPAVCQNWK--LSEMDQYVDFWNFMAYDYSG-P-WSTVSAHQANLSPSGNG--ST-----------PFNTQTGISYYIAH-GITSAKIVLGMPIYGRSFEQT----------------------------------NGLDQSFQGV----------G-QGTWA----------------------------S-------GVYDYKAL---------------------------------------------------------------------------------PLAGATE-VYDKK----IGASYSW--DTSKREIISYDNPMVAIQKAQ-WIQSMNLGGAMWWESSADGQ----G------SRSLIGSVMSVL---------GG--HMQNVTNQLSF-----PNSTYANIRN---------GMPGFSKL--PLL---------NITSPKISTCYSTKAYTSSGS--DIPPSLLGITTTSSPMEVTTAVGSSQG-------------------------------TTTAGSSKKTTAVASSKKT----------------------------TAVASSQKTTAVASSQ---------------------------------------------------------------------------------------------------------KTTAVASSQGTTTVSDSSSVTNVCSLVSDCSIVTTLFSYSTSTPITLSGTTSTSFSISTDSATLCICNSCFFDVRTAVSGASTTTYC---GNTISVPAGFTKIINGKLPISTTTTT-----------------MLPIPTAETITAIT-----TTTILSPKRRSGCNPNLECYTSSTSIVGP----------------GYSPTGLICTTDTNNDPLENQSI-----QSG-----ALSGLKKPN-----------SFI---------MLDYTRS------QV**

**>05250_C_immitis_A_V**

**MT--------------------------------------LDALIAANPQIANPNIIEVGQ-----------VLNLSQS------------------------------------------------------------------------------------------------------------------------------------------PAEDPPNP--------------------------------------------------------------------APETSATTAVADTPGPVF----S--------ATAPAR---------------------------------------------------------------------------------------------------------------------------------------------------------------------------------------EMSTATATAES--------------------------------------------------------------------------------------RGPVFTAATPA------------------------------------------PSNPAGYKTVAYFTN--WGIY--G-RNYQPNNIP--ASHITHILYSFANIRGD-TGE-VHLTDT-YADLEKHYPS--------D--------SWDEQGN-NAY--GCIKQLFLLKKKN-RHLKILLSIGGWT-YSSN----FR-AAATHEGRQTFARSAVKLLADCGFDGIDIDWEYPQ---------ND--------------TEALNFVYLLYETRM----ALENYAAQY--A-QGRHFLLTIAAPC----GPKNYSQLR--MAEMDPYLDFWNLMCYDFAG-S-WDSTAGHMANVFPSQSSPEST-----------PFSADIAVSAYISA-GIHPGKIIFGLPLYGRGFENT----------------------------------QGPGSPFQGV----------G-EGSWE----------------------------N-------GVWDYKAL---------------------------------------------------------------------------------PQPDSEE-QNDDQ----LLASWSY--SPSAKKMISYDTPHMAQRKAE-YIRNRGLGGAMWWELSGDHP--VNH------ERSLINITIAGL---------GGTAGLDGSGNCLDY-----PASVYDNLKK---------QFE--------------------------------------------------------------------------------------------------------------------------------------------------------------------------------------------------------------------------------------------------------------------------------------------------------------------------------------------------------------------------------------------------------------------------------------------------------------------------------------------------------------------------------------------------------------------**

**>03822_C_immitis_A_V**

**MDGRQSSSYGDAVR-RANSNSSFRPSGWSRG---------IFSFFLISWV----------------------FL----------------------------------------------------------------------------------------------------------------------------------------------------------------------------------------------------------------------------GFSSFTVASSVATDSIR----Y--------RRSPFFLPPA------------------------------------------------------------------------------------------------------------------------------------------------------------------------------------------------------------------------------------------------------------------------------------PEARSVSAPGN------------------------------------------GENSSGYKSVVYYVN--WAIY--D-RQYNPQDMP--VDKITHVLYAFANIRPT-TGE-VYLSDE-QADIKKRFPT--------D--------SATQPGN-NVY--GCANQLYLLKKRN-RNLKVLLSIGGWT-YSEN----FAPAASTEAGRSMFAESAVKLMLDMGMDGLDVDWEYPK---------DD--------------KEAQDFAALLKAVRQ----KLNSVA-------GGRKFLLTIAASA----GAEHYEKLH--LKEMGQDLDFINIMAYDYAG-S-WSTMAGHQANLRPSMSNPQST-----------SFSTEAALDYYINTGGIPASKLVLGMPLYGRAFANT----------------------------------EGPGKPYQGN---------GG-AGSWE----------------------------P-------GIWDYKTL---------------------------------------------------------------------------------PQPGAKE-FMDPMVNGGAGASWSY--DPSKKLMISYDTVPMVEAKTK-YIVDKGLGGAMWWEASGDRG----GKDATKAGGSLIATFAEDVVK-------AGKMGLDKTQNALQY-----SESQYDNIKG--------------------------------------------------------------------------------------------------------------------------------------------------------------------------------------------------------------------------------------------------------------------------------------------------------------------------------------------------------------------------------------------------------------------------------------------------------------------------------------------------------------------------------------------------------------------------------**

**>02795_C_immitis_A_V**

**----------------------------------------MRFLIGALLT--------------------------------------------------------------------------------------------------------------------------------------------------------------------------------------------------------------------------------------------------------------------------------------------------------------------------------------------------------------------------------------------------------------------------------------------------------------------------------LQTLVQASSM--------------------------------------------------------------------------------------SSMPNSYPVPE------------------------------------------APAEGGFRSVVYFVN--WAIY--G-RGHNPQDLK--ADQFTHILYAFANIRPS--GE-VYLSDT-WADTDKHYPG--------D--------KWDEPGN-NVY--GCIKQMYLLKKNN-RNLKTLLSIGGWT-YSPN----FKTPASTEEGRKKFADTSLKLMKDLGFDGIDIDWEYPE---------DE--------------KQANDFVLLLKACRE----ALDAYSAKH--P-NGKKFLLTIASPA----GPQNYNKLK--LAEMDKYLDFWNLMAYDFSG-S-WDKVSGHMSNVFPSTTKPEST-----------PFSSDKAVKDYIKA-GVPANKIVLGMPLYGRAFAST----------------------------------DGIGTSFNGV----------G-GGSWE----------------------------N-------GVWDYKDM---------------------------------------------------------------------------------PQQGAQV-TELED----IAASYSY--DKNKRYLISYDTVKIAGKKAE-YITKNGMGGGMWWESSSDKT----G------NESLVGTVVNGL---------GGTGKLEQRENELSY-----PESVYDNLKN---------GMPS-------------------------------------------------------------------------------------------------------------------------------------------------------------------------------------------------------------------------------------------------------------------------------------------------------------------------------------------------------------------------------------------------------------------------------------------------------------------------------------------------------------------------------------------------------------------**

**>AN5454_E_nidulans_A_V**

**MK--------------------------------------LPILFFSTIF---------------------LVLA----------------------------------------------------------------------------------------------------------------------------------------------------------------------------------------------------------------------------------QAATLP--------------------PLY-------------------------------------------------------------------------------------------------------------------HIYRSLNSTSN------------------------------------------------------------NNTNYAHN--------------------------------------------------------------------------------------NTTGRGPGYSPGPISD------------------------------------PSSGQREYRSMAYFVN--WAIY--A-RHHNPQDLP--VSHLTHILYAFANVRPE-TGE-VYLSDT-YSDIEKHYPT--------D--------SWNDQGNDNVY--GCVKQLFLLKQQN-RHLKVLLSVGGWT-YSKN----FARMAATEEGRRLFAKSAVELMGDLGMDGVDVDWEYPE---------ND--------------DQASDFVELLRETRE----ELDRYAADN--D-SDKPFLLSVASPAEP--GPSKYTTLH--LSSMDRYLDFWNLMAYDYSG-S-WDTITGHNANLFASRQNPAST-----------PFNTHTAVTAYLAA-GIPASKINLGMPLYGRSFTHT----------------------------------DGPGTEFEGV----------GYEGSFE----------------------------A-------GVWDYKAL---------------------------------------------------------------------------------PKPGAEV-VEVEE----IAASFSY--DEDKKEVISYDNVGVVRHKGV-YLMRLGMGGGMWWESSGDRE--VGS------GGSLIETLVDTL---------GGVNALDKSENQLEY-----PASRFENLRN---------GFT--------------------------------------------------------------------------------------------------------------------------------------------------------------------------------------------------------------------------------------------------------------------------------------------------------------------------------------------------------------------------------------------------------------------------------------------------------------------------------------------------------------------------------------------------------------------**

**>AN4871_E_nidulans_A_V**

**------------------------------------------------------------------------------------------------------------------------------------------------------------------------------------------------------------------------------------------------------------------------------------------------------------------------------------------------------------------------------------------------------------------------------------------------------------------------------------------------------------------------------------------------------------------------------------------------------------------------------------------------------------------------------------------MSGYKTVGYFVN--WAIY--G-RNYNPQDLP--AEKLTHILYAFANVRPE-TGE-VYLSDT-WSDIEKHYPT--------D--------SWNDTGN-NVY--GCVKQLGLLKRQH-RQLKVLLSIGGWT-YSPN----FTNGAGTPENRARFAQTATKLITDLGFDGIDIDWEYPQ---------ND--------------QQAQNYVDLLRRCRE----ALNAAQGQ-------RRFQLTVAVPA----GPDNYNKLR--LQEMTPYLDFYNLMAYDYAG-S-WDQTAGHQANLYPSTSNPTST-----------PFNTVQAVNHYIDAGGVPSNKIILGMPIYGRAFQNT----------------------------------DGPGRPYSGI----------G-QGTWE----------------------------Q-------GVYDYKAL---------------------------------------------------------------------------------PRPGATE-QLDTN----IGASWSY--DPSSREMVSYDTVAAADLKAA-YIQSRRLGGAMWWETSADKG----GKTANKADGSLIGTFVEDV---------GGVNNLDRTQNAISY-----PDSQYDNLKA---------GFPSS------------------------------------------------------------------------------------------------------------------------------------------------------------------------------------------------------------------------------------------------------------------------------------------------------------------------------------------------------------------------------------------------------------------------------------------------------------------------------------------------------------------------------------------------------------------**

**>00269_P_nodorum_A_V**

**MT-------------------------------------------------------------------------------------------------------------------------------------------------------------------------------------------------------------------------------------------------------------------------------------------------------------------------------------------------------------------------------------------------------------------------------------------------------------------------------------------------------------------------------------------------------------------------------------------------------------------------------------------------------------------------------------SAAASGYKNVAYFVN--WAIY--G-RNFNPQDLP--GQELTHVLYAFANVRPE-TGE-VYLTDT-WSDTDKHYST--------D--------SWNDVGT-NVY--GCAKQLFLQKKKN-RKLKVLLSIGGWT-YSAN----FAQPASTEAGRAKFASSAVALLQDLGFDGLDIDWEYPA---------NE--------------AQANDMVSLLAATRK----ALDAYSAAH--A-PNQHLLLTVASPA----GAKNYNIMK--LGAMDKYLDFWNLMAYDYAG-S-WDANAGHQANFNPSGNNAIST-----------PFNTQQALKDYAAA-GVPLNKIVLGMPLYGRAFANT----------------------------------DGPGKPFSGV----------G-QGTWE----------------------------A-------GVYDYKTL---------------------------------------------------------------------------------PQAGASV-TNDLD----LGASWSY--DQGQRLMISYDTPQVIQKKSE-LVKSMGLGGGMWWESSSDKK----G------SDSLISTFVNAV---------GGTGALDQSQNLLSF-----PSSKYDNLKA---------GFPNE------------------------------------------------------------------------------------------------------------------------------------------------------------------------------------------------------------------------------------------------------------------------------------------------------------------------------------------------------------------------------------------------------------------------------------------------------------------------------------------------------------------------------------------------------------------**

**>14474_P_nodorum_A_V**

**MRF--------------------------------------------------------------------------------------------------------------------------------------------------------------------------------------------------------------------------------------------------------------------------------------------------SSATTLFSLASIAQA------------------AP------------------------------------------------------------------------------------------------------------------------------------------------------------------------------------------------------------------------------------------------------------------------------------------KVHPRDHVIAA------------------------------------------RETGQQYQSQVFYTN--WAMY--G-RKHFVTDLP--ADHLTKINYGFANVNNK-TGE-VFLSDE-WSDVQFPYPG--------D--------VATN-GS-QLL--GNFNQLYKLKQKN-RNLKVILSVGGWT-WRGN----FKPALATEAGRQKFCDSSLQLIADLGLDGFDIDWEYAE---------DA--------------TDAANFLDTVMRCRKLTNQAYDEYAAAN--A-HGYHFDLGISAPA----GPSRYSVLR--MKEMDQYVDNWNLMAFDYQGGG-FSNFTGHQSNVYASKTNPKTTDGWDSVLGKFMPFNTKEAIDYYKAN-VASPCKIVLGMPLYGRSFGNVVDLSK---------------------------DQRGLGQKFNGS----------G-EGTWE----------------------------P-------GTLDYKFL---------------------------------------------------------------------------------PLNGSKV-YQDKE----TLSSWSW--DPVKKEVVSFDTPKTAVWKTD-FLKSEGLGGAWWWDSSSDLP--ITN------DKSIISTVVKQL---------GGSTGLKQAKNNLYY-----PKSKYYNIAG---------AANSTVMQ---------------------------------------------------------------------------------------------------------------------------------------------------------------------------------------------------------------------------------------------------------------------------------------------------------------------------------------------------------------------------------------------------------------------------------------------------------------------------------------------------------------------------------------------------------------**

**>13635_R_oryzae_A_V**

**M-------------------------------------------------------------------------------------------------------------------------------------------------------------------------------------------------------------------------------------------------------------------------------------------------------------------------------------EP-----------------------------------------------------------------------------------------------------------------------------------------------------------------------------------------------------------------------------------------------------------------------------------------------------------------------------------------------PPATNGPVVAGYFAN--WGIY--D-RKYNVVDLALQADKLTHILYAFANLQPD--GQ-VVLGDT-YADLEKHFPANQTISGKED--------GWNDAGK-NLY--GNFKQFYLLKQRH-RHLKVSLSIGGWT-WSTH----FGAVTRDPLKRQRFVNSSMKLLTDLALDGLDIDWEYPK---------DD--------------EEAFYYVHLLYELRL----AMDKYQQQCG-QLNQPRLLLTVAVPC----GPDHYRKLR--LREMQPYVDLFYLMAYDYAG-S-WDRVAGHQAAVFGG------------------PLHTDQAVSHYLAA-GIPAHKLVLGLPVYGRGFSNT----------------------------------AGPEQAFQGV----------P-KGTWE----------------------------E-------GQFDYKCL---------------------------------------------------------------------------------PKPGATE-YHDFQ----RLASWSY--DPNARELITYDTPQIVSAKCS-YLLQRKLGGAMFWELSADCG--HQH------PRSLVNAVYDGL---------GR--RLDPTPNHLEY-----PMSEYDNIRN---------GMK--------------------------------------------------------------------------------------------------------------------------------------------------------------------------------------------------------------------------------------------------------------------------------------------------------------------------------------------------------------------------------------------------------------------------------------------------------------------------------------------------------------------------------------------------------------------**

**>UM04261_U_maydis_A_V**

**MQ--------------------------------------QLSCFITGLK---------------------HRMSRARLD-------------------------------------------------------------DDGKK-------------------------------------------------------------------SSSSASSLPPSPTK-----------------------------------------AATASAAGSVPQTPTATAPEAS-TPSSSTQPESPVASAPSST----------------SPPSTTPT-----------------------------------------------TP---------------------------------------------------------------------------------------------------------------------------------ASNTTPASEIQN--------------------------------------------------------------------------------------NIDSQGHDFTT------------------------------------------NGAVVPRVNLAYFTN--WGIY--G-RKYSPLDVP--YCNLTHVLYAFADVNPD-TGE-CFLTDL-WADEQIHYTG--------D--------SWNDTGN-NLY--GNFKQFLLLKKKN-RALKLMLSVGGWT-FGPH----FAPMAADAKKRAKFVSTAITILENDGLDGIDIDWEYPS---------DS--------------TQAANFVLLLKELRA----GLTAHQAKK--N-ETNPYLLSIAAPC----GPDHYKVLQ--VAKMDQYLDFWNLMAYDFAG-S-WSALTGHQANLW----NIKGA-----------PPSADDSINYYIGQ-GVVSHKLVLGIPLYGRGFENT----------------------------------DGPQQPYRGT----------G-QGTWE----------------------------A-------GNWDYKFL---------------------------------------------------------------------------------PVKGAKE-MINTK----IAASWSY--DSAKREFISYDTPQNVLLKCQ-YIRNKRLRGAMFWELSGDAT-KSQG----GAERSLIALTAKNM---------G---TLDATLNHISY-----PFSKWDNVKN---------GLK--------------------------------------------------------------------------------------------------------------------------------------------------------------------------------------------------------------------------------------------------------------------------------------------------------------------------------------------------------------------------------------------------------------------------------------------------------------------------------------------------------------------------------------------------------------------**

**>01315_C_cinereus_A_III**

**MVSFRLS-----------------------------------------------------------------LLGP----------------------------------------------------W---------------------------------------------------------------------------------------------------------------------------------------------------------------------CLVALLSTLVV----------------------------------------------------------------------------------------------------------------------------------------------------------------------------------------------------------------------------------------------------------------------------------------------------------------------ASPAAV------------------------------------------EGKTRGKVAAAWYAG--WHAT----TGYPLSRVS--WKKYTHLTYAFAETSPD--VHKLSFEEATW----------------------------------TNP--QLLPQFVSEAHKH--GVKALISIGGWT-GSRF----FSTAVGSASNRTAFVKTVVNFAQKYHLDGVDFDWEYPG---------NQ-GIGCNTINP----QDTSNFLLFLQELRR----DP-----------VGSKLLVTAAAATVPFYGSNGRPSSD--VAAFAKLLDYIAIMNYDLWG-P-WSPTVGPNAPLDDACAPANSR-----------YGSATSAVRQWSAA-GIPHNQLVLGVAAYGHGFRVRRANAF------------AAGSN----------SVLASYPPFDGVD--------RP-IGDSWD--GPGGGTDV-C--------G-SPTLPG-------GTFNFWGLIENG--------------------------------------------------------FLNK--D------------GSPTQGIHY-RYDTC----SQTPYVY--NRTTEIMVAFDNAQSFAAKGN-FIKSNGLRGFATWEAGGDY------------NDILLDSIRKAG---------GY------------------------------------------------------------------------------------------------------------------------------------------------------------------------------------------------------------------------------------------------------------------------------------------------------------------------------------------------------------------------------------------------------------------------------------------------------------------------------------------------------------------------------------------------------------------------------------------------------------**

**>03252_C_cinereus_A_III**

**MLTFA-------------------------------------------------------------------QLPL--------------------------------------------------------------------------------------------------------------------------------------------------------------------------------------------------------------------------VLLSLTLSVVAVH--------------------------------------------------------------------------------------------------------------------------------------------------------------------------------------------------------------------------------------------------------------------------------------------------------------------DEQLVR------------------------------------------RAPTDDLIVAAWYTG--WHS-----RDFPLSRVS--WSKYTHMTYAFGITTPS--PD---------------------------------------------------------------NVTASLSIGGWT-GSRF----FSPAVGSASNRTTFVNTLLDVVRRYNLDGLDFDWEYPN---------MQ-GLGCNIVHE----DDTANFLTFLRELRN----TT-----------TGRDLVLSAAVYVKPFNDASGSPSTD--VSGFAEVLDYIAVMNYDVPV-N-PSVGAGSRSPLDDTCAPAGAK-----------WGSAVSSIEEWTKA-GIPLEKLVLGVPAHGHSYDVSPVVAL------------SRSNS----------SQLNAYPSYSPAD--------RK-RGDRWD--GE-GGMDV-C--------G-VTWGPG-------GVYTYWGLFEGG--------------------------------------------------------FLNA--D------------GSVAEGIVS-RYDDC----SETPFVY--NPETQVYVSYENPRSYAAKGD-FIYSTGLGGFAMWEAGGDR------------NDTLLDSIIHASLN-------GNPSARTAKSKVDTQAVVPTPGSRETTANDL------SGASPSVW----------------------------------------------------------------------------------------------------------------------------------------------------------------------------------------------------------------------------------------------------------------------------------------NATPNHILGSALA-F-----IFNL---------------------------------------------------------------LLSS-----------AW-----------------------------------------------------------------------------------------------------------------------------------------------------------------**

**>05285_C_cinereus_A_III**

**MRAQ----------------------------------------SI--------------------------LL----------------------------------------------------------------------------------------------------------------------------------------------------------------------------------------------------------------------------SLLAASTSVWAAP-----------------------------------------------------------------------------------------------------------------------------------------------------------------------------------------------AVFG--RHEGHDHGSTDVAPQDADG---------------------------------------------------------------------------------------------------------------------------------------------TVVIPVTASGWYPS--WYK-----NVMPPSEIP--WEKYTELTFAFALTTPD--TS-ALTLEG-------------------------------------EE--ETLRAFVQEAKAN--NVKALLSIGGWT-GSQY----FSPAVASAENRTTFVKTITDMATEFGLDGIDFDWEYPN---------AE-GIGCNLRTP----EDTENFLTFLRELRE----HP-----------VGVNLKLTAAAHITPLMDATGTPGSD--MSGFAEVLDHLAIMAYDIHG-N-WLDQVGANAPLRGSCPA--SP-----------LGSVEGSVEAWTAA-GFPAEKLLLGLPAYARSFTVAKDDVL------------DADGN-----------IVTFNPAFDKES--------YP-LGEGEVE-GQ-VLTDK-C--------G-RPEFPA-------GLFTFNGLIAGG--------------------------------------------------------LLGT--D------------GEAAPGVVY-GFDNC----TETPFLY--RESDSRMVSFDDARSFAAKGR-FINEKGLAGFAVWHISGDTR-----------NNILLDSIYEAM---------GIEVQDDCGSEE---V-GDDAGAEEGEEEGE------CPEVPPVP----------------------------------------------------------------------------------------------------------------------------------------------------------------------------------------------------------------------------------------------------------------------------------------ENAPLPSVSSSA-------------------------------------------------------------------------------------------------------------------------------------------------------------------------------------------------------------------------------------------------------------**

**>05291_C_cinereus_A_III**

**MQFKTSFF---------------------------------------------------------------ALLAG--------------------------------------------------------------------------------------------------------------------------------------------------------------------------------------------------------------------------FLASSTLAAYVPVAD----------------SVP-----------------------------DN------------------------------------------------------------------------------------------------------------------------------------------DSLTA--PDCTHDGSTTTTAPAGDAVVPVP------------------------------------------------------------------------------AEPDSEVRLNATQVLA------------------------------------------SLDAGGKVATAWYPS--WQA-----AAHPPESLS--WDKYNAMTFAFATTTSD-PANPLALDAE-------------------------------------SQ--ALLPKFVEQAKQH--NVKALLSLGGWT-GSIY----FSDHVSTPERRTAFVKAVVDLATQYNLDGIDFDWEFPN---------KQ-GIGCNHISN----ADSANFLAFLQELRQ----DP-----------TGGKLMLTAAVGLLPFVGSDGQPMSD--VSGFAEVFDFIAIMAYDVWG-A-WSPTVGPNAPLQDSCAANG-------------VGSVASSVAAWTGA-GFPANKLVLGVPAYGRSYYVDPANAL------------SAAGE------------LTPYAQFDKSK--------QP-LGEGET--GE-QTVDQ-C--------G-VASGPS-------GLFNFAGLVDAG--------------------------------------------------------YLNP--D------------GTAAEGMVY-LCDQC----SETPFVY--QKDTGTMITYDDAESTAAKGN-FIAEQGLKGFAIWHGIGDY------------NDILLDAVSRGM---------GQ------------------------------------------------------------------------------------------------------------------------------------------------------------------------------------------------------------------------------------------------------------------------------------------------------------------------------------------------------------------------------------------------------------------------------------------------------------------------------------------------------------------------------------------------------------------------------------------------------------**

**>01586_C_cinereus_A_III**

**MTSP--------------------------------------------------------------------------------------------------------------------------------------------------------------------------------------------------------------------------------------------------------------------------------------------------TTVAIAGHTTASSP------------------RPHILYTVK------------------------------------------VNSGGKEYVSHR---RYSEFVTLHDILKDPFKLPPKRLLVTYFIPSAWVDDAL------ISERKSGLTNYLNNLLASPDFKNHSALTQFLLGEETSSPLLDPSRFNINF--------DLEDALPS-TLPRKAALSLAATRPTAAAVAAFA--------------------------------------------------------------------------EEVDGSPKTSERAMTNAETI------------------------------------------AKVSSTMIYAAYYPA--WSA-----GTHPPEKLD--FSKFDILFYAFVMPNSS--SG-ITWDGG-------------------------------------DK--DILRRLVAAARKSGYGTKIVLSVGGWG-GCYW----FSQACSTANNRTKFCNALMEVVNQYGLDGIDLDWEYPN---------SP-GAG-NPYSA----NDTANYLSLMKLLRN----AM------------GPCKILSSAVAHLPWLGSNGRPLTN--VSEFAAIMNYICIMNYDVWG-A--SGTPGPNAPYGNLCGTSKQP-----------HASAQAALAQWKAA-GFPANKQLLGLALYGWVSKSTKNVLTGAFMPTDDMVLLTKSETSSLDGNGEITFLNGAHPRPKDGAIAQSVAVENA-DGEGSI--AA-ASKDG--DEGKKKADGLVTVQAADLRRWWGQQIPFKTIVSSG--------------------------------------------------------ALVKKSD----------GTYGQGGGFTM-GWDNC----SNTPYLF--NTAQQTVISYDDTWSLNDKAK-LARDSGMAGCFSWSLDQDD------------GMSLQNVVRKAL---------GK------------------------------------------------------------------------------------------------------------------------------------------------------------------------------------------------------------------------------------------------------------------------------------------------------------------------------------------------------------------------------------------------------------------------------------------------------------------------------------------------------------------------------------------------------------------------------------------------------------**

**>EAA71245_G_zeae_A_III**

**------------------------------------------------------------------------ML----------------------------------------------------------------------------------------------------------------------------------------------------------------------------------------------------------------------------SLKSLSASLVGVL-----------------------------------------------------------------------------------------------------------------------------------------------------------------------------------------------------------------------------------------------------------------------------------------------------------------SLSSGATAA------------------------------------------ANNSPQLVASTYFAG--FHAN----RGFPVSAMP--WDKYTDAKYSFAETTED--GG-LDLSKS-------------------------------------QP--EELSCFVNAAKKN--GVKALVSIGGWT-GARY----FSTNFGNEKNRTAFVKTCVDFAKEHSLDGLDFDWEYPN---------RQ-GLGCNTINE----NDTANFLEFLKELRK----DP-----------VGKDLYLTAAGSLLPWNDKDGAPSKD--LGGFADVLDYLMIMNYDLYG-G-WSNVAGPNAALYSSCDERNN------------MGSGNQAVTKWNDA-GIPLSQIVLGMPNYGHGFKVNATSAY------------NKNHK------------LNLYPAQNSTD--------RF-QGSSWD--ND-PLIDA-C--------G-NPNPPG-------GTYPFWSLIKEAK-------------------------------------------------------FLDA--S------------GNPAPGIAY-TWDKC----SKTPFLY--DEEKQIYVSYDNARSFTEKGK-FVKAKGLGGFATYEAGGDY------------NNILIDAVRAAV---------GLS-----------------------------------------------------------------------------------------------------------------------------------------------------------------------------------------------------------------------------------------------------------------------------------------------------------------------------------------------------------------------------------------------------------------------------------------------------------------------------------------------------------------------------------------------------------------------------------------------------------**

**Subgroup-B dataset:**

>AbChit1

-------------------------------------------------------------------------------------------------------------------------------------------------------------------------------------------------------------------------------------------------------------------------------------------------------------------------------------------------------------MGLLL-------------LPVLAS---------------------VILGTGTGI------------------------------------------VTATSTCATKGRPTGKVLQGY--------WE-NW-------DGSSN------GVHPPFGWTPIQSPLI--------------------RQHGYNVISAAFPVILP----DGTALWQDGMDANVKV---------------------------PSPAD------MCEAKA-A-GATILMSIGGA-A----AAIDLSSSAV-ADRFV------ATVVPI---------LQ----------------------KYNFDGVDIDIETG--------LT-GSGDIKTLSASQANLMRVIDGILAQMPPN--------------------------FGLTMAPETAYVT---GGGVTYGSIWGAYLP--IIKKYVD----N-GRLWWLNMQYYNGAM--Y--G-CSG------------------DSYA--AGTIE--------GFVAQTDCLDKGLVVQGTTIRVPYDMQVPGLPAQSGA------GGG-S--MSPALVGQAWDHY------GGLLKGLMTWSINWDGSKG------WSFADNLNPRFAA---------------------------------VAAAAA------------------------------------------------------------------------------------------------------------------------------------------------------------------------------------------------------------------------------------------------------------------------------------------------------------------------------------------------------------------------------------------------------------------------------------------------------------------------------------------------------------------------------------------------------------------------------------------------------------------------------------------------------------------------------------------------------------------------------------------------------------------------------------------------------------------------------------------------------------------------------------------------------------------------------------------------------------------------------------------------------------------------------------------------------------------AQQP--------------------------------------------------------------------------------------------------------------------------------------------------PARNQSFVLESR------------------------------------------------------------------------------------------------------------------------------------------------------------------------------------------------

>AbChit2

------------------------------------------------------------------------------------------------------------------------------------------------------------------------------------------------------------------------------------------------------------------------------------------------------------------------------------------------------------------------------------------------------------MPP-IPPH----------------------------------------------------VNPLPRLITY--------Y----QTHHDSATG------------KHISILPLLT------------------------QPSISVTHV-ILAAIHINS-DPHNITLND--------------------HV-----PDHPRFQTLWAE------LRILQA-S-GIKVLGMLGGA-AK---GTYAR--LDG-DDN--------HQFETYYAP-LRHL-LQ----------------------TRNLDGIDLDVEEE--------MS-LSG-----------IIRLIDRLRADFGPS--------------------------FIITMAPVAMALL---DSTK---NLSGFDYE--ALEVMR-----G-REIAWYNTQFYCG-----------------------WGDLSNTFMYD--MMLQK--------GWSP--------------------DKLVIGVVTNPANG-----S-G-F--VAWDVLASVLGFL---NQRHQNFGGVMGWEYFNSLPGD--ADQPWQWASDLSLLLRS----------------------H------------SLAA------------PGPVSPV--------QETQ------------------------------------------------------------------------------------------------------------------------------------------------------------------------------------------------------------------------------------------------------------------------------------------------------------------------------------------------------------------------------------------------------------------------------------------------------------------------------------------------------------------------------------------------------------------------------------------------------------------------------------------------------------------------------------------------------------------------------------------------------------------------------------QNLQQE----------------------------------------------IDP------------DPVDPRPL-------------------------------------------------------------------------------------------------------QVPESFDYYSDGWGGD---------------------------------------------------------------------------------------------------------------------------------------------------------------------------------------------------------------------------------------------------------------------------------------------------------------------------------------------------------------------------------------------------------------

>AbChit4

------------------------------------------------------M----------------------------------------------------------------------------------------------------------------------------------------------------------------------------------------------------------------------------------------------------------------------------------------------------RFQWSIV-------------VAALTAW--------------------TWASP-VA-------------------------------------------------QVPRAIAELPRLVIY--------F----QTTHDS-NG------------APISMLPLVR------------------------EKGIALTHI-MISAFHLN--QGGQIHLND--------------------WP-----PDSPAFYTLWNE------TRVLKQ-A-GVKVMGMIGGA-AP---GSFNTATLDG-DQ---------VTFSSYYAQ-VRDM-VK----------------------RYQLDGLDLDVEQP--------MS-QNG-----------VFRLISALRADFGLG--------------------------FIISLAPVASALR---NGGA---NLSGFNYR--LLEQQQ-----G-PNINFYNTQFYSG-----------------------FGTMASPSDYS--AIVSS--------GFSA--------------------TRVVAGQLTSPANG-----N-G-Y--MTYDRLNATIVAL---RSAYGQIGGVMGWEYFNSLPGG--NAEPWQWAQVMTQILRP----------------------DSVPK----LSVNQAFA------------NKLIDAFGLSQMTVPAQPDSSSSS-------------------------------------------------------------------------------------------------------------------------------------------------------------------------------------------------------------------------------------------------------------------------------------------------------------------------------------------------------------------------------------------------------------------------------------------------------------------------------------------------------------------------------------------------------------------------------------------------------------------------------------------------------------------------------------------------------------------------------------------------------------------------------SSSSSSSM--------------------------------------------MSLNV--------AESSGGDRPM------------------------------------------------------------------------------------------------------------FDYQS---------------------------------------------------LVKPE-------------------------------------------------------------------------------------------------------------------------------------------------------------------------------------------------------------------------------------------------------------------------------------------------------------------------------------------------------------

>AbChit7

------------------------------------------------------MKI-----FTALVVAASALALGVPP--------VENSAKTALSARSANP----------RERRETSSLASPE---VDPQIK----GES-LWK---NNRKNRKPCTT-------------------RG----PTRAGGVMPKDSGELASGGSKITPGRG-DSSGPEVGETSADQDAGTSPDKPDLTTKHASNNEPEDERTNEPVPPLANDGIKTRMGDSATTSTTPNKMRGDSSVP----------EVGGTK--PDEANEANAG-----------------SSADQ----DTGTSPDKPNL-------------TSNHASNNETRDDHTSDDDNK------------TPIRDN---------------------------------------AITPTTPNKMRGDGKHKVAVY--------W------------GQHG-----GGL-PEKRLADYCKP-----------------------DQGIDILLLAFLYQWG----NGNTVPGGSIGNHCVI----------------EAKTGKPSECNDLAKD------IQTCQK-A-GVKIFLSIGGA-V----GAYSLSSKAE-GESLA------ENIWAAYGG-GSST--------SIPRPFG----------DVEVDGFDFNIENP--------AG-LGH-----------YQHAVTKLRGLIGGQ-------------------------DVKISSAPQCPIPE-----------------P--NLDKEIQ----A-SKFDILWVQFYNNPG-------CSL-----------------DSGTN--FDEWS--------QHIA--------------KGASAGAEIYVGAPAGPKAANGAESGAKFY--LEPTKMAELVAKL----KKMKGFGGVMMWAAGFSDANK---VGGKTYAQQAKCILGT-----------GKTC------------------------------------------------------------------------------------------------------------------------------------------------------------------------------------------------------------------------------------------------------------------------------------------------------------------------------------------------------------------------------------------------------------------------------------------------------------------------------------------------------------------------------------------------------------------------------------------------------------------------------------------------------------------------------------------------------------------------------------------------------------------------------------------------------------------------------------------------------------------------------------------------------------------------------------------------------------------------------------------------------------------------------------------------------------------------------------------------------------------------------------------------------------------------------------------------------------------------------------------------------------------------------------------------------------------------------------------------------------------------------------------------------------------------------------------------------------

>MAC_Chit1

------------------------------------------------------MFV---------------------------------------------------------------------------------------------------------------------------------------------------------------------------------------------------------------------------------------------------------------------------------------------------RNALAV-------------TGLLAA---------------------------LT-----------------------------------------------QAAPDEGRASGHKLTVY--------W------------GAED---------DTTTLDDVCN------------------------DSSYDVVNLAFLSHFF----SAGGYPKMSIGN--------LDGPS---QAQKKAGATGLQDGSSLVKS------IKNCQS-K-GKPVILSMGGA-TD--YSDVQLNSDAQ-GQQIA------NTVWNLFLG-GTDH--------KELRPFG----------DVKLDGVDLDNETN---------D-GTG-----------YLAMTKQFKANFQKD----------------------TSKKYYITAAPQCPYPD-----------------Q----SEPLD----VCQLLDWVQVQFYNNGN-------CNI----------------AQRGFAKAVKNWS--------------------------KGIGSGVQLYIGALASGADG-----DEG-Y--VHAATLNRAVNQV--KAMNLPNFGGAMLWEAHSAVKNG-------NYQKKIKANL-----------------------------------------------------------------------------------------------------------------------------------------------------------------------------------------------------------------------------------------------------------------------------------------------------------------------------------------------------------------------------------------------------------------------------------------------------------------------------------------------------------------------------------------------------------------------------------------------------------------------------------------------------------------------------------------------------------------------------------------------------------------------------------------------------------------------------------------------------------------------------------------------------------------------------------------------------------------------------------------------------------------------------------------------------------------------------------------------------------------------------------------------------------------------------------------------------------------------------------------------------------------------------------------------------------------------------------------------------------------------------------------------------------------------------------------------------------------------------

>MAC_Chit2

M-----------------------------------------------------SSY--------------------------------------------------------------------------------------------------------------------------------------------------------------------------------------------------------------------------------------------------------------------------------------------------FWTLASA-------------ASLLAA---------------------------LP-----------------------------------------------SALAGFNGGSHKNIAVY--------W------------GQNSFGQ-GSGPNVQRNLAHYCQGEFFLRMPKLGPFKPQLKLLLRRVDGDIGIIPIAFMNGI-----SPPITNFANAGDKCD----KFPG------------NSNLLKCPEIEQD------IKTCQTKF-KKTIVLSLGGA-TY---SQGGWSSTRD-AENAA------QSVWDMFGP-VPSG--S-----KVDRPFG----------SAVVDGFDFDFEST-----------TNN-----------LPAFGAKLRSLMDGA----------------------GGKKFYLTAAPQCVFPD-----------------A--AAGSALD----A-VAFDFVMIQFYNN-W-------CGVSNF--------QTGSATQYAFN--FDVWD--------KWAK--------------GSKNPNVKLLLGLPANKGA------GGG-Y--TN----------------------------------------------------------------------------------------------------------------------------------------------------------------------------------------------------------------------------------------------------------------------------------------------------------------------------------------------------------------------------------------------------------------------------------------------------------------------------------------------------------------------------------------------------------------------------------------------------------------------------------------------------------------------------------------------------------------------------------------------------------------------------------------------------------------------------------------------------------------------------------------------------------------------------------------------------------------------------------------------------------------------------------------------------------------------------------------------------------------------------------------------------------------------------------------------------------------------------------------------------------------------------------------------------------------------------------------------------------------------------------------------------------------------------------------------------------------------------------------------------------------------------------

>MAC_Chit3

---------------------------------------------------------------------------------------------------------------------------------------------------------------------------------------------------------------------------------------------------------------------------------------------------------------------------------------------------------------------------------------------------------------------------------------------------------------------------------------------------------------------------------------------------------YNVISAAFPVILP----DGTALWEDGMDVNVKV---------------------------ATPAE------MCQAKA-A-GATILMSIGGA-A----AAIDLSSSTV-ADKFI------STIVPI---------LK----------------------KYNFDGVDIDIEAG--------LS-GSGSINTLSASQANLIRIIDGILAQMPSN--------------------------FGLTMAPETAYVT---GGSVTYGSIWGAYLP--IIKKYAD----N-GRLWWLNMQYYNGAM--Y--G-CSG------------------DSYE--AGTVK--------GFIAQTDCLNKGLVIQGTTIRLPYSMQVPGLPAQPGA------GGG-Y--MSPSLVGQALDHY------HGSLKG-----------------------------------------------------------------------------------------------------------------------------------------------------------------------------------------------------------------------------------------------------------------------------------------------------------------------------------------------------------------------------------------------------------------------------------------------------------------------------------------------------------------------------------------------------------------------------------------------------------------------------------------------------------------------------------------------------------------------------------------------------------------------------------------------------------------------------------------------------------------------------------------------------------------------------------------------------------------------------------------------------------------------------------------------------------------------------------------------------------------------------------------------------------------------------------------------------------------------------------------------------------------------------------------------------------------------------------------------------------------------------------------------------------------------------------------------------------------------------------------------------------

>MAC_Chit4

M-----------------------------------------------------MPS-----------------------------------------------------------------------------------------------------------------------------------------------------------------------------------------------------------------------------------------------------------------------------------------------FFSIIASLGLL------------ASASLA------------------------------------------------------------------------------APLASRATGAQNVVY--------W------------GQNG-----GGVVENNDLATYCTK-----------------------EAGIDIVVLAFLYQYG----NGNKIASGTIGQSCSIS-----------------PSGEGQNCDALAKA------IDTCKA-N-GVKVVLSLGGA-S----GAYSLTSQQE-AEAIG------QNLWDAYGK-PGSNPAA-----SVPRPFG----------NTFVSGWDFDIESN--------SG-NNF-----------YQFMIAKLRSNFASD----------------------PGNQYFITGAPQCPIPE-----------------P--NMNEIIT----K-AQFDYLWVQFYNNPG-------CSV-----------------DGTIN--FGDWK--------KNVA--------------NTPSANAKIFIGVPASPLGATGTASGAKYY--LEPKKLAALVDQH----SSDPAFGGVMMWAAGFSDANV---NNGRTYAQEAKAILTA-----------ANSC------------------------------------------------------------------------------------------------------------------------------------------------------------------------------------------------------------------------------------------------------------------------------------------------------------------------------------------------------------------------------------------------------------------------------------------------------------------------------------------------------------------------------------------------------------------------------------------------------------------------------------------------------------------------------------------------------------------------------------------------------------------------------------------------------------------------------------------------------------------------------------------------------------------------------------------------------------------------------------------------------------------------------------------------------------------------------------------------------------------------------------------------------------------------------------------------------------------------------------------------------------------------------------------------------------------------------------------------------------------------------------------------------------------------------------------------------------

>MAC_Chit5

------------------------------------------------------MFG--------------------------------------------------------------------------------------------------------------------------------------------------------------------------------------------------------------------------------------------------------------------------------------------------EYSVTLLV------------ASVLAL---------------------------AS-----------------------------------------------SVLAGFDAASSKNVAVY--------W------------GQNSFGQ-VG---SQASLAFYCE------------------------DTRIDIIPISFMISL-----RDRTLNLGP-------------------------------SSPDLEDD------IKSCQR-L-GKTILLSFPGA-YY---TEGGFDSVEM-AIKGA------QDVWAAFGP-VQAY--S-----TISRPFG----------SAVVDGFDFDFESS---------D-IGY-----------IVAFGEELDRLRRYP----------------------SDKSIILTAAPQCPFPD-----------------Q--AMNELFE----K-IPFDALFIQFYNN-Y-------CGVKNF--------NPG-ADRSSFN--FDAWD--------RWAKQ-------------ESLSRRVKLLLGAPANVAAA-----GKGSY--VEPYVLAQAIAES----QKFESFGGVMFWDMTQLYSNV-------GFLDIVVGALEGNRFS------------------------------TPSSE-------------PAPS----STEPVCPSYGRRSRAIL---------------------------------SRGFGGTLV---------------------------LGTFGACSPLY-----------------------------------------------------------------------------------------------------G--------------ARCP------PYG-------------RRY-GPI--------------PSSSH------------------------------------------------------------------------------------------------------------------------------------------------------------------------------------------------------------------------------------------------HHCRLGQ-------------------------------------------------------------------------------------------------------------------------------------------------------------------------------------------------------------------------G-------------------------------VHHSGPRANDLDH--------------------------------------------------------------------------------------------------------------------------------------------------------------------------------------------------------------QGRGTGAIASFSS------------------------------PGQTR-------------------------------------------------PTRPTR------------------------------------------------------------------------PSQ---------------------------------------------------------------------------------------------------------------------------------------------------------------------------------------------------------

>MAC_Chit6

MLLAAFIEPKVVRKEEDLVIFQAVPAYTPFLVISSLPINPGGLQRLVILTPENKMFV---------------------------------------------------------------------------------------------------------------------------------------------------------------------------------------------------------------------------------------------------------------------------------------------------RNALAV-------------TGLLAA---------------------------LT-----------------------------------------------QAAPDEGRASGHKLTVY--------W------------GAED---------DTTTLDDVCN------------------------DSSYDVVNLAFLSHFF----SAGGYPKMSIGN--------LDGPS---QAQKKAGATGLQDGSSLVKS------IKNCQS-K-GKPVILSMGGA-TD--YSDVQLNSDAQ-GQQIA------NTVWNLFLG-GTDH--------KELRPFG----------DVKLDGVDLDNETN---------D-GTG-----------YLAMTKQFKANFQKD----------------------TSKKYYITAAPQCPYPD-----------------Q----SEPLD----VCQLLDWVQVQFYNNGN-------CNI----------------AQRGFAKAVKNWS--------------------------KGIGSGVQLYIGALASGADG-----DEG-Y--VDAATLNRAVNQV--KAMNLPNFGGAMLWEAQLAVKNG-------NYQKKIKANL-----------------------------------------------------------------------------------------------------------------------------------------------------------------------------------------------------------------------------------------------------------------------------------------------------------------------------------------------------------------------------------------------------------------------------------------------------------------------------------------------------------------------------------------------------------------------------------------------------------------------------------------------------------------------------------------------------------------------------------------------------------------------------------------------------------------------------------------------------------------------------------------------------------------------------------------------------------------------------------------------------------------------------------------------------------------------------------------------------------------------------------------------------------------------------------------------------------------------------------------------------------------------------------------------------------------------------------------------------------------------------------------------------------------------------------------------------------------------------

>MAC_Chit7

------------------------------------------------------MAP-------------------------------------------------------------------------------------------------------------------------------------------------------------------------------------------------------------------------------------------------------------------------------------------------LFNTGFVL-------------LPIIVS---------------------TLLGP-IPA------------------------------------------FAQNGTCATKGKPAGRVLQGY--------WE-NW-------DGGKN------GVHPPFGWTPIQDAQI--------------------RQHGYNVISAAFPVILP----DGTALWEDGMDVNVKV---------------------------ATPAE------MCQAKA-A-GATILMSIGGA-A----AAIDLSSSTV-ADKFI------STIVPI---------LK----------------------KYNFDGVDIDIEAG--------LS-GSGSINTLSASQANLIRIIDGILAQMPSN--------------------------FGLTMAPETAYVT---GGSVTYGSIWGAYLP--IIKKYAD----N-GRLWWLNMQYYNGAM--Y--G-CSG------------------DSYE--AGTVK--------GFIAQTDCLNKGLVIQGTTIRLPYSMQVPGLPAQPGA------GGG-Y--MSPSLVGQALDHY------HGSLKGLMTWSINWDGSKG------WTFGDNLKRRL-----------------------------------------------------------------------------------------------------------------------------------------------------------------------------------------------------------------------------------------------------------------------------------------------------------------------------------------------------------------------------------------------------------------------------------------------------------------------------------------------------------------------------------------------------------------------------------------------------------------------------------------------------------------------------------------------------------------------------------------------------------------------------------------------------------------------------------------------------------------------------------------------------------------------------------------------------------------------------------------------------------------------------------------------------------------------------------------------------------------------------------------------------------------------------------------------------------------------------------------------------------------------------------------------------------------------------------------------------------------------------------------------------------------------------------------------------------------------------

>MAC_Chit8

------------------------------------------------------------------------------------------------------------------------------------------------------------------------------------------------------------------------------------------------------------------------------------------------------------------------------------------------------------------------------------------------------------MPP-IPPH----------------------------------------------------KHPLPRLITY--------Y----QTHHTP-SG------------EHISVLPLLT------------------------QPSISLTHL-ILAACHINE-PPSQLTLND--------------------HP-----PSHPRFQTLWAE------LRVLQA-S-GVKVLLMLGGA-AQ---GTYQR--LES------------DQFEAYYAP-LKQL-IR----------------------ERNLDGVDLDVEEP--------MS-LGG-----------IIRLIDRLRADFGPG--------------------------FVITLAPVAMALL---DPEK---NLSGFDYE--ALEVMR-----G-KEIAWYNTQFYCG-----------------------WGNLTNTLMYD--LMLQK--------GWSP--------------------EKLVVGVVTNPANG-----S-G-F--VPWEMLSAILAIL---HRRHRNFGGVMGWEYFNSLPGD--ASRPWEWAGNMSSLLRA----------------------H------------SL-G------------PVPTPAG--------TDIQ------------------------------------------------------------------------------------------------------------------------------------------------------------------------------------------------------------------------------------------------------------------------------------------------------------------------------------------------------------------------------------------------------------------------------------------------------------------------------------------------------------------------------------------------------------------------------------------------------------------------------------------------------------------------------------------------------------------------------------------------------------------------------------PSVDGE----------------------------------------------VDP------------DPINNRPL-------------------------------------------------------------------------------------------------------EVPKSFDYYSDGTDHE---------------------------------------------------------------------------------------------------------------------------------------------------------------------------------------------------------------------------------------------------------------------------------------------------------------------------------------------------------------------------------------------------------------

>MAC_Chit9

-----------------------------------------------------------------------------------------------------------------------------------------------------------------------------------------------------------------------------------------------------------------------------------------------------------------------------------------------------------MLLKSCL-------------IAALAAV--------------------SLASP-IH-------------------------------------------------QVVRAVDELPRLVVY--------F----QTTHDS-SG------------RPISMLPLIN------------------------EKGIALTHL-IVCSLHVN--ENGQIHLND--------------------YP-----PSNPMFYTLWNE------TAVMKN-A-GVKIMGMVGGA-AP---GSFGSGTLDG-DK---------ATFNKYYGQ-LRDV-IR----------------------KFKLQGLDIDVEQP--------MS-QAG-----------IERLVDKLQSDFGPN--------------------------FIITLAPVASALS---GGG----NLSGFDYK--LLDSRK-----G-SNINFYNGQFYSG-----------------------FGTMSRTTDYN--GIVSS--------GFSA--------------------SRVVAGQLTSPEGG-----Y-G-Y--IPYEQLNATVISL---RSTYGQIGGVMGWEYFNSAPGG--TDEPWKWAQIMTQILRP----------------------NSVPK----LTISTEIA------------QKLATAFNDSVKSNIAWSTKTDGA-------------------------------------------------------------------------------------------------------------------------------------------------------------------------------------------------------------------------------------------------------------------------------------------------------------------------------------------------------------------------------------------------------------------------------------------------------------------------------------------------------------------------------------------------------------------------------------------------------------------------------------------------------------------------------------------------------------------------------------------------------------------------------PGVDYHGM--------------------------------------------VNV----------------------------------------------------------------------------------------------------------------------------------------------------------------------------------------------------------------------------------------------------------------------------------------------------------------------------------------------------------------------------------------------------------------------------------------------------------------------------------------------------------------------------------------------------------

>MAC_Chit13

M-----------------------------------------------------AFP--------------------------------------------------------------------------------------------------------------------------------------------------------------------------------------------------------------------------------------------------------------------------------------------------VLTLWST-------------ASILAI---------------------------LP-----------------------------------------------ATLAGFNSGSGKNVAVY--------W------------GQNSYNQ-GSGPLAQQRLSYYCS------------------------NAEIDIIPVAFMNGI-----SPPITNFANAGDNCT----AFPS------------NSNLLSCPQIEAD------IKSCQVTN-GKTIILSLGGA-TY---GQGGWSSVSD-AQAAA------QNVWDMFGP-VPSG--K-----TIDRPFG----------SAVVDGFDFDFEAS-----------TNN-----------LPAFGQKLRSLMDAA----------------------GGKKFYLSAAPQCVFPD-----------------A--AVGAALN----A-VSFDFIMIQFYNN-W-------CGVSNF--------QEGSTTQNAFN--FDVWD--------NWAKT-------------TSPNKKVKLLIGVPAAPGA------GGG-Y--TSGSKLKAAINWS----QKYSSFGGAMMWDMSQLYSNS-------AFLGEIVSDISN----------------------------------GPTTT-------------LPPG---GTTTTTSSST-------------------------------------------------------------------------------------P----------------------------------------------------------------------------------------------------PPTG--------------TLVPQWGQCG--G-------------EGYTGST--------------QCQPP------------------------------------------------------------------------------------------------------------------------------------------------------------------------------------------------------------------------------------------------YKCTYG---------------------------------------------------------------------------------------------------------------------------------------------------------------------------------------------------------------------------GQWW----------------------------SSCQ-----------------------------------------------------------------------------------------------------------------------------------------------------------------------------------------------------------------------------------------------------------------------------------------------------------------------------------------------------------------------------------------------------------------------------------------------------------------------------------------------------------------------------------------------------------------------------------------------------------------

>MAC_Chit16

---------------------------------------------------------------------------------------------------------------------------------------------------------------------------------------------------------------------------------------------------------------------------------------------------------------------------------------------------------LRALAGVGL-------------VGLASG---------------------------VPF--------------------------------------------TDNISIKPRQAPGAQNIVY--------W------------GQNG-----GGTIENNDLAAYCQP-----------------------NSGIDVLVLAFLYQFG----NGGNIPSGTIGQSCYIS-----------------TSGQGQNCEALTAA------IQTCQS-A-GVKIILSLGGA-T----SSYSLQTQAQ-AEQIG------QYLWDSYGN-SGNK--------TVQRPFG----------SNFVNGFDFDIEVN--------GGSSQY-----------YQYMIAKLRSNFGSD----------------------EANTYYITGAPQCPIPE-----------------P--NMGVIIS----N-SVFDHLYVQFYNNNN--Y-TVPCAL-------------GINGNAPFN--YNNWT--------SFIS--------------NTPSANAKVFIGVPASPLASTGTPSGAQYY--ATPDQLAAIVGEY----KGDAHFGGIMMWSAGFSDANV---NNGCTYAQQAKNILVN-----------GAACGS-----SGPPI-------PTPTT--------------TPAT--TTPTTASST------------------------------------------------------------------------------------FSPT--------------------------------------------------------------------------------------------------ASPTG--------------GTVPQWGQCG--G-------------EGYSGPT--------------QCVPP------------------------------------------------------------------------------------------------------------------------------------------------------------------------------------------------------------------------------------------------YKCVKQ---------------------------------------------------------------------------------------------------------------------------------------------------------------------------------------------------------------------------GDWW----------------------------SSCR-----------------------------------------------------------------------------------------------------------------------------------------------------------------------------------------------------------------------------------------------------------------------------------------------------------------------------------------------------------------------------------------------------------------------------------------------------------------------------------------------------------------------------------------------------------------------------------------------------------------

>MAC_Chit18

------------------------------------------------------MHH------------------------------------------------------------------------------------------------------------------------------------------------------------------------------------------------------------------------------------------------------------------------------------------------LRALAGVGL-------------VGLASG---------------------------VPF--------------------------------------------TDNISIKPRQAPGAQNIVY--------W------------GQNG-----GGTIENNDLAAYCQP-----------------------NSGIDVLVLAFLYQFG----NGGNIPSGTIGQSCYIS-----------------TSGQGQNCEALTAA------IQTCQS-A-GVKIILSLGGA-T----SSYSLQTQAQ-AEQIG------QYLWDSYGN-SGNK--------TVQRPFG----------SNFVNGFDFDIEVN--------GGSSQY-----------YQYMIAKLRSNFGSD----------------------EANTYYITGAPQCPIPE-----------------P--NMGVIIS----N-SVFDHLYVQFYNNNN--Y-TVPCAL-------------GINGNAPFN--YNNWT--------SFIS--------------NTPSANAKVFIGVPASPLASTGTPSGAQYY--ATPDQLAAIVGEY----KGDAHFGGIMMWSAGFSDANV---NNGCTYAQQAKNILVN-----------GAACGS-----SGPPI-------PTPTT--------------TPAT--TTPTTASST------------------------------------------------------------------------------------FSPT--------------------------------------------------------------------------------------------------ASPTG--------------GTVPQWGQVS--------------------------------------LTPK------------------------------------------------------------------------------------------------------------------------------------------------------------------------------------------------------------------------------------------------SRLCS-------------------------------------------------------------------------------------------------------------------------------------------------------------------------------------------------------------------------------W----------------------------RKKKKKL---------------------------------------------------A------------------------------------------------------------------------------------------------------------------------------------------------------------------------------------------------------------------------------------------R---------------------------------------------------------------------------------------------------------------------------------------------------------------------------------------------------------------------------------------------------------------------------------------------------------------

>MAC_Chit21

-------------------------------------------------------------------------------------------------------------------------------------------------------------------------------------------------------------------------------------------------------------------------------------------------------------------------------------------------------------------------------MSLSLG---------------------------LGV-----------------------------------------------------GGAAPALNAY--------W------------GQT----------GGRFLRDIC-------------------------DSGVNYATVSFINNSPE---HGDGYPGSNFGANCAGE--VYIK--------DGKNTKLLSACTFIQRD------IPYCQS-K-GVKVLLAIGGA-PLPGISDYDVSSEEK-GVEFA------EFLYNAFGP-YKSS--W-----TGPRPFDK-----SPTEHVSIDGFDLDLEDRS-----TKFS-NKP-----------YIAMVNWWRQQ---------------------------SHKMFITAAPECVITR---NGN----------FN--QNDELIA----N-AEFDALFIQFYNNPV-------CDA-----------IPNNTPGDEFS--YDDWA--------ANVA--------------KGKSKDAKLFIGLPASTDSA-----GSG-Y--IVPKDMKDLVCKY----STHKNFGGVSLWDATRAMNNE--DAEGKTFLQSAVEAVQY-------------VC-------QEPP--------KTSSS-------------TSTSTKIISTTSSTTVKTTP--------------------------------------ASSTTSSQPTATSASSQ----------------KPTVSSFSE-----RLT-------------------------TASTNSGNVTTTSIPSGK--------------PTT----------------------------------SSATS--------------EKLDTTSVIAAAS--------------ATKSAT------------------------------------------------------------------------------------------------------------------------------------------------------------------------------------------------------------------------------------------------------------------------------------------------------------TSSARMTS----------------------------------------------------------------------------------------------------------------------------------------SIHWSNSTATGTVQ----------------TTSKPVSMTTSTVYTTSVHTV---------------------TECPPTVTDCPV--G-------------HVTTETIALY------TTVCP-------VSE------------NAQPPKPT---------TTKAAVMTTSTVYTTKTYTITECPPAVTDCPV-------GRVTTEVIP-VYTTVCPVTETRHPPKPT-----------TTKVPV-----------MTTSTVYTTKT-------YTITKCLPTVT-DCP--VGHITTEVVPAYTTVCPVKGTEPGSVPKP----------TG--PASLSA----------------ATETTVVT------------------------KTIKVQQ-----------VSSLSTSIRSTAVV---------------------PQPSSK----GCSGLSC--------PGATNAP---------------------------------------------------------------GSGCTGPECPGVAI---------------------------PTNSWTNKHGSGCTGPEC-SGVAIPTNAWT--------SSPVGP--SAVASS--PVTAGAST--LALGL-TGL------VAIV--AAQVLAI-

>MR_Chit1

------------------------------------------------------MFV---------------------------------------------------------------------------------------------------------------------------------------------------------------------------------------------------------------------------------------------------------------------------------------------------RNALVV-------------TGLLAA---------------------------LT-----------------------------------------------QAAPAERNTSRHKLTVY--------W------------GAED---------DTTTLDDVCN------------------------DSSYDVVNLAFLSHFF----SGGGYPRMSIGN--------LDGPS---RAQKKAGATGLQDGSSLVKS------IKNCQS-K-GKPVILSMGGA-TD--YSDVQLHSDAQ-GQQIA------NTVWDLFLG-GTDH--------KELRPFG----------DVKLDGVDLDNETN---------D-GTG-----------YLAMAKQFKANFQKD----------------------TSKKYYITAAPQCPYPD-----------------Q----SEPLD----VCRLLDWVQVQFYNNGN-------CNI----------------AQSGFATAVKNWS--------------------------RGIGSGVQLYIGALASGADG-----DEG-Y--VDAAVLNRAIDQV--KAMDLPNFGGAMLWEAQLAVNNG-------NYQKEIKANL-----------------------------------------------------------------------------------------------------------------------------------------------------------------------------------------------------------------------------------------------------------------------------------------------------------------------------------------------------------------------------------------------------------------------------------------------------------------------------------------------------------------------------------------------------------------------------------------------------------------------------------------------------------------------------------------------------------------------------------------------------------------------------------------------------------------------------------------------------------------------------------------------------------------------------------------------------------------------------------------------------------------------------------------------------------------------------------------------------------------------------------------------------------------------------------------------------------------------------------------------------------------------------------------------------------------------------------------------------------------------------------------------------------------------------------------------------------------------------

>MR_Chit2

M-----------------------------------------------------YSS--------------------------------------------------------------------------------------------------------------------------------------------------------------------------------------------------------------------------------------------------------------------------------------------------FWSLASA-------------ASLLAA---------------------------LP-----------------------------------------------SALAGFNGGSHKNIAVY--------W------------GQNSFGQ-GSGPNVQQGLAHYCE------------------------NADMGIIPIAFMNGI-----SPPITNFANAGDKCD----KFPD------------NSNLLKCPEIEKD------IKTCQTKF-NQTIVLSLGGA-TY---SQGGWSSTRD-AEKAA------QSVWDMFGP-VPSG--S-----KVDRPFG----------SAVIDGFDFDFEST-----------TNN-----------LPAFGAKLRSLMDGA----------------------GGKKFYLTAAPQCVFPD-----------------A--AVGSALK----A-VAFDFVMIQFYNN-W-------CGVSNF--------KPGSDTQDAFN--FDVWD--------KWAK--------------GSKNPNVKLLLGIPANKGA------GGG-Y--TNGEKLKAVIAYS----KKFTSFGGVMMWDMSQLYANE-------GFLGEVVSDLA----------------------------------------------------------------------------------------------------------------------------------------------------------------------------------------------------------------------------------------------------------------------------------------------------------------------------------------------------------------------------------------------------------------------------------------------------------------------------------------------------------------------------------------------------------------------------------------------------------------------------------------------------------------------------------------------------------------------------------------------------------------------------------------------------------------------------------------------------------------------------------------------------------------------------------------------------------------------------------------------------------------------------------------------------------------------------------------------------------------------------------------------------------------------------------------------------------------------------------------------------------------------------------------------------------------------------------------------------------------------------------------------------------------------------------------------------------------------------

>MR_Chit3

-----------------------------------------------------------------------------------------------------------------------------------------------------------------------------------------------------------------------------------------------------------------------------------------------------------------------------------------------------------MFRSLYL-------------LAILIPL--------------------SLASP-IV-------------------------------------------------EVVYTIPILPRLVVY--------F----QTTHDS-NG------------APISMLPLIK------------------------DKSIALTHI-IISSFHVQ--KNREIHLND--------------------FP-----PSDPMFYTLWNE------TRIMKE-A-GVKVMGMIGGA-AP---GSFTSETLDG-DE---------DVFEKYYTQ-IREV-IF----------------------RYELDGLDIDVEQP--------MS-QDG-----------IERLIDKIHCDFGSG--------------------------FIITLAPVASALK---NGG----NLSGLDYK--LLEYRK-----G-CKISFYNCQFYGG-----------------------FGTMSSTKDYH--DIVTN--------GFKA--------------------SRVVAGQLSSGTLT-----Y-D-R--LGQLNINTTIVSL---REKYGRIGGIMGWEYGLSVTRG--MDEPWRWAQAMTQILRP----------------------KFLSS----RATADQLG------------GGI---------------------------------------------------------------------------------------------------------------------------------------------------------------------------------------------------------------------------------------------------------------------------------------------------------------------------------------------------------------------------------------------------------------------------------------------------------------------------------------------------------------------------------------------------------------------------------------------------------------------------------------------------------------------------------------------------------------------------------------------------------------------------------------------------------------------------------------------------------------------------------------------------------------------------------------------------------------------------------------------------------------------------------------------------------------------------------------------------------------------------------------------------------------------------------------------------------------------------------------------------------------------------------------------------------------------------------------------------------------------------------------------------------------------

>MR_Chit4

------------------------------------------------------MPS-----------------------------------------------------------------------------------------------------------------------------------------------------------------------------------------------------------------------------------------------------------------------------------------------LFSTLASLALL------------ASTSLA---------------------------AP------------------------------------------------LAPLAPRAPGAQNVVY--------W------------GQNG-----GGVVENNDLAAYCTA-----------------------SSGIDIIVLAFLYQYG----NGHTIPSGTIGQSCYIA-----------------PSGEGQNCDALAKA------IDTCRT-N-GVKVLLSLGGA-S----GAYSLTSQQE-AEAIG------QNLWDAYGK-PGSN--A-----AVPRPFG----------NTSVDGWDFDIESN--------AG-NGF-----------YQFMIAKLRANFASD----------------------PGRAYLVTGAPQCPIPE-----------------P--NMNEIIT----K-AQFDYLWVQFYNNPG-------CSV-----------------DGTIN--FAEWK--------SNVA--------------GTPSANAKIFIGVPASPLGATGTASGARYY--LEPGKLAALVGRY----SSDPAFGGVMMWAAGFSDANV---RDGRTYAQVAKAILANDTAVPT--DVDASAC------------------------------------------------------------------------------------------------------------------------------------------------------------------------------------------------------------------------------------------------------------------------------------------------------------------------------------------------------------------------------------------------------------------------------------------------------------------------------------------------------------------------------------------------------------------------------------------------------------------------------------------------------------------------------------------------------------------------------------------------------------------------------------------------------------------------------------------------------------------------------------------------------------------------------------------------------------------------------------------------------------------------------------------------------------------------------------------------------------------------------------------------------------------------------------------------------------------------------------------------------------------------------------------------------------------------------------------------------------------------------------------------------------------------------------------------------------

>MR_Chit6

------------------------------------------------------MLS-----------------------------------------------------------------------------------------------------------------------------------------------------------------------------------------------------------------------------------------------------------------------------------------------CTGLISSLALL-----------VGSTVFA------------------------------------------------------------------------------LDQCTPRGGAQNVVY--------W------------GQNG-----GGIVENNSLAAYCTK-----------------------ESGIDIVVLSFLYQYG----NGLRIPSGTIGQSCFIS-----------------TTGEGQQCDDLARA------IDVCKS-N-GIKVIISLGGG-S----GAYSLSSREE-AETIG------QNLWLAYGN-SKSN--S-----SIPRPFG----------KTFVDGWDFDIESN--------SG-TQF-----------YEFLIAKLRSNFASD----------------------RVNKYFITGAPQCPIPE-----------------P--NMNQIIT----R-AQFDYLWVQFYNNPG-------CSV-----------------DGTIN--YNSWK--------KNIA--------------NSPSSDAKIFIGVPASPLGATGTQSGGKYY--LEPDQLSNLTRIY----TNDPAFGGIMIWAAGFSDHNV---NNGRTYAQQAKCILTS-----------GKTCQRA------------------------------------------------------------------------------------------------------------------------------------------------------------------------------------------------------------------------------------------------------------------------------------------------------------------------------------------------------------------------------------------------------------------------------------------------------------------------------------------------------------------------------------------------------------------------------------------------------------------------------------------------------------------------------------------------------------------------------------------------------------------------------------------------------------------------------------------------------------------------------------------NFFT-----------------------------------------------------------------------------------------------------------------------------------------------------------------------------------------------------------------------------------------------------------------------------------------------------------------------------------------------------------------------------------------------------------------------------------------------------------------------------------------------------------------------------------------------------------

>MR_Chit7

------------------------------------------------------MAP-------------------------------------------------------------------------------------------------------------------------------------------------------------------------------------------------------------------------------------------------------------------------------------------------LLNTGLVI-------------LPLIVS---------------------TLLGP-MPA------------------------------------------FAQNETCATKGKPAGKVLQGY--------WE-NW-------DGAKN------GVHPPFGWTPIQDAQI--------------------RQHGYNVISAAFPVILP----NGTALWEDGMDANVKV---------------------------ATPAE------MCQAKA-A-GATIVMSIGGA-T----AAIDLSSSSV-ADKFV------STIVPI---------LK----------------------RYNFDGVDIDIEAG--------LS-GSGTIGTLSTSQANLVRIIDGILAQMPSN--------------------------FGLTMAPETAYVT---GGSVTYGAIWGAYLP--IIKKYAD----N-GRLWWLNMQYYNGDM--Y--G-CSG------------------DSYK--AGTVE--------GFVAQTDCLNKGLVIQGTTIRVPYDKQVPGLPAQSGA------GGG-Y--MSPSLVGQAWDHY------NGSLKGLMTWSINWDGSKG------WTFGDNVKGRL-----------------------------------------------------------------------------------------------------------------------------------------------------------------------------------------------------------------------------------------------------------------------------------------------------------------------------------------------------------------------------------------------------------------------------------------------------------------------------------------------------------------------------------------------------------------------------------------------------------------------------------------------------------------------------------------------------------------------------------------------------------------------------------------------------------------------------------------------------------------------------------------------------------------------------------------------------------------------------------------------------------------------------------------------------------------------------------------------------------------------------------------------------------------------------------------------------------------------------------------------------------------------------------------------------------------------------------------------------------------------------------------------------------------------------------------------------------------------------

>MR_Chit8

------------------------------------------------------------------------------------------------------------------------------------------------------------------------------------------------------------------------------------------------------------------------------------------------------------------------------------------------------------------------------------------------------------MPP-IPPH----------------------------------------------------KHPLPRIITY--------Y----QTHHAP-SG------------EHISVLPLLT------------------------QPSISLTHL-ILAACHINA-SPSQLTLND--------------------HP-----PSHPRFQTLWAE------LRILQA-S-GVKVLLMLGGA-AP---GTYQR--LDS-ES---------DQFESYYAP-LRQL-IR----------------------DKNLDGVDLDVEEP--------MS-LAG-----------IVRLVDRLRADFGPG--------------------------FVITLAPVAMALL---DPEK---NLSGFDYE--ALEVMR-----G-KEIAWYNTQFYCG-----------------------WGNLTNTLMYD--LMLQK--------GWSP--------------------EKLVVGVVTNPANG-----S-G-F--VPWEMLSAILAIL---HRRHRNFGGVMGWEYFNSLPGD--ASRPWEWAGNMSSLLRA----------------------H------------SL-G------------PVPTPAG--------TDIQ------------------------------------------------------------------------------------------------------------------------------------------------------------------------------------------------------------------------------------------------------------------------------------------------------------------------------------------------------------------------------------------------------------------------------------------------------------------------------------------------------------------------------------------------------------------------------------------------------------------------------------------------------------------------------------------------------------------------------------------------------------------------------------PSVDGE----------------------------------------------VDP------------DPVNNRPL-------------------------------------------------------------------------------------------------------EVPKSFDYYSDGTDHE---------------------------------------------------------------------------------------------------------------------------------------------------------------------------------------------------------------------------------------------------------------------------------------------------------------------------------------------------------------------------------------------------------------

>MR_Chit9

------------------------------------------------------------------------------------------------------------------------------------------------------------------------------------------------------------------------------------------------------------------------------------------------------------------------------------------------------------MLKSCL-------------IAALAAV--------------------SLASP-IH-------------------------------------------------QVVRAVAELPRLVVY--------F----QTTHDS-SG------------RPISMLPLIE------------------------EKGIALTHL-IVCSLHVN--ENGQIHLND--------------------YP-----PSNPMFYTLWNE------TAVMKN-A-GVKIMGMVGGA-AP---GSFSSGTLDG-DT---------ATFNKYYGQ-LRDV-IK----------------------KFKLQGLDIDVEQP--------MS-QAG-----------IERLVDKLRSDFGPS--------------------------FIITLAPVASALS---DGG----NLSGFDYK--LLDSRK-----G-SKINFYNGQFYSG-----------------------FGTMSRPTDYT--TIVSN--------GFSA--------------------SRVVAGQLTSPSNG-----Y-G-Y--IPYEQLNATVISL---RDKYGQIGGVMGWEYFNSAPGG--TDEPWKWAQIMTQILRP----------------------DSVPK----LAISTEIA------------QKLTTAFNDSVKSNPVWATKSGGV-------------------------------------------------------------------------------------------------------------------------------------------------------------------------------------------------------------------------------------------------------------------------------------------------------------------------------------------------------------------------------------------------------------------------------------------------------------------------------------------------------------------------------------------------------------------------------------------------------------------------------------------------------------------------------------------------------------------------------------------------------------------------------LGVDYHRM--------------------------------------------INV----------------------------------------------------------------------------------------------------------------------------------------------------------------------------------------------------------------------------------------------------------------------------------------------------------------------------------------------------------------------------------------------------------------------------------------------------------------------------------------------------------------------------------------------------------

>MR_Chit13

M-----------------------------------------------------TFP--------------------------------------------------------------------------------------------------------------------------------------------------------------------------------------------------------------------------------------------------------------------------------------------------VHTWPSI-------------ASILAI---------------------------LP-----------------------------------------------ATLAGFNSGSGKNVAVY--------W------------GQNSYNQ-GSGPLAQQRLSYYCS------------------------NTEIDVIPVAFMNGI-----SPPITNFANAGDNCT----AFPS------------NSNLLSCPQIEAD------IKSCQATN-GKTIILSLGGA-TY---GQGGWSSVSA-AQAAA------QNVWDMFGP-VPSG--K-----AIDRPFG----------SAVVDGFDFDFEAS-----------TNN-----------LPAFGQKLRSLMDAA----------------------GGKKFYLTAAPQCVFPD-----------------A--AVGAALN----A-VSFDFIMIQFYNN-W-------CGVSNF--------QEGSTTQNAFN--FDVWD--------NWAKT-------------TSPNKNIKLLIGVPAAPGA------GGG-Y--TSGSKLKAAINWS----QKYSNFGGAMMWDMSQLYSNS-------AFLGEIVSDISN----------------------------------GPTTT-------------LPPG---ATTTTTTTTT--------------------------------------------TATTTT---------------------------TTTSSTTSP----------------------------------------------------------------------------------------------------APTG--------------TLVPQWGQCG--G-------------EGYTGPT--------------QCQPP------------------------------------------------------------------------------------------------------------------------------------------------------------------------------------------------------------------------------------------------YKCVYG---------------------------------------------------------------------------------------------------------------------------------------------------------------------------------------------------------------------------GQWW----------------------------SSCQ-----------------------------------------------------------------------------------------------------------------------------------------------------------------------------------------------------------------------------------------------------------------------------------------------------------------------------------------------------------------------------------------------------------------------------------------------------------------------------------------------------------------------------------------------------------------------------------------------------------------

>MR_Chit16

------------------------------------------------------MHH------------------------------------------------------------------------------------------------------------------------------------------------------------------------------------------------------------------------------------------------------------------------------------------------LRALVGVGL-------------AGLAAG---------------------------VPL--------------------------------------------TDKISVKPRQAPGAQNVVY--------W------------GQNG-----GGTIENNDLAAYCQP-----------------------NSGIDVLVLAFLYQFG----NGGNIPSGTIGQSCYIS-----------------TSGQGQNCEALTAA------IHTCQS-A-GVKIILSLGGA-T----SSYSLQTQAQ-AEQIG------QYLWDSYGN-SGNK--------TVQRPFG----------SNFVNGFDFDIEVN--------GGSSQY-----------YQYMIAKLRSNFASD----------------------KSNTYLITGAPQCPIPE-----------------P--NMGVIIS----N-SVFDHLYVQFYNNNN--Y-TVPCAL-------------GINGNAPFN--YNNWT--------SFIA--------------DTPSAGAKIFIGVPASPLASTGTPSGAQYY--AAPDQLAAIVGEY----RSDAHFGGIMMWSAGFSDANV---NNGCTYAQQAKSILVN-----------GAPCAS-----SGPPS-------STPAT--------------APAP--TATTMPSSTS----------------------------------------------------------------------------------VSSPA--------------------------------------------------------------------------------------------------ASPTG--------------GTVPQWGQCG--G-------------EGYSGPT--------------QCVAP------------------------------------------------------------------------------------------------------------------------------------------------------------------------------------------------------------------------------------------------YQCVKQ---------------------------------------------------------------------------------------------------------------------------------------------------------------------------------------------------------------------------GDWW----------------------------SSCR-----------------------------------------------------------------------------------------------------------------------------------------------------------------------------------------------------------------------------------------------------------------------------------------------------------------------------------------------------------------------------------------------------------------------------------------------------------------------------------------------------------------------------------------------------------------------------------------------------------------

>MR_Chit20

------------------------------------------------------MKFLLASHFIACISVITAAA--------------------------------------------------------------C--GQKVLWVA--N-------CGD------------------TVGA--LP----------------------AGMG-DTFKALNSAVVSDINSIYAGSTYTVAYAHTTTS--------------ASWSYTTRGGNRYLVSPT------NSAVC---------------------TQHANVG--------------------------GVFGATTAVATTQSSAQRQITPVPLSPTWA---------------------------TPVA------------------------------------------AVQTRTLTGTAPDSAQNVVY--------W------------GQHG-----GAV-PENELAAFCTR-----------------------EAGIDIIVLAFLFKYG----NGNVIPSGSFGYSCTI--------------------GEGQQCDSLAKA------IETCKS-N-GIKVIVSLGGN-A----GDYSLSSKEE-AEKIG------QNLWDAYGN-SNKT--G-----SVPRPFG----------KTFVDGWDFNIEHN--------SG-SKY-----------YEFLIASLRSNFASD----------------------PGNKYLITGAPQCPIPE-----------------P--NMGDIIS----R-AKFDYLWVQFYNNHE-------CSV-----------------NGGIN--YDDWT--------RMVA--------------NTPSANAKIFIGVPAHPHAANGQDSGAIYY--LQPSKLARLVGQY----RSDPAFGGIMIWAAGLSDANA---NNGCSYAQEAKRILTH-----------GKVC------------------------------------------------------------------------------------------------------------------------------------------------------------------------------------------------------------------------------------------------------------------------------------------------------------------------------------------------------------------------------------------------------------------------------------------------------------------------------------------------------------------------------------------------------------------------------------------------------------------------------------------------------------------------------------------------------------------------------------------------------------------------------------------------------------------------------------------------------------------------------------------------------------------------------------------------------------------------------------------------------------------------------------------------------------------------------------------------------------------------------------------------------------------------------------------------------------------------------------------------------------------------------------------------------------------------------------------------------------------------------------------------------------------------------------------------------------

>MR_Chit21

-------------------------------------------------------------------------------------------------------------------------------------------------------------------------------------------------------------------------------------------------------------------------------------------------------------------------------------------------------------------------------MSLSLG---------------------------LDV-----------------------------------------------------SGAAPALNAY--------W------------GQT----------GGRFLRDIC-------------------------DSGVNYATVSFINNSPE---NGDGYPGSNFGANCAGE--VYTNT-------DGKKTKLLSACSFIQRD------IPYCQS-K-GVKVLLAIGGA-HIPGTSEYAVSSVDK-GVEFA------EFLYNAFGP-YKSS--W-----KGPRPFD------SATEHVSVDGFDLDLEDRT-----PKFS-NKP-----------YIAMVDWWRQQ---------------------------SHKMFITAAPECVMFG-----------------N--QNDELIK----N-AEFDALFIQFYNNPV-------CDA-----------IPNNTPGDKFS--YDEWV--------AKIA--------------TGKSKEAKLFIGLPASTDSA-----GSG-Y--IDPEAMKNLVCKY----HNHKNFGGVSLWDATRAMNNE--DAEGKSFLQSAAEAVQY-------------VC-------GEPP--------KTTSS-------------APTSTKITSTTSSTTVKT-----------------------------------------SSTSSEKPTTSNTASE----------------KSATTSTSSASSSAKLT-------------------------TSSIQTGNVTTSSISSGK--------------PTS----------------------------------SSATS--------------EKLDTTSVIATAS--------------ATKSAI------------------------------------------------------------------------------------------------------------------------------------------------------------------------------------------------------------------------------------------------------------------------------------------------------------TSSLRMTS----------------------------------------------------------------------------------------------------------------------------------------SIHWSNSTGTRTVQTTSKPVAITTSKPVAITTSKPVSMTTSTVYTTSVHTV---------------------TECPPTVTDCPV--G-------------HVTTEIIALY------TTVCP-------VTE------------TAQPPKPT---------TTKAADMTTSTVYTTKTYTITECPPTVTDCPV-------GHVTTKVIP-LYTTVCPVSETAQPPKPT-----------TTKVPV-----------MTTSTVYTTKT-------YTITKCPPTVT-DCP--VGQVTTEVIPAYTTVCPVKETETGNVPKP----------TG--PAPSSA----------------ATKTTVVS------------------------KTVKVQQ-----------PSSLATSTRSAAVV---------------------PQPSGK----GCSGPSC--------PGATNAPGSGC----------------TGPECP----------------------------GVAIPTNASGSGCTGPECPGVAI---------------------------PTNSWTNKHSSGCTGPQC-SGVAIPTNAWT--------SSSVGP--SAVPSS--PVTAGAST--LALGL-TGL------VAIV--AAQVLAL-

>11212_S_sclerotiorum_B_II

MV--------------------------------------------------RA-------------------------------------------------------------------------------------------------------------------------------------------------------------------------------------------------------------------------------------------------------------------------------------------------------------------------LELMSNND---------------------------------------------------------------------------------------------------F-----------------------------------------------------------HIIAQIIPLAFLYSI-----NTPVLNFANQGDLCT----TISG-------------STLFHCSELEAD------ITTCQQTY-NKTILLSVGGA-TY---TEGGFTSTAA-AITAA------NNIWSWFGP-YSSG---------VVRPFG----------TAVVDGFDFDFEST-----------VSN-----------MPAFANQLRSLMDTD----------------------NSRTWLLSAAPQCPYPD-----------------A--ADGPMLN----GAVSFDIVWVQFYNN-Y-------CGVQSF--------VAGSSTQNNFN--FDTWD--------NWAKT-------------VSLNPNVKVMLGIPANTGA------GAG-Y--TTGSALASVIAYS----KSFSSFGGVMMWDMSQLYANA-------GFLDAINADLGNPAITVP-------------VT-------------TSTTT-------------STPP---YTTTTATTTT--------------------------------------------PGTTLT---------------------------TVTTTSATPTS--------------------------------------------------------------------------------------------------TGGS--------------GLVNEWNQCG--G-------------QGWNGGT--------------VCVPG------------------------------------------------------------------------------------------------------------------------------------------------------------------------------------------------------------------------------------------------TTCVAY---------------------------------------------------------------------------------------------------------------------------------------------------------------------------------------------------------------------------SICFLLHITIYYYYNPGSENFRLLLTNILQILNCCN--------I-------------RLLLETYTGNRF--------------------------------------------------------------------------------------LKRFLISRSKIEVQYSQIFPFAIKA---------FEFDFDFFDNYLPLLK-----------IEDVLLDRQRALKF-TLTIQNSLSFLK-SAFANRGSTAN-------KLWPIHIFSQ--SQTPDR--VLDSWKQDSLPSLRKAIYNHPDTF-------------LVS----------------------FVAALPALN-----------------------------G-------------LASDQEKNKE---------------TDPPDIKLYEIIEQ----------------LAPMEVCLLDLLF---------CVVSIALREGMGKVGKLKYGEASN---HICGGCCIVYN-------------------------------------------ACVAVACDARSA---------------------------------ATAGV----------IDVYR---------

>08695_S_sclerotiorum_B_II

------------------------------------------------------MYS--------------------------------------------------------------------------------------------------------------------------------------------------------------------------------------------------------------------------------------------------------------------------------------------------TRSLIGF-------------ASMLAS---------------------------LP-----------------------------------------------SAFAGFSTTATDNVAIY--------W------------GQGA---------NQGTLASYCS------------------------NTDFNIIPIAFLVSI-----NKLTVNVGNA------------------------------DPTQVGKD------IVTCQG-M-GKTILLSIGGA-TY---SDSELKTSDE-ATTAA------KNVWAAFGP-KTSS--------STTRPFG----------DAVVDGFDFDIETV---------G-LAN-----------LEVFAQELRSLSDAE----------------------TSKKYYLTAAPQCPYPD-----------------Q--ADKSFLQ----GEVSFDAVFVQFYNN-N-------CGLNKF--------VKGSSTQSVFN--MDTWD--------KWASG-------------TSKNRNVKVFVGIPGSSTAA-----TVG-Y--VDQDTMTDVIKYS----KTFKSFGGVMAWDMVTLIGNS-------GYLANINKALGGTPASGS-------------VAATTSKA-----TVTTAKAS----------STAVGS---TLTTKVRSTI-TSTKAI-----------------------------------ATATA-------TRAA---------------DGQSSVKSASASASAT------------------------------------------------------------------------------------------------LISSS--------------GTVPKWSQCG--G-------------EGYTGST--------------ECESG------------------------------------------------------------------------------------------------------------------------------------------------------------------------------------------------------------------------------------------------SKCVKT---------------------------------------------------------------------------------------------------------------------------------------------------------------------------------------------------------------------------DDWW----------------------------SACQ-----------------------------------------------------------------------------------------------------------------------------------------------------------------------------------------------------------------------------------------------------------------------------------------------------------------------------------------------------------------------------------------------------------------------------------------------------------------------------------------------------------------------------------------------------------------------------------------------------------------

>13155_S_sclerotiorum_B_I

------------------------------------------------------MAS-----------------------------------------------------------RD---------------LK----------------------------------------------------------------------------------------------------------------------------------------------------------------------------------------------------------------------------------------IGILVK---------------------------VDEY---------------------------------------------DSMENDEQCCEEKVERYRRVAPGLPW-----------IGSGP---------SQTNLSYYCE------------------------QSTIDIIPLAFMNVFPA---QGDGYPAENFGNACYGQPIFTPGPGYPLGDVDTSKDQLYVQCPGIQEG------IPYCQS-L-GKKILLSLGGA-S----NTYQLTGVAD-GEYFA------DFLWGSYGP-FKQS--WL--DNGGIRPMDGGYYGTDPNIHIDIDGFDFDIEIA------PTDS-SEG-----------YIAMINRLREHFAEN----------------------PSKKYFISGAPQCPLPE-----------------P--NMGAMIA----G-AKFDLLWIQFYNNDQ-----AQCTARQWAEN---YAITGQKDSEEFT--YDEWI--------ATVN--------------GGASAGASIYIGLLGSPLAGA----ATD-Y--ISPLEAQTLIESY----HAKPQFGGVMIWEATYSQENTDAELNGDSYHGFIKSCLSP---------------------YAPPPP-------TSISS-------------VVSSTIISSSSSTSEASITSSATLLIE------------------------------SSASASSTLTLISSSSIS----------------ISISESSAAASSTSSTY--------------------------SSQSSAAASPTSLEVPI--------------TLS----------------------------------STSSA--------------APTSTAQSFSSSG---SSAAQFSSASESTVSSTVSS-----------ILASPTQASSGYS---------------------------------------------------------------IPGYSQNATSTLTPSQSPSSASNSV-TTSLSSDSQSFEPSSTYTA-------------------------PAYS-----------------------FNSTI----------------SGSVYSTEPYST----------------KSVVTGTGESSKPRPTKSLGTGVKSCKSRT---KTRTKTSSSGPSGFASSSIIIPS--------TAPYGNTTYSQSSTSPASSS-----------------------TTSAEGITVSSGSAIQTGSISTKSTAIGSSTTAI---------------------GRVSSG----------------------------------------SVTSSGSLPSETGSLSASGSIPSGSG----SITSVVQYTTSTAYVTTVYTV---------------------TSCAATVTDCPARIG-------------SVTTETISSY------TTICP-------VTATAISSAEGVSVSPQSSGSPSSSQGLATSPITSVVKYTTSIAYVTSVYTVKSCAATVTDCPA-----RIGSVTTETIY-SYTTICPVTETGVDSGIAS----------STSAPAIAPTQTTAFTSYGTSTVYQTSV-------YTITSCAPSVT-DCPARSGQVTTETVSSYTSVYTITVSRA--------------------PVGVSALG----------ASSAAGIETL-------------------------IPVVGTSS--LAGISSPIPTSEFSSAAGIVTSL---------------------PVLSAS----PISGESSPA----ATPEAPPTYLYTTQISTLINSQTTTISAVIAPSTIT----------------VIPISI---ASTTFVPAYSSIAP---PYGAGNGTLTVSETLIT------TASSLSITFVPVPSSYVVKA-----------EGESTKSGSSKT----------QSAITSPTPST-ITFEGGAGRMIEKMGVCTGLFM----------MGLVFLV-

>05455_B_fuckeliana_B_II

------------------------------------------------------MPS--------------------------------------------------------------------------------------------------------------------------------------------------------------------------------------------------------------------------------------------------------------------------------------------------LSALAPL-------------AGIIAS---------------------------IP-----------------------------------------------SVMAGFDPTSQSNVAVY--------W-----------------------------------------------------------ATAFILLP---------------------------------------------------------KAD------ITTCQKTY-GKTIMLSVGGA-TY---TEGGFTSTQA-ATTAA------NNLWSWFGP-DTSG---------DIRPFG----------SAVIDGFDFDFEST-----------VSN-----------MPTFANQLRSLMDTD----------------------TTKTWLLSAAPQCPYPD-----------------A--ADGPMLD----GTVAFDIVWVQFYNN-Y-------CGVQSF--------VSSASIQNNFN--FDTWD--------NWAKT-------------VSLNPNVKVMLGIPSNTGA------GAG-Y--TSGAALASVIAYS----KSFSSFGGIMMWDMSQLYANP-------GFLDAVNADLGKPAVTVP-------------VTTTTITS-----TVTSTTT-------------TTTT---STTTSATTTS--------------------------------------------PGSTLT---------------------------TITTTSASPTS--------------------------------------------------------------------------------------------------TGGS---------------LVNEWNQCG--G-------------QGWNGGT--------------VCVAG------------------------------------------------------------------------------------------------------------------------------------------------------------------------------------------------------------------------------------------------TSCVAY---------------------------------------------------------------------------------------------------------------------------------------------------------------------------------------------------------------------------SIWY----------------------------SQCNPN---------------------------------------------------------------------------------------------------------------------------------------------------------------------------------------------------------------------------------------------------------------------------------------------------------------------------------------------------------------------------------------------------------------------------------------------------------------------------------------------------------------------------------------------------------------------------------------------------------------

>14944_B_fuckeliana_B_II

------------------------------------------------------MYS--------------------------------------------------------------------------------------------------------------------------------------------------------------------------------------------------------------------------------------------------------------------------------------------------TKSLIGF-------------ASMLAA---------------------------LP-----------------------------------------------SAIAGFSSSSSNSVAIY--------W------------GQGP---------NQGTLASYCS------------------------NAGFDIIPIAFLISL-----NKLTVNVGNA------------------------------DPAQVAKD------IVTCQG-L-GKTILLSIGGA-TY---TENELASADA-ATTAA------KNVWAAFGP-KTSS--------STTRPFG----------DAVVDGFDFDIETQ---------G-LTN-----------LDVFAQELRTLSDAE----------------------TSKKYYLTAAPQCPYPD-----------------Q--ADKSFLQ----GAVSFDAVFVQFYNN-N-------CGLNKF--------VKGSTTQSEFN--MATWD--------KWAST-------------TSKNKNVKVFVGIPGSTSAA-----TTG-Y--IDQATLTDLITYS----KTFKSFGGIMSWDMVTIIGNS-------GYLANINKALGGVPASGS-------------TGSSKTSA-----AATTAKA------------TAAPT---TLTTKVKSTV-VSSKVV-----------------------------------ATTTAKAAATTTAKAA---------------DDQTTVSSAVASATA----------------------------------------------------------------------------------------------------AS--------------GAVPQWSQCG--G-------------EGYTGST--------------VCASG------------------------------------------------------------------------------------------------------------------------------------------------------------------------------------------------------------------------------------------------FKCVKT---------------------------------------------------------------------------------------------------------------------------------------------------------------------------------------------------------------------------DDWW----------------------------SACE-----------------------------------------------------------------------------------------------------------------------------------------------------------------------------------------------------------------------------------------------------------------------------------------------------------------------------------------------------------------------------------------------------------------------------------------------------------------------------------------------------------------------------------------------------------------------------------------------------------------

>04500_N_crassa_B_II

------------------------------------------------------MYS--------------------------------------------------------------------------------------------------------------------------------------------------------------------------------------------------------------------------------------------------------------------------------------------------SSMLVAL-------------LALANS---------------------------LP-----------------------------------------------GALGGFSSTATGNIAIY--------W------------GQNSANQ-VG---GQQRLAYYCR------------------------NTNVNTIPLAFLNVI-----RDTEMNFSNAGDDCS----IFPG-------------TKLIKCTQIEED------IKTCQS-L-GKSILLSIGGA-TY---SEGGFSSPAE-AISWA------DRLWAMFGP-VSNS---------DKRPFG----------TAVIDGFDFDFEAV-----------AQN-----------MVPFATRLRSLMDSAA-------------------ATSGKKFLLSSAPQCPFPD-----------------R--ANNDLLQ----S-VAFDFVSVQFYNN-Y-------CGVHTF--------QFGSPSQNNFN--FGTWD--------NWAKT-------------VSKNKDVKILLGVPGSSSAA-----GTG-Y--IAGSQLANVIKYA----QTFSSFGGVMMWDMSQVWANA-------GFLDSVASALGSTGGSGVGITP--------TIETTSLPG-----STTTSST-------------PVVQ---TATTWVTVTI-WATATV-----------------------------------NGPAATAKSTSTTTLW------------------AESTTAISAP----------------------------------------------------------------------------------------------------TPAA--------------GLVNQWGQCG--G-------------NGYKGLT--------------SCRAP------------------------------------------------------------------------------------------------------------------------------------------------------------------------------------------------------------------------------------------------YSCVKL---------------------------------------------------------------------------------------------------------------------------------------------------------------------------------------------------------------------------SDWW----------------------------SHCN-----------------------------------------------------------------------------------------------------------------------------------------------------------------------------------------------------------------------------------------------------------------------------------------------------------------------------------------------------------------------------------------------------------------------------------------------------------------------------------------------------------------------------------------------------------------------------------------------------------------

>02184_N_crassa_B_I

------------------------------------------------------MH-----------------------------------------------------------------------------------------------------------------------------------------------------------------------------------------------------------------------------------------------------------------------------------------------------------------------KSLVAAA--------------------------VL-----------------------------------------------AATARAHTLTEAKVNVY--------W------------GQR----------GDARLRDHCD------------------------QANFDYVTI---------------------GAHCDAT--YYTNG--------TTSGHMNGKCSVVASD------IKHCQE-K-GKKVLLSLDGV-EH-MGSRFSLSSEAK-AEEFA------SFLWGAFGP-YDAK--W-----TGPRPFD------FAGHRVSVDGFNLDGELKL-----NGAG-EGA-----------YAAMAKKLRELYNGN------------------------AELLLTAAPGCSLDD-----------------V--KMKAIFD----N-AQFDALFIQFYNNPS-------CEA---------------ASASGFN--YLQWE--------KAIA--------------AGMSKEAKLFIGLAGASDAA-----GSG-Y--IEPLEAAALINTY----KTRSSFGGAMVLDAFRGQTLA----NGMTFLDVINTVVSSAEAV----DLSSEFCEDE----SALPKV---PSVTEGSAGGFFTVADPSAITSGPVVLPSGGSSVLPTGGSSEDEVCED------------------------------ENVIPKVPSVTDGAHEVNPTI-----------VDTSIITSVPVALPSGDRSEIIGGSPATEDDVCEGEIMTEDPLRSGVVPSGVMPTGALPSGVLPSGDRSDIIGAIPSGSIPLGSAPSGLVPSGSIPLGSAPSGLVPSGSIPLGSAPSGLVPSGAVPSGSIPLGSVPSGDRSDIIGAIPSGSIPLGSVPSGVVPSGDRSEIIGSPADEDDVCEGEDPVRSGLVPSGAVPTGDRSDIIGAIPSGSIPLGSAPSGLVPSGAVPSGDRSEIIGAIPSGSIPLGSVPSGAVPSGDRSEIIGAIPSGSIPLGSAPSGAVPSGLVPSGIVPSGAVPSGDRSEIIGSPADEDDYCEGEITPEDPVRSGLVPSGIVPTGAVPTGLVPSGGVIGSTVPGGVIPSGVAPGGILPSGVVSSVGSKVTGPIDGDNVALPSVGLPSGGVIASGVVPSGVVPSGVVSSIGSKVTGPIDGDNVALPSVGLPS-GAIASGVIPSGVIP-SGIVPSGVVSSIGSKVTGPIDGDNVAL-------------PSVG-LPSGDLPSGVVPSGVVPSGVLSSVGSKITGPIN----------GHNVAVPSAGVPSGVVPSGILSNVG----------SKVTGPIDGNNVVPTGILPSGSVPSGVVSSVVSMVTADPINGDKIADPS-----AVTAPAEWTTSTIYATTTSTI---------------------TSCAPEVTDCPAKIG-------------QVTTVTVPIG------VTVCP-------VTA------------TETAARAPTGIFTSVPAESIPAGFTTSTVYSTTTSTIASCAPEMTDCAG-----KIGQVTTVIVP-VGVTVCPVTATETAATETAARAP---TGIMTSVPVES-----IPAGYTTSTVYSTAT-------STIASCAPEMT-DCAGKIGQVTTIIVPVGVTVCPITEAFPPATSVPAAPAAV-PTGAASVPAVSAVPT------------GGAGVPAVSA------------------------PAVPTGGAGVPAVSTPVVPSGVAGVPAVSAPA-VPSG--------------GADVPAVSAPAVPSGGADVPAVPSNGAPGAGVNKVATS----------SVVSVSSMPFTTI---------------------------TVAKPAASAPGA---PGAPGAGVPA-ESAAVPAGGAPAVPSAVNAPVV--PSGAV----------PTG-TGVSVAPSSAASTYSMPAPPAQTEPVTGSEPSE-VPVTAGAGRNVVAMGV-PALV-----------AALVLAL-

>MGG_01333_M_grisea_B_II

--------------------------------------------------------M--------------------------------------------------------------------------------------------------------------------------------------------------------------------------------------------------------------------------------------------------------------------------------------------------LRRASVL-------------PLAMAI---------------------------FG-----------------------------------------------TAQAGFNPSMQDNIAAY--------W-----------------------------------------------------------DVNVDIIPLAFLNIIV----NPTNINFANVGDRCS----KFPG-------------TDLLKCPEIEAD------INTCHS-L-NKTILLSVGGA-TY---SEGGFPNVEA-ANKAA------DNLWAMFGP-PPAA--STNDAATVNRPFG----------TAYIDGFDLDFETH---------G-IKN-----------LAAFARRLRQVMDTAARPAAVLPSDDKSYPMVPSPSEATKKFYLAAAPQCVFPD-----------------A--AMDAALS----SDVAFDFIMVQFYNN-Y-------CGLQNF--------QPGAAQQNAFN--FERWD--------QWAR---------------EKGRSTKVMLGVPGSPNAS-----GSG-Y--TAAGPLDAIIKYC----KRFSSFGGVMIWDASQVWSNN-------GFLDGVVRSLGGAAAT------------------------------ETSDA-------------EAPQ---QAGRPIQQT---------------------------------------------------------------------------------------------------------------------------------------------------------------------------------------------RA--------------VKVPKFGRCG--G-------------RLYTGPR--------------ECEQG------------------------------------------------------------------------------------------------------------------------------------------------------------------------------------------------------------------------------------------------SKCVYG---------------------------------------------------------------------------------------------------------------------------------------------------------------------------------------------------------------------------GALW----------------------------SVCA-----------------------------------------------------------------------------------------------------------------------------------------------------------------------------------------------------------------------------------------------------------------------------------------------------------------------------------------------------------------------------------------------------------------------------------------------------------------------------------------------------------------------------------------------------------------------------------------------------------------

>MGG_03599_M_grisea_B_I

------------------------------------------------------MKV----------------------------------------------------------------------------------------------------------------------------------------------------------------------------------------------------------------------------------------------------------------------------------------------------SGSNLT------------GALLAC---------------------------CAL----------------------------------------------ASAARYSPNVKTNTVVY--------Y------------GQGP---------DQKDLMTYCR------------------------EPAIDVIVLSFVHLFPQ---QANGLPGTNFGNQCGGA--VYPGP-----GPDPARDALQANCPRLVPQ------IEQCQR-IFRKKILLSLGGG-V----AGYQLTGADA-GRAFA------DQLWAIFGP-RPDG--S-----TLPRPFD------GESRVADLDGFDLDIEFP------PVDG-GEG-----------YRALALRLRAHYASVP--------------------GRRKIRLLTASPQCVVPD-----------------S--NLSDVIR----A-VRFDALFIQFYNTIV-------CSAARWANENPTYTPGDIANPAGFT--FDAWT--------ESIR--------------GTASSRAKLYLGLAGGPNAA-----NPGHY--IDESASRRLVEAF----FCRPNLGGVAIWEATNANPGG-----KKAYYTTAREHLERANRD-----PKAREC-----LVAPPP--------DNSNAP------------PAPAT---------------------------------------------------------------------------------------------------------------------------------------------------------------------------------------------------------------------------------GTCG-----------SGVGSCAGD--------------LCCSQ-----------------------------------------------------------------------------------------------------------------------------------------------------------------------------------------------------------------------------------------------YGYCGQEP----------------------------------------------------------------------------------------------------------------------------------------------------------------------------------------------------------------------------------------------------------EYCGAGC----------------------------------QWQFGTCT--------------------------------------------------------------------------------------------------------------------------------------------------------------------------------------------------------------------------------------------------------------------------------------------------------------------------------------------------------------------------------------------------------------------------------------------------------------------------------------------------------------------------------------------------------------------

>AN8241_E_nidulans_B_I

------------------------------------------------------MAP----------------------------------------------------------------------------------------------------------------------------------------------------------------------------------------------------------------------------------------------------------------------------------------------------KLFTF-------------VSALSG---------------------------LA-----------------------------------------------SLASAFHAEAKSNIAVY--------Y------------GQGV---------NQPRLAEFCA------------------------ETSYDIINIGFINSFPEQN-PLTGLPGSDFGNQCWAD--TFVV--------DGIASQLYSHCPNIAED------IPKCQA-A-GKKVFLSLGGA-T----PTYWFDTIDA-STKLA------DFLWGAFGP-VTDA--WT--VADKPRPFG----------NAVVDGFDFDIEFF---------G-SKG-----------YANMIKRFRRRFGEV----------------------PDQTFYISAAPQCSIPD-----------------E--QLSVAIK----N-AVIDFVWVQFYNTPG-------CSARDFV----------LGTKNGFN--YDSWV--------EVIK--------------AGANPNAKLYVGLPASGAAA-----NLGYY--LTPEEVKPLVKKY--MDKYPETFGGVMLWEATQARNNQ---IDGVGYNEKIREILYD------------------LDPNHPPP--------TTSPTP-----------TPTPS---TTTTSTTSTTSTTSATS---------------------------------TTSTTSTTSTTSTTPTTSTT------------STTSTTTPTPSPSPSTASSSTT-------------ETVTPSPKPSPSESSTTSETSSLPSTSTP------VVSETPSET----------------------------------KTPTS--------------SSAPPLSSSSPVG------------GSSSTASS--------------STSTPSETPSASSTRAVSETSTHI----------------------------------------------------------STSTSSGPETSLTGSSTSVPATSSSVPSSAISPSST--------------------------------------------------------------------------------------------------------------------PVISETPRPPVTSSSSSTFVSSTSTSTDCS-----ESSTAIGTH--------SSSSISETPSASTPAASPSTSPE--------------------TTKTLTVFPTPGSSVSTGTTSASTLSSSVPAT---------------------------------------------------------------------SGGHTETSTVSTSSANQTPS----------ASTSKPLIPTNSASSTSTGSV-------------------TSTPSAPGVPSSSA--GSD----------ETATTSTTDSE-----PTSTSS-----GSVTA------------KPTTTEPAT--------------------------------------------------TTTIIVTSYTSICPTGFTTITTTITS-----------TYCPGTA-------SATATAIAPTTDVPGS---GSGSSPAQPTITADIP--EGWTTTVTVCTVCAATPTTVTLT----LP--------------PATTTEES---------------------------------------------TSAQPTGE-------VPSSDGSGSGEVSTTTVVV-------------------VPAPTGN------AGDGVP------APGANVGEEYT-----------------AAPGSA----------------------------TTSKPLIGGGAS-------GAHTAY-----------PYASSTFHI----IPSAS---------------AHVPVPSGSGS---------SPSGTQGGASP----TFTGAGSRYDVVKGV-PAL------VALA--LSLLAVL-

>07348_C_immitis_B_II

------------------------------------------------------MYC--------------------------------------------------------------------------------------------------------------------------------------------------------------------------------------------------------------------------------------------------------------------------------------------------LR--TAL-------------LPVIGL---------------------------FN-----------------------------------------------AAYARLDTDSASNLAVY--------WAFEGHLLRQYRVGQNSYNQ-GSGNLTQQPLGYYCE----------------------------------MINGIG----GAPQVNFANQGDNCT----TFPG-------------TDLLNCPQIGAD------ITKCQG-K-GKTIILSIGGA-TY---SEGGFGSEEE-AIAGA------NLIWETFGP-QKNS--------SRPRPFG----------DATVDGFDLDFEA----------T-VRN-----------MVPFANRLRSLMAAD----------------------ASKKYFLTVAPQCPYPD-----------------L--YNKEMLE----GKVDFDAVFVQFYNN-F-------CGLNTF--------EPGQEEQKSFN--LGEWN--------EWAKT-------------VSKNKKVKVIVGAPANQRAA-----GSG-Y--VDASKLAEIVKYS----RKFSSFGGVMLWDASQAYANG-------NFIETVKGALNS------------------------------------------------------------------------------------------------------------------------------------------------------------------------------------------------------------------------------------------------------------------------------------------------------------GFAG-----------------CRFG------------------------------------------------------------------------------------------------------------------------------------------------------------------------------------------------------------------------------------------------------------------------------------------------------------------------------------------------------------------------------------------------------------------------------------------------------------------------------------W----------------------------RRTQ----------------------------------------------------------------------------------------------------------------------------------------------------------------------------------------------------------------------------------------------------------------MPMAR--------------------------------------------------------------------------------------------------------------------------------PVV---------------------------------------------------------------------------------------------------------------------------------------------------------------------------------------------------------

>Chi18-17_H_jecorina_B_II

------------------------------------------------------MPS--------------------------------------------------------------------------------------------------------------------------------------------------------------------------------------------------------------------------------------------------------------------------------------------------ISSFTSA-------------LGLLSL---------------------------LP-----------------------------------------------AARAGWNQNSNDNIVVY--------W------------GQNS------GSVGQNRLSYYCQN-----------------------APDVDVINISFMVGI-----TNLNLNLANVGNNCT----AFPQ------------APNLLNCPQVAAD------IIECQQTY-GKTIMMSLFGS-TY---SESGFSSSSA-AVAAA------QEIWAMYGP-VQSG--N-----STPRPFG----------DAVVDGFDFDLEDP---------I-ENN-----------MEPFAAELRSLIDAS----------------------TSKKFYLSAAPQCPYPD-----------------V--SDESFLD----GQVPFDWVNVQFYNN-G-------CGVSHY--------------PADFN--WATWD--------NWAKT-------------VSANKNAKVLIGTPANVGGA-----NAGSF--PTDSQLSGAISLA----KGSSSFGGVMLWDMAQLFTNQ-------GYLAKIVADLGS------------------------------------ASS-------------PPPP---ASTTLQTIT------------------------------------------------------------------------------RSSTASTGPTN--------------------------------------------------------------------------------------------------PPSG--------------GSVPQWGQCG--G-------------NGYTGPT--------------QCQAP------------------------------------------------------------------------------------------------------------------------------------------------------------------------------------------------------------------------------------------------YKCVAT---------------------------------------------------------------------------------------------------------------------------------------------------------------------------------------------------------------------------SEWW----------------------------SSCQ-----------------------------------------------------------------------------------------------------------------------------------------------------------------------------------------------------------------------------------------------------------------------------------------------------------------------------------------------------------------------------------------------------------------------------------------------------------------------------------------------------------------------------------------------------------------------------------------------------------------

>Chi18-12_H_jecorina_B_II

-----------------------------------------------------------------------------------------------------------------------------------------------------------------------------------------------------------------------------------------------------------------------------------------------------------------------------------------------------------MPSLTAL-------------AGLLAL---------------------------VP-----------------------------------------------SALAGWNPDSKQNIAVY--------W------------GQNSANS-QS---TQQRLSFYCN------------------------DDNINVIEIAFLNGI-----NPPMTNFANAGDRCT----PFSD------------NPWLLSCPEIEAD------IKTCQA-N-GKTILLSLGGD-TY---SQGGWASPEA-AQDAA------AQVWAMFGP-VQSD--S-----SAPRPFG----------DAVVDGFDFDFEST-----------TNN-----------LVAFGAQLRTLSDAAA-------------------TDSNKKFYLAAAPQCFFPD-----------------A--AVGPLIN----A-VPMDWIQIQFYNN-P-------CGVSAY--------TPGSEQQNNYN--YQTWE--------DWAK--------------TSPNPNVKLLVGIPAGPNA------GHG-Y--VSDAQLKSVFEYS----KKFDTFAGAMMWDMSQLYQNS-------GFEDQVVDALK----------------------------------------------------------------------------------------------------------------------------------------------------------------------------------------------------------------------------------------------------------------------------------------------------------------------------------------------------------------------------------------------------------------------------------------------------------------------------------------------------------------------------------------------------------------------------------------------------------------------------------------------------------------------------------------------------------------------------------------------------------------------------------------------------------------------------------------------------------------------------------------------------------------------------------------------------------------------------------------------------------------------------------------------------------------------------------------------------------------------------------------------------------------------------------------------------------------------------------------------------------------------------------------------------------------------------------------------------------------------------------------------------------------------------------------------------------------------------

>Chi18-14_H_jecorina_B_I

------------------------------------------------------MFF------------------------------------------------------------------------------------------------------------------------------------------------------------------------------------------------------------------------------------------------------------------------------------------------TKAVGGLGLL------------ASLASS---------------------------AP------------------------------------------------NPIARRQAPGAQNVVY--------W------------GQNG-----GGTVENNDLSAYCTP-----------------------TSGIDIIVLSFLYQWG----QGSSALGGTIGQSCGIT-----------------TSGEPQNCDALTAA------ITKCKT-A-GVKIILSLGGA-S----AFSSFQTADQ-AAQAG------QYLWNAYG---GGS--------GVTRPLG----------NNVMDGFDLDIESN--------PGTNEN-----------YAALVSALRSNFASD----------------------PSRQYVISGAPQCPLPE-----------------P--NMGVIIQ----N-AQFDYLWVQFYNNNE--YPGDPCSL-------------GLPGDAPFN--FNNWT--------TFIQ--------------STPSKDAKVFVGVPAAPLAANGAPSGEVYY--ATPSQLADIVNDV----KSNPAFGGIMMWSAGFSDTNV---NDGCNYAQEAKNILLT-----------GSPCSSGPVSVSRPPV-------SSPTI------------TSSPPG--TSPAPPSQT-------------------------------------------------------------------------------------------------------------------------------------------------------------------------------------------------------------GSVPQWGQCG--G-------------NGYTGPT--------------QCVAP------------------------------------------------------------------------------------------------------------------------------------------------------------------------------------------------------------------------------------------------FKCVAT---------------------------------------------------------------------------------------------------------------------------------------------------------------------------------------------------------------------------SEWW----------------------------SQCE-----------------------------------------------------------------------------------------------------------------------------------------------------------------------------------------------------------------------------------------------------------------------------------------------------------------------------------------------------------------------------------------------------------------------------------------------------------------------------------------------------------------------------------------------------------------------------------------------------------------

>Chi18-16_H_jecorina_B_I

------------------------------------------------------MLS------------------------------------------------------------------------------------------------------------------------------------------------------------------------------------------------------------------------------------------------------------------------------------------------RTLLTALGL-------------TTIAAA---------------------------AP-----------------------------------------------SQTVKTRQAPGGQNAVY--------W------------GATN-----N---ENDNLSTYCTA-----------------------SSGIDIVILSFLDIYG----ATGNFPSGNMGNSCYVG-----------------TNGVPQLCDDLASS------IATCQA-A-GIKVIISLGGA-A----SSYSLQSQSQ-AVAIG------QYLWNAYGN-SGNT--------TVQRPFG----------NVFVNGFDFDIELN--------AG-SQY-----------YQYLISTLRSNFAND----------------------PKNTYYITGAPQCPIPE-----------------P--NMGEIIS----T-SQFDYLWVQFYNNNP--V----CSL-------------GLPGDAPFN--FNDWV--------SFIS--------------TTPSKNAKLFVGAPASTLGANGNAGGAKYY--ATPEQLAGIVNSV----KSSPFFGGIMLWDAGYSDSNV---NNGCNYAQEAKNILLT-----------GTACGGE----SSPPP-------STTTT------------AVPPPA--SSTPS-----------------------------------------------------------------------------------------------------------------------------------------------------------------------------------------------NPSG--------------GSVPQWGQCG--G-------------DGYTGPT--------------QCVAP------------------------------------------------------------------------------------------------------------------------------------------------------------------------------------------------------------------------------------------------YKCVAT---------------------------------------------------------------------------------------------------------------------------------------------------------------------------------------------------------------------------SEWW----------------------------SSCQ-----------------------------------------------------------------------------------------------------------------------------------------------------------------------------------------------------------------------------------------------------------------------------------------------------------------------------------------------------------------------------------------------------------------------------------------------------------------------------------------------------------------------------------------------------------------------------------------------------------------

>Chi18-13_H_jecorina_B

------------------------------------------------------MFF---------------------------------------------------------------------------------------------------------------------------------------------------------------------------------------------------------------------------------------------------------------------------------------------------SKALAA-------------AGLLAT---------------------------AAY----------------------------------------------AAPTMEKRAAGGKLVVY--------W------------GAED---------DSTTLANVCA------------------------DSSYDIVNLAFLSRFF----AGGGYPELSLST--------LGGPS---AAQRAAGATNLQDGTSLIPA------IQACQA-A-GKLVILSMGGA-VD--FSAVTLSSDAQ-GQQLA------DTVWNLFLG-GTAN--------PTLRPFG----------SVKLDGVDLDNETG---------N-PTG-----------YLAMAQRFKSNFAKD----------------------TSKKYYLTAAPQCPFPD-----------------A----SEPLN----VCQLADYIWVQFYNNGN-------CNI----------------AQSGFNNAVKNWS--------------------------KSIG-NATLFIGALASGADG-----DQG-Y--VSASSLLSAYQGV--SALNLPNIGGIMLWEAQLAVKNG-------NFQKTVKAGI-----------------------------------------------------------------------------------------------------------------------------------------------------------------------------------------------------------------------------------------------------------------------------------------------------------------------------------------------------------------------------------------------------------------------------------------------------------------------------------------------------------------------------------------------------------------------------------------------------------------------------------------------------------------------------------------------------------------------------------------------------------------------------------------------------------------------------------------------------------------------------------------------------------------------------------------------------------------------------------------------------------------------------------------------------------------------------------------------------------------------------------------------------------------------------------------------------------------------------------------------------------------------------------------------------------------------------------------------------------------------------------------------------------------------------------------------------------------------------

>08680_P_nodorum_B_II

------------------------------------------------------MH-----------------------------------------------------------------------------------------------------------------------------------------------------------------------------------------------------------------------------------------------------------------------------------------------------STTIL-------------HTLLFI---------------------------IG-----------------------------------------------HAFAAYDNSSKTNLAVY--------W------------GQNSLKLSEPDPNAQGRLSKYCA------------------------DPDVDIIMIAFISALN----NAKGEIEINLANQLWY-----------------AQGGQPYPAPKTGAD------IKTCQT-TNQKTILLSFGGAETA---ADKGYASDDE-AKAGA------KKIWEMFGP-KTAA--------SSIRPFD----------DAVVDGFDFDFEDET-----AVFA-SRH-----------RDAFIKELSSLARAAE-------------------TAGGKKFYFSAAPECT-----------------------QKNSLLS----S-IKYDMVFVQFYNNPT-------CDVRKY----------QQSESTNTF--FNQWD--------EIAK-----------------SNGTKFFVGLPGFSSAA-----TNGGYSGLGPNDMYH------------KKVNTALLYVAFEINNQG------------------------------------------------------------------------------------------------------------------------------------------------------------------------------------------------------------------------------------------------------------------------------------------------------------------------------------------------------------------------------------------------------------------------------------------------------------------------------------------------------------------------------------------------------------------------------------------------------------------------------------------------------------------------------------------------------------------------------------------------------------------------------------------------------------------------------------------------------------------------------------------------------------------------------------------------------------------------------------I---------------------------------------------------------------------------------------------------------------------------------------------------------------------------------------------------------------------------------------------------------------------------------------------------------------------------------------------------------------------------------------------------------------------------------------------------------------------------------------------------------

>00864_P_nodorum_B_I

--------------------------------------------------------------------------------------------------------------------------------------------------------------------------------------------------------------------------------------------------------------------------------------------------------------------------------------------------------------------------------------------------------------------------------------------------------------------------------MY--------W------------GQGN---------DQYPLSVACA------------------------DPAIDVVTLGFVNGFPS---KEGEYPATNFANACWGT--YYPDP-----FDSEKKSGLLKDCPSINEG------IAVCRK-N-NKKVLLSLGGG-LP---TNYYLPDEKT-TTWFA------KFLVGAFGP-KQDG--W-----TGPRPIG----------DEFVDGFDLDLEAEASKVPRPELI-SAN-----------YGFLVNELKRF---------------------------NNDFLITAAPQCEVPD-----------------I--RLFDAIK----N-AHFDMIFTQFYNTPK-------CSARQGFKE---LTPKKGDPPSTFT--FENWA--------VWLSE-------------NSKNKAVKLYLGLPASPKGS---PSYQDHY--LKPNEANDLVAKWRFAQKTSPFFGGIMLWEHKVSADNV---IDCKNYGAWMKDILSSKFGS-----TWTKGCVK-----------------TSSSA------------TVASSTRLSSTRASSTRLSSTRASST-------------------------------RASSTRASSTRASSTPASSTLA-----------SSTRVSSSSASSTPASSSIASSIPS-------------------SSSIASSTSASSSIASSI-------------PSSS----------------------------------SIASST------------SASSSVASSISASS----------SIASSTAASS--------------TAASSTESS-------------------------------------------------------------------------SSIASSTPASSAQGSSTIASSSTVSTTSAAVSPTATMP--------------------------------------------------------TVDGHC--------GDQGHGIVYTCHGYNNGNCCS----------QYGYCGGLGDAGAAYCGEGCNPLFGECDNTSQSSSVASSTSVASSS-VASSSTVESS--------SATESSSTVEASSTVASSSSVAS--------------------SSAIASSSVASSSIVSSTATSASSSASVS------------------------------------------------------------------------SSSYSETTPVATSSASQSSVIESSTL----SVPYPSGNITYTPYPTGSASIVY--P------------EDVSKSLYPHSSQT-----------------LTYSSQSGVHYPA-GNATTPCT------TSTA------------APSSSSPAPM-------------YGASSSAPAPVYG-GQYPPSVTSMPGQNYPSYPASSTVKVITTTYVDACETGITTKTETITQ--------TVCNKCKGDS----------------VTSV-------YVCNKCGPAVV------------------------TYTIT----KPAS------------PATDV-------------------------------------------------PVVPTM-----------------------------------------------PVQPSS----PSHGGDKP-----QSPAKESTPSYGG----------------ERPHNT--------------------------------PTYEVPKK---QEVPGASTTV-EIVYITKTPIPMAPTVQHTPAMPYPSAPVQNGTSGYPVKPSG-TGVATPTKPS-----------------SYTPPA--QFTGAASH--LSAGM-AGL------LAVV---AGLFVL-

>EAA72615_G_zeae_B_I

------------------------------------------------------MDR--------------------------------------------SP------------------------------------------------------------------------------------------------------------------------------------------------------------------------------------------------------------------------------------------------------------------------VPVTGA-------------------------PVIQ--------------------------------------------------------AFGGLNGY--------W------------GQL----------GSEPLKAYC-------------------------DSHPEYITLSFVNQAPEQ--NPSNLPGTNFAGHCAGG--TYGN------------SNLLSECYTIQEG------IPYCKD-R-GVKILLSIGGV-YSDEGSNYKVTTDDK-GRDFA------DFLYKSFGP-HNEH--S-----NPIRPFDSID-NDGNTVHAAVDGFDFDIEHD--------LP-NGP-----------YIAMINRLREL---------------------------DEGLTITGAPQCPTSD-----------------EYFYMKDMIK----S-AKFDALFVQFYNNPW-------CDA-----------VSQGHEGESFN--YDKWV--------EIIEE-------------SDCSKDAKLYVGLPASEEAAP----GGG-Y--LEPEDLKDLVCEL----TTKPHFGGVSLWDLTRGAGNI---IEGKSYNEHVMDALKY-------------GC-----DLVSVP--------TTTTS--------------------AMTTTTEEAS-----------------------------------------TSTNITTTEATTVHTTT----------------TSDVSTTSGASSTEDAT--------------------------STDITTASDVSSMEEAT--------------ATS----------------------------------DVSAS--------------ASTDATTSMDATS-------------TDTAAST--------------DTAASTEAVASTYTTAATNSDAST-------------------------------------------------SADVATSMDAATSTDATASADATASTDV-TTSSDASVSTDATAST----------------------------------------------------------------------------------------------------------ETTATGANTSSVVTDPAASGVSATATE---SSADTATDSTTDS--ASKSDVLSA--------TSDLTVTSDTSSVTGAASSSTIDA-------------PVESDTATGTAATTNVSGATEPTTIATAYSTYRGWNTT--------STGAAAYTQAYGNPTTLAHSGSLTTEG----------------------------------SPAYTN--------------------Y--PGNTGAVSMTTSTVCTTRVHTV---------------------TQCPPEVVDCLF--G-------------SVTTETIPLY------TTVCP-------VTD------------KAKHTGYATQ-------YAPPPQYETRTVYTTSVHAINKCAPGVVDCPY-------GSVTTETIP-VYTTICPVTETYNQA--------------PTDVPV----------NHEIKTLYTSQA-------HTVAQCQPENP-NCQ--VGAVTTEIASWTTAIVPAHETQLMKAYKP----------AAELPKPLTV------------------------------------------------------------------ESHLNGTVYTSVVV---------------------P------------------------PATLKTA--------------------TKPGVF----------------------------EQARPTYKQTAQ-----------------------------EQH-------------------------SGAAAPTGGR----------NDKGPIETYAPAPITPATAGASS--MVVGL-TAL------AGIA--LLQVVALW

>Chi18-18_H_jecorina_B_I

------------------------------------------------------M------------------------------------------------------------------------------------------------------------------------------------------------------------------------------------------------------------------------------------------------------------------------------------------------------------------------VSASAG---------------------------LA--------------------------------------------------------AVGLLNGY--------W------------GQYT---------TTEGLRPHC-------------------------DSGVDSITLGFVNGAP----DASGYPSLNFGPNCWAE--SYPGN-----L--GLPSKLLSHCMSLQSD------IPYCRS-K-GVKVILSIGGV-YNALTSNYFVGDNGT-ATDFA------TFLYNAFGP-YNAS--Y-----TGPRPFDDI----TTGLPTSVDGFDFDIEAD--------FP-NGP-----------YIKMIETFRSL---------------------------DSSMLITGAPQCPTNP-----------------QYFVMKDMIQ----Q-AAFDKLFIQFYNNPV-------CDA-----------IPGNTAGDKFN--YDDWE--------AVIAG-------------SAKSKSAKLYIGLPAIQEPN-----ESG-Y--IDPIAMKNLVCQY----KDRPHFGGLSLWDLSRGLVNN---INGTSFNQWALDALQY-------------GC-------NPIP--------TTTTT--------------------TSTVSSTTAA-----------------------------------------SSTTASSTTASTTKASS----------------TSKASSTSKASSTSKAS-------------------------STSKASSTSKASSTSKAS--------------STS----------------------------------KASTT--------------SKASTTSKVSTTS----KASSTSKASSSTKAST--------------TSKASSTSKASTTSKASTTSKAST-------------------------------------------------TSKASTTSKASTTSKASSTSKASSSTKA-STTSKASSTSKASTTS--------------------------------------------------------------------------------------------------------KASTTSKASTTSKASTTSKASTTSKASST---SKASSSTKASTTS-KASSTSKAST--------TSKASTTSKASTTSKASTTSKAST-------------TSKASTTSKASTTSKASTTSKASTTSKASTTSKASTTSKASTTSKVSTTSKASTTSKASTTSKASSTSKVSTTSKASTTSKVSAKATTSTKASTTVKPSTTSKASTTSKASTTSKASTTSKASTTSKASTTSK----ASTTSKASTTSKAATTSVKPTS-----------------KTSTSSKPNVSASSSNVGRDAT----SLVEASTSTSAAVLY-----PTTTSR--WSNSTITR------------SSSLTTPI---------VSDPASLTTSVVYTTSVHTVTKCPAYVTDCPAG------GYVTTETIP-LYTTVCPISEATQTAAPTV----------TTEAPQ----------PWTTSTVYTTRV-------YTITSCAPGVV-DCP--ANQVTTETIPWYTTVCPVTATAT-----P----------VG--PGSVVFPQ-----------NTEVGQPSLVG------------------------PVVEAAY--------PTASSSLQTLVKPATSV---------------------GVPQGSP-----AGSSV-------APGSSSKP--------------------TAPAGP--------------------------------PSY----------------------------------------------------------PTGGSGNASPSGSWS--------GVPVGP--SSVPGI--PEANAASV--MSASL-FGL------VIVM--AAQVFVL-

>04523_R_oryzae_B

------------------------------------------------------MI-----------------------------------------------------------------------------------------------------------------------------------------------------------------------------------------------------------------------------------------------------------------------------------------------------SCNILG------------ITIAAF---------------------------IT------------------------------------------------STLAAYSSNGVNVMYY--------W------------GQNS----AGGSNTQGSLVSYCQ------------------------SGQVDVIILSFLNKF-----NMGGLPEINLASACEQT--FFPN-------------TNLLHCPTVGSD------IKTCQS-N-GVKVLLSLGGA-A----GSYGFSSDSE-GQTFA------ETIWNLFGG-GTSD----------TRPFD----------DAVIDGIDLDIEGG---------S-STG-----------YAAFVTALRSK----------------------------GHFLIGAAPQCPFPD-----------------A--ILGSVID----A-VGLDFVNVQFYNN-V-------CSV---------------ASGSSFN--FDVWN--------DWAKN-------------KSPNKNIKVMLTVPGSSTAA-----GSG-Y--ASIAELGPIVSSV---ISQYSSFGGVSVWDASQAWNNN-------GFHSELYSIVHG----------------------------------SSAALG-----------QAARKTPFPNTSSITTTS----------------------------------------------------------------------------LATASSALFPL---------------------------------------------------------------------------------------------------LYAG--------------RSCSSQSKVS--------------------------------------CTSTG---------------------------------------------------------------------------------------------------------------------------------------------------------------------------------------------------------------------------------------------SYTICNYGK-----------------------------------------------------------------------------------------------------------------------------------------------------------------------------------------------------------------------------WVT--------------------------APCPPGVICLSS----------------------------------------------A------------QSNNTD-----------LTVSAQFFITSFAGGSF--------------------------KAIINARRTTLTPFEKMKTIEFTVP------------------------------DHVRLSQCNL-----GKIEQVGRSV---------RIRLKDHPN--MAFVLNLSGN--------------------VSSDAFVA----------------------------------------------P----------------------------------------------------------------------------DPSSWRFS-----------------------------------------------------------------------------------------------------------------------------------------------------------------------------------------------------

>11152_R_oryzae_B

------------------------------------------------------M--------------------------------------------------------------------------------------------------------------------------------------------------------------------------------------------------------------------------------------------------------------------------------------------------------FILG------------MVIAGL---------------------------LKA----------------------------------------------LHVQAAYSSNGPNVMYY--------W------------GQNS----AGGATTQGTLSSYCQ------------------------SGKADIILLSFLHIF-----NLGGLPQINLSNACENT--FFPN-------------SQLLSCPAIGSD------IKTCQA-K-GVKILLSLGGA-T----GAYGFTSDAE-GQQFA------ETLWNLFGR-GSSE----------TRPFG----------DAIIDGIDLDIEGG---------S-SGG-----------YAALVTALRSKS-------------------------ASQDFLIGAAPQCPFPD-----------------A--LLGPVID----A-VGLDYVNVQFYNN-Y-------CSA----------------LGASFN--FDVWD--------TWAKT-------------QSANKQIKVYLTLPGSPRAA-----GSG-Y--VDMTSLSRLVPSV---ASRYASYGGVSVWDASQAWNNP-------GFPSSLYDLVHG----------------------------------SAPAL--------------------------TTST----------------------------------------------------------------------------ASRSTSSPMPT---------------------------------------------------------------------------------------------------VPSS--------------GACTSDQACSVPG--------------------Q------------YICTPHG---------------------------------------------------------------------------------------------------------------------------------------------------------------------------------------------------------------------------------------------AYAFCVHGQ-----------------------------------------------------------------------------------------------------------------------------------------------------------------------------------------------------------------------------WLT--------------------------ASCPPGTVCIPTVDG-------------------ASIYCGYGTPGSSCV---HGLNASS------------VALNTQPVPR---PYRLSNTHVQLVITSFSDHVF--------------------------EAMIHARRTTLFPFHPTITLELNVS------------------------------DQVQLDPSKG-------VQQKDKTV---------RVKVINPSNQTMALVLHLTGS--------------------VSSDVFVA----------------------------------------------------------------------------------------------------------------------------PASLRFN-----------------------------------------------------------------------------------------------------------------------------------------------------------------------------------------------------

>10112_R_oryzae_B

------------------------------------------------------MNH---------------------------------------------------------------------------------------W----------------------------------------------------------------------------------------------------------------------------------------------------------------FI------------------------------------------------------------ITFLCIL---------------------------------------------------------------------------LYKAQASFLDSPSIATY--------W------------GQNS----KGGSDTQHSLATYC-------------------------DGNSDVIILAFVLDF-----RNKELPQLNLANSCDGP--RFPG-------------TNLLQCPEVGKD------IKTCQK-K-GKTILLSLGGA-A----GAYGFANDKD-AVAFA------DTLWATFGG-GKSE----------RRPFG----------DAVVDGFDLDIEGG---------G-STG-----------YAAMVKRLRSHFNSD----------------------RSKKYYITGAPQCPFPD-----------------A--MLGPALD----A-SEFDAVFVQFYNN-Y-------CST----------------TSGNFN--FETWD--------QWARH-------------TSPNRNVKVFLGLPGSSAAA-----GSG-Y--VPYKTLEPVIRHL---YSTYSSFGGVMLWDASASYSNK---EVSPHYQAAIFKLLSS----------------------------------LSKGG---------------PS---KGGSSKTIKN-----------------------------------------------------TKTIS----------------SHTTKTTKSHRPTD--------------------------------------------------------------------------------------------------TPSV--------------RECVQKDQPCSGG---------------------------------FACSGN----------------------------------------------------------------------------------------------------------------------------------------------------------------------------------------------------------------------------------------------SFATCVHGR-----------------------------------------------------------------------------------------------------------------------------------------------------------------------------------------------------------------------------WLL--------------------------RPCPEGLVCLSSTDG-------------------VSAYCA-QGKAKTCT---RQGSFQA------------LAAVDSSVAK---AYTGRSVTAQFSFIGARQHRF--------------------------TAILNARCLSHQAFGERVKVQLKVS------------------------------QGIKITQVKG-----GTVRQQGDTV---------LIHYHNPIDKAMAIVIQMQGL-------------------MPTHGVFIG----------------------------------------------------------------------------------------------------------------------------PSMDSIK----------------------------------------------------------------------------------FY-----------------------------------------------------------------------------------------------------------------

>16099_R_oryzae_B

----------------------------------------------------------------------------------------------------------------------------------------------------------------------------------------------------------------------------------------------------------------------------------------------------------------------------------------------------------------------------------------------------------------------------------------------------------------------------------------------------------------------------------------------------------------------------------------------------------------------MEAD------IKFCQD-N-GKKVLLSLGGA-T----PEYGLNSYEE-GESLA------DELWYTFGG-GSDT--------NTFRPFG----------NASVDGFDLDIENG---------A-KDG-----------YPAFVNRMREHYAKE----------------------TSKEYYIAAAPQCPFPD-----------------F--FLGETLD----S-SWFDFIMIQFYNN-Y-------CNV---------------INGEQFN--YDIWD--------KWAKT-------------SSVNKDVRLFVGVPGSPSAA-----GRG-Y--VPFDKLVDTVKSL----QELESFGGIMIWDVSQAYGNT--LDVSPSYAHGVARLIHGDKNE----D-------------DGLPM-------LSSSA---------------PSLVISPASSSTDQAVLASATDATK------------------------------ENNTPKGTTTAINTGKED--------------------------------------------------------------------------------------------------------------------------------TPSS--------------NVNSNISNNSVYG-----------------------------------------------------------------------------------------------------------------------------------------------------------------------------------------------------------------------------------------------------------------------------------------------------------------------------------------------------------------------------------------------------------------------------------------------------------------------------------------------------------------------------------------------------------------------------------------------------------------------------------------------------------------------------------------------------LTEYQAMDNVQMDIG---------------------------------------------------------------------------------------------------------------------------------------------------------------------------------------------------------------------------------------------------------------------------------------------------------------------------------------------------------------------------------------------------------------------------------------------------------

>Chi18-15_H_jecorina_B

------------------------------------------------------MTR-------------------------------------------------------------------------------------------------------------------------------------------------------------------------------------------------------------------------------------------------------------------------------------------------LLDASFVL-------------LPAIAS---------------------ALFG---TA------------------------------------------SAQNATCATKGKPAGKVLQGY--------WE-NW-------DGASN------GVHPPFGWTPIENPQI--------------------QQHGYNVINAAFPVILP----DGTALWQDGMDATVKV---------------------------ATPAE------MCQAKA-A-GATILMSIGGA-T----AAIDLSSSAV-ADKFI------STIVPI---------LK----------------------QYNFDGIDIDIETG--------LT-GSGSVGTLSTSQANLIRIIDGILAQMPSN--------------------------FGLTMAPETAYVT---GGSITYGSIWGSYLP--IIKKYVD----N-GRLWWLNMQYYNGDM--Y--G-CAG------------------DSYA--AGTVA--------GFIAQTDCLNNGLTIQGTTFKVPYSMQVPGLPAQSGA------GGG-Y--MSPSLVGQAWDHY------NGALKGLMTWSINWDGSKN------WTFGDNLNGRI-----------------------------------------------------------------------------------------------------------------------------------------------------------------------------------------------------------------------------------------------------------------------------------------------------------------------------------------------------------------------------------------------------------------------------------------------------------------------------------------------------------------------------------------------------------------------------------------------------------------------------------------------------------------------------------------------------------------------------------------------------------------------------------------------------------------------------------------------------------------------------------------------------------------------------------------------------------------------------------------------------------------------------------------------------------------------------------------------------------------------------------------------------------------------------------------------------------------------------------------------------------------------------------------------------------------------------------------------------------------------------------------------------------------------------------------------------------------------------

>Chi18-19_H_jecorina_B_V

------------------------------------------------------------------------------------------------------------------------------------------------------------------------------------------------------------------------------------------------------------------------------------------------------------------------------------------------------------------------------------------------------------MPP-IPPQ---------------------------------------------------ATNPLPRVITY--------Y----QTHHDP-AG------------KHISLLPLIT------------------------QPGISLTHV-ILAAIHLND-DPHHITLND--------------------HP-----PHHPRFDPLWAE------LRILQA-S-GVKVLAMLGGA-AK---GSYAR--LDA-SD---------EQFERYYRP-LRDV-IR----------------------ARGLDGLDLDVEEE--------MS-LGG-----------VVKLIDRLRSDFGKG--------------------------FLITLAPVAAALL---DHRS---NLSGFDYE--ALEVMR-----G-HEIAWYNAQFYCG-----------------------WGDMNNPIMYE--MIIRK--------GWPI--------------------EKVVVGLVTNPANG-----S-G-F--VIWEFLSAVLTLL---RGRHERFGGVMGWEYFNSLPGG--HERPWEWARQMTSVLRN----------------------D-----------VSITS------------SRPPPIN--------ESTK------------------------------------------------------------------------------------------------------------------------------------------------------------------------------------------------------------------------------------------------------------------------------------------------------------------------------------------------------------------------------------------------------------------------------------------------------------------------------------------------------------------------------------------------------------------------------------------------------------------------------------------------------------------------------------------------------------------------------------------------------------------------------------PELDVE----------------------------------------------ADP------------DAPSDKPV-------------------------------------------------------------------------------------------------------QVPSQFDYYSDGSDA----------------------------------------------------------------------------------------------------------------------------------------------------------------------------------------------------------------------------------------------------------------------------------------------------------------------------------------------------------------------------------------------------------------

>MGG_04073_M_grisea_B_V

------------------------------------------------------------------------------------------------------------------------------------------------------------------------------------------------------------------------------------------------------------------------------------------------------------------------------------------------------------------------------------------------------------MPP-IPST---------------------------------------------------PGPHLPRIITY--------F----QTTHTR-DG------------KPISVLPLIR------------------------EPGVSVTHV-IVAAIHLNE-DPDAITLND--------------------HH-----PGHERYQTMWAE------MRILQA-S-GVKILGMLGGH-AR---GSYER--LDA-DQ---------AQFERYYAA-LHRM-IE----------------------THALEGLDLDVEEP--------MS-LQG-----------IVRLIDRLRADFGPD--------------------------FIITLAPVATALLGDDDPRR---NLSGFGYD--ALHAER-----G-HEIAWYNTQFYCG-----------------------WGDCSNPVMYE--LIMSR--------GRFP-------------------ADKVVVGLVTNHANG-----S-G-W--VPWEMLASVLPIL---VGRRADFGGVMGWEYFNSLPGG--EEKPWEWAMFMTALIRG----------------------D------------RAVA------------PQEQHVL-ESVESESQRERDERAR---------------------------------------------------------------------------------------------------------------------------------------------------------------------------------------------------------------------------------------------------------------------------------------------------------------------------------------------------------------------------------------------------------------------------------------------------------------------------------------------------------------------------------------------------------------------------------------------------------------------------------------------------------------------------------------------------------------------------------------------------------------------------QKKEAESQKAAAAV----------------------------------------MEADP------------DDSKGGDA-------------------------------------------------------------------------------------------------------PVPHEFEYHSDGRETPG----------------------------------------------SP--------------------------------------------------------------------------------------------------------------------------------------------------------------------------------------------------------------------------------------------------------------------------------------------------------------------------------------------------------------

>01513_S_sclerotiorum_B_V

------------------------------------------------------------------------------------------------------------------------------------------------------------------------------------------------------------------------------------------------------------------------------------------------------------------------------------------------------------------------------------------------------------MPP-IP------------------------------------------------------IPTTPRIVIY--------Y----QTQHHS-DG------------RPCSILPIIT------------------------QPNIAVTHV-NVAAIHLND-PPGNITLND--------------------HV-----PHHERYATMWAE------LRILQA-T-GIKVMGMLGGA-AK---GSFTR--LDQ-DE---------ATFELYYGP-LRNL-IR----------------------ERGLDGLDLDVEEE--------MT-LAG-----------IIRLIDRLKADFGSG--------------------------FIITLAPVAAALI-SERPEH---NLSGFNYE--ALEVMR-----G-NSIEWYNTQFYCG-----------------------WGDMSKTAGYD--MMMMR--------GWPA--------------------HKVVVGMVTNPGNG-----N-G-W--VPFETLREVLLNL---KSRYPTFGGVMGWEYFNSLPGD--KDRPWEWAAWMTNVLGK----------------------S------------PISA------------PSGPSTS-ASVAG--EKQR------------------------------------------------------------------------------------------------------------------------------------------------------------------------------------------------------------------------------------------------------------------------------------------------------------------------------------------------------------------------------------------------------------------------------------------------------------------------------------------------------------------------------------------------------------------------------------------------------------------------------------------------------------------------------------------------------------------------------------------------------------------------------------PLVDNN----------------------------------------------VDD------------DLQGDAPL---------------------------------------------------------------------------------------------------------PAEFEYHSEGLWED---------------------------------------------------------------------------------------------------------------------------------------------------------------------------------------------------------------------------------------------------------------------------------------------------------------------------------------------------------------------------------------------------------------

>05559_B_fuckeliana_B_V

------------------------------------------------------------------------------------------------------------------------------------------------------------------------------------------------------------------------------------------------------------------------------------------------------------------------------------------------------------------------------------------------------------MPP-VP------------------------------------------------------IPTTPRIVIY--------Y----QTQHHS-DG------------KLCSILPIIT------------------------KPNISVTHV-NVAAIHLND-PPGNITLND--------------------HA-----PHHERFVTMWAE------LRILQA-S-GIKVMGMLGGA-AK---GSFTR--LDQ-DD---------ITFELYYIP-LRNL-IR----------------------ERGLDGLDLDVEEE--------MT-LAG-----------IIRLIDRLKADFGAG--------------------------FIITLAPVAAALV-SDLPKH---NLSGFNYE--ALEVMR-----G-SSIDWYNTQFYCG-----------------------WGDMSKTAGYD--MMMMR--------GWPA--------------------HKIVVGMVTNPGNG-----S-G-W--VPFEVLQEVLLNL---KLRYPTFGGVMGWEYFNSLPGD--RDRPWEWAAWMTRVLGK----------------------L------------PVSA------------PKQPMTS-ASVIAEVETQK------------------------------------------------------------------------------------------------------------------------------------------------------------------------------------------------------------------------------------------------------------------------------------------------------------------------------------------------------------------------------------------------------------------------------------------------------------------------------------------------------------------------------------------------------------------------------------------------------------------------------------------------------------------------------------------------------------------------------------------------------------------------------------PLVSRN----------------------------------------------VDD------------DLEGDAPL---------------------------------------------------------------------------------------------------------PSEFEYHSEGYGDED--------------------------------------------------------------------------------------------------------------------------------------------------------------------------------------------------------------------------------------------------------------------------------------------------------------------------------------------------------------------------------------------------------------

>06595_C_immitis_B_V

------------------------------------------------------------------------------------------------------------------------------------------------------------------------------------------------------------------------------------------------------------------------------------------------------------------------------------------------------------------------------------------------------------MPP-IPESHG--------------------------------------I----------STLTNPRVICY--------Y----QTYYPN-NGT-----------DYISTLPLLT------------------------N-DCGVSHI-ILAAIHIND-EPGNITLND--------------------HS-----PDDPRYVPLWAE------MRVMQA-S-GTKVMGMLGGA-AK---GSYQR--LDG-SV---------ADFEAFYCP-LRDM-IR----------------------TRNLNGLDLDVEED--------MS-LDG-----------IIRLIDRLKSDFGDE--------------------------FIITLAPVATAMV---HGLR---HLSGFDYR--ALETAR-----G-SKISWYNVQFYNG-----------------------WGHMLHPSVYD--TIIHQ--------GWEA--------------------ERIVIGLLTNPANG-----SQG-Y--VPMETISSVLANV---LTKYPSFGGVSGWEYFNAMPGG--ISRPWEWAASISLIVGM----------------------K------------TVLA------------CATSALY-SKLATA----------------------------------------------------------------------------------------------------------------------------------------------------------------------------------------------------------------------------------------------------------------------------------------------------------------------------------------------------------------------------------------------------------------------------------------------------------------------------------------------------------------------------------------------------------------------------------------------------------------------------------------------------------------------------------------------------------------------------------------------------------------------------------------------------------------------------------------------------------------------------------------------------------------------------------------------------------------------------------------------------------------------------------------------------------------------------------------------------------------------------------------------------------------------------------------------------------------------------------------------------------------------------------------------------------------------------------------------------------------------------------------------------------

>01393_N_crassa_B_V

------------------------------------------------------------------------------------------------------------------------------------------------------------------------------------------------------------------------------------------------------------------------------------------------------------------------------------------------------------------------------------------------------------MPP-LP------------------------------------------------------HQHVPRLITY--------Y----QTHHTP-SG------------DPISVLPFLQ------------------------KPGINLTHL-ILAAIHINS-DPEGITLND--------------------HP-----PSHERFTTLWAE------LRILQA-S-GIKVLGMLGGA-AK---GSFAR--LDV-PDPDSPDKTALERFERYYQP-LKRL-IK----------------------ERGLDGLDLDVEEE--------MS-LAG-----------VIYLIDRLRSDFGPD--------------------------FLITLAPVAAAML---NPRH---NLSGFDYE--ALEVMR-----G-QEIAWYNTQFYCG-----------------------WGDCSNPVMYE--MMVMK--------GWAP--------------------EKIVVGLITNPENG-----G-G-W--VPWPALAGVLPLL---VGRHPRFGGVMGWEYFNSLPGS--RERPWEWAQVMTALLRG----------------------RQASD----AG-AQLLG------------ALGAPLHLDVAQQQHQEQQQQQAK---------------------------------------------------------------------------------------------------------------------------------------------------------------------------------------------------------------------------------------------------------------------------------------------------------------------------------------------------------------------------------------------------------------------------------------------------------------------------------------------------------------------------------------------------------------------------------------------------------------------------------------------------------------------------------------------------------------------------------------------------------------------------Q-SQPEQEKEGGGFEL----------------------------------------VDK------------DEV-DTPI-------------------------------------------------------------------------------------------------------AVPNSFDYHTDAEDEDE--------------------------------------------------------------------------------------------------------------------------------------------------------------------------------------------------------------------------------------------------------------------------------------------------------------------------------------------------------------------------------------------------------------

>XP_386145_G_zeae_B_V

------------------------------------------------------------------------------------------------------------------------------------------------------------------------------------------------------------------------------------------------------------------------------------------------------------------------------------------------------------------------------------------------------------MPP-LPPS----------------------------------------------------ANPLPRLVTY--------Y----QTHHDS-SG------------NLISTLPLIT------------------------QPGIAITHV-IVAAIHINE-DPEKIALND--------------------YH-----PSHPIFQTMWAE------LRILQA-S-GVKVMGMLGGA-CK---GSYAR--LDA-DE---------NTFERFYCP-VRDM-IR----------------------ERALDGLDLDVEEV--------MS-LGG-----------IVRLIDRLRSDFGPT--------------------------FIITLAPVAMSLI---DFRR---NLSGFDYE--ALEVMR-----G-RDIAWYNTQFYCG-----------------------WGDCSNPLMYD--LMVQK--------GWPP--------------------EKIVIGLLTNPENG-----H-G-Y--VPFNPLGMVLTTL---RGRYGNFGGVMGWEYFNGLPGG--KERPWEWAREMTELLRG----------------------H------------ILSE------------PAQVTQL--------TQQQQPVPV---------------------------------------------------------------------------------------------------------------------------------------------------------------------------------------------------------------------------------------------------------------------------------------------------------------------------------------------------------------------------------------------------------------------------------------------------------------------------------------------------------------------------------------------------------------------------------------------------------------------------------------------------------------------------------------------------------------------------------------------------------------------------E-SAPKKNEE----------------------------------------------VDP------------DTPGGKDV-------------------------------------------------------------------------------------------------------ALPKQFDYYTDGSDA----------------------------------------------------------------------------------------------------------------------------------------------------------------------------------------------------------------------------------------------------------------------------------------------------------------------------------------------------------------------------------------------------------------

>AN8245_E_nidulans_B_V

------------------------------------------------------MYAS---YF--------------------------------------------------------------------------------------------------------------------------------------------------------------------------------------------------------------------------------------------------------------------------------------R-LQSTVDNMLDL-------------VSIVAALWIV------------FELQQGKMPP-IPP-----------------------------------------------------PSYPHRIVLY--------H----QTLHPD-RG------------PYVSMLPLLN-------------------------NHTGTTHV-ILAAFHLNAPNPNHITLNN--------------------DP-----PDNPMYDRLWAE------VSILQE-S-GIKVMGMLGGA-AP---GTFTC--LDG-DE---------AQFEQYYGP-LLDM-IR----------------------RHGLDGIDLDVEEP--------MS-LAG-----------IVRLIDRLKADLGER--------------------------FIITLAPVAAALL----GIG---NLSGFDYR--LLEQQR-----A-GSIAWYNTQFYNG-----------------------WGPADDPRMYA--AIVAQ--------GWSP--------------------ARIVYGLLTNPGNG-----SQG-Y--VPQEVIGPVLATL---VEQFPNFGGVMGWEYFNSMPGR--LERPWEWAAQMSLNMGL----------------------NKAPGHAFVPIIFTSLA------------VYSTSLYFSSKKSNNGTIKTLLTGVPSRQSWATHITILINLLCGLFTADFLLRGFILYPTAGLRFSRVGYITHNTAELLLREPDASLLPLSVSYKEAQNEDSDPTSWIH--------------------------------AATIDALDESTDY------------TTTVTF-----------------------------TNLSPSSSYHYAL---------------SNNQTGFF-----ITSPDPDSSAANR--------------------------------------------------------------------------------------------------------------------------------------------------------------------------------------------------------------------------------------------------------------LSFLTSSCMKPNF----------------------------PYDPRRHPLRIPGLEKMTSAISSLPRLPSFMLFLGDFIYIDVPQR-----------------------------FGSSVDHY-------------------------------------------------------------------RSEYRR------------------------------------------------------------------VYSSPSWTWSAANLPWIHTLDDHEIANDWKHGNSTPPY-----PAAIDPYVHYHASVNP------------PAPDVTTE-------------------------------------------------------------------------------------------------------NTKHSYTMFTNGPAAFFLLDTRSYRSP----DDETILGKAQLNALLSF-------------LARPEPPHVRWKIVSSSVPFTRNWHAGTADTWGGFLSERRVVFDAMRAAQRALDIRVVLLSGDRHEFAATRFPTVPEYD--------PLVDSESQSPRIQNQEV-LESGEDLVEFCTGPLSMFYVPVRTYY----QSGPDDVPI---KYLPDGNTKFGLVEIE-------DGVVD--GVPSSVLTYSLYIDENMVWKYRLSVPLAL---TSEAEEPAASADAA--------AGTSA--------------------------------------------AGALLPPGEVL-----------------------VDKTVDGWVDVIAKRV-FGW-----DVMRVEHASKDWVV-

>07854_B_fuckeliana_B_V

------------------------------------------------------MKF-------------------------------------------------------------------------------------------------------------------------------------------------------------------------------------------------------------------------------------------------------------------------------------------------LNLLSVSL-------------LPLIQAI------------------------P-IEPL--------------------------------------------AAQNSTSDVATTPRLAVY--------V----QTYHDE-YNKD----------TNLSLLPLLG-------------------------EDILPTHV-ILAALHVMS-TPGVIHLND--------------------DP-----PDAPMYDELWDQ------VKALQS-A-G----------------------------------------FHRYYDPLLNNF-IK----------------------KYNLNGIDIDVEQF--------VD-ITV-----------ALRLIKQLYTDMGPD--------------------------FDITMAPVASALY---HGG----SLSGFSYS--DLDAQARDPDTGTPMISFYNAQFTNG-----------------------WGSAGGTNDYD--AIIGA--------GWDP--------------------SRVVMLASAATNDA-----S-G-W--VPISTLKNTIKSL---RNKYPNFGGVNGWEYFDAGSSD-GLSQPYMWMANLREALNV---------------------------------------------------------------------------------------------------------------------------------------------------------------------------------------------------------------------------------------------------------------------------------------------------------------------------------------------------------------------------------------------------------------------------------------------------------------------------------------------------------------------------------------------------------------------------------------------------------------------------------------------------------------------------------------------------------------------------------------------------------------------------------------------------------------------------------------------------------------------------------------------------------------------------------------------------------------------------------------------------------------------------------------------------------------------------------------------------------------------------------------------------------------------------------------------------------------------------------------------------------------------------------------------------------------------------------------------------------------------------------------------------------------------------------------------------------------------------

>03420_S_sclerotiorum_B_V

------------------------------------------------------MKF-------------------------------------------------------------------------------------------------------------------------------------------------------------------------------------------------------------------------------------------------------------------------------------------------LNFL--PL-------------LPLIQAI------------------------P-VESP--------------------------------------------AAKSFAHDVATTPRLAVY--------V----QTYHDE-YNKN----------RNLSLLPLLS-------------------------ADIQPTHV-ILAALHVMS-TPGVLHLND--------------------DP-----PDAPMYDELWAQ------VKTLQS-A-GIRVSCMMGGA-AW---GTWNY--FTG-DD---------NQFHQYYDPLLNNF-IK----------------------KYNLNGIDIDVEQF--------VD-ITV-----------ALRLIKQLYKDMGLG--------------------------FDITMAPVASALS---YGG----SLSGFSYS--DLDAQARDPNTGAPMISFYNAQFTNG-----------------------WGYAGNTNDYD--AIINA--------GWNP--------------------SRVVMLASAATNDA-----S-G-W--VPIGSLKNTIRSL---RNKYPNFGGVNGWEYFDAGSTD-GLSQPYMWMANLRDALDS---------------------------------------------------------------------------------------------------------------------------------------------------------------------------------------------------------------------------------------------------------------------------------------------------------------------------------------------------------------------------------------------------------------------------------------------------------------------------------------------------------------------------------------------------------------------------------------------------------------------------------------------------------------------------------------------------------------------------------------------------------------------------------------------------------------------------------------------------------------------------------------------------------------------------------------------------------------------------------------------------------------------------------------------------------------------------------------------------------------------------------------------------------------------------------------------------------------------------------------------------------------------------------------------------------------------------------------------------------------------------------------------------------------------------------------------------------------------------

>MGG_01876_M_grisea_B_V

------------------------------------------------------MKSS----F-------------------------------------------------------------------------------------------------------------------------------------------------------------------------------------------------------------------------------------------------------------------------------------------LGNLLVAL-------------LATTATQ--------------------SLALP-IDDG------------QAANA--------------------------TSAAADLLGVSPLPRLAIY--------F----QTTHSSATG------------RPISMLPLVT------------------------EKGIALTHL-YVCTLHVN--YDGEIHLND--------------------FP-----PGHARFATLWRE------AAVLRG-S-GVRVMAMVGGA-AE---GSFSRRTLDA-PDG---D----PDFEKYYGQ-LRDV-LR----------------------DFGLQGLDVDVEQP--------MS-LRG-----------AVRLVRRLREDFGPD--------------------------FVLTQAPVGTALS---RGGA--PNLSGFSYR--DLDQAV-----G-GSIEFYNAQFYNG-----------------------FGDMSTTAAYD--SVVAA--------GWDP--------------------RRIVAGQITTPDNG-----Q-Q-F--VPFDQLRRTMDEL---RARYGEIGGIMGWEYFNGKPGG--ESEPWEWAQEITAILRP----------------------DVPTV----LTVSQADA------------DELDEAWAVSVAAQPVMALGTAGVDPE---------------------------------------------------------------------------------------------------------------------------------------------------------------------------------------------------------------------------------------------------------------------------------------------------------------------------------------------------------------------------------------------------------------------------------------------------------------------------------------------------------------------------------------------------------------------------------------------------------------------------------------------------------------------------------------------------------------------------------------------------------------------------TPDVDYQAM--------------------------------------------VNA----------------------------------------------------------------------------------------------------------------------------------------------------------------------------------------------------------------------------------------------------------------------------------------------------------------------------------------------------------------------------------------------------------------------------------------------------------------------------------------------------------------------------------------------------------

>08394_N_crassa_B_V

------------------------------------------------------MPD-----F-----------SG--------------------SWKINVPSL---------------------------------FGSSSLWGLSFRSENRDLLCLG-----------------------------------------------------------------------------------------------------------------------------------------TCQAGEGM--GEKTSS------FLITSAIANHL-------NHRARPWSTIFRAATAL-------------MAILTPIIV------------------TAANP-LQIR-------THINGHTLSPRATFAYG-------------------QGNTTDVIIRTEIPRLIIY--------F----QTTHDE-SG------------KPISMLPLVT------------------------EKNIALTHL-IVCSLHIQ--KDGNITLND--------------------HQ-----PTDPRYGTLWQE------TQILGY-A-GVKIMGMIGGA-AA---GSFTPDTLDSANE---------TVFNRYYGQ-LHET-IK----------------------RYGLQGLDIDVEQP--------MS-QGG-----------IERLILRLRADFGKD--------------------------FIITLAPVASALT---MGGW---NLSGFDYR--KLMLAPSG--AG-KEVAFYNCQFYSG-----------------------FGDAGNPNQFH--QIITDNAQRDMRGALSP--------------------TKIVIGQLTNPNNG-----G-G-F--VTAGTLANSIKTL---RDVYGQIGGVAGWEYFNGQPGG--VEEPWKWAQTMTAILRP----------------------NQVPK----LKLTVEMA------------VKLKDVWKESAAAGMRQVVEVDGVEEN-------------------------------ADRTLAMVVVGLLDPDDADDVF-----AFVVLQIPHFRRNKHNHQQAQ-----------------------------------------------------------------------------------------------FQNNPEEPYCKGC-------GKLIDPKRTFGTTG-----------APASTAASQKQG---------------------------------------------------------------------------------------------------------AGKSKGFKRGGKDQDKEDEKPGQLQQQ---------------------------------------------------------------------------------------------------------------------EVSRFCTSRCRSESQHKKRDIK---------EQERQIEET----YVKLLQGQMVDGMEQLADGQADGKK----------------------KGKNGGVKPKKGEGRVVTLEEVETIVFGREKDAE--------------------------------------------KVYG-------------------RKKNRKSRALTSVGPNDEDTI---------TSTDEESQSTST------------------------------KAGRPSSRDSAI----------------------------------------GGIQIDA------------LQLGDKAFDD-----------------------------------------------------------EIAPL-------------------------------------PKGKSHPYTTGGHEVARL-------------------------------------AVRSGTRIRPPQE-------VSEVNGSVGGEKGRA--------ERKAESEEMKE-----EIRQKKAEGDRKAQQKEMIKSVARRG--------------------------------------------------------VVFGFDVPS-----SPDGNKKT---------------------------------------------------------------------------------------------------------------------------CECVMPGGKVVEPH-------------------------------FAKGN-WGIRWRGD---------------

>Chi18-20_H_jecorina_B_V

-----------------------------------------------------------------------------------------------------------------------------------------------------------------------------------------------------------------------------------------------------------------------------------------------------------------------------------------------------------MKASVYL-------------ASLLATL--------------------SMAVP-VKE-----------------------------------------------LQLRAEPTDLPRLIVY--------F----QTTHDS-SN------------RPISMLPLIT------------------------EKGIALTHL-IVCSFHIN--QGGVVHLND--------------------FP-----PDDPHFYTLWNE------TITMKQ-A-GVKVMGMVGGA-AP---GSFNTQTLDSPDS---------ATFEHYYGQ-LRDA-IV----------------------NFQLEGMDLDVEQP--------MS-QQG-----------IDRLIARLRADFGPD--------------------------FLITLAPVASALE---DSS----NLSGFSYT--ALQQTQ-----G-NDIDWYNTQFYSG-----------------------FGSMADTSDYD--RIVAN--------GFAP--------------------AKVVAGQLTTP-EG-----A-G-W--IPTSSLNNTIVSL---VSEYGQIGGVMGWEYFNSLPGG--TAEPWEWAQIVTEILRP----------------------GLVPE----LKITEDDA------------ARLTGAYEESVKAAAADNKSFVKR-------------------------------------------------------------------------------------------------------------------------------------------------------------------------------------------------------------------------------------------------------------------------------------------------------------------------------------------------------------------------------------------------------------------------------------------------------------------------------------------------------------------------------------------------------------------------------------------------------------------------------------------------------------------------------------------------------------------------------------------------------------------------------PSINYYAM--------------------------------------------VNA----------------------------------------------------------------------------------------------------------------------------------------------------------------------------------------------------------------------------------------------------------------------------------------------------------------------------------------------------------------------------------------------------------------------------------------------------------------------------------------------------------------------------------------------------------

>XP_382346_G_zeae_B_V

------------------------------------------------------------------------------------------------------------------------------------------------------------------------------------------------------------------------------------------------------------------------------------------------------------------------------------------------------------------------------------------------------------MLV-KGS------------------------------------------------ESVSEAKPYKRIVFY--------H----QTQYKD-E-------------RYISLQPTIL-------------------------PSVGVTHV-IVAAIHLN--TPTRITLND--------------------DL-----YDADKFIPLWDE------VKQLQA-A-GIKVLGMLGGA-AQ---GSYTK--LDG-SL---------EGFHKWYQP-LRKM-IK----------------------WAGFDGLDLDIEEA--------MS-LGG-----------VIRLIDHLKTDFGQS--------------------------FLVTLAPVAPALR----NEQ---NLGGFNME--ELEKGL-----G-SRIAWYNTQFYCG-----------------------WGDMSEPHDYD--RIIAR--------GWPQ--------------------EKVVIGLVTNPSNC-----S-G-Y--VKEAKLRKCLGEL---VMKYPRIGGVMGWEYYNARTEE--SSEPWKWAEMINDILNC----------------------DSKAQ----GG--NI--------------------------------------------------------------------------------------------------------------------------------------------------------------------------------------------------------------------------------------------------------------------------------------------------------------------------------------------------------------------------------------------------------------------------------------------------------------------------------------------------------------------------------------------------------------------------------------------------------------------------------------------------------------------------------------------------------------------------------------------------------------------------------------------------------------------------------------------------------------------------------------------------------------------------------------------------------------------------------------------------------------------------------------------------------------------------------------------------------------------------------------------------------------------------------------------------------------------------------------------------------------------------------------------------------------------------------------------------------------------------------------------------------------------------------------

**Subgroup-C dataset:**

>AbChit3

MPLEGLRLDG-------------------------------------------------------------------------------------------------------------------------------------------------------------------------------------------------------------------------------------------RVLHLV------------------------------------------------------------------------------------------------------------------------------------------------------------------------------------------------------------------------------------------------------------------------------------------------------------------------E------------------------------------------------------------------------------------------------------SDRRQ----------------------------------------------------------------------------------------------------------------------------------------------------------------------------LRSMRHTIRRLWQSE----------------------------------------------------------------------QAVLRRRQ----------------------R--------------------------------------------------------------------------------------------------------------------------------------------------------------------TAAQCG------------------------------------------------------------------------------------------------------------MLRIVGEHSGLPE-RRS-------------RG---------SGLDPRSFQIAPMD-ANA---ASLY---SRFTALKS--KKAGLETWISIGGWSFTDPG----ATRTIFSD------------------------MASSA-SGRSTFINGLVQFMSTYGFDGVDIDW--NIPVLMIAA-----------DVRTMV--------------------RIILTQPS------------------------------------------------I-------------------------------------------------SC---------------------------GVH-----------------------------EQ-------------------------------------------------------------RADAE-----------------------------------------------------------------------------------------------------------QGTANNIANTVCYTAR----------------------------------------C-------------GE-S------------CKPGTAEVAQFN---GQPGSLSTN-------DKC-DKKQY---------RSTCCDS-----KS---EVGT---------------------------------------------------------------------------------------------------CR----------------WRGY-------------RG--------------------------------AGL-----SC----IGG-----YAEGKTELTTNTNSH------------EKKGDKNCHG-G--------------------TQSYCCANFKP--DTSNLKTDLKDAAQAAAKAAAAQVAL--------------------------------------------------------------------------------------------------------------------------------------DIAAKAFCRLA----VPAL-------------LTPLELIEAA---------------------VLHRGRNLGY---------------C------------------------------------------------------------------------------------------------------------------------------------------------------------------------------------------------------------------------------------------------------------------------------------------------------------------------------------------------------------------------------------------------------------------------------------------------------------------------------------------------------------------------------------------------------------------------------------------------------------------------------------------------------------------GNRS-----------------------------------------------------------------------------------------------------------------------------------------------------------------------------------------------------------------------------------------------------------------------------------------------------------------------------------------------------------------------------------------------------------------------------------------------------------------------------------------------------------------------------------------------------------------------------------------------------------------------------------------------------------------------------------------------------------------------------------------------------------------------------------------------------------------------------------------------------------------------------------------------------------------------------------------------------------------------------------------------------------------------------------------------------------------------------------------------------------------------------------------------------------------------------------------------------------------------------------------------------------HPGPN-----------------------------------------------------------------------------------------------------------------------------------------------------------------------------------------------------------------------------------------------------------------------------------------------------------------------------------------------------------------------------------------------------------------------------------------------------------------------------------------

>AbChit9

-------------------------------------------------------------------------------------------------------------------------------------------------------------------------------------------------------------------------------------------------------MNL-----------------------------------------------------------------------------------------------------------------------------------------------------------------------------------------------------------------------------------------------------------------------------------------------------------------------------------------------------------------------------------------------------------------------------------------------------------------------------------------------------------------------------------------------------------------------------------------------------------------------------------------------------------------------------------------------------------------------------------------------------------------------------------------------------------------------------------------------------SL-----------------------------------------------------RLGPR------------------RRAWACVLALGLFQVSRAS----------SQSCASTELCGD-------------------SEPQRPSCS-----------AN--SHK-LKRVVGYYEEWSK-KRPCNQIYPEQIP---TGVYTHLNAAFAYIHAETYKLQ-PR-SEE---SSPY---ESLTSLKK--RERDLKVFIAVGGWAFNAPG----PTATIFSD------------------------MEADP-RKCDTFISSVVDFVNSHEFDGIDIDW--EYPGAED-----RGGKDE--DFANFPVFIAKLKKALSTTT-AGRH-GVSITLPAYSQYLRHFDMKALEPYVDFFNIMTYDL--HGSWDMSNG-V-----------GPYLNAHTNLTEVKKVMDLLWRDGVNPDKVVLGLAFYGRTYIMQEPDTCRDPG--CKYISKG-----SEAPCGNEAGV-------VFNSEIEQVAS------------QT-SGQA--------------QLYEDAAVKILRY-NH-----DHWMSF----------------DDEETFAMRVGFAKSQCLGGVMVWAVSHDTSDGRYS----LAVGNAVGRNAT--------------------PGPI-EAKLVSDTYGD-------------------------------ELNRTGSP----------------------------------------E-------------GE-G------------CPTGWLMTERNASEFRDGKELTEF-------KHC-QDRGN---------RQFCCAP-----QK---SLAS---------------------------------------------------------------------------------------------------TP----------------GKLP-------------RN----------------------------------------------------------LFVV--------------------GCLVLGLDGSAI------------------AAAAACLLA-----------------AYCLSFQAV-------------------------------------------------------------------------------------------------------------------------------------------------------------------------------------------------------------------------------------------------------------------------------------------------------------------------------------------------------------------------------------------------------------------------------------------------------------------------------------------------------------------------------------------------------------------------------------------------------------------------------------------------------------------------------------------------------------------------------------------------------------------------------------------------------------------------------------------------------------------------------------------------------------------------------------------------------------------------------------------------------------------------------------------------------------------------------------------------------------------------------------------------------------------------------------------------------------------------------------------------------------------------------------------------------------------------------------------------------------------------------------------------------------------------------------------------------------------------------------------------------------------------------------------------------------------------------------------------------------------------------------------------------------------------------------------------------------------------------------------------------------------------------------------------------------------------------------------------------------------------------------------------------------------------------------------------------------------------------------------------------------------------------------------------------------------------------------------------------------------------------------------------------------------------------------------------------------------------------------------------------------------------------------------------------------------------------------------------------------------------------------------------------------------------------------------------------------------------------------------------------------------------------------------------------------------------------------------------------------------------------------------------------------------

>AbChit10

-------------------------------------------------------------------------------------------------------------------------------------------------------------------------------------------------------------------------------------------------------MDI-----------------------------------------------------------------------------------------------------------------------------------------------------------------------------------------------------------------------------------------------------------------------------------------------------------------------------------------------------------------------------------------------------------------------------------------------------------------------------------------------------------------------------------------------------------------------------------------------------------------------------------------------------------------------------------------------------------------------------------------------------------------------------------------------------------------------------------------------------HCA---------------------------------EGVCINNCE-------GIKECGAG------------------KR--TCPLNE---EQSSLR----------SACGTNSGLCSR-------------------SEPQRPSCS-----------AD--SHK-LKRVVGYYEEWSK-ERPCNQISPEQIP---AGVYTHLNFAFARIDPKSYKLQ-----AN---ISDY---EGLTSLKKRDRDRDLKVFIAVGGFSSNKPD----SAATIFSN------------------------MVASP-EKSDIFISSVVDFVNSHGFDGIDLDW--EYPGAQD-----RGGRAE--DYVNFPNFVEKLKKALRKTD--GRD-GISITLPSSSGYLSHFDIKALELHVDHFNMMTYDL--HGTWDQQD--------------NPYLNAHTNLTEAKQAMDLLWHKGIIPDKIALGLAFYGKTYIMQDPDACHEPG--CRYVSGG-----AAAPCSNEDGV-------MLNSEIEQVIS------------NT-SVSP--------------RLYEDAAVKILSY-NH-----DHWVSF----------------DDEETLKMRIDFAKSQCLGGVMVWAVSQDASDGRYS----LAVGRAATTADADA-----------DNVSMFMPGPI-EAKVYEAEKPD-------------------------------DKECKWTN----------------------------------------C-------------LE-R------------CPKGLLRTKLNESDAHDGKDMSDA-------EYC-QGQGK---------LQLCCPP-----KQ---SRAF---------------------------------------------------------------------------------------------------TQ----------------RKRR-------------YN----------------------------------------------------------VWIA--------------------GVIILGWDGS---------------------VTSACLIA-----------------AGFITLKM--------------------------------------------------------------------------------------------------------------------------------------------------------------------------------------------------------------------------------------------------------------------------------------------------------------------------------------------------------------------------------------------------------------------------------------------------------------------------------------------------------------------------------------------------------------------------------------------------------------------------------------------------------------------------------------------------------------------------------------------------------------------------------------------------------------------------------------------------------------------------------------------------------------------------------------------------------------------------------------------------------------------------------------------------------------------------------------------------------------------------------------------------------------------------------------------------------------------------------------------------------------------------------------------------------------------------------------------------------------------------------------------------------------------------------------------------------------------------------------------------------------------------------------------------------------------------------------------------------------------------------------------------------------------------------------------------------------------------------------------------------------------------------------------------------------------------------------------------------------------------------------------------------------------------------------------------------------------------------------------------------------------------------------------------------------------------------------------------------------------------------------------------------------------------------------------------------------------------------------------------------------------------------------------------------------------------------------------------------------------------------------------------------------------------------------------------------------------------------------------------------------------------------------------------------------------------------------------------------------------------------------------------------------------

>AbChit12

M-------------------------------------------------------------------------------WTIL-------------------------------------------------------------------------------------------------------------------------------------------------------------------LIALALLQAA-------------------------------------------------------------------------------------------------------------------------------------------------------------------------------------------------------------------------------------------------------------------------------------------------------------------------------------------------ELCFASPESLPS------------------------------------------------------------SSRRS----------------------------------------------------------------------------------------------------------------------------------------------------------------------------LYARRFEVSRDNDPT--------------------------------------------------------------------RALSTLSARE-DY---------------------------------------------------------------TCGPG-----------------------RPCS-----NKACCGE------------SGNCSYGASY----C----------------------------------GKGCTSNCD-------SVAECG--------------KDSKDGKT--TCPLNT---CCSQY-----------GFCGTTKEFCGK-------------------------GCQSNCDEKPSPPIGSTKDKT-LSKVIGYYEAWNA-RSSCHMTSPRDLP---LAALTHLNYAFAYLDPTSFSIKTMD-AATP--TSLF---DDISDLKT--TNPDLKIFVSIGGWTFSDNN---TYTQPIFGD------------------------IARDP-SNRQLFANQLVAFLIYYGFDGVDLDW--EYPGAPD-----RGGKKE--DTKNYVELVQTIRATFDESQ--RGF-AITFTAPSSYWYLKWFDLAGMMKHCDWVNLMSYDL--HGAWDSTNP-I-----------GAIVQGHTNLTEIKLATELFWRAKIPAHKIALGFGFYGRAFTLANP-GCSTPG--CPFSAGA-----KPGVCTKTSGY-------LAYYEVQDILK------------KNPDIKI--------------VHDKESAVKYFTF-DT-----DQWISY----------------DDKDTFKQKIEWANGVGFSGSLIWASDLAHAALTGKGV--LNLNSALSKKTLLAS---------------------------QKTKEDL--------------------------SSYLRKDCYVLED---------------------------------------S--------------------------LTACKSGDDQVGYDK-----------------------AGRPK-------DYKPICCPK-----GS---GLTK---------------------------------------------------------------------------------------------------CQ----------------WRGG-------------TG-----------------------------GTNAGR-----DC----NGQ-----CHAGEVKITMSGYGGSPGE---------SSETAHCTR-G--------------------QKAFCCG-------------------IGVFNDALGQCSWSDGV--------------------------------------------------------------------GSSCKSDQQKIAY------------VWDKTGWGT---------------------------VFKHGNEFCCPK---------------------NEPPPLSD-------------CRWVGK--------GD-------------------C------------ADNTCGGS-EVTLFTD-------------DRGDSFSGCSWG-------RKR---------------ALCCTPNMQTL---------QTETCDLDLCKLF----ENRLACD------DEAGD----YDSESYSEAGYDATEEGYTA----------------------------------------------------------------------------------------------------------------------------------------------------------------------------------------------------------------WVRRSSYVTGDGKIL------------------------------------------------------------------------------------------------WT------------------------YEDSGMPELNKRHIES--------------------------------------------------------------------------------------------------------------------------DAGSA--------------------------------------------------------VPGKPRHTILSLVEF-----------------------------------TGSALYRGK--------------------------------------------------------------------------------------------------------------------------------------------------------------NLVVTSRPYWPGLKSLKGDGSD-------------------------------------------------------------------------TLQLRGG---YSLLRDTC-----------------------------------------------------SSTAMKFIPA--------------------------------------------------------------------------------------------------------------------------AEFPKKGFQLEHVREINMIYSFV----------KSLLSGKLFSGADMK-----------NKLDPLAVYKG-WNKVYDVSLPQIGRVIRDAADYTRP----------------------------VTPNDRIFETIGSYAYREGVTFLPGPLN---LIKRTLMEGKSPLGSPQNFEKLLGRVSRLG---------------------------------------------------DEENMKIVLTAMQGTVAIF-----NYLNDAMLHRAFTKAGL------------------------------------------------TLTNEMQHADEFIPEL-------------------------------------------------------------------------------------------------KGILAAWKEWEP-------DYYEVVTS-----KATKWLVDRGGLIAGKFVNTVNNPAAV-----KLASEAAQLVAQAGQIR--------------------------------------------------------------------------------------------------------------------------SPLSP-----------------------------------------------------------------------------------------------------------------------------------------------------------------------------------------------------------------------------------------------------------------------------------------------------------------------------------------------------------------------------------------------------------------------------------------------------------------------------------------

>AbChit13

MR---------------------------------------------------------------------VFFSSTACLWLHVA-----------------------------------------------------------------------------------------------------------------------------------------------------------LLIEQSSASQLDRSAIPRIGSMS--------------------------------------------------------------------------------------------------------------------------------------------------------------------------------------------------------LESKVHLGVESLGHQRIDF------------------LYHDTWG-------------------------------------------------------------------------------TPLVNGCPKPCAATGPDPLN---WTQVHSKHQLWRCDEDLLFDMNIYGAADGRGV---IRTCSLRRAFETSSTPVMSELDASRRQNFQ---NMTGLASKRATSNLTIS--HGC---GAHLSTVPVILSASNKLFSS------------PGENGTRAAKHLADYLLN------------SATCGTTILFAKTGPAITGLYVGGDIEMGSFGQLLLKYG-------------------------------------------AIRAVKLWSEGG---CLDRVG-----ARVLSNATVLTILSST-----------VKQDIVPKTTTNSTLRPSQP---------VSVLVARG-------------------------------------------------------VCRVIQVQQGDSCASLSSRCGIRGRDFSNYN-----PKRDLCSTLMPKQWVCCSEGNLPDMRPQRESDGTCRSYRVQSGDGCWAIANNFGLEQRDIEDLNK-MTWGWAGCAHLQAGQIICLSEGNTPMPAQVDGVRCGPQKPGTQKPSGSFTGKDLEKLN--PCPLNA---CCSGW-----------GYC---------------------DTGAPGAFKPGTNGCISNCE---MDIVGNEMTPG-GFARVGYFQGYNP-TRKCLRMDASEIESLEGESFSHVHFAFAGITHD-FNVRIPPESQE-----QF---EKFSRMEAT-----FRKILSFGGWAESTEP----ATFQLYRD------------------------AVKP--ENRLRFAKNVVTFLDRHGFQGVDFDW--EYPGATDIPGT-PPGSAD--EGQNYYRFLVVLRELLGW----GQK-TISIALPSSFWYLQNFPVDKMAKVLNYFIYMTYDL--HGQWDYGNKWSNPGCPS-----GNCLRSHINRTETFNSLVMVSKAGVPPWKLYVGVASYGRSFRMSNA-GCTGPM--CTFTGSFTVSEAEPGFCTETAGY--------TNADIR-------------------------------------LSDGTHGGGM-----------TDWVAY----------------MEDDTKSARRDWIRRLNFGGTTDWAMDLAEWHEGPQ----VEGSWS-----------------------------------------------------------------------------------------------------------------------------------------------------------------------------------------------------VQTKDVVCNPE----EW-PSTLEE----------------------------------------------------------------LSAKLDKIP------------------------------------------------------------------------------------------------------QEC-----------------------------------------------------------------------RARATVGIM-------------------------------------------------------------------------------------------------------------------------------------------------------------------------------------------------------------------------------------------------------------------------------SL-SLSNAIK--------------------------------------------------------------------------------------------------------------EYQAV-SNDYGDQFGWYADW-------------------------------------VKDAIDPRL--------------EEFMAIGRGEGLKYM----------DCKWWT----EKNEGEGPC--------------TEARLE----------------------------YIPSL---------------------------------RAGPRDVEFKMRDEEGFYKALLENT-------------------------------------------------------------------------------------GISKDWIT----------WRDHAIHDPCLHAC---------------------------------------------------------------------------------------------------------------------------------------PPSFPG----------CSIHAIQCSKN-YYMRKNFPRRI-------------------------------------------------------------------------------------------------------------------------------------------------------------------------NDKDAIHVENPKKIVD----------------------------------------------------------------------------DAIPNMGELVMVA----------------------------ISSYLEMTLGLSDAD-----------------------------------------------------------------------DADIIEALS----------------MPL-FM-LEEASRSIVEIKK-------------------------------------------------------------------------IGKEHKE---------------------------------------------EKTKELVMMILNIVFAAIPFVGQA-GTALGAAASIARAALIVGE-----------------------------LGNIAISVVE--------------IIDNPESAPFAI-LG--------------VLLGAGG--------------------------------------------------------------------------------------------------------------------------------LRSRTPKSA--------------------------------------------FKEAVKARRALSPADL-----------------------------------------------------------------------------KRFSPE-------------------------------------------------------------------------------FR--RRDGVVQGILWQ----------------------------------------------------------------------------------------------------------------------------------------------------------------------------------------------------------------------------------------------------------------------------------------------------------------------------------------------------------------------------------------------------------------------------------------------------------------------------------------------------------------------------------------------------------------------------CVR----------------------------------------------

>AbChit14

MR---------------------------------------------------------------------VFFSSTACLWLHVA-----------------------------------------------------------------------------------------------------------------------------------------------------------LLIEQSSASQLDRSAIPRIGSMS--------------------------------------------------------------------------------------------------------------------------------------------------------------------------------------------------------LESKVHLGVESLGHQRIDF------------------LYHDTWG-------------------------------------------------------------------------------TPLVNGCPKPCAATGPDPLN---WTQVHSKHQLWRCDEDLLFDMNIYGAADGRGV---IRTCSLRRAFETSSTPVMSELDASRRQNFQ---NMTGLASKRATSNLTIS--HGC---GAHLSTVPVILSASNKLFSS------------PGENGTRAAKHLADYLLN------------SATCGTTILFAKTGPAITGLYVGGDIEMGSFGQLLLKYGSS-WTAHELDNGAQSIQTCNT-EGHRSYTGGVFVADETDPLESAIRAVKLWSEGG---CLDRVG-----ARVLSNATVLTILSST-----------VKQDIVPKTTTNSTLRPSQP---------VSVLVARG-------------------------------------------------------VCRVIQVQQGDSCASLSSRCGIRGRDFSNYN-----PKRDLCSTLMPKQWVCCSEGNLPDMRPQRESDGTCRSYRVQSGDGCWAIANNFGLEQRDIEDLNK-MTWGWAGCAHLQAGQIICLSEGNTPMPAQVDGVRCGPQKPGTQKPSGSFTGKDLEKLN--PCPLNA---CCSGW-----------GYCGTTAEHCTE---------SRADTGAPGAFKPGTNGCISNCE---MDIVGNEMTPG-GFARVGYFQGYNP-TRKCLRMDASEIESLEGESFSHVHFAFAGITHD-FNVRIPPESQE-----QF---EKFSRMEAT-----FRKILSFGGWAESTEP----ATFQLYRD------------------------AVKP--ENRLRFAKNVVTFLDRHGFQGVDFDW--EYPGATDIPGT-PPGSAD--EGQNYYRFLVVLRELLGW----GQK-TISIALPSSFWYLQNFPVDKMAKVLNYFIYMTYDL--HGQWDYGNKWSNPGCPS-----GNCLRSHINRTETFNSLVMVSKAGVPPWKLYVGVASYGRSFRMSNA-GCTGPM--CTFTGSFTVSEAEPGFCTETAGYKSLPLSKIANADIR-------------------------------------LSDGTHGGGM-----------TDWVAY----------------MEDDTKSARRDWIRRLNFGGTTDWAMDLAEWHEGPQ----VEGSWS-----------------------------------------------------------------------------------------------------------------------------------------------------------------------------------------------------VQTKDVVCNPE----EW-PSTLEE----------------------------------------------------------------LSAKLDKIP------------------------------------------------------------------------------------------------------QEC-----------------------------------------------------------------------RARATVGIM-------------------------------------------------------------------------------------------------------------------------------------------------------------------------------------------------------------------------------------------------------------------------------SL-SLSNAIK--------------------------------------------------------------------------------------------------------------EYQAV-SNDYGDQFGWYADW-------------------------------------VKDAIDPRL--------------EEFMAIGRGEGLKYM----------DCKWWT----EKNEGEGPC--------------TEARLE----------------------------YIPSL---------------------------------RAGPRDVEFKMRDEEGFYKALLENT-------------------------------------------------------------------------------------GISKDWIT----------WRDHAIHDPCLHAC---------------------------------------------------------------------------------------------------------------------------------------PPSFPG----------CSIHAIQCSKN-YYMRKNFPRRI-------------------------------------------------------------------------------------------------------------------------------------------------------------------------NDKDAIHVENPKKIVD----------------------------------------------------------------------------DAIPNMGELVMVA----------------------------ISSYLEMTLGLSDAD-----------------------------------------------------------------------DADIIEALS----------------MPL-FM-LEEASRSIVEIKK-------------------------------------------------------------------------IGKEHKE---------------------------------------------EKTKELVMMILNIVFAAIPFVGQA-GTALGAAASIARAALIVGE-----------------------------LGNIAISVVE--------------IIDNPESAPFAI-LG--------------VLLGAGG--------------------------------------------------------------------------------------------------------------------------------LRSRTPKSA--------------------------------------------FKEAVKARRALSPADL-----------------------------------------------------------------------------KRFSPE-------------------------------------------------------------------------------FR--RRDGVVQGILWQ----------------------------------------------------------------------------------------------------------------------------------------------------------------------------------------------------------------------------------------------------------------------------------------------------------------------------------------------------------------------------------------------------------------------------------------------------------------------------------------------------------------------------------------------------------------------------CVR----------------------------------------------

>AbChit15

MT-----------------------------------------------------------------------------------------------------------------------------------------------------------------------------------------------------------------------------------------------------------------------------------------------------------------------------------------------------------------------------------------------------------------------------------------------------------------------------------------------------------------------------------------------------------------------------------------------------------------------------------------------------------------------------------------------------------------------------------------------------------------------------------------------------------------------------------------------------------------------------------------------------------------------------------------------------------------------DA----------------------------------------------------------------------------------------------------------------------------------------------------------------------------------------------------------------------------------------------------------------------------SRKKKSTKGCQSNCNLEPTPQGAKNSNGA-LEKVVAYYEGWSA-TRSCHTFPPSALP---IQALTHVNLAFAYIQPETFKITPMD-SSVP--ESVYLQTASVRGLKI--QNPDLKVFVSIGGWTFSDND---TSTQPVFGD------------------------VAKSQ-ANREAFASNLITFMREYGFDGVDLDW--EYPGAPD-----RGGQEQ--DGEHYVELLTTLRRDFDASG--QAY-GLTFTAPSSYWYLRWFSIERMAAYADWINLMTYDL--HGTWDSTDP-I-----------GSIIQAHTNLTEIKDAASLLWRNNVPPEKVVLGLGFYGRSFQLQNR-DCTKPG--CPFAGAA-----TKGDCTDNGGT-------LAYFEIQDIIS------------KQ-KPDV--------------IWDKEDAVKYIVYGSDK----DQWVSF----------------DDKDTFQQKVKYANDTGLGGVMIWSIDQDTDDFT-------ALEGLLGRSIPVTV------------------------DLEKKSIATA-----------------------ESWSSQNGQKCKMTE----------------------------------------CLND-------SDKGS--------------WGRGWAMAPNGD----------------AFKDNC-GKGKNKYVSNTLPLHRLCCKA---------YSPAF---------------------------------------------------------------------------------------------------VP----------------TRGG-------PLLTMDRS---------------------SAQ--------------MTRC---------RVLAHGEEVSL--------------------AAHVMVSAT-------P--------------TRSLCFTLSTGLAVVANLA-----GRLSAANRIHGSSSWALVA--------------------------------------------------------------------SPTM-------------------------------------------------------RNVLAASQKFLGDTHL-------------------TTPVSGQE---------------------------GNSVD-------------SGTC--------W------------EIEMTTD-------------DLGQSYSQCGAG----WLNRKK---------------VLCCKPPDGSI----------TPFLPVELSKVF---PDLPPAGDT--------------PIFDIQSIQSYGGSREAFGW------VLIDGPPSVV------------------SSLSKRDGSHAEF--------------LDCEP-SRKQDMRTYI--------------ARYVCRSSDNTSNCHD---VHLGGA-PGTVVRLPEECGYATWGVVHDIYPARNQ---TVQ-GELMKRLPT-----HSEVYEIEF-SYDFTRVKR------GAGDVYVRIDYGDINDYWEKVVKAA---------------------PVNGNKRDVDG-----------------------------------------------------RLDKRFWSSISKA-----WNNKTLPGSGE---------------YSLEISKEKFSSLIYQKDDSSSCNSG--------------------------------------GWLSVSLAGTAQS------------SIRWGFSMVGTIS-------PTLDFSEAYGFFDSKLLLDASLLVD----GRGSLDMNGSTKLAPLFSSDISKF-AFSHPGIVSFSPKMNVAVAMSGSGLFDG-----HSPIISGCRN---------------------------FA-------------VNFKAMTDGNVRVNSPGLGDL----AGDMQEYTYNNAFSGHVSGSTSGSQGTILGVQVRPR------SWIDVDVYDFG-SSLAAGNIQFSAELPHQVRVNTNG--------------GLLMVTDGSEKVV-AEAFSVGISDWES---RGGSAQLLGHNQ--SPA----VLYSGSGDDPPDRRPPKWNGHSV-------------------------------------------LTG----------DYITCS---------------------STKKLVCPML--------------GNMSIWDPDILIDPD------------------TGSSYEKRSLFNTLNFSENELR--------------DLHL---RGTGSA----RTYTITTPSGTTFVITS-HSYPNGQNGAYLILQNPDAGYHTLIDAS-------------DCENIEFT-----------ENGDP--NLEKFVTEHIMELSYMAELL----------EFSLSGFSTRADGSVHI---------ASAKPVDATFV---------------------------------DTNGYLQTPWNR-WAANNPSTTTPLDDMWAAFGDINNPGRLVNAEAAFN---GLKMRVWRGHAPFSEATIQDNSWLDTSEST---------------------------------------------------GFNHATQFTGAIRNVIAVF-----EYLNSNTVNTNLADVHN------------------------------------------------SILDILQRFDGAVEALT--------------------------------------------------------------SHTITM-------------------AD-----------------------FHVDLFRNVVI-PRLESVELWVERQIQRLTAAWEQVQSQPMSS-----SRAAEVALILEALKQLK----------------------------------------------------------------------------------------------------------------------------------------KRAEDSILFDLSRVRTS---------------------------------------------------------------------------------------------------------------------------------------------------------------------------------------------------------------------------------------------------------------------------------------------------------------------------------------------------------------------------------------------------------------------------------------------------------------

>AbChit16

M-----------------------------------------------------------------------------------------------------------------------------------------------------------------------------------------------------------------------------------------------------------------------------------------------------------------------------------------------------------------------------------------------------------------------------------------------------------------------------------------------------------------------------------------------------------------------------------------------------------------------------------------------------------------------------------------GKTR-----------------------------------------------------------------------------------------------------------------------------------------------------------------------------------------------------------------------------------------------------------------------------------------------------------------------------------------------------------------------------------------------------------------------------------------------------AELLLTKSN---------------------------------------PQQI--------------------WVLLASEFCGD--------GCQKNSKGEGCGQPTREKCASDTN-----------LVD-LSRRVAYFNMDSA-SKSCSYRNFSQFQ---LGALTDLNLAFVNFGDD-FQLKVGF-------SSLL---NDALALKL--RYPSLRVSIAVGGWDFSDP-----PSASRWSNSTPLISLVPCCCLRRPKNTDPQLAVVSTH-ESRRAFITSVYVTIQNYGLDGVDLDW--EYPSALD-----RGGSPD--DAANFVALVSEMRDYFDVQN--PRW-TITVTLPTSYWYLRGFDLENMNRFVSWFNLMSYDLAQHGTWDHDNRFT-----------GPYLRGHTDISEIQAGLDILRRNHIPMENVAMGMAFYGRSFTLGSD-DCVDPNGVCQFSRGG-----RPGDCSEQAGI-------LTYTEIVSRND------------SL-GLST--------------FYNSTTTVKYNVY-EG-----DQWISY----------------DDAQSWEDKRKFLSEQCFGGVMIWSLDQDDDGFD-------ALYGLMGDVSSLELS---------------------GGPPARELKEKL----------------------AHEIGAFTGQDCFVTPR---------------------------------------C-TD----------GSHDQQGPEQ-----VCPNGYQSMSTAHAPRQAPGH--------PYYGEC-AQGWY---------RHICCPR-----DA---LPKD---------------------------------------------------------------------------------------------------CS----------------WSTK-----------LSKS----------------------------------------SC----LHG-----CTQGQFMLNSDSFEE-------------ATGDNACYL-G--------------------RRAFCCD----------------------STRKISECVWSGCQ---------------------------KPDRDKPDQDWDCGRGFRYQTVRRG-----W----------GPGC-EDEDG-----------------------------------------------------------------------------------------------------------------------GR--------------------------------DPLTCPEG-KVKVAEA---------KYPSSNYDWKNVCDTYTLAPGMNPRF---------------PYCCAPPSQY-----------DRKWPVDPEKLFKTYYDDPSHSDVVWSYNDEKDNNNKINPSNNQGPDLYGSDSYGF--------VMLDGPEGSIDNTFA--ATFTVVREEREIRSSKRSLLTSNQTLIDS---------------VFDHSEETLQ----------------IYCNFPLGAKECER---LWLNGP-EDTIVRLPSHVGEGPFARLVSIKELDSQSHSQLPSHHLEHRSTED---LRGPLYEMKI-DYDFQDIVPKD----EDKPVMMRIDFTNLYGYWDEMTNAE---------------------PDSNSKRDEG----VVSDWHSRVQRAIARDEV-LRKR-SSPVNFTVPMAVDDVFEAQQSEAESGHIDKRWWGTFTN------WLSRMTTVTKSEIGDLPLSWGDTINLFRAKS--------------------------------------------GCPG-------RTFSASLSVDLEAEVEM------------DATYAYYFSGTF--------VPPSTPDVYVYFGMNPNAYIGVKIT------GDAQMQYKSEKKKIIDTLAHP--GLAVKGIAAVGSTLDIYGQIAGKVTIHGE-------AKAGARVNFGKAE---AYWPQ---------------------SDEASQ--KYEKLLGIDSKSEVPHKDTL-------EPRFEAGVEIDASLDVTLQPQAN--VGIKIGGGKLVAGKVIMDAQLSGFV-------TGTLSFQASGSADTSSKQFRYSYGVYVFYNI-----GYSAKATVL-------GIIDWAL-----------GPRQAYNPSRRL-DVYGPIEGTIPLTADSKRDL----------IPALIQEQPD----PNNGSARLLDARANLALE----------PSDPQFSQPITCP------------AGAAG-WRLPELRYNCDIL----------------GDQQIPGQG-------------------------ETGRGAIVPGVCSA--------------------------------------------------------W---------------------------------------------------------------------------------------------------------------------------------------------------------------------------------------------------LGLDPFPETLTFSKSADRVNARRGRQCPRGYCGKEEKGYREA------------------------------------------------------------TGSDIQVHCDETPPASSEEGG---DYLPEGSRSRLCVTAVQNSNY---------------------------------------AGLFGNLRSNWGQMEPSPPAGG-RIDRWVGWEDNAWTTEGTE---------------------------------------------------TDNRQRVTDYPTNMPDPDGI--AFRHNLATSWVFKRNY-----TWSINTSPN-----QASSWFDATSREFSTTKYATS-------PGTPNGWDGVLCVLNSFGQDTYYQY---------------KQDFNAYCVH---------------------------------------------------------------------------------------------GPKF----------KSRGFGEAWHLGRCKVSFGNTEMPTSK--------------------------------------------------------------------------------------------------------------------------------------------------------------------EWDIQH--------------------------------------------------------------------------IEYLEDYDED-EEIAL-------------------------------------------------------------------------------------------------------------------------------LTGNLDLNKEAFN-----------------------NFSGEN--------

>MAC_Chit20

MI-----------------------------------------------------------------------------------------------------------------------------------------------------------------------------------------------------------------------------------------------------------------------------------------------------------------------------------------------------------------------------------------------------------------------------------------------------------------------------------------------------------------------------------------------------------------------------------------------------------------------------------------------------------------------------------------------------------------------------------------------------------------------------------------------------------------------------------------------------------------------------------------------------------------------------------------------------------------------------------------------------------------------------------------------------------------------------------------LRP-----------------------------------------------------------------------------------------------------------------------------------------------------------------------------------------------------TGSIPSIHIRQIA---KGNFPGNNETLA------MSSGHLD-SEG---KDLW---EDFKKLPN------VKRILSLGGWAYSTEP----ATYGIIRN------------------------AIV---NNSQVFAGNCAQFVKDEGIDGVDIDW--KYPGA--------------------------------------------------------FPFDRIASIVDCIVYMAYDL--HGQWDAGNPNAFDECPS-----GRCIRSHGSRTD----------------------------------------------------SSANPGRCTNTRGY-------LAYAEIKEVKR------------AGVNVKT--------------FHDDDSNTDVLLY-NA------DHVGY----------------TTPTTKDTRRTDWRKLNFAGTIDWTVDLQKFSEDEM----TNTDQPISGQACVSG---------------------------------------------------------HDVTLETAEMCEFACEHDF------------------CPPF-------------LCICD--------EKGKLK-----------GLPAEKKDVEGHH-----------------LGTFCNESQPQ--------WATITCPP-----EA---GKPN---------------------------------------------------------------------------------------------------------------------WRQD-----------------------------------------------------------------------------------------------------------G--------------------------------------------KATLTWAVQHDGNEWEDKA-------------------------------------------------------H----------RQEPQCERDEYPPAY-----------------------------------------------------------------------------------PMNMQDDA-----------WKWGGQPQ------GN------------------------------GQLVRFLPAK-QNQKAGKLWQ-----------------------------------------------GIC------------------------------------------------------------------------------------FEGPVGAISDT----------------------------------------DLVNRVRNTPAKQQDVFKDADKPP----------------------------------PLPRLQLPK-----------------------------------------------------------------------------DHDGKWRKPTPQR-------------------------------------------------------------------------------------------------------W------------------------------------------------------------------------------------------------------------------------------------------------------------------------------------------------------------------------------------------------------------------------------------------------------------------------------------------------------------------------------------------------------------------------------------------------------------------------------------------------------------------------------------------------------------------------------------------------------------------------------------------------------------------------------------------------------------------------------------------------------------------------------------------------------------------------------------------------------------------------------------------------------------------------------------------------------------------------------------------------------------------------------------------------------------------------------------------------------------------------------------------------------------------------------------------------------------------------------------------------------------------------------------------------------------------------------------------------------------------------------------------------------------------------------------RP------------------------------------------------------------------------------------------------------------------------------------------------------------------------------------------------------------W-----------------------------------------------------------------------------------------------------------------------------------------------------------------------------------------------------------------------------PC-------------------------I----------------------

>MAC_Chit22

MKAL---------------------------------------------------------------------------------------------------------------------------------------------------------------------------------------------------------------------------------------------------VTFTVFL------------------------------------------------------------------------------------------------------------------------------------------------------------------------------------------------------------------------------------------------------------------------------------------------------------------------------------------------------------------------------------------------------------------------------------------------------------------------------------------------------------------------------------------------------------------------------------------------------------------------------------------------------------------------------------------------SALVRLSVQQ-DG---------------------------------------------------------------QCSAT-----------------------KLCP------AGCCSS------------AGYCGYGPDY----C----------------------------------GKGCQSTCG-------RKSDCNPGW----------DSGDFSKKD--KCPLNV---CCSKH-----------GFCGYTDEFCEG-------------------NEVKRPSCS-----------VS--DTS-VDRVIGYYEGWATSNRGCNALRPEDIP---YGAYTHLIFSFATINPKTFE-----------------------------------IWVAIGGWAFNDPG----PTQTTFSD------------------------VAASK-ANTNTFLSSLVKLMDKFNFDGVDIDW--EYPVAPD-----RHGRGE--DYENIVTFMKKLRERMDK----SRR-GVSMALPASYWYLQHFNVVKLEEHVDWFNLMTYDM--HGAWDIDNKWT-----------GPWANSHTNLTEIQLGLDLLWRNDISPSKVTMGMSYYARTFTLTDP-GCNKPG--CRVSSPG-----DAGKCSGTAGV-------LLHPEIQEIIS------------KN-NLKP--------------VLDREAAVKTVSW--G-----NQWTSF----------------DDVATWRLKANKLRGQCITGFMVWAISQDDEFGTNA----KALAAVLGRKKLEFP-----------NFAVR---------AKVEDSPDL-----------------------------ESKTCRWTS----------------------------------------C-------------YE-G------------CPSGFKEVQRDG----HKEIMLDT-------TPC-FGKGH-------GFSRLCCPT-----SF---KSPT---------------------------------------------------------------------------------------------------CT----------------WRGH-------------RN--------------------------------TG------KC----KPG-----CNDFELEV--------------------GSLDVGCRS-G--------------------YQSACCS-------------------DTLVTSLYSRCVW---------------------------------TECTSKG--------------------------------KDAC-------------TGNNPHFLTSSSIGSGG------------------------------------------------------------------------------------------------------------------------------------ACSSD-QLMLARESGLTIG------------------------------SEGGDGCYGDL---AFCCTEPKKLKPR------D---------------------------------------EDPGSSEGREFQALIRKY----------MENPTC---------------PAT-ILFPTVHDKFT-------------------RKR-SLEFEAAEF---------------NVLHARAKDCTLDTWV---RLLQYA-------------------------------------------------------TLVF-SVSRAGMDP-------------------LVKVWDKEF-------------------------------------------------AGYY----------------------------DKQLEYQNLHAFL--------------------------------GKYRD-YEPRS--------VVEWVLYNPTQAG----------------------------------------------------------------------------------------------------------------------------------------------------------PGIKGANAAS-------------------------------------------------------------QALCR-------------------------------------------------------------------------------------------------------------------------------------------------------------------------VPGSSTRSVPE--------------------------SALSDIKSRKVDAWRT--------------------------------------------------------------------------------------------------------GRGSIPALDTILSA-------------------------------------------------------------------------------------------------------------------------IISGAVTLHYARWQYYRSSQ--------------------------------------------------------NGNVPGPMLEMAYWLGSQPG-----------------------------------------------RENNED-VTQYQDRTPRPNWPSDQWVLFHPHIDPSN-----------------------------------------------------------------RNWLINENGN---TYVG---VRDFRVMHGQYWMH--------------------------------------------------------------------GSPGQAWRVSR---------------------------------------------------SNGRY-NSRDGFSCE------------EELGSLWYVGSR-------ITLPNNEP-----EWARLLHQWGEMIHRQGYVGNRGLRM--------------IIEPPQGGSSTEVDPH-------------------------------------------NPGTLS------------------------------------------------------------------RGYM--------GTTVVGGNNPYDINFLLSE-QGYEFSTPPPPKDREDPDTPADQ-----------------------------------------------------------------------------------------------------------------------------------------------------PEW-------------------------------------------------------------------------------------------------------------------------------------------------------------------------------------------------------------------------------------------------------------------------------

>MAC_Chit23

ML---------------------------------------------------------------------------------------------------------------------------------------------------------------------------------------------------------------------------------------------------DSQAAVDAYLEE------------------------------------------------------------------------------------------------------------------------------------------------------------------------------W-----------------------------------------TAANDRTP---------------------------------------------------------------------------------------------------------ASAERKCPPLCTEFGSDPKN---WHLYSEVAELQQCNETLVFDFAVDQELDSKHNKGMFRACVADYGPAP----------ATRRAGLST---------------------AGCS--VVYPSKIKATVELGWA---------GSSSSSDASRAAVIAGSQISRHIKSR----------ISTCANNTISFGSSGRTLVGLHAGSQVLSQGVAENVLDEFASHAEKNGLSDK-TVFQLCASRDRGADFAIGIAVSLSGD-VSFIQSTVKAWNNGA---CAPDVG-----STEKWADFTLQVPNVT-----------SVHGLSRRS--------------------PPPANSDG-------------------------------------------------------TCATKRVDAGDSCAALAA-----------------------------------NRGTLPDFTPKPEPDGSCHVYNVYDDDSCSSIAAANSLTVDKLESFNK-YTWGWNGCKMLWSGINICLSEGSPPMPAPVSNAVCGPTVPGTKKPS---GGKNLTDLN--PCLLKV---CCNIW-----------GQCGISS----------------SETGAPGTAKPSTNGCIQNCG---SDIIQSS-PPK-ETVRIGYFSSWNA-NREC---GVHEID---TKAYTHIHFAFANVTSD-FKVDISG-VPD-----EF---ERFKKLTGV------KKIISFGGWDFSTNA----GTFRIIRE------------------------AVKP--ANRNTFRDNVVNFVNDHGLDGFDLDW--EYPGAPDIPGVPPSGNPQ--EGLNYYKFLSIANSALG-----SGK-SVSFAAPASYWYLKAFPIELMAKSL--VVYMTYDL--HGQWDYNNKWASSGCPD-----GNCLRSHVNLTETILSLSMITKAGIPSNKVAVGITSYGRSFHMAHA-GCDGPD--CKFTGSATHSDAKVGMCTNTGGY-------IADAEIKDIINL-----------YPENIKQ--------------WID-TSNSNILVY-ND-----VEWVAY----------------MDKDIKDYRKKVYDVFHFAGTSDWALDLQEFQETKD-----------------------------------------------------------------------------------------------------------------------------------------------------------------VIPYEPPREMGI-----------------------------------------CNQAY-------DTIQA----------------------------------------------------------------IKADMDNIP--------------------------------------------W----------------------------------------------------------NC-----------------------------------------------------------------------KHQYIVSARQK--------------------------------------------------------------------------------------------------------------------------------------------------------------------------------------------------------------------------------------------------------------------------------QLTAALK--------------------------------------------------------------------------------------------------------------QYNDIFNDGYSHN-----------------------------------------------------------------DTGDIKR--------------------------------------C------------SFHNISIAC--------------PPKHP----------------------------------------------YDQAFNIYLLPKDDNKILNHLDKDF-------------------------------------------------------------------------------------GVSPDAV-------------------------------------YAEDW-------------------------------HY-------------------------------------------------------------------------------------GPCLPEQDKICS-----------CSKW-----GHLIGRK-------------------------------------------------------------------------------------------------------------------------------------------------------------------------AIKPDWKLPDPKKYVE----------------------------------------------------------------------------QALANITIINDFL----------------------------EEATTAMDMGIFIDS-----------------------------------------------------------------------ATDTVDATA----------------LPV-LM-IVAAVDSMEQIKE-------------------------------------------------------------------------AGEKIEE---------------------------------------------EERKNIILLALTAVFFVIPGLGEALGAVSGIAA-IARLATIIAE-----------------------------AGGAGLGVYN--------------IVNDPKSAPLEI-FS--------------MLMGGLA--------------------------------------------------------------------------------------------------------------------------------VKGAA---------------------------------------------RGWIGEAAGMRRAMDATHV-----------------------------------------------------------------------------KGMGRI-------------------------------------------------------------------------------LS--DGLGDVASVLKYWA-----------------------------------------------------------------------------------------------------------------------------RP-----------------------------------------------------------------------------------------------------------------------------------------------------------------------------------------------------------QWT------------------RTSL---------------------------------------------------------------------------------------------------------------------------------------------------------------------------------------EHL-GLIQIE-----RPGGNIRGRTAQEPRRIYERR-LAW----PSLRKR-SYRGGHGR-RGYLV

>MR_Chit22

MKAL---------------------------------------------------------------------------------------------------------------------------------------------------------------------------------------------------------------------------------------------------VAFTVFL------------------------------------------------------------------------------------------------------------------------------------------------------------------------------------------------------------------------------------------------------------------------------------------------------------------------------------------------------------------------------------------------------------------------------------------------------------------------------------------------------------------------------------------------------------------------------------------------------------------------------------------------------------------------------------------------SALVQLTVQQ-DG---------------------------------------------------------------ECSAT-----------------------NQAL-------------------------------PCR----M----------------------------------LKGCQSTCD-------RKSDCNPGW----------DSGDFSKKD--KYPLNV---CCSKH-----------GFCGYTEEFCKG-------------------NEVKRPSCS-----------VS--DTP-VDRVIGYYEGWATSNRGCNALRPEEIP---YGAYTHLIFSFATINPKTFEVSPDN-YKT---EDMM---ERIGTMKL--LQPDLKIWVALGGW-------------------------------------------------------CSLVKLMDKFNFDGVDIDW--EYPVAPD-----RDGRGE--DYENIVTFMKKLRERMDK----SRR-GVSMALPASYWYLQHFDIVKLEEHVDWFNLMTYDM--HGAWDIDNKWT-----------GPWANSHTNLTEIQLGLDLLWRNDISPSKVTMGMSYYARTFTLTDP-GCNKPG--CRVSSPG-----EAGKCSGTAGV-------LLHPEIQEIIY------------KN-NLKP--------------VLNREVAVKTISR--G-----NQWTSF----------------DDAATWRLKANKLRGQCITGFMVWAISQDDEFGTNA----QALASVLGRKKLELP-----------NFAIR---------AKVEDSPDL-----------------------------ESKTCRWTS----------------------------------------C-------------YE-G------------CPSGFKEVQRDG----HEEIMLDT-------TPC-FGKGH-------GFSRLCCPT-----SF---KSPT---------------------------------------------------------------------------------------------------CT----------------WRGH-------------RN--------------------------------TG------KC----KPG-----CNDFELEV--------------------GSLDVGCRS-G--------------------YQSACCS-------------------DTLVTSLYSSCVW---------------------------------TGCTNKG--------------------------------QDAC-------------TGNNPHFLTSSSIGSGG---------MKSCD--------------KGEKRSLCCIS---------------------PPPYDVSGK------------CEWRKKA-------GFLNADNEKLL----------C-------------EGACSSN-QLMLARERGLTI---------------------------------------------------------------------------------------------------------DPGSSEGREFQALIRKY----------MEKPTC---------------PAT-ILFPTVHDKFT-------------------RKR-SLKFEAAEF---------------NVLRARAKDCTLDTWV---RLLQYT-------------------------------------------------------TLVF-SVSRAGMDP-------------------LVKVWNKEF-------------------------------------------------AGYY----------------------------DKQLEYENLHAFL--------------------------------GNYRD-YEPRS--------VVEWVLYNPIQAG----------------------------------------------------------------------------------------------------------------------------------------------------------PGIKGADAAS-------------------------------------------------------------QALCR-------------------------------------------------------------------------------------------------------------------------------------------------------------------------VPGSSARSVPE--------------------------STLSDIKSRR--SMRG--------------------------------------------------------------------------------------------------------GPDEVTFLP------------------------------------------------------------------------------------------------------------------------------------------------------------------------------------------------------------------------------------------------------------------------------------------------------------------------------------------------------------------------------------------------------------------------------------------------------------------------------------------------------------------------------------------------------------------------------------------------------------------------------------------------------------------------------------------------------------------------------------------------------------------------------------------------------------------------------------------------------------------------------------------------------------------------------------------------------------------------------------------------------------------------------------------------------------------------------------------------------------------------------------------------------------------------------------------------------------------------------------------------------------------------------------------

>MR_Chit23

MKAS-----------------------------------------------------------------VYQVAIPHVLFFLQAAA----------------TLQG-----------------------------------------------------------------------------------------------------------------------------------GPPTVVRTSSAGALETL-----------------------------------------------------------------------------------------------------------------------------------------------------------------------------------------D-----------------------TEGNKELEEWLAANIRTP---------------------------------------------------------------------------------------------------------PSMLKPCPLPCSEA-PDSINGGGWFLVPDATSFAQCNKTLLLDLVVKS--DDPPA---LRACTADY--------------GSN--------YQLSKIHSPDDGNAALCS-------TPNHEMVKTSIRLLEVST------PTQNNDTFATQHLVSASRQVMHYLASQ----------KPSCSKNALAFGYSQTSVVGVFAGAEVHQHGLAIDIINSLLSYATERSISQTT-VLQLCQEHGLGADYSFGLVATNANN-LKFAQEAVRRWASGK---CVGPDG-----GRDWKE-VTIRVP----------SIVRPTNTTASSNMTGSRLGSRSFWQDL-ARVHCKTLTVRSGDGCWALAKICGVSQSDLTKYNRRANFCDTLVVGEKACCTRGILPSSIPDSKSDGTCVTKEVKSGDSCGSLASKCGLSASDFMRLN-----TKEDLCSTLAEGQQVCCSRGKLQDRRPKPESDGTCASVKTNRGDSCASIAASRDLTVRDIESFNE-DTWGWNGCKVLWVGFRLCVSEGKPPMPEPVSNAVCGPTVPGTKAPA---KGFDLSRLN--PCPLKA---CCNVW-----------GQCGLSDDFCIE---------SKSETGAPGTSGV-RNGCISNCG---RAIIRSS-PPP-SVIKIAYFEAWNH-KRSCLWMDIDDID---TTKYSHIHFAFGEVTKD-FRIDISK-VQT-----QF---YKLKAMVGV------KRIISLGGWDFSALP----GTYNILRE------------------------AVQP--RNRDKFITNIVDFLIQHDLDGVDLDW--EYPGATD--------DHQ--NGLNYFKLVDELKYRLS-----NAK-SVSFAAPSSYWYLKAFPISLMAMSLDYIVFMTYDL--HGQWDYGNKWTSPGCPT-----GNCLRSHVNMTETKDALVMITKAGVPSNKVVVGVASYGRSFKMAEA-GCYGPL--CKFTGTPRISHAAKGRCTDTSGY-------IANAEIEEILA------------SGKVTKQ--------------WR--EAGSNILVY-DD-----TEWVAY----------------MDDKLKDIRTQFYLMNNFAGTTDWAVDLQKFLPGDG----IPDDDF------------------------------------------------------------------EDEYEPYIDPDFYSD---------------------------------------------------------------------------------------------------------------------------CSGKY-------STLGD----------------------------------------------------------------VEKHLTTMP------------------------------------------------------------------------------------------------------THC-----------------------------------------------------------------------TEKYIAQAE-------------------------------------------------------------------------------------------------------------------------------------------------------------------------------------------------------------------------------------------------------------------------------SA-MIKEALT--------------------------------------------------------------------------------------------------------------KHLELLQDDYDSKFKTYERF-------------------------------------VKQQVPVQI--------------NNFMA------------SDKVHQYFKCSEYKKVICCSDCTYATCLETCANFAGCKSGYQTLDIKC--------------PQQKD--------ELEMIS--------------------------------METTPNATFRLEDEEGFWKEIGSQY-------------------------------------------------------------------------------------GIEEAWVA----------FGRRHMRTANGCQ-------------YAGKAIHE------------------------------------------------------------------------------------------------------------------------------------------CQNQNDRWWYNYPIAA---------------------------------------------------------------------------------------------------------------------------------------------------------------------------DDKIKVYNPKKIFG----------------------------------------------------------------------------DSTDTIASLAENL-------DIIKDLNI--------------------------------------------------------------------------------------YDTLML-------WSDVVDAAS----------------LPA-LT-LQAAVENMKSIID-------------------------------------------------------------------------KAEEIEK---------------------------------------------KQKEEFILNMVMGILFFIPVVGEAAGSAGLTAA--RSMLRLIGA-----------------------------AGDAGMTVYD--------------IVKDPSNAFMAA-FG--------------YVLGAGV--------------------------------------------------------------------------------------------------------------------GR--------------------S-----------------------------------------GFRDAANSRRAVRPREL-----------------------------------------------------------------------------AAVGSL---------------------------------------------------------------------------------------------------------------------------------------------------------------------------------------------------------------------------------------------------KTDLRRIQVSRKNI----------------------------------------------------------------------------------------------------------------------------------------------------------------------------------------------------------------------------------------------------------------------------------------------------------------------------------------------------------------------------------------------------------C---------------------------------------Y-L------

>MR_Chit24

MRRI---------------------------------------------------------------------------------------------------------------------------------------------------------------------------------------------------------------------------------------------------LEALILL------------------------------------------------------------------------------------------------------------------------------------------------------------------------------------------------------------------------------------------------------------------------------------------------------------------------------------------------------------------------------------------------------------------------------------------------------------------------------------------------------------------------------------------------------------------------------------------------------------------------------------------------------------------------------------------------SCWIQFSNQQ------------------------------------------------------------------QCSST-----------------------KHCA------IGCCSS------------AGYCGFGPDY----C----------------------------------GAGCQSTCE-------RQADCNPGW----------DGSQWSKQD--KCPLNV---CCSRH-----------GFCGFTEEFCDG-------------------KEVQRPSCS-----------PK--DKP-VSRVIGYFESWATSERSCFTMSPEEIP---YGLYTHIIFSFATIHPQTFEVSPGN-SQT---EYIM---SRIGAIKL--VQPDVKVWVAIGGWAFNDPG----PTATTFSD------------------------LAASE-AHMERFFTSLIKMMNTYGFDGIDIDW--EYPVAPD-----RNGRDE--DYKNIVSFMRELRHRMDQ----HKK-GVSMAIPASYWYLQHFDIAALEAAVDWFNLMSYDM--HGAWDIDNRWT-----------GPWANAHTNITEIQLALDLLWRNNINPNKVTMGMSFYSRSFTLTKP-SCNKPG--CRVSSAG-----NPGRCSGTTGV-------LLHAEIRDIIK------------KE-GLKP--------------VLHRDAAVKTVSW--G-----NQWASF----------------DDLVTWRLKANRFRSQCISGFMVWAMSQDDQSYTNA----QALNSALERPTMDLP-----------DFNQKDV----PADAVVESPPKL---------------------------------CRWTS----------------------------------------C-------------FE-G------------CPSGFKEIQRDG----HKEIMMDT-------TQCPGGTGH-------GFIRFCCPA-----DQ---ELPV---------------------------------------------------------------------------------------------------CT----------------WRGH-------------KN--------------------------------SG------NC----SPG-----CKTGEVEV--------------------GTVGSGCKN-N--------------------HQSACCS-------------------EGKGTAAYGKCSW---------------------------------TGCSKNP--------------------------------QEAC-------------RGFYPYFVVASSVASGG---------AKSCG--------------KSQKRSYCCAE---------------------PPPAEFSDK------------CDWHKKA-------GFLTDKQYAYV----------C-------------EGACPSG-QIRLSVESGLTV---------------------------------DGDGCYGNV---AFCCPEPAPPTTK--PSRR----------------------------------------DDFGSTQAKEFELLMQKY----------MENPTC---------------PAA-ILQPNLHDMFNGG---------------MPAKR-SIEMEAREY---------------DILRGRAINCAMDNWI---RLLSYA-------------------------------------------------------TLMF-TMNKKALDP-------------------FRKVWDSEF-------------------------------------------------AASY----------------------------DTALEYSALEGFF--------------------------------RDYPI-FDTRG--------VIEYILYNPLVAG----------------------------------------------------------------------------------------------------------------------------------------------------------AGVRRARNAR-------------------------------------------------------------EVFCE-------------------------------------------------------------------------------------------------------------------------------------------------------------------------RSSSRRSLERR--------------------------DDQGPLWARRVWAWGG--------------------------------------------------------------------------------------------------------GTNDVPGLETILEG-------------------------------------------------------------------------------------------------------------------------IRVGHLTLHYARWQYQEGQA--------------------------------------------------------TASAPGPILELAYWIGPQPG----------------------------------------------VHTEDAE-LDQYRDTTAR-NAPDDRWVVFHLHVDPT------------------------------------------------------------------REWLRRINGH---TYLG---VDYLSVYHGQEVRG--------------------------------------------------------------------ARGRYAWRVQN---------------------------------------------------IDSGYLNARDGFSCD------------SVGDGLWYVGSP-------RAVEGQEP------WYQLVQEWSEVIYNQGYVRSPGIRL--------------ILEGGNPPS-GEIDPQ-------------------------------------------DPGVLA------------------------------------------------------------------LPNT--------QTNAASGADPYTINWLITQGDRYDFFPPAPPSS-------------------------------------------------------------------------------------------------------------------------------------------------------------------------------------------------------------------------------------------------------------------------------------------------------------------------------------------------------------------------------------------------------------------------------------------------

>MR_Chit25
[truncated: 173,538 more chars]
