# Supplementary material for: Genome sequence and comparative analysis of clavicipitaceous insect-pathogenic fungus Aschersonia badia with Metarhizium spp
Source: BMC Genomics. 2016 May 17;17:367. doi: 10.1186/s12864-016-2710-6 (PMC4869207; doi:10.1186/s12864-016-2710-6)
Supplement: Additional file 3: — Phylogeny and comparative genomics of clavicipitaceous fungi Ab, MAC, and MR. (PDF 1319 kb) [file 12864_2016_2710_MOESM3_ESM.pdf]

Additional File 3.

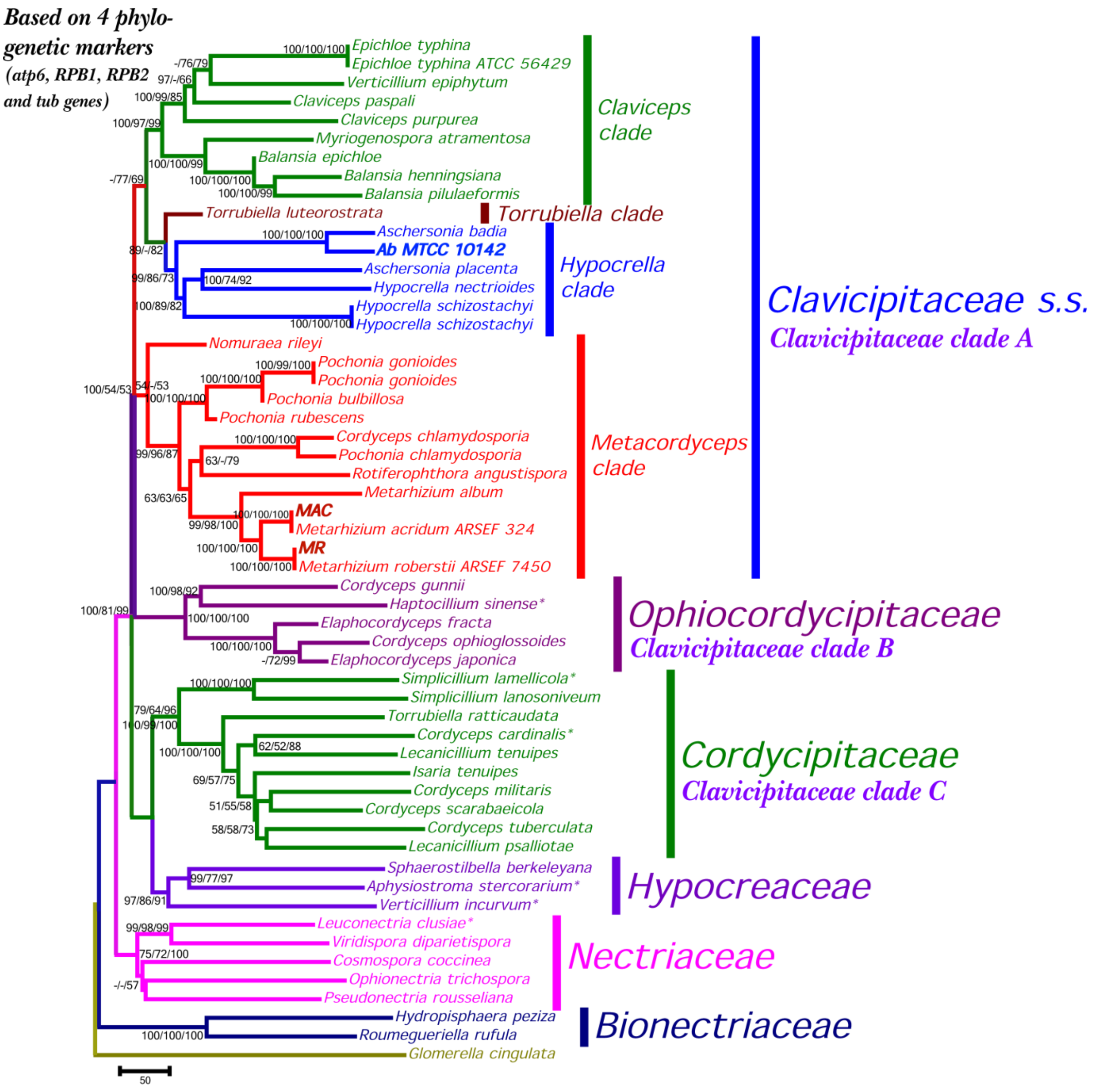

Figure S1: Phylogenetic relationships of clavicipitaceous fungi based on concatenated mitochondrial *atp6*, *RPB1*, *RPB2* and *β-tubulin* genes. The series of values over the branches corresponds to ML, MP and NJ bootstrap values (>50%). * indicates the type strain. The phylogenetic tree is drawn to scale where the evolutionary distances were in the units of the number of base differences per sequence.

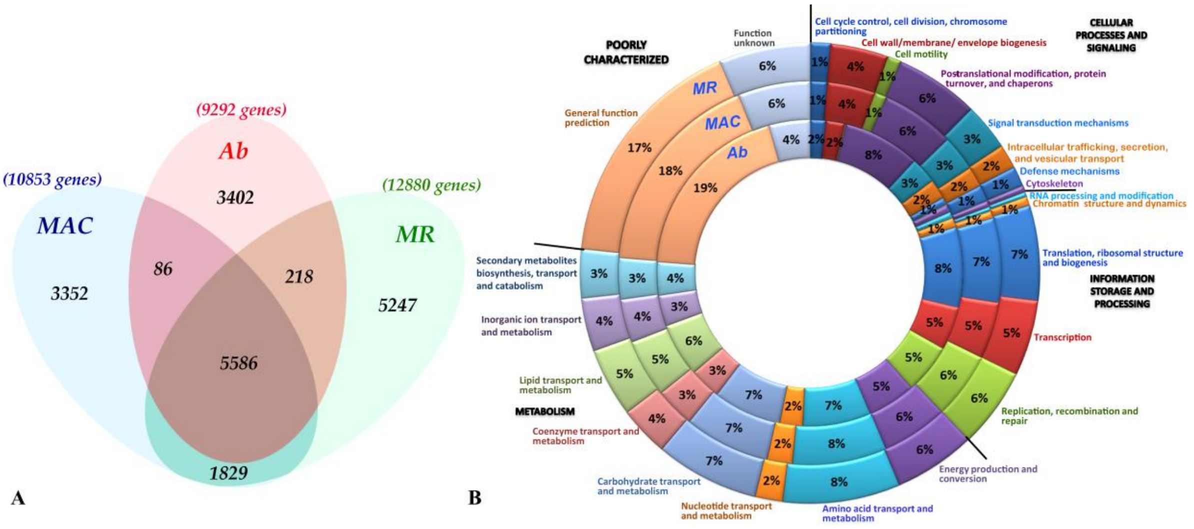

Figure S2: Comparative genome analysis of *Ab, MAC* and *MR*. (A) Distribution of orthologs and non-orthologs. Each oval represents distinct fungal species, *Ab* (red), *MAC* (blue) and *MR* (green). Shared region displays the orthologs between the sets of genomes. 5586 genes represent the core gene pool, shared by all three fungal genomes. Unique genes of each species are represented by the unshared region. **(B) Functional annotation of predicted proteins.** Functional categorization was assigned using COG database, showing percent-wise distribution of predicted proteins in each COG category. Black lines are used as a marker to delineate between the four summarized functional annotation categories.

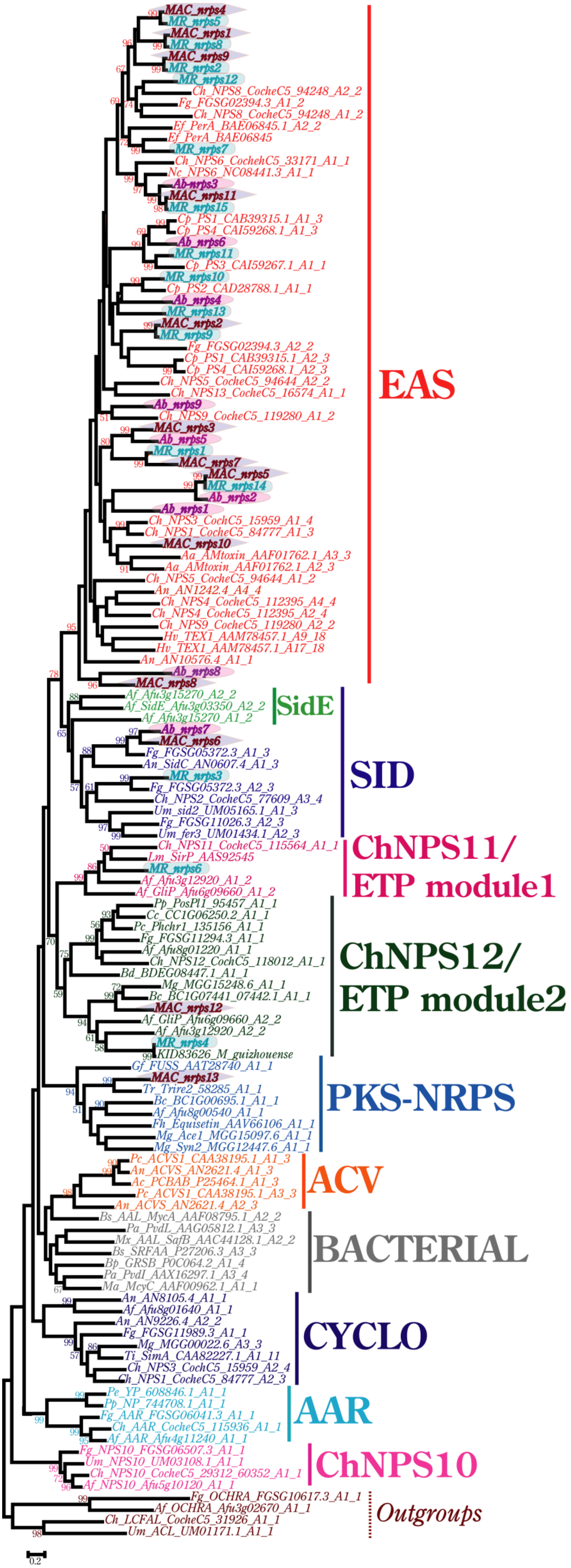

MAC_nrps4
MR_nrps5
MAC_nrps1
MR_nrps8
MAC_nrps9
MR_nrps2
MR_nrps12
Ch_NPS8_CocheC5_94248_A2_2
Fg_FGSG02394.3_A1_2
Ch_NPS8_CocheC5_94248_A1_2
Ef_PerA_BAE06845.1_A2_2
Ef_PerA_BAE06845
MR_nrps7
Ch_NPS6_CochehC5_33171_A1_1
Nc_NPS6_NC08441.3_A1_1
Ab_nrps3
MAC_nrps11
MR_nrps15
Cp_PS1_CAB39315.1_A1_3
Cp_PS4_CAI59268.1_A1_3
Ab_nrps6
MR_nrps11
Cp_PS3_CAI59267.1_A1_1
MR_nrps10
Cp_PS2_CAD28788.1_A1_1
Ab_nrps4
MR_nrps13
MAC_nrps2
MR_nrps9
Fg_FGSG02394.3_A2_2
Cp_PS1_CAB39315.1_A2_3
Cp_PS4_CAI59268.1_A2_3
Ch_NPS5_CocheC5_94644_A2_2
Ch_NPS13_CocheC5_16574_A1_1
Ab_nrps9
Ch_NPS9_CocheC5_119280_A1_2
MAC_nrps3
Ab_nrps5
MR_nrps1
MAC_nrps7
MAC_nrps5
MR_nrps14
Ab_nrps2
Ab_nrps1
Ch_NPS3_CochC5_15959_A1_4
Ch_NPS1_CocheC5_84777_A1_3
MAC_nrps10
Aa_AMtoxin_AAF01762.1_A3_3
Aa_AMtoxin_AAF01762.1_A2_3
Ch_NPS5_CocheC5_94644_A1_2
An_AN1242.4_A4_4
Ch_NPS4_CocheC5_112395_A4_4
Ch_NPS4_CocheC5_112395_A2_4
Ch_NPS9_CocheC5_119280_A2_2
Hv_TEX1_AAM78457.1_A9_18
Hv_TEX1_AAM78457.1_A17_18
An_AN10576.4_A1_1
Ab_nrps8
MAC_nrps8
EAS
Af_Afu3g15270_A2_2
Af_SidE_Afu3g03350_A2_2
Af_Afu3g15270_A1_2
SidE
Ab_nrps7
MAC_nrps6
Fg_FGSG05372.3_A1_3
An_SidC_AN0607.4_A1_3
MR_nrps3
Fg_FGSG05372.3_A2_3
Ch_NPS2_CocheC5_77609_A3_4
Um_sid2_UM05165.1_A1_3
Fg_FGSG11026.3_A2_3
Um_fer3_UM01434.1_A2_3
SID
Ch_NPS11_CocheC5_115564_A1_1
Lm_SirP_AAS92545
MR_nrps6
Af_Afu3g12920_A1_2
Af_GliP_Afu6g09660_A1_2
ChNPS11/
ETP module1
Pp_PosPl1_95457_A1_1
Cc_CC1G06250.2_A1_1
Pc_Phchr1_135156_A1_1
Fg_FGSG11294.3_A1_1
Af_Afu8g01220_A1_1
Ch_NPS12_CochC5_118012_A1_1
Bd_BDEG08447.1_A1_1
Mg_MGG15248.6_A1_1
Bc_BC1G07441_07442.1_A1_1
MAC_nrps12
Af_GliP_Afu6g09660_A2_2
Af_Afu3g12920_A2_2
MR_nrps4
KID83626_M_guizhouense
ChNPS12/
ETP module2
Gf_FUSS_AAT28740_A1_1
MAC_nrps13
Tr_Trire2_58285_A1_1
Bc_BC1G00695.1_A1_1
Af_Afu8g00540_A1_1
Fh_Equisetin_AAV66106_A1_1
Mg_Ace1_MGG15097.6_A1_1
Mg_Syn2_MGG12447.6_A1_1
PKS-NRPS
Pc_ACVS1_CAA38195.1_A1_3
An_ACVS_AN2621.4_A1_3
Ac_PCBAB_P25464.1_A1_3
Pc_ACVS1_CAA38195.1_A3_3
An_ACVS_AN2621.4_A2_3
ACV
Bs_AAL_MycA_AAF08795.1_A2_2
Pa_PvdL_AAG05812.1_A3_3
Mx_AAL_SafB_AAC44128.1_A2_2
Bs_SRFAA_P27206.3_A3_3
Bp_GRSB_P0C064.2_A1_4
Pa_PvdI_AAX16297.1_A3_4
Ma_McyC_AAF00962.1_A1_1
BACTERIAL
An_AN8105.4_A1_1
Af_Afu8g01640_A1_1
An_AN9226.4_A2_2
Fg_FGSG11989.3_A1_1
Mg_MGG00022.6_A3_3
Ti_SimA_CAA82227.1_A1_11
Ch_NPS3_CochC5_15959_A2_4
Ch_NPS1_CocheC5_84777_A2_3
CYCLO
Pe_YP_608846.1_A1_1
Pp_NP_744708.1_A1_1
Fg_AAR_FGSG06041.3_A1_1
Ch_AAR_CocheC5_115936_A1_1
Af_AAR_Afu4g11240_A1_1
AAR
Fg_NPS10_FGSG06507.3_A1_1
Um_NPS10_UM03108.1_A1_1
Ch_NPS10_CocheC5_29312_60352_A1_1
Af_NPS10_Afu5g10120_A1_1
ChNPS10
Fg_OCHRA_FGSG10617.3_A1_1
Af_OCHRA_Afu3g02670_A1_1
Ch_LCFAL_CocheC5_31926_A1_1
Um_ACL_UM01171.1_A1_1
Outgroups
0.2

Figure S3: ML phylogenetic tree of NRPS gene types. Bootstrap support (>50%) is shown over the branches. NRPS gene sequences obtained from *Ab*, *MAC* and *MR* genomes are highlighted and shaded. The evolutionary tree is drawn to scale with distances in the units of the number of amino acid substitutions per site. EAS (Euascomycete clade synthetases) and SID (siderophore synthetases) subclasses are multimodular. The remaining are mono/bi-modular NRPS types, where fungal NRPS from the three genomes clustered in ChNPS11/ETP module 1 toxin-like synthetases, ChNPS12/ETP module 2 toxin-like synthetases and PKS-NRPS hybrid synthetases.
